# Latent resistance mechanisms of steel truss bridges after critical failures

**DOI:** 10.1038/s41586-025-09300-8

**Published:** 2025-09-03

**Authors:** Juan C. Reyes-Suárez, Manuel Buitrago, Brais Barros, Safae Mammeri, Nirvan Makoond, Carlos Lázaro, Belén Riveiro, Jose M. Adam

**Affiliations:** 1 ICITECH, Universitat Politècnica de València, Valencia, Spain; 2 CINTECX, Universidade de Vigo, Applied Geotechnologies Research Group, Vigo, Spain; 3 Departamento de Mecánica de los Medios Continuos y Teoría de Estructuras, Universitat Politècnica de València, Valencia, Spain

**Keywords:** Engineering, Civil engineering

## Abstract

Steel truss bridges are constructed by connecting many different types of bars (components) to form a load-bearing structural system. Several disastrous collapses of this type of bridge have occurred as a result of initial component failure(s) propagating to the rest of the structure^1–3^. Despite the prevalence and importance of these structures, it is still unclear why initial component failures propagate disproportionately in some bridges but barely affect functionality in others^4–7^. Here we uncover and characterize the fundamental secondary resistance mechanisms that allow steel truss bridges to withstand the initial failure of any main component. These mechanisms differ substantially from the primary resistance mechanisms considered during the design of (undamaged) bridges. After testing a scaled-down specimen of a real bridge and using validated numerical models to simulate the failure of all main bridge components, we show how secondary resistance mechanisms interact to redistribute the loads supported by failed components to other parts of the structure. By studying the evolution of these mechanisms under increasing loads up to global failure, we are able to describe the conditions that enable their effective development. These findings can be used to enhance present bridge design and maintenance strategies, ultimately leading to safer transport networks.

## Abstract (web summary)

Experimental tests and computational simulations of a scaled-down specimen of a real steel truss bridge identify and characterize the latent resistance mechanisms following critical failures, demonstrating how loads supported by failed components can be redistributed and enable structure resilience.

## Main

Advancements in steel production and the efficiency of truss designs fuelled a surge in the construction of steel truss bridges during the late nineteenth and early twentieth centuries^8^. Many bridges from that period are still in operation^9^ (usually carrying higher vehicular loads than those considered when they were conceived^10,11^) and this form of construction continues to be used today. Owing to their strategic importance, the failure of these bridges can have huge negative consequences, including fatalities and economic losses that amount to millions of dollars per day of closure^12,13^. As we face increasingly intense and erratic natural hazards that are able to cause severe damage^14–17^, as well as environmental changes that accelerate deterioration processes^18–20^, it is arguably more important than ever to ensure that these structures are sufficiently robust to withstand the initial failure of any one of its numerous components. This is particularly important considering that steel truss bridges are typically designed to operate for at least 100 years (ref. ^21^).

Similar to the way spider webs are able adapt (to continue trapping prey) after suffering damage^22,23^, the loads supported by failed components in steel truss bridges can travel to other parts of the structure through alternative load paths (ALPs), thus preventing collapse thanks to effective load redistribution^24–29^. Owing to morphological changes to the truss structure after initial component failures^30^, the secondary resistance mechanisms that enable ALPs to form will differ completely from the primary resistance mechanisms through which the undamaged structure resists its operational loads.

In the case of buildings, the secondary resistance mechanisms that can develop are now well understood thanks to decades of research^31^. For instance, after suffering the loss of a column, it is well known that building structures can redistribute loads through a variety of mechanisms, including Vierendeel action of frames^32^, as well as flexural action, compressive arching and catenary action of beams^33,34^. On the other hand, although the primary resistance mechanisms of steel truss bridges are well understood and used during structural design, the secondary resistance mechanisms they can develop after initial failures are still very much unknown.

To design or assess bridges for robustness, it is first necessary to identify and characterize these secondary resistance mechanisms. Reliably achieving this requires experimental data on the response of bridges after initial failures, which are extremely scarce because they are almost impossible to obtain from in-service bridges and are expensive to reproduce in the laboratory^28,29^.

Here we present the outcomes of a research programme that allowed us to uncover the fundamental secondary resistance mechanisms of steel truss bridges and how they can contribute to residual load-bearing capacity after initial component failures. This research involved experimentally testing a scaled steel truss bridge specimen and analysing damage scenarios involving the loss of all main components using validated computational simulations.

## Testing and analysing failure scenarios

**Fig. 1:**
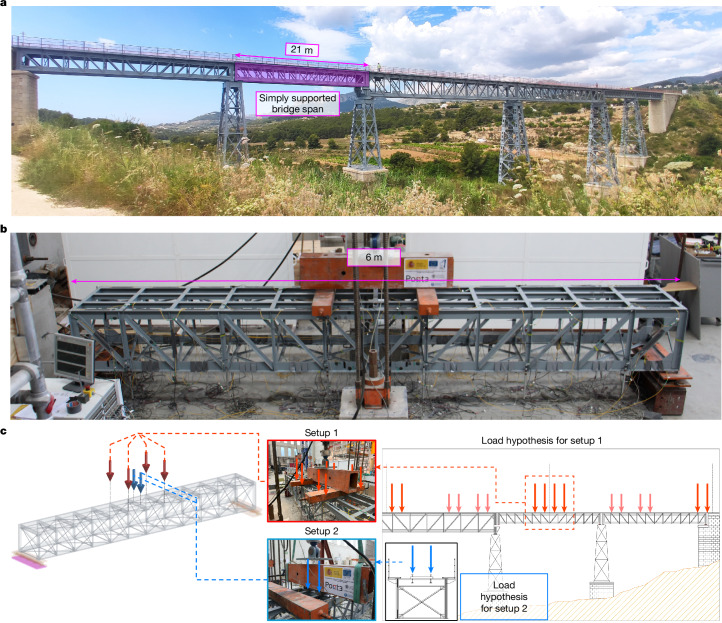
Specimen design and experimental test protocol. **a**, Real bridge span used as a reference for creating the scaled-down specimen. **b**, Scaled-down bridge specimen prepared for testing in the laboratory. **c**, Definition of loading setup for tests based on unfavourable operational loads. A different loading setup sometimes had to be used depending on the nature of the component whose initial failure was being studied.

**Extended Data Fig. 1:**
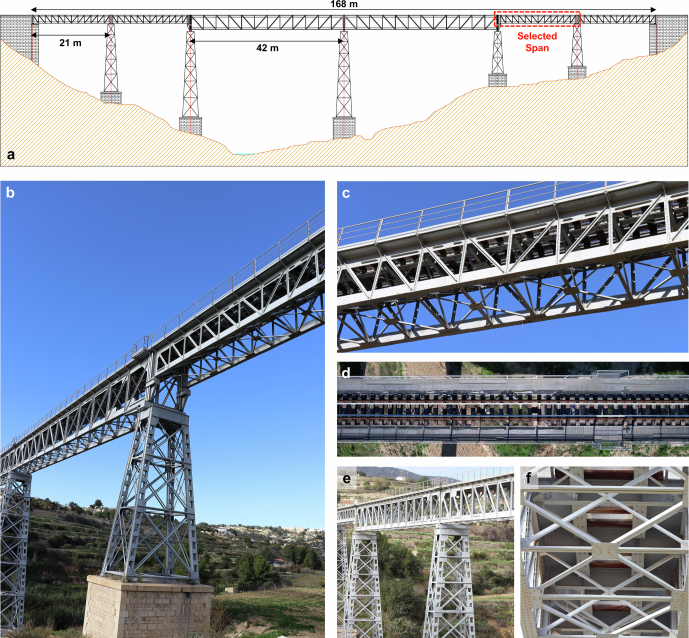
Real railway bridge used as a reference. **a**, Schematic front elevation of complete bridge showing span dimensions. **b**, View of a pier at which isostatic and hyperstatic spans meet. **c**, Isostatic span. **d**, Top view showing rails. **e**, Hyperstatic spans. **f**, Horizontal and vertical bracings.

**Extended Data Fig. 2:**
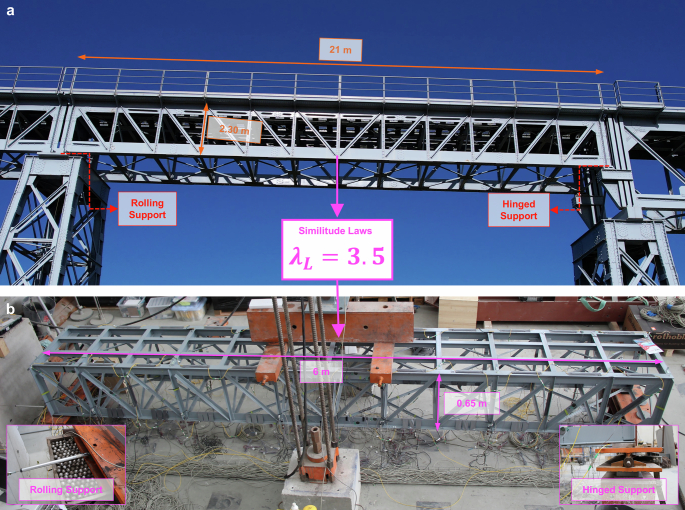
Scaling an isostatic span of the real bridge. **a**, Isostatic span of the real bridge. **b**, Scaled-down specimen using a scale factor (*λ*_L_) of 3.5 for member lengths.

**Extended Data Fig. 3:**
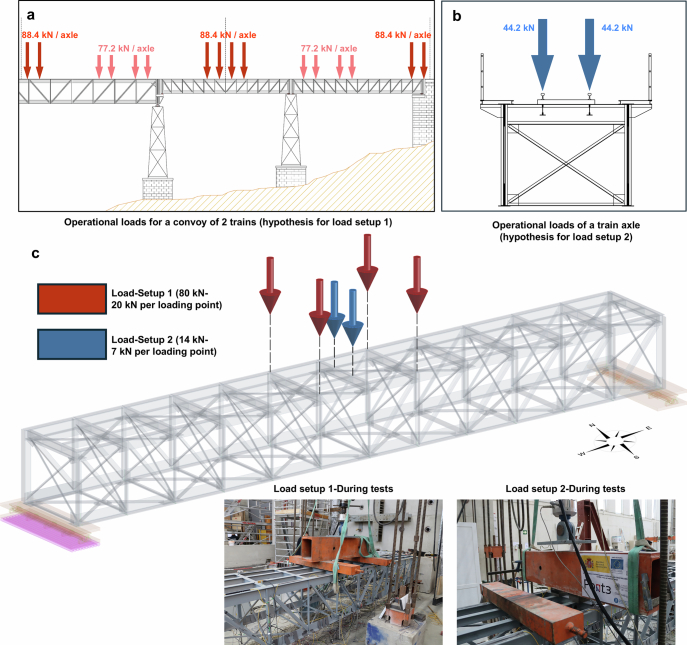
Design and implementation of loading setup. **a**, Hypothesis for load setup 1: considering two attached trains and the effect of several axles applying the maximum load on the centre span of the trusses, activating the main system. **b**, Hypothesis for load setup 2: considering the effect of one axle on the flooring system. **c**, Load setups used in the experimental campaign and related photographs.

To ensure that results from the research are directly applicable to as many cases as possible, a scaled-down laboratory specimen was designed on the basis of one of the most common types of steel truss bridge. Specifically, an isostatic span of a real railway bridge built with Pratt trusses^35,36^ was used as the basis to define the specimen (Fig. 1a,b, Methods section ‘Specimen design’, Extended Data Figs. 1 and 2, Supplementary Video 1 and Supplementary Information Section 1). The reference loading setups used for testing were also derived from the most unfavourable operational loads of the full-scale bridge (Fig. 1c, Methods section ‘Specimen design’ and Extended Data Fig. 3).

It should be noted that such scaled-down tests are an effective way of studying the onset and development of ALPs in this type of structure because the steel material experiences notable plastic deformation before failure, which scales more uniformly compared with brittle fracture^37^. The component dimensions and imposed loads were consistently scaled according to similitude laws derived from dimensional analysis^38–41^ to ensure that the scaled-down tests accurately represent the failure of components owing to yielding and member buckling (Methods section ‘Specimen design’ and Supplementary Information Section 1).

**Fig. 2:**
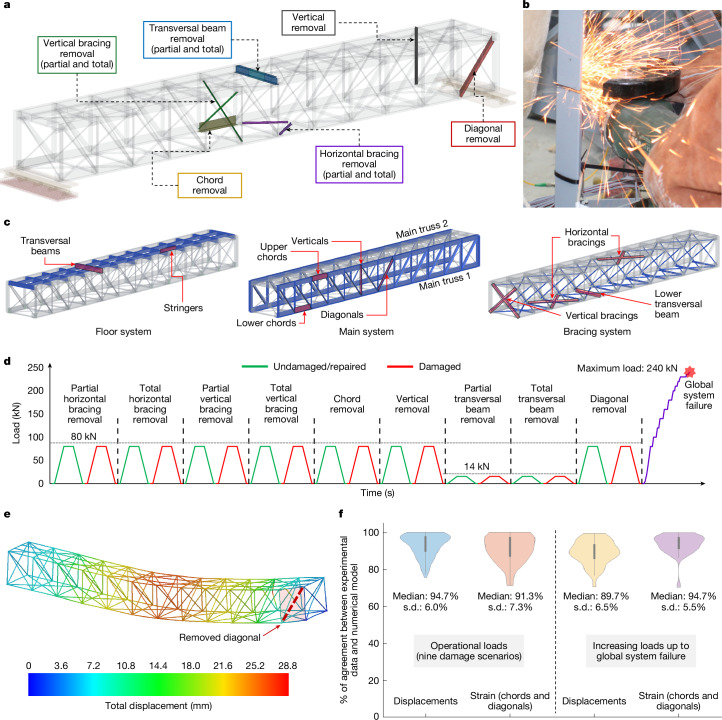
Damage scenarios tested and validation of computational simulations. **a**, Locations of the nine damage scenarios tested experimentally. **b**, Reproducing the loss of a component by cutting it. **c**, Classification of components according to their load-bearing function. **d**, Complete experimental protocol. **e**, Simulation of a damage scenario. **f**, Level of agreement between simulation predictions and experimental records (computed as 1 − the relative error). Distributions are shown for displacements and strains using bounded kernel density plots. Source Data

**Extended Data Fig. 4:**
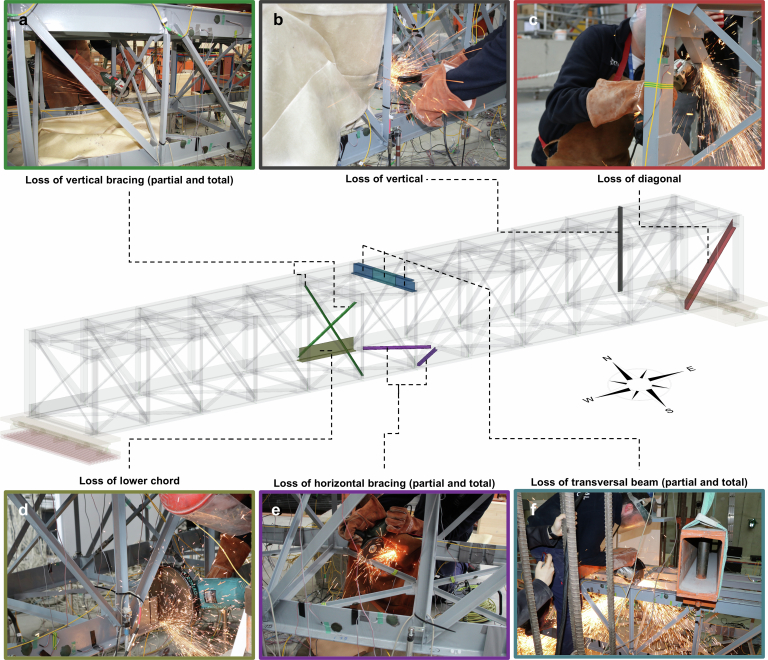
Tested damage scenarios. **a**, Loss of vertical bracing (partial and total). **b**, Loss of vertical. **c**, Loss of diagonal. **d**, Loss of lower chord. **e**, Loss of horizontal bracing (partial and total). **f**, Loss of transversal beam (partial and total).

Nine initial damage scenarios, each involving the failure of a single component, were tested in the laboratory (Fig. 2a, Extended Data Fig. 4 and Supplementary Video 2). In each case, the targeted component was fully severed across its cross-section to simulate failure at a specific location (Fig. 2b). The scenarios were chosen to capture the failure of components with unique structural functions in the three load-transfer systems of the bridge, namely the floor system, the main system and the bracing system (Fig. 2c). As a result, the study covers the failure of any type of main component in the entire bridge. The specific locations were selected because they correspond to those for which each type of component sustains very high internal loads under operational conditions (Fig. 2a).

**Extended Data Fig. 5:**
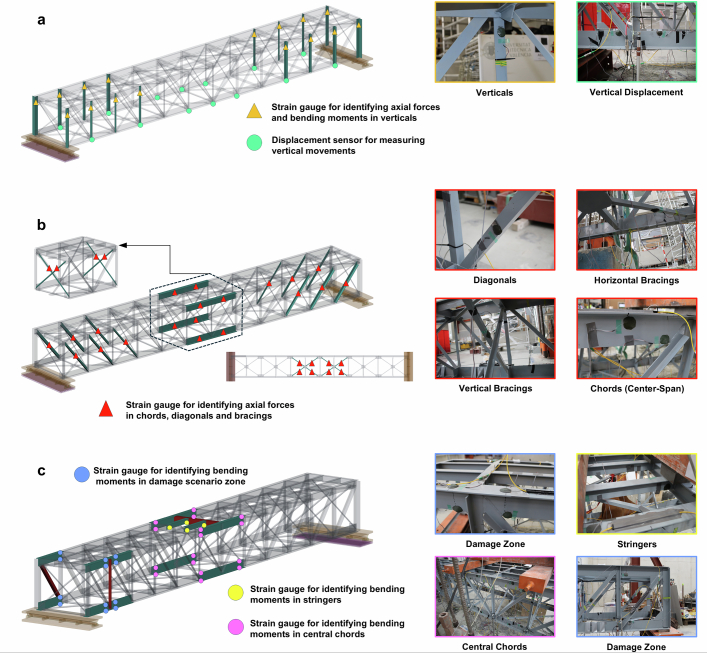
Distribution of sensor types and their locations on the scaled bridge. **a**, Location of the displacement sensors on the lower chord joints for vertical displacement measurement and strain gauges on the upper part of the verticals for axial force and bending moment identification. **b**, Strain gauges on chords, diagonals and bracings for axial force identification. **c**, Strain gauges on chord ends and stringers for bending moment identification.

Each test consisted of imposing the reference test load on the bridge specimen. After each test, the damaged component was fully repaired and the structural response of the repaired structure under the effect of the reference test load was recorded (Fig. 2d). This allowed performing a benchmark comparison for each scenario between the responses of damaged and undamaged states. It should also be noted that, under the reference operational loads used for testing, none of the components of the bridge specimen ever entered the nonlinear regime (in which the material experiences irreversible deformations). This ensured that subsequent tests were not affected by previous ones. For the final damage scenario tested, the imposed load was gradually increased until global system failure (collapse) could be observed. During all of these tests, the structural response was closely monitored using 80 strain gauges and 14 displacement transducers (Extended Data Fig. 5 and Supplementary Information Section 1).

**Extended Data Fig. 6:**
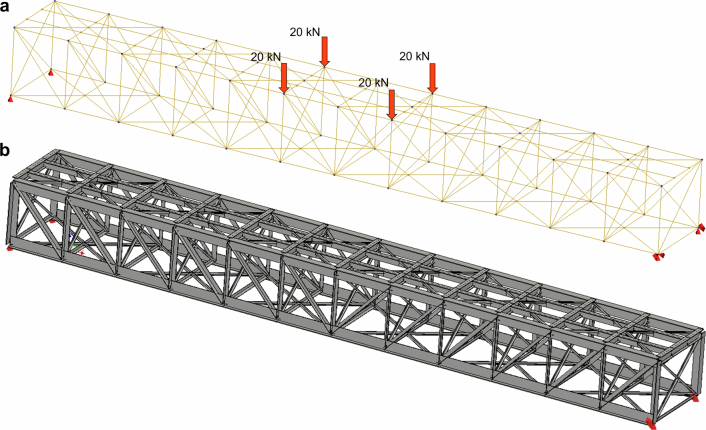
The nonlinear finite element model of the scaled bridge implemented in DIANA FEA software. **a**, Unifilar representation of the beam elements used for modelling the bridge, together with the applied loads. **b**, Visualization of the model with extruded profiles.

The measured structural response during all tests was used to validate a nonlinear finite element model of the bridge specimen (Fig. 2e,f, Methods sections ‘Experiment and monitoring design’ and ‘Simulations for studying the onset of ALPs’, Extended Data Fig. 6, Supplementary Information Sections 2 and 3 and Supplementary Video 3). This validated model was used to simulate the loss of main components from all possible locations in the bridge (not tested experimentally), allowing any location-dependent trends in the activation of secondary resistance mechanisms to be studied. A total of 222 damage scenarios were simulated during this process.

**Extended Data Fig. 7:**
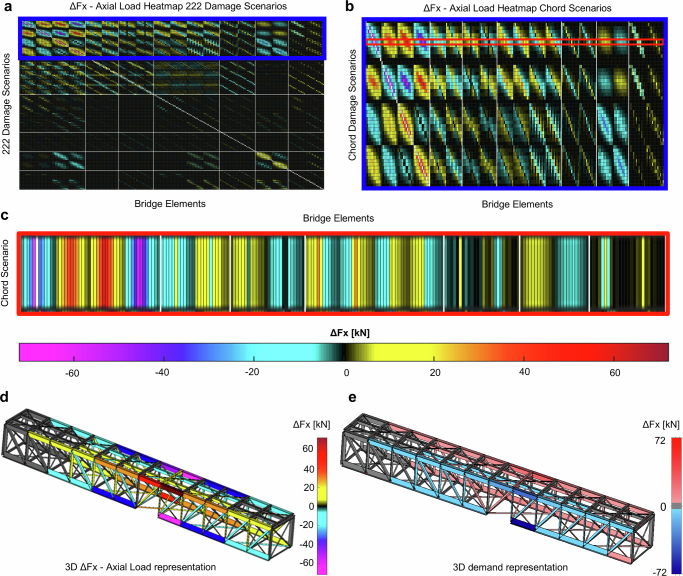
Example of results for the analysis of the axial force increments (Δ*Fx*) between damaged and undamaged states for a damage scenario. **a**, Heat map considering the 222 damage scenarios in which the chord damage scenarios are highlighted in blue. **b**, Heat map of the chord damage scenarios in which the specific lower chord damage scenario is highlighted in red. **c**, Heat map of the specific lower chord damage scenario. **d**, Representation of the results on the bridge. **e**, Conversion of the results in terms of axial demand increase or decrease with respect to the original demand and representation on the bridge. Source Data

Structural response and load redistribution were analysed on the basis of carefully defined performance indicators. These were defined in terms of changes in displacements, axial loads, bending moments and support reactions (Methods section ‘Simulations for studying the onset of ALPs’ and Supplementary Information Section 2). The computed performance indicators were systematically analysed for each type of initial component failure, with the aim of identifying and characterizing the common underlying secondary resistance mechanisms that can prevent collapse (Extended Data Fig. 7).

## The first line of defence

**Fig. 3:**
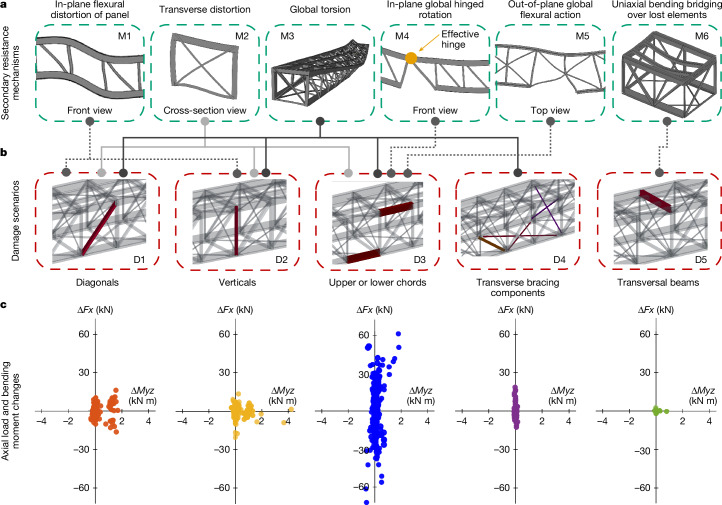
Secondary resistance mechanisms activating ALPs. **a**, Six fundamental secondary resistance mechanisms that interact to enable load redistribution after initial component failures. **b**, Different possible types of initial component failure (damage scenarios). **c**, Changes in axial loads and bending moments in all components of the bridge span for all simulated damage scenarios of each type. Source Data

The structural response of the steel truss bridge specimen across an extensive spectrum of possible damage states was analysed in detail based on the data collected by sensors during experimental testing and on the results of the computational simulations of 222 damage scenarios (Methods section ‘Simulations for studying the onset of ALPs’ and Supplementary Information Section 2). We found that, after the failure of any main component, the structural system always relied on a subset from six fundamental resistance mechanisms to redistribute loads to other parts of the system and thus prevent collapse (Fig. 3a). The specific mechanisms that are activated and the nature of their interaction depend on the type of initial component failure triggering load redistribution (Fig. 3a,b).

One of the simplest ways in which the structure resists collapse is through the in-plane flexural distortion of the panels (Fig. 3a, M1) in one of the two main trusses (Fig. 2c) of the bridge. This two-dimensional mechanism causes marked bending of the chords and verticals or diagonals in the panel(s) that enclose a failed component, with the highest bending stresses occurring near their connections. Naturally, this form of response is only possible if the connections between components have sufficient capacity to transfer bending moments. Another important deformation mode that also imposes notable bending stresses in the involved components is the transverse distortion of panels making up the cross-section of the bridge in the region around an initial component failure (Fig. 3a, M2). This mechanism helps transfer loads from the damaged main truss on one side of the bridge to the undamaged one on the opposite side. This is typically a localized response that only affects a few transverse panels in modules close to the failed component. Otherwise, one of the most prevalent load-redistribution behaviours that was observed involves the activation of a global torsion mechanism (Fig. 3a, M3). This phenomenon twists the entire structural system, resulting in asymmetric reactions at the supports of the bridge span. Because all components participate interdependently in this global twisting motion, it tends to change the axial stresses in individual components much more than their bending stresses (Supplementary Information Section 2). If an initial component failure greatly reduces the flexural stiffness of the bridge at a specific location along its length, the global in-plane hinged rotation mechanism (Fig. 3a, M4) plays a crucial role in allowing the structure to continue sustaining gravity loads. The localized reduction in flexural stiffness tends to concentrate bending stresses around specific connections in its vicinity. This effectively creates a hinge at that location (Fig. 3a, M4), causing parts of the bridge structure on either side of it to rotate in a similar manner as rigid bodies. If an initial component failure creates substantial asymmetry between the flexural stiffness of the two main trusses, the entire cross-section of the bridge can also bend out of plane (Fig. 3a, M5). This response helps alleviate the load-redistribution burden carried by other secondary resistance mechanisms. Finally, the simplest mechanism that can prevent collapse is the uniaxial bending of adjacent components that directly bridge over a failure (for example, bending of stringers after the failure of a transversal beam; Fig. 3a, M6).

After the failure of verticals or diagonals (Fig. 3b, D1 and D2), the structural system relies mainly on (the relatively local) flexural distortion mechanisms of panels in the two principal planes of the bridge to prevent collapse (Fig. 3a, M1 and M2). Although the global torsion mechanism (Fig. 3a, M3) is also noticeable, its role in load redistribution is small compared with the flexural mechanisms, as evidenced by the fact that bending moment changes are more notable than axial load changes after these failures (Fig. 3c). On the other hand, after chord failures (Fig. 3b, D3), load transfer from the damaged to the undamaged main truss occurs primarily through the global torsion mechanism, as evidenced by the large axial load increases in undamaged components of the bridge (Fig. 3c). Given that chords contribute substantially to the flexural stiffness of the bridge (as part of their primary function), both global-in-plane hinged rotation (Fig. 3a, M4) and out-of-plane global flexural action (Fig. 3a, M5) activate to prevent collapse after their failure. Transverse bracing components clearly play a key role in transverse distortion, global torsion and out-of-plane global flexural mechanisms after the failure of main truss components (Fig. 3b, D1–D3). However, their own failure (Fig. 3b, D4) is less critical, as shown by the smaller magnitude of redistributed loads that they produce (Fig. 3c). In these cases, collapse is prevented through the activation of (less pronounced) global torsion mechanisms. Similarly, after the failure of transversal beams of the floor system (Fig. 3b, D5), redistributed loads tend to be very small (Fig. 3c) and the simple uniaxial flexural mechanisms (Fig. 3a, M6) alone can prevent collapse. Nevertheless, although failure of floor-system components is less important for the structural integrity of the bridge as a whole, it remains critical for operation owing to the high risk it poses for the derailment of trains or the disruption of vehicle traffic. In summary, steel truss bridges may have an extraordinary capacity to withstand the failure of any single main component thanks to the activation of six fundamental secondary resistance mechanisms.

## The evolution of ALPs

**Fig. 4:**
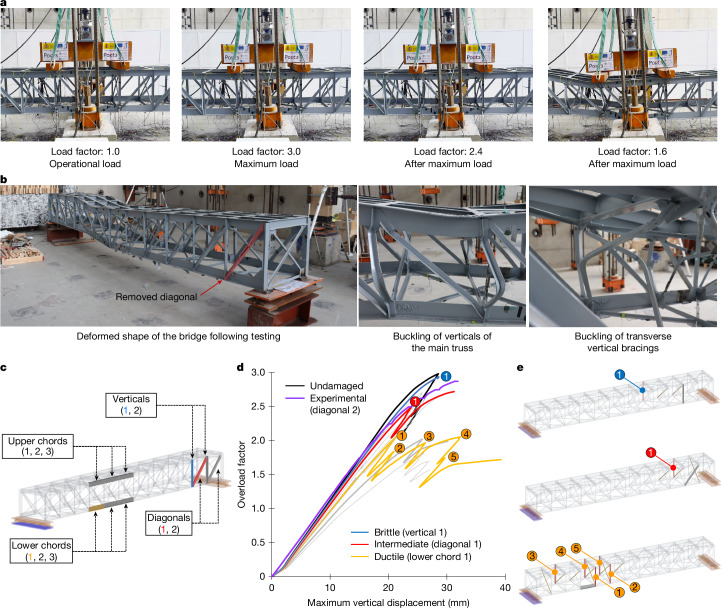
The evolution of secondary resistance mechanisms. **a**, Final experimental test in which the imposed load on the bridge specimen was increased until global system failure (collapse). **b**, Detailed view of damage and buckling failures occurring during the final experimental test. **c**, Ten initial component failures (damage scenarios) considered for study on the evolution of ALPs. **d**, Overload factor (applied load divided by standard test load of 80 kN) plotted against maximum vertical displacement for the undamaged bridge and for all damage scenarios. A representative curve has been highlighted for each type of global failure (brittle, intermediate and ductile). The corresponding curve is also shown for the experimental test until buckling of the first (main) vertical. **e**, Verticals and vertical bracings that buckled during failure propagation (before collapse). Number labels indicate the failure of verticals that correspond to the limit points shown in panel **d**. Source Data

A final experimental test was performed to assess the residual load-bearing capacity of the bridge and to study the evolution of ALPs in more unfavourable situations (for example, owing to higher vehicular loads that can arise over the service life of bridges^10^). This test involved increasing the load imposed on the damaged bridge specimen until global system failure (collapse) occurred (Figs. 2d and 4a and Supplementary Video 4). Specifically, the damage scenario tested under these conditions involved the failure of one of the main diagonals (Fig. 4b). Computational simulations were validated on the basis of this test (Fig. 2e,f) and used to extend the study on ALP evolution to a total of ten damage scenarios (Fig. 4c). The type and location of initial component failures considered in these scenarios were chosen to ensure that this study (under increasing loads) provides representative coverage of the possible combinations of secondary resistance mechanisms that can prevent collapse (Methods section ‘Simulations for studying the evolution of ALPs’ and Supplementary Information Section 3).

The results show that, even when damaged, the bridge still has a very high residual capacity of between 1.8 and 3.0 times the operational load (overload factor shown in Fig. 4d), thus demonstrating its remarkable structural robustness. Initially, the structure relies on the same secondary resistance mechanisms described in the previous section to redistribute the increasing imposed loads. This response changes when a second component gives way (by buckling) and triggers a series of consecutive buckling events that eventually lead to collapse (Supplementary Information Section 3).

**Extended Data Fig. 8:**
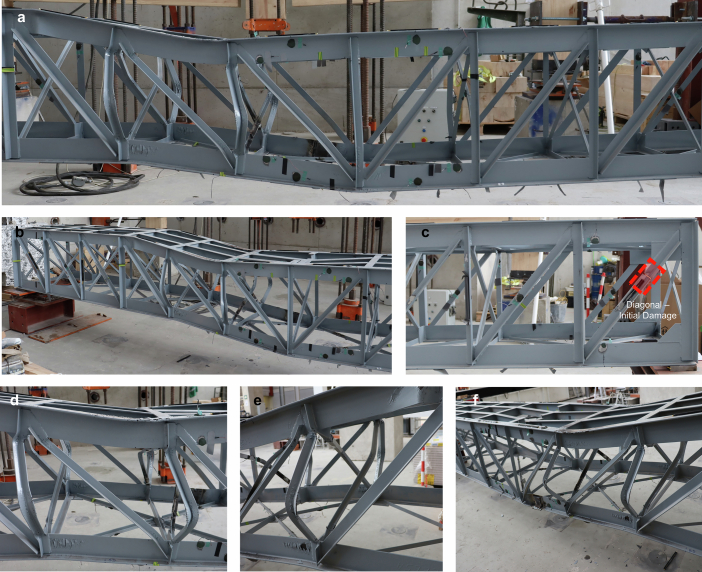
The state of the bridge after the collapse test. **a**, General frontal view. **b**, View of the western support and of the zone in which most failed elements were located. **c**, View of the zone close to the initial damage on the diagonal. **d**–**f**, Detailed views of the failed elements.

The components that tend to buckle during failure propagation are either (main) verticals or (transverse) vertical bracings (Fig. 4b,e). During this process, the failure of vertical bracings will typically have relatively small effects that only change the overall stiffness of the structural system. On the other hand, failures of verticals mark clear limit points in the global (load–displacement) response of the system (Fig. 4d,e). Although this phenomenon leads to complex structural response patterns, these always tend to represent superpositions of the previously described secondary resistance mechanisms that change depending on the type and location of buckled components (Extended Data Fig. 8 and Supplementary Information Section 3). As a result, just as the behaviour of a damaged spider web depends on the type of thread that has failed^23^, the response and residual capacity of the damaged steel truss bridge depend on the original function of the (initially) failed component(s). In the case of steel truss bridges, our results indicate that their structural response after an initial component failure can be classified as follows: (1) brittle global failure modes, as occurs after the failure of upper chords or verticals (Fig. 4d); (2) ductile global failure modes, as occurs after the failure of lower chords (Fig. 4d); and (3) intermediate global failure modes, as occurs after the failure of diagonals (Fig. 4d). Notably, the nature of the load sustained by a component in the undamaged structure (which is redistributed as it fails) clearly plays a decisive role in determining the global failure mode. Specifically, failures of components that initially carry high compression loads (such as upper chords or verticals) tend to cause brittle system failures, whereas failures of components that initially sustain high tensile loads (such as lower chords) lead to more ductile system failure modes.

In general, these findings allow us to identify the initial component failures that have the greatest negative impact on residual load-bearing capacity (that is, lower chords) and global system ductility (that is, verticals and upper chords).

## Discussion and future outlook

This work reveals the fundamental types of secondary resistance mechanism that are activated in steel truss bridges to prevent collapse after the loss of any main component. By studying the evolution of ALPs that form through these mechanisms at different stages of failure propagation, we have also shown that damaged steel truss bridges may still be capable of withstanding loads that are much higher than their present operational loads.

It should be mentioned that, in the case of building structures, characterization of such secondary resistance mechanisms has transformed the way that buildings are designed to ensure their robustness, with notable changes made to building codes all around the world^42–45^. In fact, this understanding has also changed the way that existing buildings are assessed^46,47^ and strengthened^48–50^. The same understanding that has now been brought about for steel truss bridges could therefore enable an equally impactful shift in the field of bridge engineering. Design principles for new bridges can now be revised to maximize their ability to develop secondary resistance mechanisms, whereas assessment and retrofitting strategies can be improved to target key locations for the activation of these mechanisms. This will enhance the robustness of steel truss bridges and thus help ensure the safety of transport networks.

## Methods

### Specimen design

We performed experimental tests on a scaled specimen that reproduces one of the simply supported spans of a real railway bridge (Supplementary Video 1). The considered simply supported span (Extended Data Fig. 1) consists of two lateral Pratt trusses (the main system) connected by vertical transverse X-bracings, horizontal upper and lower X-bracings and lower transverse beams (the bracing system), as well as upper transversal floor beams with longitudinal rail beams (that is, stringers) joined to them (the floor system).

To accurately reproduce structural behaviour, load redistribution and modes of failure, we designed the scaled bridge specimen using the following scaling criteria based on dimensional analysis^38,39^:

1. The model is made of steel with a yield strength *f*_y_ = 275 MPa (S275 according to EN 10025-1:2004 (ref. ^51^)). The density and mechanical properties of the steel of the actual bridge are practically the same as the density and mechanical properties of S275 steel. Therefore, all scaling factors related to the material (density: *λ*_*ρ*_, Young’s modulus: *λ*_*E*_, Poisson’s ratio: *λ*_*ν*_) are taken equal to one.
2. A scale factor *λ*_L_ = 3.5 has been chosen for member lengths. Cross-section areas and moments of inertia have been scaled accordingly (${\lambda }_{{\rm{A}}}={\lambda }_{{\rm{L}}}^{2}$, ${\lambda }_{{\rm{I}}}={\lambda }_{{\rm{L}}}^{4}$) (Supplementary Information Section 1).
3. Assuming that the scale of forces is *λ*_F_, measured displacements of the model scale according to ${\lambda }_{{\rm{d}}}={\lambda }_{{\rm{F}}}\frac{{\lambda }_{{\rm{L}}}}{{\lambda }_{E}{\lambda }_{{\rm{A}}}}=\frac{{\lambda }_{{\rm{F}}}}{{\lambda }_{{\rm{L}}}}$ and measured rotations scale according to ${\lambda }_{\theta }=\frac{{\lambda }_{{\rm{F}}}}{{\lambda }_{{\rm{L}}}^{2}}$.
4. Internal forces (axial and shear) are scaled according to *λ*_N,V_ = *λ*_F_ and bending moments are scaled according to *λ*_M_ = *λ*_F_*λ*_L_.
5. Three similitude conditions are especially relevant for the selection of the scale factor for the external forces:
  - Similitude of stress and yield strength: ${\lambda }_{\sigma }=\frac{{\lambda }_{{\rm{F}}}}{{\lambda }_{{\rm{A}}}}={\lambda }_{{f}_{{\rm{y}}}}=1$. This criterion leads to ${\lambda }_{{\rm{F}}}={\lambda }_{{\rm{L}}}^{2}$.
  - Similitude of axial force and member buckling load: ${\lambda }_{{\rm{F}}}=\frac{{\lambda }_{E}{\lambda }_{{\rm{I}}}}{{\lambda }_{{\rm{L}}}^{2}}={\lambda }_{{\rm{L}}}^{2}$.
  - Similitude of stress and local buckling stress. The critical local buckling stress is given by a relation of the type ${\sigma }_{{\rm{cr}}}=k{\rm{\pi }}\frac{E{t}^{2}}{12(1-{\nu }^{2}){b}^{2}}$, in which *k* is a buckling constant, *E* is Young’s modulus, *ν* is Poisson’s ratio, *t* is the section wall thickness and *b* is a representative wall length. Therefore, ${\lambda }_{\sigma }=\frac{{\lambda }_{{\rm{F}}}}{{\lambda }_{{\rm{L}}}^{2}}=\frac{{\lambda }_{{\rm{E}}}{\lambda }_{t}^{2}}{{\lambda }_{(1-{\nu }^{2})}{\lambda }_{b}^{2}}=\frac{{\lambda }_{t}^{2}}{{\lambda }_{b}^{2}}$ and this criterion leads to ${\lambda }_{{\rm{F}}}={\lambda }_{{\rm{L}}}^{2}\frac{{\lambda }_{t}^{2}}{{\lambda }_{b}^{2}}$.
  We have selected a scaling factor ${\lambda }_{{\rm{F}}}={\lambda }_{{\rm{L}}}^{2}$ for the external forces. As well as reproducing the similitude of stresses, yield strength and axial forces, this choice ensures the similitude of member buckling loads; this is crucial, as member buckling determines the load redistribution in the tested specimen near the collapse.
6. The selected scale factor for external forces is not applicable to the mass, because the latter scales with the volume (${\lambda }_{\rho }={\lambda }_{{\rm{L}}}^{3}$). To keep the ratio of vehicle load to self-weight of the scaled-down specimen as in the actual bridge and for the analysis of forces in the bridge, self-weight should be considered as an external load, with a scaling factor ${\lambda }_{{\rm{L}}}^{2}$. This leads to a scaled self-weight load larger than the actual weight of the bridge specimen plus the dead load of the testing rig used for distributing the test loads. To take this into account, we have defined the total value of the test load so that it includes the correction of the self-weight. The experimental setup and the justification of the test load considering the self-weight correction are explained in the following section.

The scaled bridge design is shown in Extended Data Fig. 2 and the complete geometric properties of all cross-sections are provided in Supplementary Information Section 1 (together with comparisons between theoretical and actual scaled-down values). Riveted connections typically used in such steel truss bridges do not behave as the idealized pin joints assumed in design. Instead, they effectively perform as rigid connections and usually have a high capacity for resisting bending moments^28^. As such, all joints of the scaled-down specimen were welded to ensure full-strength connections in a simplified manner.

On the basis of the selected scaling factors, the measured displacements scale in the same way as member lengths, *λ*_d_ = *λ*_L_; rotations and load factors do not require scaling, *λ*_*θ*_ = *λ*_Load factor_ = 1; internal forces scale as the external ones, ${\lambda }_{{\rm{N,V}}}={\lambda }_{{\rm{L}}}^{2}$; and bending moments scale as ${\lambda }_{{\rm{M}}}={\lambda }_{{\rm{L}}}^{3}$. The results presented in this work can thus be easily scaled up by applying these conversions.

### Experiment and monitoring design

After fabrication, the scaled-down bridge specimen had a self-weight of 4.2 kN. It was transported to the ICITECH laboratory and positioned to reproduce the same boundary conditions as those of the real bridge span, with a hinged support on one end and a rolling support on the other (Extended Data Fig. 2). Tests were carried out on the steel material according to EN ISO 6892-1 (ref. ^52^) to characterize its stress–strain behaviour (Supplementary Information Section 1).

The load applied during tests corresponded to the operational loads of the reference bridge (Extended Data Fig. 3). The real bridge is loaded by actual convoys of two vehicles with two-axle bogies and a maximum nominal axle load of 88.4 kN. The loading setup used for testing corresponds to the convoy position that is most unfavourable for global bending of the bridge span. Specifically, this occurs when the heaviest bogies of each vehicle are centred with respect to mid-span (with four axles of 88.4 kN). For testing, these loads were applied using a hydraulic jack and distributed on the main system of the bridge through a testing rig with a weight of 5.4 kN. Loads of the same bogie were grouped together.

The load of the hydraulic jack was introduced pseudo-statically (at a displacement rate of 0.05 mm s^−1^). The magnitude of this load results from multiplying the nominal load of the convoys by the following dynamic effects: (1) an estimated dynamic impact factor of 1.18 representing the dynamic effect of the vehicles passing through the bridge at a certain speed^53^ and (2) a dynamic amplification factor of 1.9 owing to the sudden loss of structural members during the analysed damage scenarios^54,55^. Consequently, the operational load for the bridge specimen was scaled as follows:

$$\text{Scaled operational load}\,=\,88.4\times 4\times 1.18\times 1.9/{3.5}^{2}=64\,{\rm{kN}}$$

To (1) keep the ratio of operational load to self-weight of the scaled-down specimen as in the actual bridge and (2) correctly analyse forces in the bridge, self-weight should also be considered as an external load with a scaling factor ${\lambda }_{{\rm{L}}}^{2}$. Considering that the actual self-weight of the real bridge span is 171 kN and that only the dynamic amplification factor owing to sudden loss of structural members (that is, 1.9) is applicable in this case, the scaled self-weight should be:

$$\text{Scaled self-weight}\,=\,171\times 1.9/{3.5}^{2}=26\,{\rm{kN}}$$

This leads to a scaled self-weight load that is larger than the actual weight of the bridge specimen (4.2 kN) plus the dead load (5.4 kN) of the testing rig used for distributing test loads. The total test load applied by the hydraulic jack was therefore corrected to account for this:

$$\text{Scaled total load for tests}\,=\,64+26-4.2-5.4=80\,{\rm{kN}}$$

Before tests, it was also checked that the load was small enough to ensure that none of the components of the bridge would enter a nonlinear mechanical regime, thus preventing failure and damage of one test from affecting the next.

Up to nine different damage scenarios were considered through component removal (that is, by cutting main components), representing the failure of different member types at critical positions in which they support very high levels of internal load. The considered damage scenarios were: (1) a lower chord in the middle of the span; (2) one of the first diagonals of the span; (3) one of the second verticals of the span; (4) and (5) a horizontal and a vertical bracing located close to the load applied by the jack; and (6) a transversal beam. The fourth and fifth damage scenarios were performed in two testing phases representing the failure of one and two bracing elements of each damage scenario type. The sixth damage scenario was also performed in two phases representing partial and total loss of a transversal beam. For these damage scenarios that involve the failure of transversal beams, the load setup was changed because a transversal beam is most critically loaded when only one of the axles of a bogey is positioned directly on it (Extended Data Fig. 3). For these tests, the scaled operational load only considers the highest traffic load that can be transmitted through a single axle and corresponds to 16 kN. Because the dead load of the testing rig was 2 kN in this case and the self-weight of the entire bridge span is not involved in the structural response of the transversal beam, the total applied load by the hydraulic jack was 14 kN. The defined damage scenarios with the different load setups are shown in Extended Data Figs. 3 and 4. For each damage scenario, the bridge specimen was tested twice, once with the undamaged state and again after component removal. After finishing both tests for each damage scenario, the two parts of the cut component were welded together to retrofit the bridge to its original state before assessing the following damage scenario (Supplementary Information Section 1 and Supplementary Video 2). For the last damage scenario (involving the removal of a diagonal), as well as testing with the specified load of 80 kN, the applied loads were also increased until the system collapsed. This last test was used for the validation of the computational models presented in the final section of Methods.

To monitor the structural behaviour during tests, we heavily instrumented the bridge specimen with several sensors. A total of 80 strain gauges and 14 displacement transducers were placed at key locations in different parts of the bridge. The data from these sensors were complemented by pictures and videos of the structural response captured by three high-resolution cameras (Supplementary Information Section 1 and Extended Data Fig. 5).

### Simulations for studying the onset of ALPs

We performed an extensive set of computational simulations to characterize how the structural response of the bridge changes after the failure of main components. Each failure was represented by removing a single component in the corresponding simulation. At this stage, our computational simulations are aimed at identifying and extracting the resistance mechanisms and the corresponding ALP patterns that the bridge can activate for several damage scenarios.

The kernel of the computational campaign is a three-dimensional finite element model of the undamaged scaled bridge subjected to the same conditions reproduced in tests. With this model, we performed 222 different simulations, each involving the removal of a main component. For each damage scenario simulation, we analysed joint displacements, support reactions and element internal forces at different points on the element cross-sections. The entire process (including modifying the base finite element model and post-processing results) was implemented using a MATLAB^56^ code.

The finite element model of the scaled bridge was implemented in DIANA FEA software^57^. The bridge was modelled using a total of 2,798 two-node, three-dimensional Timoshenko/Mindlin^57^ (shear-deformable) beam elements with six degrees of freedom per node. Geometric and mechanical nonlinearities were considered. The former considers possible member and system instabilities, whereas the latter considers the nonlinear stress–strain characteristics of the material derived from tensile tests (Supplementary Information Section 1). We assumed^58^ a Poisson’s ratio of 0.3 and a density of 7,850 kg m^−3^. Each structural member was discretized into at least five beam finite elements. All joints were modelled as rigid. The boundary conditions and load arrangement reproduce those of the experimental campaign (see Extended Data Figs. 2 and 6).

Before running all simulations, we validated the base finite element model by comparing the results of the laboratory tests with the results of simulations for the undamaged state and the nine damage scenarios tested in the laboratory (Fig. 2f, Supplementary Information Section 2 and Supplementary Video 3). Specifically, vertical displacements of the lower joints and strains at the midpoints of diagonals and chords were used for the validation (Fig. 2f). The differences in structural response variables (for example, internal forces) between damaged and undamaged states define the way in which loads are redistributed. Two complementary approaches were used to characterize the resistance mechanisms for each damage scenario:

1. We can conceptually understand the changes in the response after damage (for example, after the loss of a diagonal component) by analysing the structure, without the damaged component, subjected to joint forces that would counteract the axial force carried by the component before its removal (Supplementary Information Section 2). This provides a sound conceptual picture of the resistance mechanisms activated in each damage scenario and informs the interpretation of the bulk of data provided by the simulation campaign.
2. We also followed a quantitative and systematic approach to reveal and track the changes caused by each damage scenario and to support the definition of ALPs and resistance mechanisms. For this purpose, the following set of key performance indicators were defined:
  - Increment of vertical displacements, Δ*Dz*, at truss joints:
    $$\Delta {Dz}_{i,j}={Dz}_{i,j}^{{\rm{damaged}}}-{Dz}_{j}^{{\rm{undamaged}}}$$
    in which the indices refer to the *i*th damage scenario and *j*th measurement point.
  - Increment of member axial forces, Δ*Fx*:
    $${\Delta Fx}_{i,k}={Fx}_{i,k}^{{\rm{damaged}}}-{Fx}_{k}^{{\rm{undamaged}}}$$
    in which the indices refer to the *i*th damage scenario and *k*th structural member.
  - Maximum increment of member bending moments, Δ*Myz*
    The norm of the bending moment at a given member cross-section *j* of a member *k* is expressed as:
    $${Myz}_{k,j}=\sqrt{{My}_{k,j}^{2}+{Mz}_{k,j}^{2}}$$
    The increments of bending moment at the start section, j_start, and at the end section, j_end, of the structural member *k*, for each damage scenario *i*, are:
    $${\Delta Myz}_{i,k,{\rm{j}}\_{\rm{start}}}={Myz}_{i,k,{\rm{j}}\_{\rm{start}}}^{{\rm{damaged}}}-{Myz}_{k,{\rm{j}}\_{\rm{start}}}^{{\rm{undamaged}}}$$
    $${\Delta Myz}_{i,k,{\rm{j}}\_{\rm{end}}}={Myz}_{i,k,{\rm{j}}\_{\rm{end}}}^{{\rm{damaged}}}-{Myz}_{k,{\rm{j}}\_{\rm{end}}}^{{\rm{undamaged}}}$$
    Finally, the performance indicator is defined as follows:
    $$\Delta {Myz}_{i,k}=\left\{\begin{array}{ll}{\Delta Myz}_{i,k,{\rm{j}}\_{\rm{end}}} & {\rm{if}}\,| {\Delta Myz}_{i,k,{\rm{j}}\_{\rm{end}}}|  > | {\Delta Myz}_{i,k,{\rm{j}}\_{\rm{start}}}| \\ {\Delta Myz}_{i,k,{\rm{j}}\_{\rm{start}}} & {\rm{otherwise}}\end{array}\right.$$
  - Increment of joint support vertical reactions, Δ*Rz*:

$${\Delta Rz}_{i,j}={Rz}_{i,j}^{{\rm{damaged}}}-{Rz}_{j}^{{\rm{undamaged}}}$$

in which the indices refer to the *i*th damage scenario and *j*th support point.

For interpreting the results, we grouped the damage scenarios according to the types of structural member. Each group was also divided into subgroups, which are labelled according to their position:

1. Removal of chord segments: south lower (SL), north lower (NL), south upper (SU) and north upper (NU).
2. Removal of diagonals: south (S) and north (N).
3. Removal of verticals: south (S) and north (N).
4. Removal of horizontal bracings: south to north lower (SNL), north to south lower (NSL), south to north upper (SNU) and north to south upper (NSU).
5. Removal of vertical bracings: north upper to south lower (NU-SL) and north lower to south upper (NL-SU).
6. Removal of stringers: south (S) and north (N).
7. Removal of transversal beams: lower (L) and upper (U).

We assessed the effect of each group of damage scenarios on the different performance indicators of each group of members. The values of each performance indicator were represented in three-dimensional illustrations of the bridge and in heat map charts (Extended Data Fig. 7). In the latter, colour temperature and intensity show the magnitude of the indicator for each group of bridge members (horizontal axis) and for each damage scenario (vertical axis). These heat maps are key to identifying patterns and variations along consecutive structural members and across different damage scenarios.

Combining these conceptual and quantitative approaches, we analysed changes in the deformed shape as well as changes in the magnitude of axial forces, bending moments and reactions, to reveal meaningful ALP patterns for each group of damage scenarios.

### Simulations for studying the evolution of ALPs

We studied the way in which internal forces are redistributed when external loads increase until the collapse of the bridge. This redistribution is a consequence of the propagation of failures within the whole structural system.

In this case, we carried out nonlinear analyses of a set of ten representative damage scenarios up to the collapse of the bridge. The numerical strategy uses arc-length control on the equilibrium path to be able to track the structural behaviour including consecutive local buckling of structural members. Otherwise, the simulations use the same basic modelling assumptions described in the previous section. The ten damage scenarios (Fig. 4) include three different upper and lower chord failures near the centre of the span (six scenarios) and two different failures of diagonals and verticals near the supports (four scenarios).

We validated the collapse simulations by comparing measurements from the last experimental test, in which loads were increased until collapse, with results from a simulation of the same scenario (Extended Data Fig. 8 and Supplementary Information Section 3).

In this part of the research, we analysed the information at two levels:

1. At a global level, we plotted force–displacement curves for the entire structural system for each of the ten representative scenarios. In these curves, displacement corresponds to the vertical displacement measured at mid-span. The force is shown as an overload factor, which represents the multiplier of the reference load of 80 kN used for operational conditions. These curves (Fig. 4) provide information about the evolution of the global stiffness of the structure. They also show the occurrence of local instabilities as snap-through/snap-back events on the equilibrium path, as well as the degradation of global stiffness until collapse.
2. At a local level, we defined two new sets of performance indicators that can be compared to understand the progression of damage and changes to the load-redistribution patterns within the system:
  - The first indicators characterize changes that occur under the effect of the reference operational load of 80 kN. Specifically, they quantify the differences in internal forces (IF), that is, axial forces or bending moments, between the undamaged and initial damaged condition, normalized by the applied load (that is, 80 kN). This allows analysing changes in load-redistribution patterns independently of the total applied load to the bridge. The sign of the indicator is defined as positive if there is an increase in the magnitude of the load (regardless of whether it is tension or compression), whereas if the total magnitude of the indicator decreases, the index will be negative, indicating an unloading or decrease in demand, for both axial forces and bending moments:
    $$\Delta {{\rm{IF}}}_{\text{1st line}}={\rm{abs}}\left(\frac{{{\rm{IF}}}_{{\rm{Damaged}}(80{\rm{kN}})}-{{\rm{IF}}}_{{\rm{Undamaged}}(80{\rm{kN}})}}{80{\rm{kN}}}\right)\times {\rm{sgn}}$$
    $${\rm{with}}\,\left\{\begin{array}{c}{\rm{sgn}}=1\,{\rm{if}}\;{\rm{abs}}({{\rm{IF}}}_{{\rm{Damaged}}}) > {\rm{abs}}({{\rm{IF}}}_{{\rm{Undamaged}}})\\ {\rm{sgn}}=-\,1\,{\rm{if}}\;{\rm{abs}}({{\rm{IF}}}_{{\rm{Damaged}}}) < {\rm{abs}}({{\rm{IF}}}_{{\rm{Undamaged}}})\end{array}\right.$$
  - The second indicator pertains to the collapse condition. It represents the same differences but, in this case, they are normalized by the collapse load obtained from the simulation considering initial damage:

$$\Delta {{\rm{IF}}}_{{\rm{Collapse}}}={\rm{abs}}\left(\frac{{{\rm{IF}}}_{{\rm{Damaged(collapse)}}}-{{\rm{IF}}}_{{\rm{Undamaged(collapse)}}}}{\text{Collapse load}}\right)\times {\rm{sgn}}$$

$${\rm{with}}\,\left\{\begin{array}{c}{\rm{sgn}}=1\,\text{if abs}\,({{\rm{IF}}}_{{\rm{Damaged}}}) > {\rm{abs}}({{\rm{IF}}}_{{\rm{Undamaged}}})\\ {\rm{sgn}}=-\,1\,\text{if abs}({{\rm{IF}}}_{{\rm{Damaged}}}) < {\rm{abs}}({{\rm{IF}}}_{{\rm{Undamaged}}})\end{array}\right.$$

**Extended Data Fig. 9:**
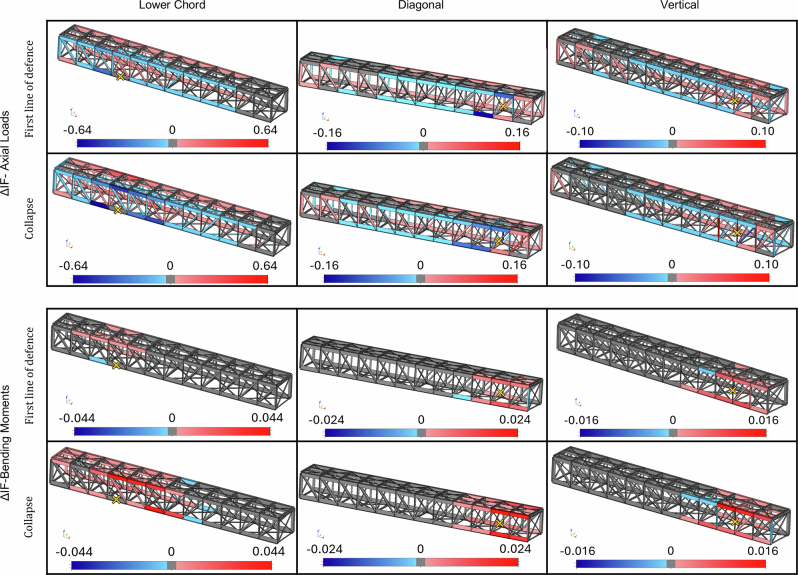
Three-dimensional representations of the difference in internal forces (ΔIF) variables. Representations are shown for axial loads and bending moments in the first line of defence (under operational loads −80 kN) and in the collapse situations for the three types of global failure mode: ductile mode (lower chord), intermediate mode (diagonal) and brittle mode (vertical). Source Data

We extracted conclusions from both approaches to characterize the evolution of ALPs for different damage scenarios (Extended Data Fig. 9) and to identify the mechanisms that are activated by the propagation of failures until collapse.

## Online content

Any methods, additional references, Nature Portfolio reporting summaries, source data, extended data, supplementary information, acknowledgements, peer review information; details of author contributions and competing interests; and statements of data and code availability are available at 10.1038/s41586-025-09300-8.

## Supplementary information

Supplementary Information This file contains a comprehensive report that describes in detail the execution, results and analyses of the experimental tests and computational simulations that were performed as part of this research.

Supplementary Video 1 **Design of the scaled bridge**. This video presents aerial footage of the reference bridge alongside photographs of its scaled model. It follows a structured progression, initially outlining the general characteristics of the bridge before advancing to key considerations for the construction of the scaled model, its intended purpose and the monitoring equipment used.

Supplementary Video 2 **Damage scenarios**. This video presents the nine damage scenarios reproduced during the experimental campaign. A three-dimensional model is used to highlight the removed element in each damage scenario. Each scenario is accompanied by a series of photographs and videos illustrating the cutting and repair process.

Supplementary Video 3 **Data validation**. This video shows a comparison between experimental and computational-simulation data. Vertical displacements at the centre span are shown for the lower chord removal scenario. Test footage is shown, along with synchronized results from both the experimental test and the computational simulations, revealing the good level of agreement between them.

Supplementary Video 4 **Global system failure**. This video shows the final test of the diagonal removal scenario, in which the load is gradually increased until global system failure occurs. A recording of the bridge during the test is presented, along with a synchronized plot of load versus time, highlighting the various buckling events experienced by the structure.

## Source data

Source Data Fig. 2

Source Data Fig. 3

Source Data Fig. 4

Source Data Extended Data Fig. 7

Source Data Extended Data Fig. 9

## Data Availability

Experimental and simulation data used to generate all figures presented in this article have been provided as source data. All experimental data are available at Zenodo: 10.5281/zenodo.15658670 (ref. ^59^). Source data are provided with this paper.
