# Supplementary material for: Latent resistance mechanisms of steel truss bridges after critical failures
Source: Nature. 2025 Sep 3;645(8079):101–7. doi: 10.1038/s41586-025-09300-8 (PMC12408385; doi:10.1038/s41586-025-09300-8)
Supplement: Supplementary file 1 — This file contains a comprehensive report that describes in detail the execution, results and analyses of the experimental tests and computational simulations that were performed as part of this research. [file 41586_2025_9300_MOESM1_ESM.pdf]

nature portfolio

https://doi.org/10.1038/s41586-025-09300-8

Supplementary information

Latent resistance mechanisms of steel truss bridges after critical failures

In the format provided by the authors and unedited

SUPPLEMENTARY INFORMATION

June 2025

Index of contents

Introduction **4**
Section 1. Experimental campaign and relevant results **6**
Section 1.1: Design of the bridge specimen design **7**
Section 1.2: Production of the scaled-down bridge and the test protocol **9**
Section 1.3: Monitoring **11**
Section 1.4: Results and analyses **14**
1.4.1 Criteria adopted for the analysis of the results 14
1.4.1.1 Vertical displacements and related bridge distortions 14
1.4.1.2 Strain measurements 15
1.4.2 DS – Loss of a lower chord 16
1.4.3 DS – Loss of a diagonal 20
1.4.4 DS – Loss of a vertical 25
1.4.5 DS – Loss of horizontal lower bracings 29
1.4.6 DS – Loss of vertical bracings 35
1.4.7 DS – Loss of a transversal beam 40
Section 2. Evaluation of the first defence mechanisms after initial failure **47**
Section 2.1: Description of the computational modelling **47**
Section 2.2: Validation of Simulation Strategy **48**
2.2.1 Displacements 48
2.2.2 Strains 50
2.2.2.1 At Diagonals 50
2.2.2.2 At Central Chords 51
Section 2.3: Simulation of Potential Damage Scenarios **52**
Section 2.4: Results and Analyses **53**
2.4.1 Chord Removal Scenarios 54
2.4.1.1 Displacements 56
2.4.1.2 Bending Moments 57
2.4.1.3 Axial Forces 58
2.4.1.4 Reactions 62
2.4.1.5 Validation of the experimental campaign and conclusions 63
2.4.2 Diagonal Removal Scenarios 64
2.4.2.1 Displacements 65
2.4.2.2 Bending Moments 66
2.4.2.3 Axial Forces 67
2.4.2.4 Reactions 69
2.4.2.5 Validation of the experimental campaign and conclusions 70

2.4.3 Vertical Removal Scenarios ... 71
2.4.3.1 Displacements ... 72
2.4.3.2 Bending Moments ... 73
2.4.3.3 Axial Forces ... 74
2.4.3.4 Reactions ... 76
2.4.3.5 Validation of the experimental campaign and conclusions ... 77
2.4.4 Other Damage Scenarios ... 77
2.4.4.1 Displacements ... 78
2.4.4.2 Bending Moments ... 78
2.4.4.3 Axial Forces ... 79
2.4.4.4 Reactions ... 81
2.4.4.5 Validation of the experimental campaign and conclusions ... 81
Section 3. Evaluation of the additional defence mechanisms up to collapse ... 83
Section 3.1: Description of most important additional assumptions for computational modelling ... 83
Section 3.2: Validation of the simulation strategy ... 85
3.2.1 Displacements ... 86
3.2.2 Strains ... 86
Section 3.3: Load increases for each damage scenario until collapse ... 88
Section 3.4: Internal forces analysis for different load conditions ... 90
3.4.1 Damage Scenario 4 ... 90
3.4.2 Damage Scenario 5 ... 92
3.4.3 Damage Scenario 6 ... 93
3.4.4 Damage Scenario 28 ... 95
3.4.5 Damage Scenario 29 ... 96
3.4.6 Damage Scenario 30 ... 98
3.4.7 Damage Scenario 59 ... 99
3.4.8 Damage Scenario 60 ... 101
3.4.9 Damage Scenario 83 ... 103
3.4.10 Damage Scenario 84 ... 104
Section 3.5: Discussion of results ... 105
3.5.1 Main Effects ... 105
3.5.2 Defence mechanisms at collapse load ... 107
3.5.3 Summary of Conclusions ... 113

Introduction

This report was prepared as supplementary information for the main article. The report aims to elaborate in detail on the results and analyses of the experimental campaign and computational simulations.

The report is structured into three main sections:

- **S1**. Experimental campaign and relevant results.
- **S2**. Evaluation of the first defence mechanisms after initial failure.
- **S3**. Evaluation of the additional defence mechanisms up to global failure.

Nomenclature

The definition of symbols used in this report are summarised in the following table.

Symbol	Definition
f_y	Yield strength of steel
f_u	Ultimate strength of steel
λ_L	Scaling factor for member lengths.
λ_E	Scaling factor for Young's modulus
λ_ρ	Scaling factor for material density
λ_{f_y}	Scaling factor for yield strength
λ_υ	Scaling factor for Poisson's ratio
λ_A	Scaling factor for cross sectional areas
λ_I	Scaling factor for second moment of area
λ_d	Scaling factor for displacements
λ_θ	Scaling factor for rotations
λ_N	Scaling factor for axial forces
λ_N	Scaling factor for shear forces
λ_M	Scaling factor for bending moments
E_s	Young's modulus of steel
F_u	Ultimate tensile strength of steel
ε_y	Strain at the yield stress
ε_u	Strain at ultimate tensile strength
Δ_d	Displacement increment between damaged and undamaged states
d_D	Vertical displacement for the damaged state
d_U	Vertical displacement for the undamaged state
θ_D	Transverse distortion for the damaged state
θ_U	Transverse distortion for the undamaged state
Δ_θ	Transverse distortion increment between damaged and undamaged states
b_{bridge}	Width of scaled bridge
$\Delta\varepsilon$	Strain increment between damaged and undamaged states
ε_D	Strain for the damaged state
ε_U	Strain for the undamaged state
$\Delta Dz_{i,j}$	Displacement indicator between the damaged and undamaged states, for the i-th damage scenario and the j-th measured point
$Dz_{i,j}^{damaged}$	Vertical displacement for the i-th damage scenario and the j-th measured point
$Dz_j^{undamaged}$	Vertical displacement of the j-th measured point for the undamaged state

$\Delta Rz_{i,j}$	Reaction forces indicator between the damaged and undamaged states, for the i-th damage scenario and the j-th support point
$Rz_{i,j}^{damaged}$	Vertical reaction for the i-th damage scenario and the j-th support point
$Rz_{j}^{undamaged}$	Vertical reaction of the j-th support point for the undamaged state
$\Delta Fx_{i,k}$	Axial force indicator between the damaged and undamaged states, for the i-th damage scenario and the k-th structural member
$Fx_{i,k}^{damaged}$	Longitudinal axial forces for the i-th damage scenario and the k-th structural member
$Fx_{k}^{undamaged}$	Longitudinal axial forces of the k-th structural member for the undamaged state
$My_{k,j}$	Bending moment around y-axis at the j-th node of structural member k
$Mz_{k,j}$	Bending moment around z-axis at the j-th node of structural member k
$Myz_{k,j}$	Resultant bending moment at the j-th node of structural member k
$\Delta Myz_{i,k}$	Maximum bending moment indicator between the damaged and undamaged states, for the i-th damage scenario and the k-th structural member
$\Delta Myz_{i,k,j\_end}$	Bending moment indicator between damaged and undamaged states, for the i-th damage scenario and the j-th end node of the k-th structural member
$\Delta Myz_{i,k,j\_start}$	Bending moment indicator between damaged and undamaged states, for the i-th damage scenario and the j-th start node of the k-th structural member
$Myz_{i,k,j\_end}^{damaged}$	Resultant bending moment at the j-th end node of structural member k for the i-th damage scenario
$Myz_{k,j\_end}^{undamaged}$	Resultant bending moment at the j-th end node of structural member k for the undamaged state
$Myz_{i,k,j\_start}^{damaged}$	Resultant bending moment at the j-th start node of structural member k for the i-th damage scenario
$Myz_{k,j\_start}^{undamaged}$	Resultant bending moment at the j-th start node of structural member k for the undamaged state
$\Delta IF_{1^{st}\ line}$	Normalized indicator of the internal forces difference between damaged and undamaged states for a load of 80 kN
$IF_{Damaged\ (80kN)}$	Internal force of a structural member for the damaged state under a load of 80 kN
$IF_{Undamaged\ (80kN)}$	Internal force of a structural member for the undamaged state under a load of 80 kN
$\Delta IF_{Collapse}$	Normalized indicator of internal force differences between damaged and undamaged states at collapse load
$IF_{Damaged\ (Collapse)}$	Internal force of a structural member for the damaged state under the collapse load
$IF_{Undamaged\ (Collapse)}$	Internal force of a structural member for the damaged state under the collapse load

Section 1. Experimental campaign and relevant results

Section 1.1: Design of the bridge specimen design

The bridge used as a reference for this study was built between 1913 and 1915 and is representative of the type of bridges built at the beginning of the 20th century. Due to concerns on the remaining fatigue life of several elements, an exhaustive control of the bridge was carried out through continuous monitoring and regular inspections during the last 4 years of operation. Finally, at the end of 2024 (after 109 years under operation), it ceased its activity, and today, it is part of the historical and cultural heritage of the region.

The bridge is a steel truss-type bridge with riveted connections. It has six spans: four identical isostatic spans with 21m length at the ends and two identical continuous spans with 42m length each in the central part. One of the isostatic spans was considered for this study. Fig. 1 shows pictures, and a plan, lateral and cross-section views of the whole bridge.

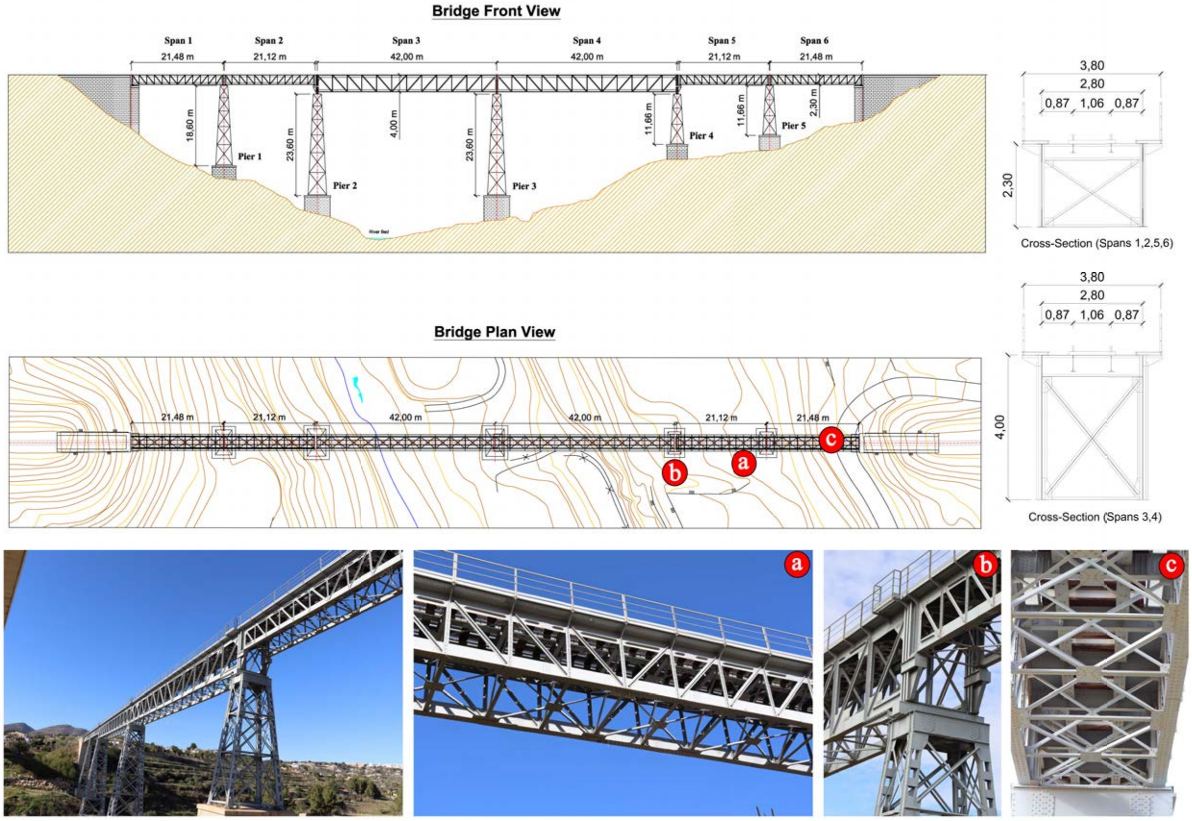

Fig. 1 | The bridge.

For the study presented in this article, a scaled version of the bridge described above was used. The scale factor used was λ_L =3.5, so the scaled isostatic span length is 6m. This 6m bridge was the one that was subsequently fabricated and used for both testing and computational modelling. As evidenced by different works[1–3], the high costs of testing means that such scaled-down specimens are commonly used in structural engineering and structural robustness research. It is worth mentioning that scaled-down tests are especially suitable for structures made of ductile materials such as steel (as opposed to brittle ones such as concrete). This is due to the fact that these materials experience significant plastic deformation and fail through yielding rather than brittle fracture. Yield criteria scale much more uniformly (based on a simple power law) compared to fracture mechanics principles[4].

The scaled bridge was designed using a material with mechanical properties similar to the original and determined by tensile tests[5]. Table 1 shows the results obtained for three specimens taken directly from the real bridge. It is also known from the existing documentation of the original bridge and similar bridges on the same railway line that Young's modulus of the steel of the real bridge is 210 GPa. As can

be seen, the steel of the reference bridge can be assimilated to S275 steel. In addition, riveted joints typically used in such steel truss bridges usually have a high capacity for resisting bending moments[6]. As such, all joints of the scaled-down specimen were welded to ensure full-strength connections in a simplified manner.

Table 1 | Tensile mechanical properties of the steel of the bridge.

Specimen	Yield Strength f_y [MPa]	Ultimate Strength f_u [MPa]	Ultimate strain [%]
#1	290.3	423.3	31.3
#2	270.0	355.0	31.0
#3	276.2	422.9	31.8
Mean	278.8	400.4	31.4

Considering the similarity in the mechanical properties of the materials (i.e. $\lambda_E = \lambda_\rho = \lambda_{f_y} = \lambda_\upsilon = 1$, representing Young's modulus, density, yield strength and Poisson's ratio, respectively) and that the external loads (e.g. traffic loads) are scaled as $\lambda_F = {\lambda_L}^2$, the scaled bridge design was carried out by applying the following scaling rules[7,8] (more in-depth explained in Methods Section 1)

- Dimensions (member length and bridge length): $\lambda_L = 3.50$
- Areas: $\lambda_A = {\lambda_L}^2 = 12.25$
- Moments of inertia: $\lambda_I = {\lambda_L}^4 = 150.06$

Applying these scaling rules to the different elements of the real bridge, Fig. 2 shows the final design of the scaled bridge. In this final design, commercial steel profiles currently in stock were chosen, adapting the selection of the profiles to those with a minimum deviation regarding the scaled-down sectional characteristics (areas and inertias) of the real elements of the reference bridge. Table 2 shows the areas and inertias of the different profiles of the reference bridge and the scaled-down specimen.

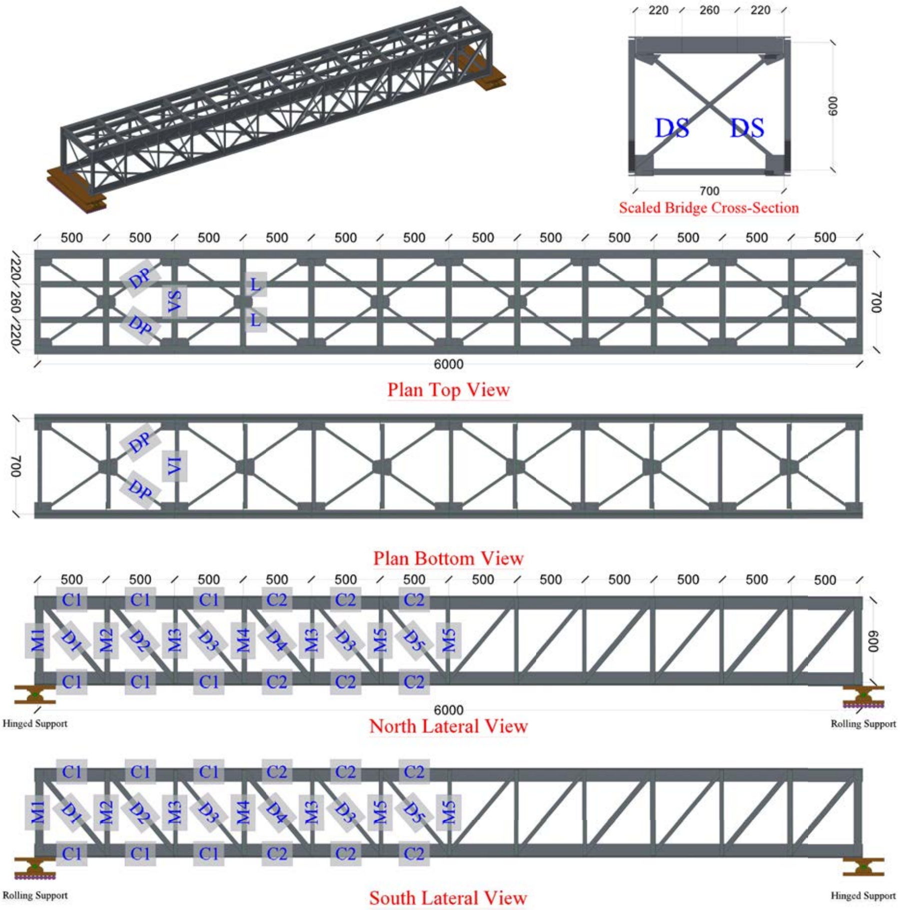

Fig. 2 | Final design of the bridge specimen.

Table 2 | Areas and inertias of the original profiles of the reference bridge and the scaled-down specimen.

		Reference bridge			Scaled-down properties			Scaled-down specimen			
Elements		A [cm^2]	Ix [cm^4]	Iy [cm^4]	A [cm^2]	Ix [cm^4]	Iy [cm^4]	Commercial profile	A [cm^2]	Ix [cm^4]	Iy [cm^4]
Main system	C1	82.7	21160.0	1661.6	6.8	141.0	11.1	T from IPE120 (100mm high)	8.4	83.0	8.0
	C2	104.3	25250.0	2973.8	8.5	168.3	19.8	T from IPE120 (100mm high)	8.4	83.0	13.9
	M1	102.8	2592.0	5644.6	8.4	17.3	37.6	L60.6	6.9	22.8	22.8
	M2	44.0	568.4	387.7	3.6	3.8	2.6	L40.4	3.1	4.5	4.5
	M3	40.0	509.2	347.2	3.3	3.4	2.3	L30.3	1.7	1.4	1.4
	M4	48.0	628.1	428.5	3.9	4.2	2.9	L40.4	3.1	4.5	4.5
	M5	31.6	392.4	267.5	2.6	2.6	1.8	L30.3	1.7	1.4	1.4
	D1	60.0	849.0	849.0	4.9	5.7	5.7	L40.4	3.1	4.5	4.5
	D2	52.0	588.3	588.3	4.2	3.9	3.9	L40.4	3.1	4.5	4.5
	D3	42.2	467.0	467.0	3.4	3.1	3.1	L30.3	1.7	1.4	1.4
	D4	56.8	649.8	649.8	4.6	4.3	4.3	L40.4	3.1	4.5	4.5
	D5	37.2	407.1	407.1	3.0	2.7	2.7	L30.3	1.7	1.4	1.4
Floor system	VS	88.6	28150.0	787.4	7.2	187.6	5.2	IPE80	7.6	80.1	8.5
	L	53.3	5139.0	473.5	4.4	34.2	3.2	T from IPE80 (65mm high)	4.9	19.6	4.2
Bracing system	DP	11.0	35.5	35.5	0.9	0.2	0.2	L20.3	1.1	0.4	0.4
	DS	11.0	35.5	35.5	0.9	0.2	0.2	L20.3	1.1	0.4	0.4
	VI	22.0	70.9	193.8	1.8	0.5	1.3	L30.3	1.7	1.4	1.4

The applied scaling ensures that the tests accurately represent the most common types of failure, namely yielding and member buckling. Although the full-scale bridge may also be susceptible to additional forms of local plate buckling within individual members, these have a negligible influence on the development of secondary resistance mechanisms at the scale of the bridge span.

The conversion of the outputs (e.g. stresses) obtained from the scaled-down version to the full-scale bridge, both from tests (this section) and simulations (Sections 2 and 3), can be achieved using the following expressions:

- Displacements: $\lambda_d = \lambda_L = 3.50$
- Rotations and load factors used in Section 3 do not require scaling: $\lambda_\theta = \lambda_{Load\ factor} = 1.00$
- Axial (N) and shear (V) forces: $\lambda_N = \lambda_V = {\lambda_L}^2 = 12.25$
- Bending moments (M): $\lambda_M = {\lambda_L}^3 = 42.88$

This final conversion, which only consists of applying the aforementioned conversion factors to the outputs, has not been carried out in this work. Obviously, the conclusions obtained in this work are equally valid and can be extrapolated to the full-scale bridge by applying these conversion values. Note that although the magnitude of forces will be different when scaled up, trends related to the onset and evolution of alternative load paths will not be affected.

Section 1.2: Production of the scaled-down bridge and the test protocol

Fig. 3 shows the designed scaled-down bridge after production and how the actual bridge boundary conditions are reproduced in the lab. As is the case in the real bridge, supports reproduced a hinged support on one side and a rolling support on the other. The total self-weight of the specimen was 4.2 kN.

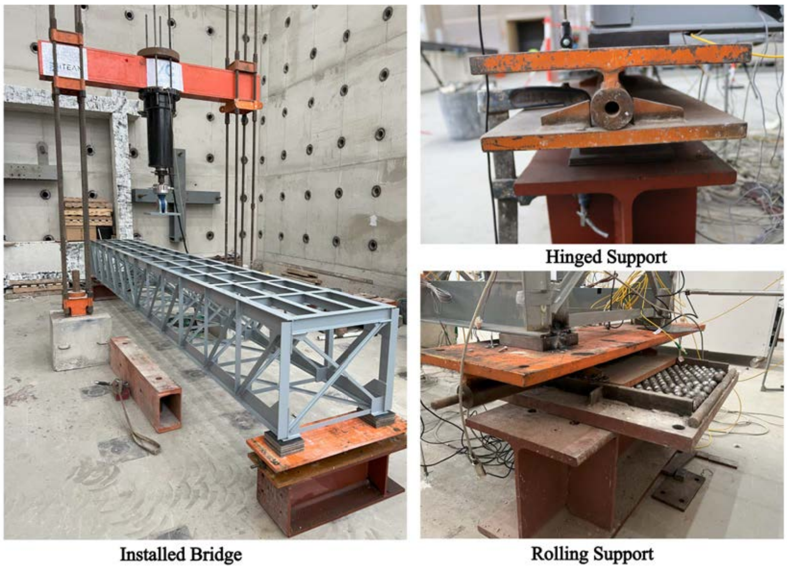

Fig. 3 | Bridge specimen after production and reproduction of the actual boundary conditions.

Tensile strength tests were carried out on specimens of the same material used to manufacture the scaled bridge to obtain the material's mechanical properties of the different elements. Tensile tests were performed according to EN-ISO-6892 standard. A controlled displacement test was carried out on dog-bone specimens with a rectangular cross-section (see Fig. 4). Tests were carried out on 6 specimens with the aim of determining a series of fundamental parameters to characterise the constitutive stress-strain curve of the steel. The output parameters were the Young's modulus (E_s), the yield stress (F_y), ultimate tensile strength (F_u), strain at the yield stress (ε_y) and the strain at ultimate tensile strength (ε_u). Fig. 4 shows the average stress-strain curve which has been used later in the computational models.

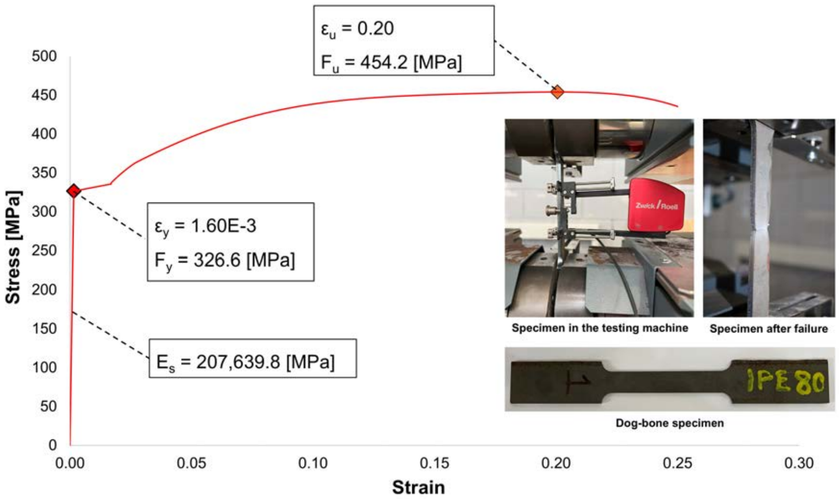

Fig. 4 | Average stress-strain curve and testing setup of the steel characterisation.

The level of load applied to the scaled bridge during tests was adopted in agreement with the real level of load of the reference bridge. The real-scale bridge is loaded by actual convoys of two vehicles with 2-axle bogies and a maximum nominal axle load of 88.4 kN (see Fig. 5). The tests are carried out assuming that the position of a convoy is such that the heaviest bogies of each vehicle are centred, at mid-span (Fig. 5 and Extended Data 3). Thus, the final load setup for the test, considering that loads of the same bogie were grouped, is shown in Fig. 6 (load setup 1), where the applied load of the hydraulic jack is distributed on the bridge through a testing rig (testing rig weight equal to 5.4 kN).

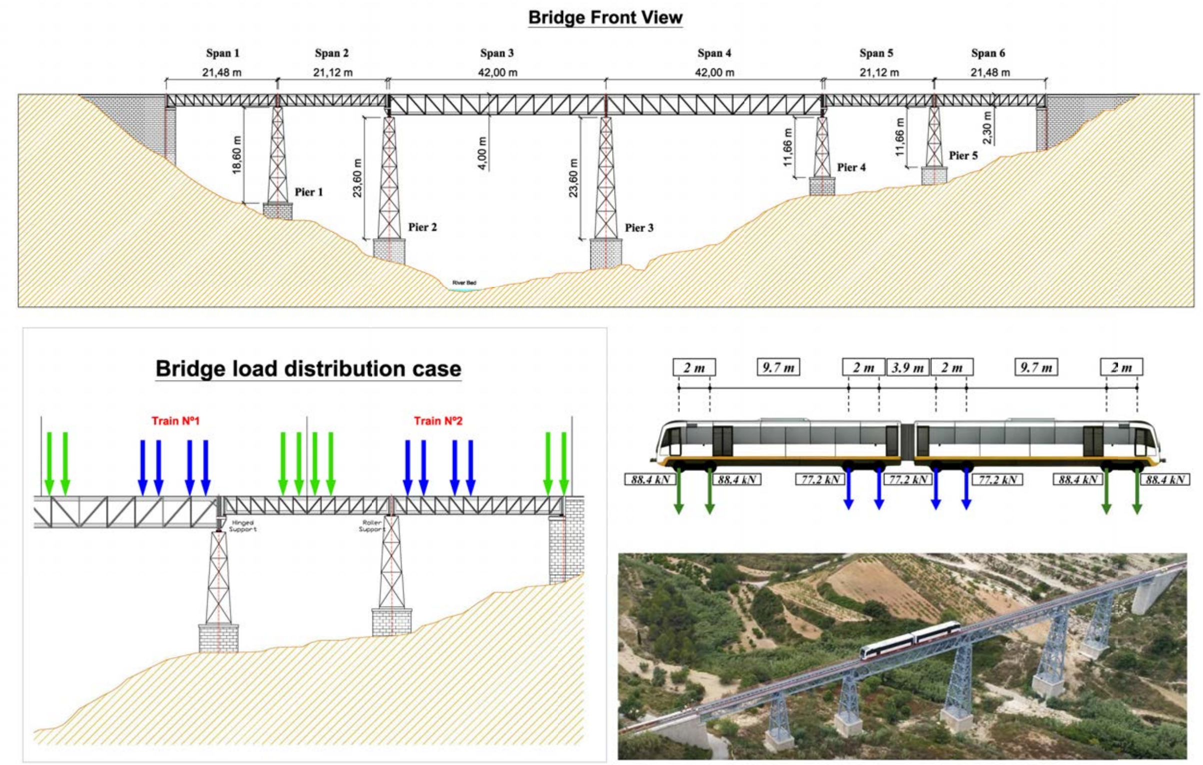

Fig. 5 | Load and load distribution of the convoys.

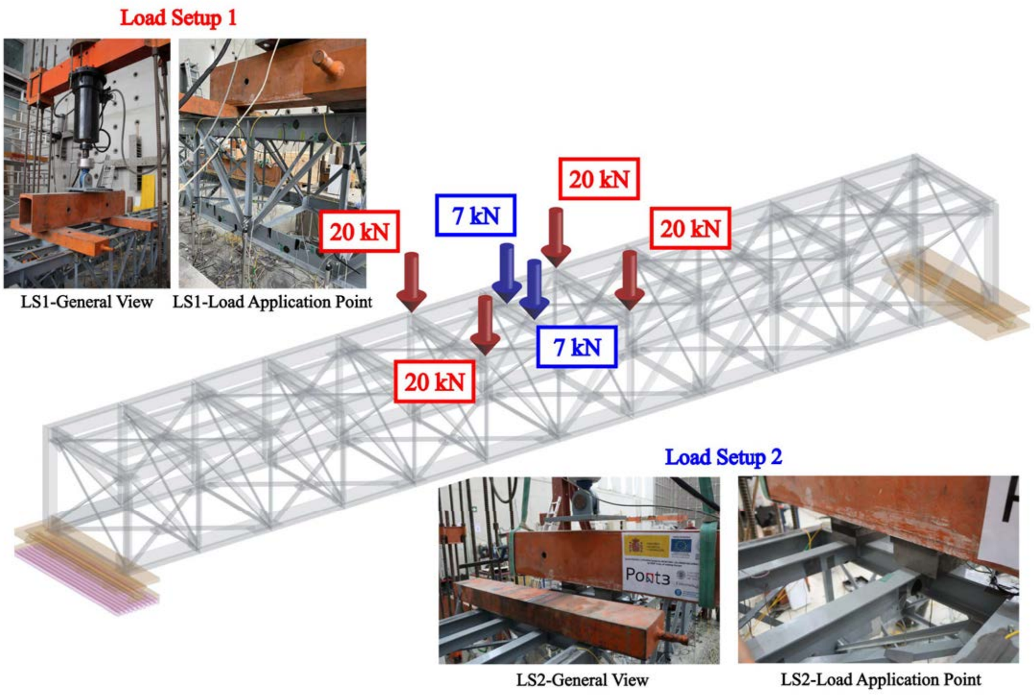

Fig. 6 | Load setup of the test.

For the study, as tests were performed applying the load slowly (quasi-static tests), the assumed magnitude of the actual load applied during tests is widely justified in Methods Section 2. Out of the elements involved in the damage scenarios (Fig. 7), only one is part of the flooring system (Fig. 7, DS-8,9: Loss of transversal beam), and two are part of the bracing system (Fig. 7, DS-4,5,6,7: Loss of horizontal and vertical bracings) while the remaining three belong to the Pratt trusses and, therefore, to the main system (Fig. 7, DS-1,2,3: Loss of a chord, a diagonal and a vertical, respectively). The load configuration for the scenarios involving elements of the main and bracing systems (Load Setup-1) was characterised by applying four point 20 kN loads, symmetrically distributed on the upper nodes on both sides of the central axis of the bridge. On the other hand, the evaluation of the damage scenario involving

the flooring system (DS: Loss of transversal beam) required the adoption of a different load configuration (Load Setup-2), where the applied load simulates the passage of one of the axles of the train over the longitudinal members (i.e. stringers). For this test, the scaled traffic load is 16 kN (only considering a traffic axle load), and the dead load of the testing rig, in this case, was 2 kN. Thus, the total load applied by the jack to this test was 14 kN.

Each of the DSs was applied independently. This means that the undamaged structure was first structurally evaluated before imposing damage. A specific DS was reproduced and the structural response of the damaged bridge was assessed. The bridge structure was then repaired and assessed again (after repair) before testing the subsequent DS. Fig. 8 shows the adopted workflow.

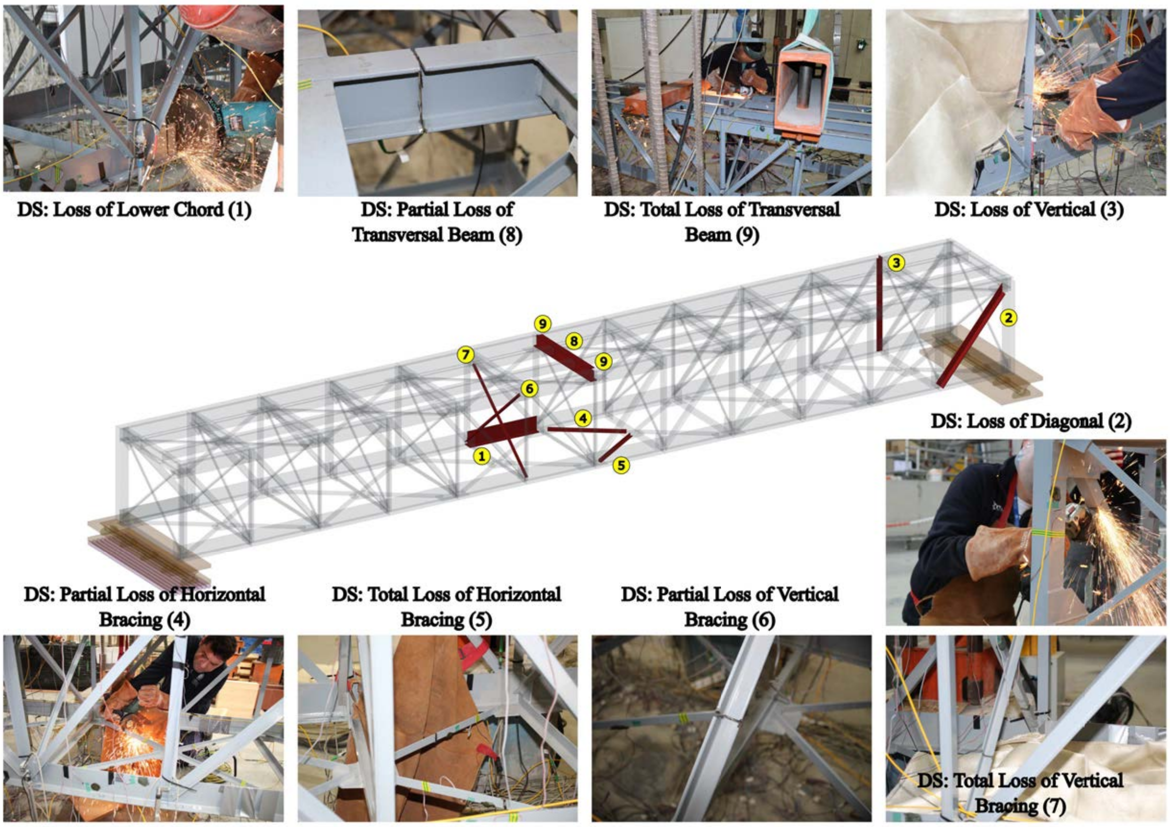

Fig. 7 | Damage Scenarios.

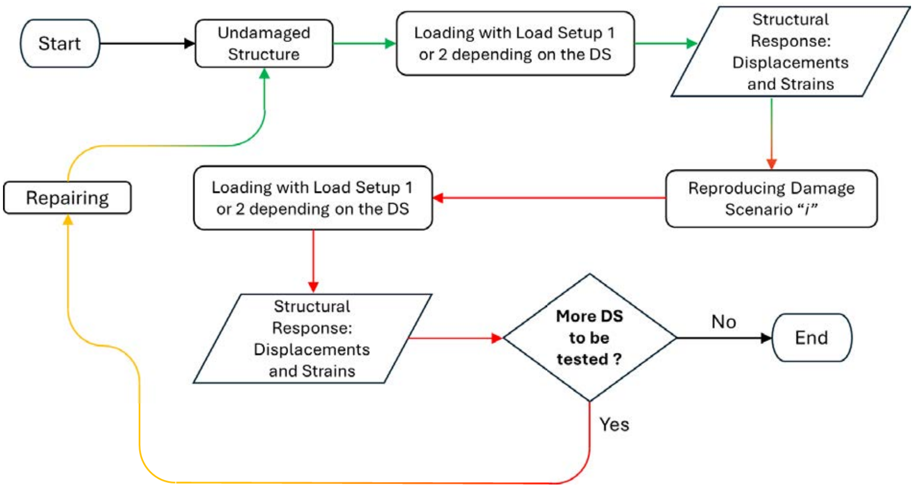

Fig. 8 | Workflow.

Section 1.3: Monitoring

This section describes the monitoring plan used on the scaled bridge. As described above, the scaled bridge was composed of an interconnected system of stringers and transversal beams (Floor System)

that distributes the loads to 2 Pratt trusses (Main System), which in turn are connected by vertical and horizontal bracings (Bracing System).

In the undamaged condition, these bridges are characterised by their triangulated geometric configuration, where axial forces predominate with a very low magnitude of bending moments at the connections. In this way, Pratt trusses work globally as girders: gravity loads cause compressive axial forces acting on the upper chords and tensile axial forces on the lower chords, which are equivalent to global bending moments. Complementarily, global shear forces result from compressive forces on the vertical members and tensile forces on the diagonals. The vertical and horizontal bracing systems, on the other hand, contribute to the lateral and torsional stiffness of the bridge, also through mainly axial loads in the undamaged configurations. Regarding the flooring system, transversal beams and stringers mainly work under flexure. However, for each damage scenario, the loss of a structural member causes a disruption in the triangular pattern of the system. This triggers a redistribution of internal forces and favours the increase of bending moments within the remaining truss members.

The monitoring involved the use of 80 strain gauges and 14 displacement transducers. To measure the vertical displacements of the bridge, displacement sensors were used at the lower nodes of the bridge, specifically at the point of connection between the lower chord, the vertical and the diagonal. Seven sensors were installed on each side of the bridge (South and North), as shown in Fig. 9.

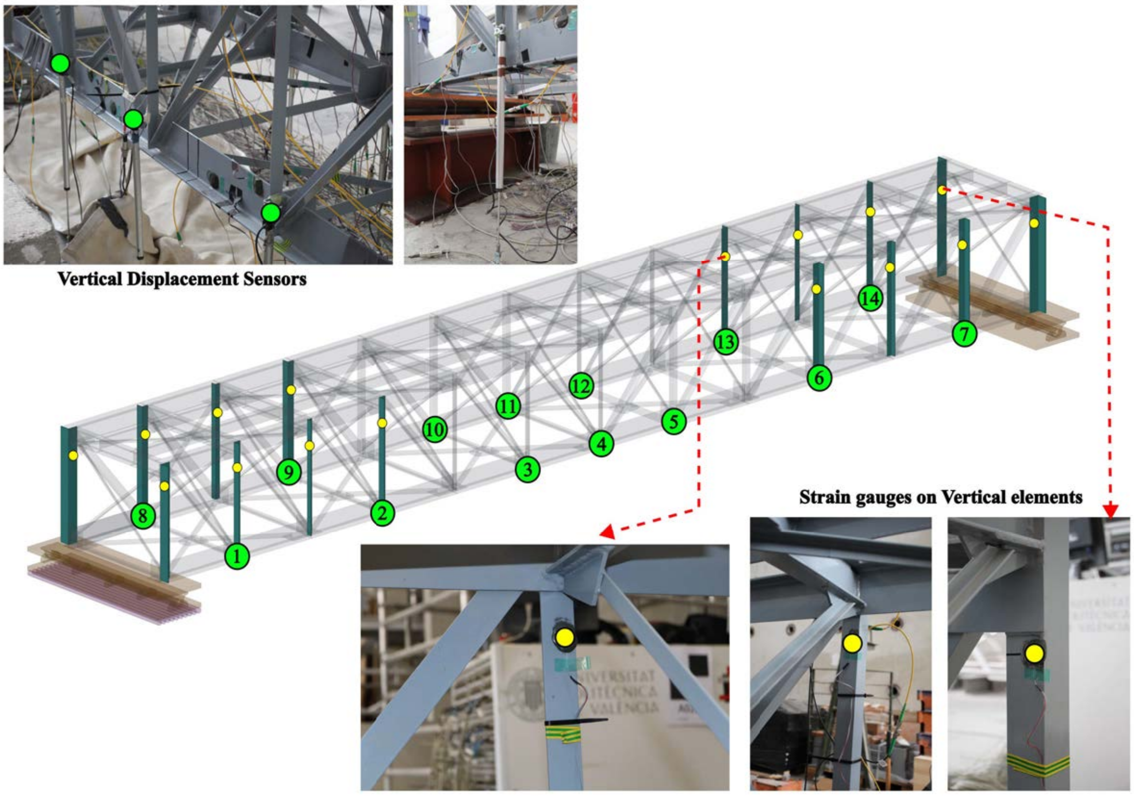

Fig. 9 | Vertical displacement sensors and strain gauges on vertical elements.

The strain gauges were installed on various elements, including diagonals, verticals, chords, bracing, and stringers. Each sensor's location was strategically chosen to relate it to the internal forces resulting from the bridge's structural response. As detailed below, in some situations, the placement of the strain gauges allowed for the isolation of axial loads from bending moments. In other cases, they captured the simultaneous effect of both internal responses (i.e., the interaction between axial forces and bending moments).

As shown in Fig. 10, the strain gauges placed specifically to evaluate axial loads were installed in the 12 external diagonals, in the 8 central chords, in 8 central horizontal bracings and in 4 vertical bracings. For all cases, the sensors were located at the centre of the element length and in the centroid of the cross-section, limiting the possible influence of bending moments on the sensors' measurement.

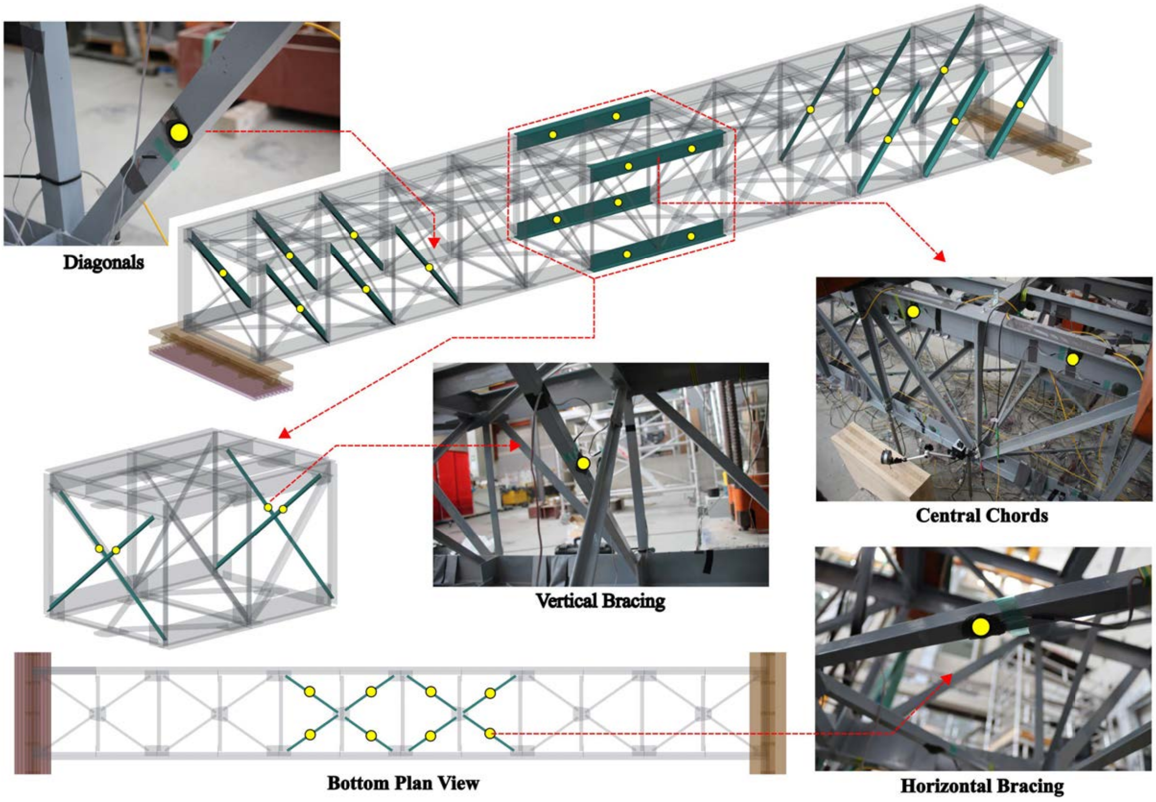

Fig. 10 | Strain gauges located in representative positions to only measure axial forces.

In addition, as shown in Fig. 9 and Fig. 11, sensors were installed on verticals, chords, and stringers to evaluate the axial force-bending moment interaction after the damage scenarios. Concerning the verticals, all 16 external elements of the bridge were instrumented, and sensors were installed at the element's higher part (i.e. 5 cm below the web of the upper chord). Regarding the chords, 28 gauges were installed at the ends of the central chords and in those that required special attention due to the associated damage scenario. For chords, the arrangement of the sensors was always done in pairs, installing one in the upper fibre of the flange and the other in the lower fibre of the web (located at 5 cm from the intersection between the flange of the transversal beam and the flange of the chord). As for the stringers, the two stringers located to the west of the removed transversal beam in DS-8,9 were instrumented (see Fig. 7). On each beam, 2 gauges were placed on the lower fibre of the web, 5 cm from its connections with the transversal beam.

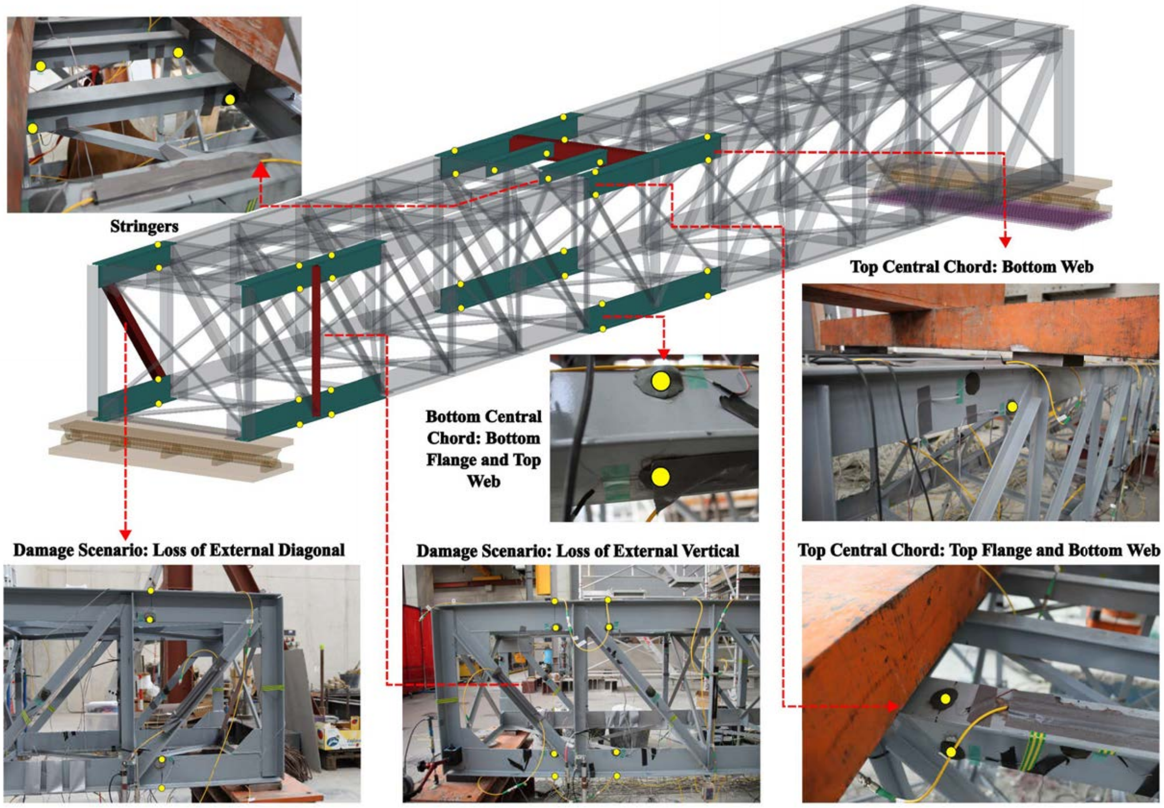

Fig. 11 | Strain gauges located in representative positions to measure axial forces and bending moments.

Section 1.4: Results and analyses

This section presents the results and analysis of the different damage scenarios in order to discuss the different Alternative Load Paths (ALPs) that the structure is capable of activating after each of the initial failures. The results obtained from the experimental campaign, as described above, are based on data recorded by the sensors in terms of vertical displacements and strains. Therefore, this section first describes the criteria used to analyse the results. Subsequently, each damage scenario (DS) is analysed individually, and the activated ALPs are determined. A summary of the conclusions is given at the end of each subsection.

1.4.1 Criteria adopted for the analysis of the results

This section describes the criteria adopted for the analysis of the results. This is only a description of the criteria, but it does not intend to show where the results of sensors were acquired. In subsequent sections, for each case, the specific locations of the different sensors will be defined together with the extracted results.

1.4.1.1 Vertical displacements and related bridge distortions

Vertical displacements are considered positive when the measured value indicates a downward movement of the bridge. The displacements measured by each sensor for the damaged (D) and undamaged (U) conditions are processed to obtain the increments indicated by Eq. 1 in absolute values and Eq. 2 relative to the undamaged condition (expressed as a percentage).

$$\Delta_d = d_D - d_U \qquad \text{Eq. (1.)}$$

$$\frac{\Delta_d}{|d_U|} = \frac{d_D - d_U}{|d_U|} \qquad \text{Eq. (2.)}$$

Where d_U denotes the displacement for the undamaged bridge, d_D the displacement recorded for the damaged condition, Δ_d the increment of the aforementioned variables and $\frac{\Delta_d}{|d_U|}$ the relative increment, expressed as a percentage.

From the vertical displacements measured in the different parts of the structure (i.e. north and south according to Fig. 12), the bridge transverse distortions were obtained as expressed in Eqs. 3-5. The distortions in the transverse direction for any *i* section of the bridge are represented by θ_i and obtained from the difference of opposite vertical displacements (South: S; and North: N) as shown in Eqs. 3-4 (see also Fig. 12). Therefore, for the undamaged bridge condition, the vertical displacement in the southern part of any *i* section is denoted by $d_{i,US}$, while the same, in the northern part, correspond to $d_{i,UN}$. The same applies to the damaged condition, expressing the variables as $d_{i,DS}$ and $d_{i,DN}$ for the southern and northern parts, respectively. It is important to note that the distance between measurement points corresponds to the bridge's width, denoted by b_{bridge}. The relative increments between the distortions in damaged and undamaged situations are calculated according to Eq. 5.

$$\theta_{i,U} = \tan^{-1}\left(\frac{d_{i,US} - d_{i,UN}}{b_{bridge}}\right) \qquad \text{Eq. (3.)}$$

$$\theta_{i,D} = \tan^{-1}\left(\frac{d_{i,DS} - d_{i,DN}}{b_{bridge}}\right) \qquad \text{Eq. (4.)}$$

$$\Delta_{\theta,i} = \frac{\theta_{i,D} - \theta_{i,U}}{|\theta_{i,U}|} \qquad \text{Eq. (5.)}$$

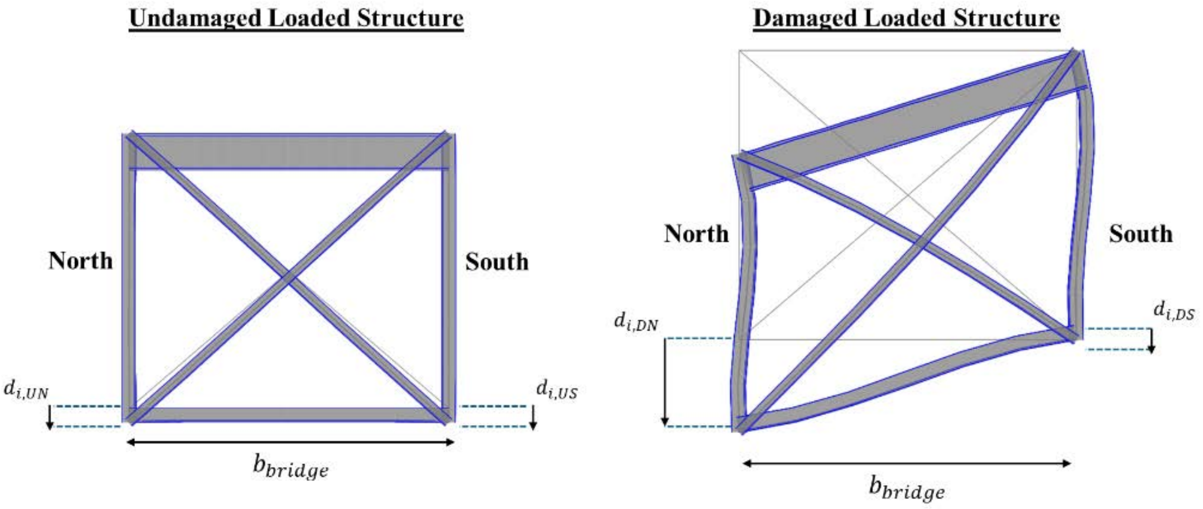

Fig. 12 | Variables for the definition of the distortion

1.4.1.2 Strain measurements

For strain measurements, tensions are considered positive, and compressions negative. The values measured by the sensors have also been processed into relative strain increments between the damaged and undamaged situations ($\varepsilon_D - \varepsilon_U$), relative to the undamaged condition (ε_U), as defined in Eq. 6. Furthermore, these same increments ($\varepsilon_D - \varepsilon_U$) were computed relative to the yielding strain[1] (Eq. 7) as a measure of the significance of this increase in comparison to a reference value related to the steel strength.

$$\frac{\Delta\varepsilon}{|\varepsilon_U|} = \frac{\varepsilon_D - \varepsilon_U}{|\varepsilon_U|} \qquad \text{(Eq. 6.)}$$

$$\frac{\Delta\varepsilon}{\varepsilon_y} = \frac{\varepsilon_D - \varepsilon_U}{\varepsilon_y} \qquad \text{(Eq. 7.)}$$

Additionally, the damaged state of the bridge, with respect to its undamaged state, has been represented by a set of symbols that indicate whether the value may be related to an increase, decrease, or reversal of the load conditions, as shown in Table 3.

---

[1] The yielding strain is defined as the ratio between the yield stress and the Young's modulus, in this case, 326 MPa and 207,000 MPa respectively → εy=0.00157

Table 3 | Convention for the state of loading.

Symbol	Meaning
↑	Loading
↓	Unloading
↺	Strain reversal

Finally, a colour scale has been adopted for the values obtained in Eq. 7 to visualise the processed results better and to identify the most significant differences between the situations with and without damage. This colour legend and its meaning are defined in Fig. 13. Positive values are associated with tensioning or decompression, while negative values are associated with compression or un-tensioning. The grey colour is reserved for small values.

Δε/εy Range of Values and colour convention	
Value	State
≥ 100 %	Tensioning or decompression
95.50%	
86.50%	
77.50%	
68.50%	
59.50%	
50.50%	
41.50%	
32.50%	
23.50%	
14.50%	
5.00%	
-5.00%	Compression or untensioning
-14.50%	
-23.50%	
-32.50%	
-41.50%	
-50.50%	
-59.50%	
-68.50%	
-77.50%	
-86.50%	
-95.50%	
≤ -100 %	

Fig. 13 | $\Delta\varepsilon/\varepsilon_y$ range of values and colour convention.

1.4.2 DS – Loss of a lower chord

Fig. 14 shows the definition of this damage scenario.

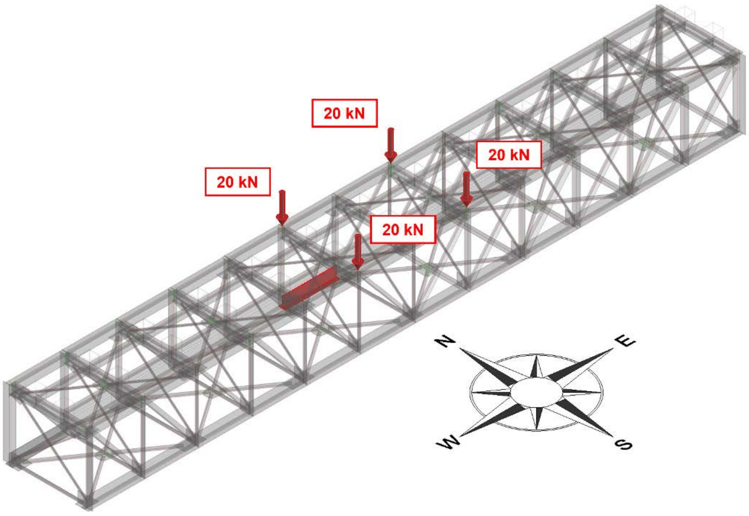

Fig. 14 | Bridge, loads and damage scenario identified by the red colour element.

The results obtained for vertical displacements and distortions are shown in Fig. 15 and Fig. 16. Each figure includes the references and units for the sensor measurements: d for displacements (in mm) and θ for distortions (in degrees). Additionally, the absolute values recorded by each sensor in undamaged

(UD) and damaged (D) conditions are presented, along with the processed results, as discussed at the beginning of Section 1.4. From the results, it can be concluded that:

- It has been determined that the loss of the lower central chord exerts a substantial influence on the vertical stiffness of the bridge, with the affected part demonstrating vertical displacements that are up to 56% greater than those observed in the undamaged condition.
- The displacement values measured on the west side exceed those recorded on the east side, attributable to the off-centre location of the damage towards the west side.
- The disparity in displacements measured on the North and South sides is an initial indicator of ALP activation. This phenomenon occurs because a part of the load exerted on the damaged (South) side is redistributed to the undamaged (North) through the ALP.
- The difference in vertical displacements between the north and south sides of the bridge results in a distortion of the bridge cross-section, which is mainly concentrated in the damage zone and becomes less prominent towards the supports.

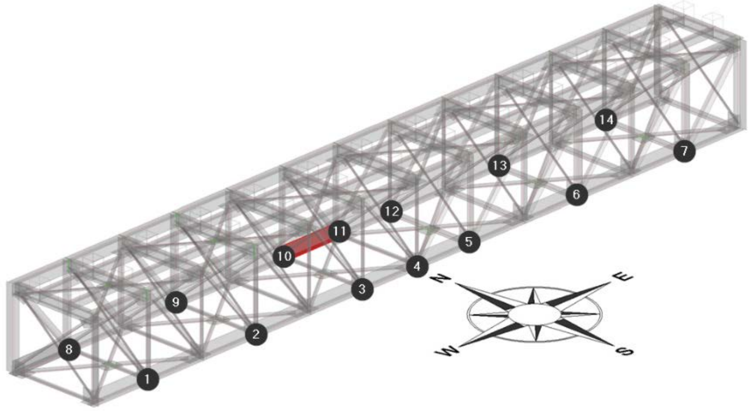

#	Unit	Ref	UD	D	$\Delta d / \|d_U\|$ [%]
1	[mm]	d	2.49	3.29	32.16%
2	[mm]	d	5.73	7.91	37.98%
3	[mm]	d	8.19	11.29	37.85%
4	[mm]	d	8.36	11.34	35.68%
5	[mm]	d	7.95	10.46	31.65%
6	[mm]	d	5.49	6.99	27.51%
7	[mm]	d	2.23	2.73	22.06%

#	Unit	Ref	UD	D	$\Delta d / \|d_U\|$ [%]
8	[mm]	d	1.81	2.47	36.75%
9	[mm]	d	5.03	7.34	45.79%
10	[mm]	d	7.55	11.78	56.16%
11	[mm]	d	8.03	11.64	44.91%
12	[mm]	d	7.74	10.74	38.76%
13	[mm]	d	5.23	7.02	34.19%
14	[mm]	d	1.86	2.46	32.28%

Fig. 15 | Vertical displacement results.

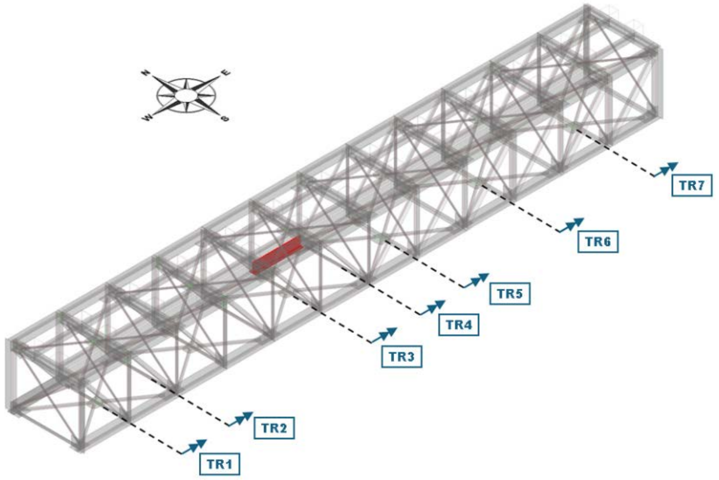

#	Unit	Ref	UD	D	$\Delta\theta / \|\theta_U\|$ [%]
TR1	[º]	θ	0.06	0.07	20.00%
TR2	[º]	θ	0.06	0.05	-18.07%
TR3	[º]	θ	0.05	-0.04	-177.35%
TR4	[º]	θ	0.03	-0.03	-195.41%
TR5	[º]	θ	0.02	-0.02	-229.93%
TR6	[º]	θ	0.02	0.00	-108.61%
TR7	[º]	θ	0.03	0.02	-29.42%

Fig. 16 | Distortion results.

The results of the measured strains are presented in Figures 17-22. Each figure details the monitored elements, including the unit of measurement for each sensor and whether the location is representative of axial forces (N) or both axial forces and bending moments (N, M). The absolute values recorded by each sensor are displayed for both undamaged (UD) and damaged (D) conditions, along with an indication of whether the values represent tension (T) or compression (C). The data were processed as described at the start of Section 1.4. This analysis enables the following conclusions to be drawn:

- <u>*Horizontal bracings*</u> (Fig. 17): The loss of the lower central chord triggers the development of ALPs through the development of tension and compression in the horizontal bracing system

near the affected area. These ALPs constitute a primary and critical mechanism for transferring loads from the northern to the southern part of the bridge.

- *Vertical bracings* (Fig. 18): The vertical bracing system plays a fundamental role in the activation of additional ALPs. However, it is important to note that only the vertical bracing closest to the damage fulfils this critical function. In the absence of the chord, the load on the affected northern section is transferred to the unaffected southern section through this system.
- *Verticals* (Fig. 19): The verticals have been identified as responsible for the ALPs. Analysis of load transfer between the affected (North) and unaffected (South) parts has been demonstrated by the load levels in these elements. The affected part (North) experiences a reduction in load, while the unaffected part (South) shows an increase. Although the verticals play a crucial role in maintaining the bridge's stability, the observed relative strain increments in these elements do not necessarily indicate critical importance.
- *Diagonals* (Fig. 20): The unloading of the diagonals in the affected part (North), along with the loading of those in the unaffected part (South), indicates that the diagonals also play a significant role as ALPs in this particular damage scenario. The load transfer between the two systems can be monitored through the load levels in the diagonals. While the verticals and diagonals are recognised as fundamental elements for bridge stability and have been identified as significant in the activation of ALPs, they are not considered critical, given the reduced $\Delta\varepsilon/\varepsilon_y$ ratios.
- *Chords* (Fig. 21 & Fig. 22): A chord failure unloads the neighbouring chords in the affected area and, conversely, significantly loads the chords in the unaffected part. The transfer of load from one part to the other is evident from the strain increments in the unaffected part (South) and the unloading of the chords near the damage in the affected part (North), highlighting the load transfer from North to South. Strain measurements near the connections, in the web and flange of the chords, enabled the identification of new mechanisms through which the ALPs are activated, with the presence of significant bending moments.

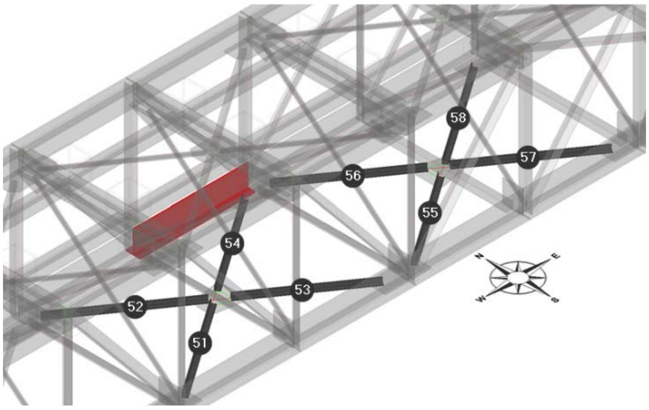

#	Unit	Ref	UD	D	$\Delta\varepsilon / \lvert\varepsilon_U\rvert$ [%]		$\Delta\varepsilon/\varepsilon_y$ [%]
51	[mε]	N	0.2320 T	0.8645 T	272.62%	↑	40.16%
52	[mε]	N	0.2627 T	1.2826 T	388.20%	↑	64.76%
53	[mε]	N	0.3195 T	0.9644 T	201.87%	↑	40.95%
54	[mε]	N	0.2319 T	1.4500 T	525.28%	↑	77.35%
55	[mε]	N	0.2511 T	0.6352 T	152.97%	↑	24.39%
56	[mε]	N	0.3146 T	-0.2403 C	-176.39%	↻	-35.24%
57	[mε]	N	0.2515 T	-0.0892 C	-135.46%	↻	-21.63%
58	[mε]	N	0.2288 T	0.4148 T	81.33%	↑	11.81%

Fig. 17 | Strain results for the horizontal lower bracing monitored elements.

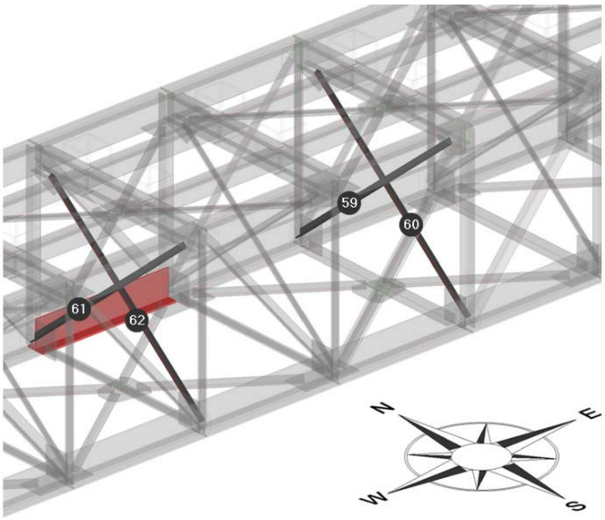

#	Unit	Ref	UD	D	$\Delta\varepsilon / \lvert\varepsilon_U\rvert$ [%]		$\Delta\varepsilon/\varepsilon_y$ [%]
59	[mε]	N	-0.0625 C	-0.1460 C	-133.53%	↑	-5.30%
60	[mε]	N	-0.1550 C	-0.0486 C	68.61%	↓	6.75%
61	[mε]	N	-0.1161 C	0.2887 T	348.73%	↻	25.71%
62	[mε]	N	-0.0824 C	-0.5549 C	-573.26%	↑	-30.00%

Fig. 18 | Strain results for the vertical bracing monitored elements.

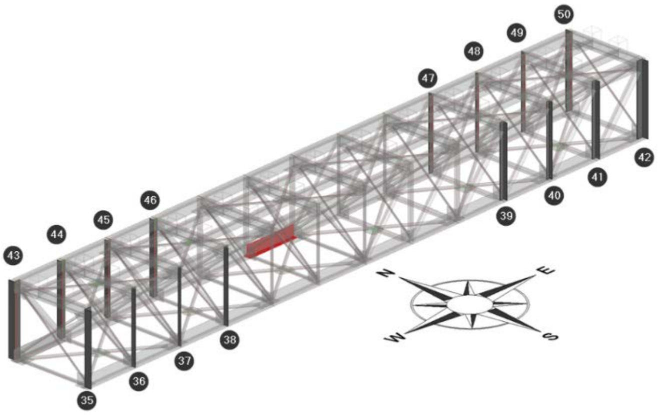

#	Unit	Ref	UD	D	$\Delta\varepsilon / \lvert\varepsilon_U\rvert$ [%]		$\Delta\varepsilon/\varepsilon_y$ [%]
35	[mε]	N,M	-0.0436 C	-0.0220 C	49.59%	↓	1.37%
36	[mε]	N,M	-0.1866 C	-0.2285 C	-22.46%	↑	-2.66%
37	[mε]	N,M	-0.3785 C	-0.4930 C	-30.27%	↑	-7.27%
38	[mε]	N,M	-0.2183 C	-0.2820 C	-29.19%	↑	-4.05%
39	[mε]	N,M	-0.2087 C	-0.2410 C	-15.43%	↑	-2.04%
40	[mε]	N,M	-0.4532 C	-0.5335 C	-17.72%	↑	-5.10%
41	[mε]	N,M	-0.2489 C	-0.2685 C	-7.89%	↑	-1.25%
42	[mε]	N,M	-0.0521 C	-0.0463 C	11.23%	↓	0.37%

#	Unit	Ref	UD	D	$\Delta\varepsilon / \lvert\varepsilon_U\rvert$ [%]		$\Delta\varepsilon/\varepsilon_y$ [%]
43	[mε]	N,M	-0.0545 C	-0.0691 C	-26.69%	↑	-0.92%
44	[mε]	N,M	-0.2749 C	-0.2110 C	23.23%	↓	4.06%
45	[mε]	N,M	-0.4422 C	-0.3150 C	28.78%	↓	8.08%
46	[mε]	N,M	-0.2001 C	-0.1227 C	38.66%	↓	4.91%
47	[mε]	N,M	-0.2305 C	-0.1930 C	16.26%	↓	2.38%
48	[mε]	N,M	-0.4921 C	-0.4347 C	11.65%	↓	3.64%
49	[mε]	N,M	-0.2386 C	-0.2284 C	4.27%	↓	0.65%
50	[mε]	N,M	-0.0672 C	-0.0728 C	-8.31%	↑	-0.35%

Fig. 19 | Strain results for the vertical monitored elements.

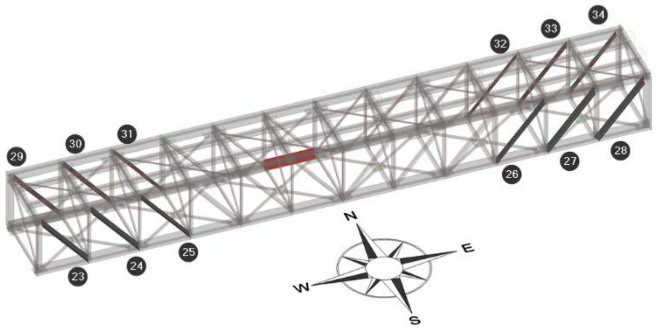

#	Unit	Ref	UD	D	$\Delta\varepsilon / \lvert\varepsilon_U\rvert$ [%]		$\Delta\varepsilon/\varepsilon_y$ [%]
23	[mε]	N	0.4371 T	0.5351 T	22.43%	↑	6.23%
24	[mε]	N	0.4926 T	0.6182 T	25.51%	↑	7.98%
25	[mε]	N	0.6906 T	0.9026 T	30.69%	↑	13.46%
26	[mε]	N	0.6789 T	0.7646 T	12.62%	↑	5.44%
27	[mε]	N	0.4676 T	0.5104 T	9.16%	↑	2.72%
28	[mε]	N	0.4250 T	0.4550 T	7.05%	↑	1.90%

#	Unit	Ref	UD	D	$\Delta\varepsilon / \lvert\varepsilon_U\rvert$ [%]		$\Delta\varepsilon/\varepsilon_y$ [%]
29	[mε]	N	0.4469 T	0.3460 T	-22.57%	↓	-6.40%
30	[mε]	N	0.4692 T	0.3568 T	-23.94%	↓	-7.13%
31	[mε]	N	0.6639 T	0.4668 T	-29.69%	↓	-12.52%
32	[mε]	N	0.6633 T	0.5776 T	-12.92%	↓	-5.44%
33	[mε]	N	0.5183 T	0.4686 T	-9.58%	↓	-3.15%
34	[mε]	N	0.4086 T	0.3745 T	-8.35%	↓	-2.17%

Fig. 20 | Strain results for the diagonal monitored elements.

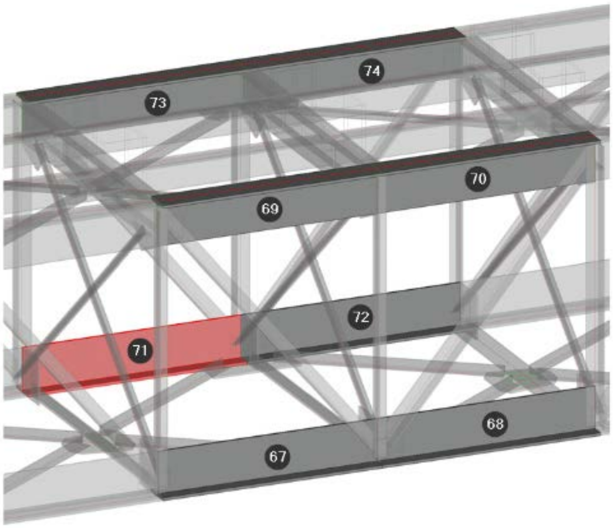

#	Measure	Ref	UD	D	Δε / |ε_u| [%]		Δε/ε_y [%]
67	[mε]	N	0.4090 T	0.5879 T	43.72%	↑	11.36%
68	[mε]	N	0.4038 T	0.5688 T	40.87%	↑	10.48%
69	[mε]	N	-0.3017 C	-0.5557 C	-84.20%	↑	-16.13%
70	[mε]	N	-0.3437 C	-0.5279 C	-53.60%	↑	-11.70%

#	Unit	Ref	UD	D	Δε / |ε_u| [%]		Δε/ε_y [%]
71	[mε]	N	0.3911 T	0.0058 T	-98.52%	↓	-24.47%
72	[mε]	N	0.4024 T	0.2527 T	-37.20%	↓	-9.50%
73	[mε]	N	-0.3205 C	-0.0494 C	84.60%	↓	17.22%
74	[mε]	N	-0.3098 C	-0.1416 C	54.27%	↓	10.68%

Fig. 21 | Strain results for the main chords in the centre of the gravity and centre of the length of the monitored elements, only representatives of the axial forces.

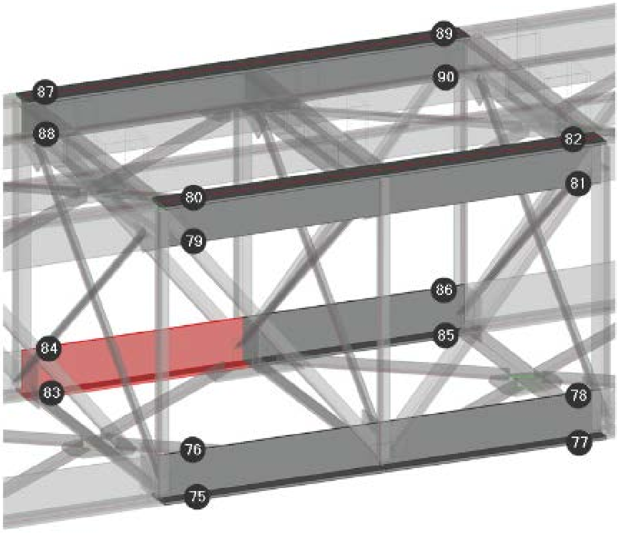

#	Unit	Ref	UD	D	Δε / |ε_u| [%]		Δε/ε_y [%]
75	[mε]	N,M	0.5225 T	0.7869 T	50.59%	↑	16.79%
76	[mε]	N,M	0.1943 T	0.1989 T	2.33%	↑	0.29%
77	[mε]	N,M	0.4851 T	0.6422 T	32.39%	↑	9.98%
78	[mε]	N,M	0.2403 T	0.4055 T	68.74%	↑	10.49%
79	[mε]	N,M	-0.4627 C	-0.7026 C	-51.84%	↑	-15.23%
80	[mε]	N,M	-0.1850 C	0.0610 T	133.00%	↻	15.62%
81	[mε]	N,M	-0.3750 C	-0.5610 C	-49.60%	↑	-11.81%
82	[mε]	N,M	-0.3900 C	-0.6530 C	-67.45%	↑	-16.70%

#	Unit	Ref	UD	D	Δε / |ε_u| [%]		Δε/ε_y [%]
83	[mε]	N,M	0.4815 T	0.0056 T	-98.84%	↓	-30.22%
84	[mε]	N,M	0.2704 T	0.0286 T	-89.42%	↓	-15.35%
85	[mε]	N,M	0.5085 T	0.3586 T	-29.49%	↓	-9.52%
86	[mε]	N,M	0.1732 T	-0.0026 C	-101.50%	↻	-11.16%
87	[mε]	N,M	-0.4745 C	-0.4665 C	1.68%	↓	0.51%
88	[mε]	N,M	-0.1486 C	0.5623 T	478.43%	↻	45.14%
89	[mε]	N,M	-0.4083 C	-0.2018 C	50.56%	↓	13.11%
90	[mε]	N,M	-0.1843 C	0.0603 T	132.71%	↻	15.53%

Fig. 22 | Strain results for the main chords near joints and far from the centre of gravity of the monitored elements, representing both axial and bending forces.

In summary, for a Pratt truss in undamaged conditions, the **chords** are responsible for transmitting global **bending moments** induced by vertical loads. This transmission occurs through **compression and tension** in the upper and lower chords, respectively (in the case of positive deflections, as in the scenario studied**). In the event of a failure of the lower central chord**, the system activates new mechanisms for transferring these global bending forces. Firstly, part of the **vertical load is transferred to the unaffected zones via the bracings through a torsional mechanism**. Secondly, **local bending mechanisms at the element level** in the chords near the damaged area are activated. Both the bracings and the chords exhibit significant strain increments.

In the undamaged state of the bridge, **the verticals and diagonals** are responsible for transmitting shear forces. However, the failure of the lower chord does not interrupt shear transmission across the structure. While the verticals and diagonals contribute to bridge stability and are components of the ALPs developed by the bridge, the measured strain increments were not substantial, and therefore, these elements do not constitute critical load paths in this damage scenario.

1.4.3 DS – Loss of a diagonal

Fig. 23 shows the definition of this damage scenario.

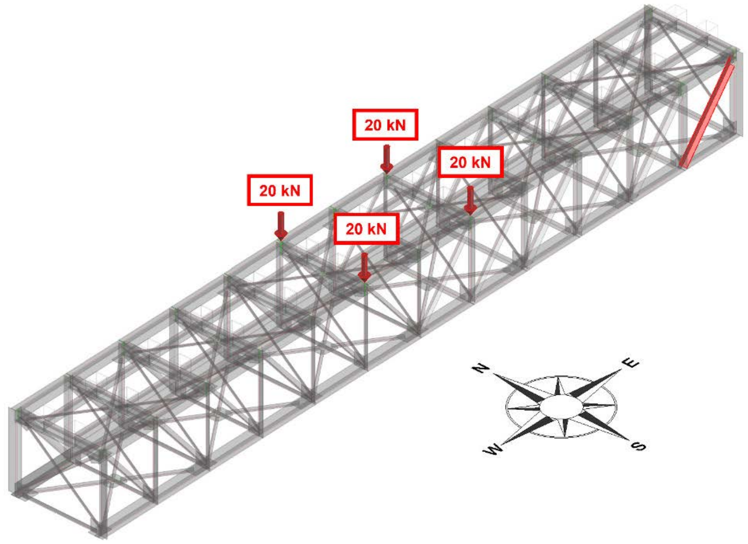

Fig. 23 | Bridge, loads and damage scenario identified by the red colour element.

The results obtained for vertical displacements and distortions are shown in Fig. 24 and Fig. 25. Each figure includes the references and units for the sensor measurements: d for displacements (in mm) and θ for distortions (in degrees). Additionally, the absolute values recorded by each sensor in undamaged (UD) and damaged (D) conditions are presented, along with the processed results, as discussed at the beginning of Section 1.4. From the results, it can be concluded that:

- The loss of the diagonal at the south-eastern end of the bridge has been shown to significantly impact the vertical stiffness of the south truss, resulting in substantial displacements throughout the entire structure. These displacements are particularly pronounced at the ends of the structure, with a notable increase observed in the area where the diagonal is removed. In addition to these findings, the northeastern part of the structure has also shown an increase in vertical displacements. Conversely, the area diagonally opposite the affected region has experienced a decrease in displacements following the loss of the diagonal. These observations suggest an asymmetric redistribution of loads in both the north-south and east-west directions.
- In the undamaged bridge, the loads are distributed symmetrically with respect to the West-East and South-North axes. However, with the loss of the external diagonal, a modification occurs in the torsional (more globally pronounced) and flexural (more locally pronounced) responses of the final module of the bridge, leading to an asymmetric redistribution of loads and clear torsional effects along the entire length of the bridge.
- The loss of the diagonal at the eastern end of the bridge induces distortions, confirming the torsional effect after the element removal.

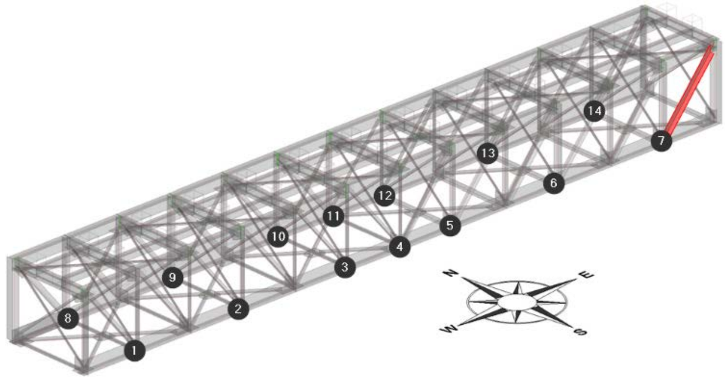

#	Unit	Ref	UD	D	$\Delta d / \lvert d_U \rvert$ [%]
1	[mm]	d	2.45	2.70	10.14%
2	[mm]	d	5.73	6.12	6.75%
3	[mm]	d	8.19	8.66	5.70%
4	[mm]	d	8.43	8.96	6.28%
5	[mm]	d	7.97	8.56	7.41%
6	[mm]	d	5.52	6.26	13.50%
7	[mm]	d	2.24	3.14	40.27%

#	Unit	Ref	UD	D	$\Delta d / \lvert d_U \rvert$ [%]
8	[mm]	d	1.97	1.79	-8.77%
9	[mm]	d	5.19	5.07	-2.23%
10	[mm]	d	7.71	7.67	-0.46%
11	[mm]	d	8.19	8.19	-0.01%
12	[mm]	d	7.90	7.92	0.26%
13	[mm]	d	5.31	5.38	1.31%
14	[mm]	d	1.89	1.96	4.18%

Fig. 24 | Vertical displacement results.

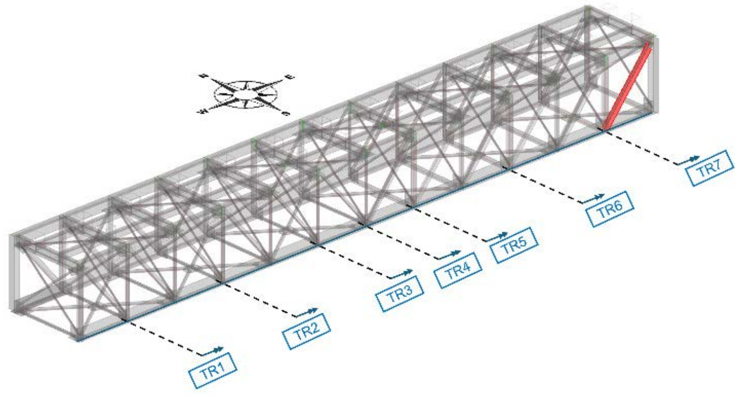

#	Unit	Ref	UD	D	$\Delta\theta / \lvert\theta_U\rvert$ [%]
TR1	[º]	θ	0.039	0.074	87.71%
TR2	[º]	θ	0.044	0.086	92.58%
TR3	[º]	θ	0.040	0.081	103.68%
TR4	[º]	θ	0.020	0.063	218.27%
TR5	[º]	θ	0.006	0.052	838.80%
TR6	[º]	θ	0.017	0.072	333.38%
TR7	[º]	θ	0.029	0.096	232.82%

Fig. 25 | Distortion results.

The results of the measured strains are presented in Figures 26-32. Each figure details the monitored elements, including the unit of measurement for each sensor and whether the location is representative of axial forces (N) or both axial forces and moments (N, M). The absolute values recorded by each sensor are displayed for both undamaged (UD) and damaged (D) conditions, along with an indication of whether the values represent tension (T) or compression (C). The data were processed as described at the start of Section 1.4. This analysis enables the following conclusions to be drawn:

- *Horizontal bracings* (Fig. 26): The lower horizontal bracing system contributes to load redistribution and ALPs activation by increasing its axial load to control the torsion generated by the loss of the diagonal. As a result, the distortions in the affected zone activate the bracings in a clearly defined pattern, with load increases in the Southwest-Northeast oriented elements and unloading in the Northwest-Southeast oriented elements. This alternating pattern is characteristic of the activation of a strong torsional mechanism. Additionally, the degree of system activation depends on the distance from the affected zone, which is why the East elements exhibit more significant $\Delta\varepsilon/\varepsilon_y$ ratios than those on the West. It is important to note that the magnitudes of the $\Delta\varepsilon/\varepsilon_y$ ratios for the bracing system range from -2.32 % to 2.04 %, indicating that, while they are not a critical element in load redistribution, they do play a significant role in it.
- *Vertical bracings* (Fig. 27): The $\Delta\varepsilon/\varepsilon_y$ ratios in the vertical bracings range from -0.58 % to 0.51 %, indicating that they cannot be considered fundamental elements in the activation of ALPs, at least for those far from the damage zone, which are the only ones monitored. Clearly, vertical bracings located closer to the damage zone experience more significant activation, as will be demonstrated in Section 2 with computational models.
- *Verticals* (Fig. 28): The loss of the diagonal alters the shear force transfer path, causing the nearby verticals to take on higher loads. In contrast, the verticals diagonally opposite (North-West) to the affected zone experience unloading in the vertical forces, further corroborating the torsional effects and inducing asymmetric load distribution. The loss of the outer diagonal activates Vierendeel-type resistance mechanisms. Without the diagonal removed, equilibrium can no longer be maintained by axial forces alone, resulting in the appearance of bending moments near joints. As a result, the verticals activate their bending capacity as a mechanism for load redistribution, thus activating a new alternative load path (ALP). In conclusion, it can be established that the verticals are a critical element in the redistribution of loads and the activation of ALPs in the event of diagonal failure.
- *Diagonals* (Fig. 29): Values in the ratios $\Delta\varepsilon/\varepsilon_y$ reaching 8.01% indicate that the external diagonals are a very crucial element in the redistribution of loads in the event of a diagonal removal. The ALPs activation occurs, on the one hand, through the redirection of shear forces towards the diagonals closest to the affected zone (sensors 27 and 34). Judging by the relative increments with respect to the yielding strain, the ALP resulting from this response is the most important in the redistribution of loads on the diagonals. On the other hand, the response

highlights how, with the removal of the outer diagonal, the vertical stiffness of the bridge is affected, causing the torsional effects mentioned above. Therefore, an asymmetrical load distribution is generated where the diagonals furthest away in the affected truss exhibit loading processes while the diagonals furthest away in the unaffected truss exhibit unloading processes.

- Chords (Fig. 30 & Fig. 31 & Fig. 32): The $\Delta\varepsilon/\varepsilon_y$ ratios in the central chords, both lower and upper, barely exceed 1.00%, indicating the limited importance of the central chords, that are located far from the failure, in load redistribution and ALP activation. However, they help to identify alternative paths through which the load transfers from one truss to another by the activation of the torsional behaviour. In the chords closest to the removed diagonal, $\Delta\varepsilon/\varepsilon_y$ ratios ranging from -6.68% to 22.19%, along with relative strain increments to the yielding strain of 21.64% in the vertical adjacent to the removed diagonal, suggest that Vierendeel-type behaviour is one of the primary ALP activation mechanisms. Without the diagonal, the truss is unable to transfer shear forces locally through axial forces alone. As a result, the load redistributes to the diagonals and verticals of the north truss and locally triggers bending mechanisms in the chords and verticals on the south side. This phenomenon is also associated with significant displacements, characteristic of Vierendeel-type behaviour.

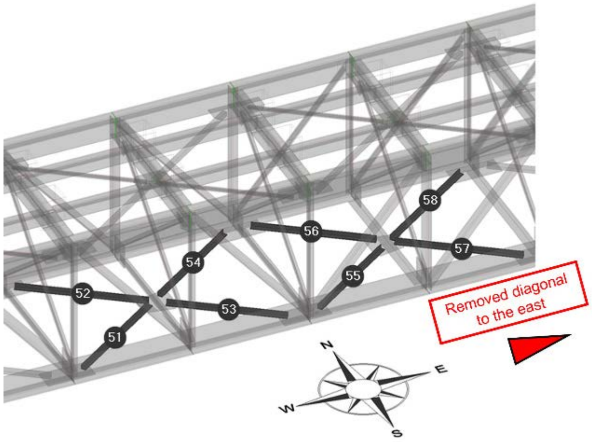

#	Unit	Ref	UD	D	Δε / |εu| [%]		Δε/εy [%]
51	[mε]	N	0.2337 T	0.2514 T	7.57%	↑	1.12%
52	[mε]	N	0.2655 T	0.2470 T	-6.96%	↓	-1.17%
53	[mε]	N	0.3146 T	0.2902 T	-7.77%	↓	-1.55%
54	[mε]	N	0.2548 T	0.2820 T	10.67%	↑	1.73%
55	[mε]	N	0.2560 T	0.2842 T	11.00%	↑	1.79%
56	[mε]	N	0.3102 T	0.2815 T	-9.27%	↓	-1.83%
57	[mε]	N	0.2403 T	0.2038 T	-15.18%	↓	-2.32%
58	[mε]	N	0.2380 T	0.2701 T	13.49%	↑	2.04%

Fig. 26 | Strain results for the horizontal lower bracing monitored elements.

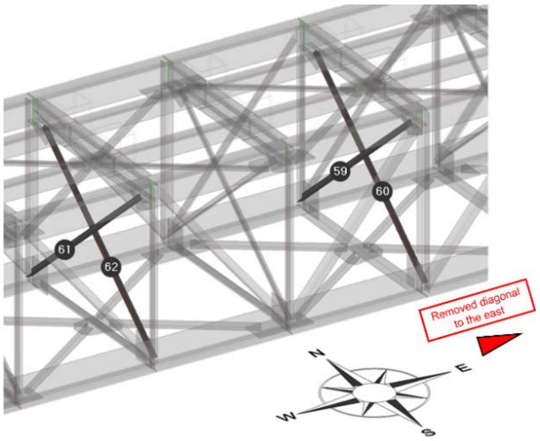

#	Unit	Ref	UD	D	Δε / |εu| [%]		Δε/εy [%]
59	[mε]	N	-0.0606 C	-0.0526 C	13.23%	↓	0.51%
60	[mε]	N	-0.1650 C	-0.1742 C	-5.58%	↑	-0.58%
61	[mε]	N	-0.1204 C	-0.1170 C	2.81%	↓	0.21%
62	[mε]	N	-0.0807 C	-0.0843 C	-4.47%	↑	-0.23%

Fig. 27 | Strain results for the vertical bracing monitored elements.

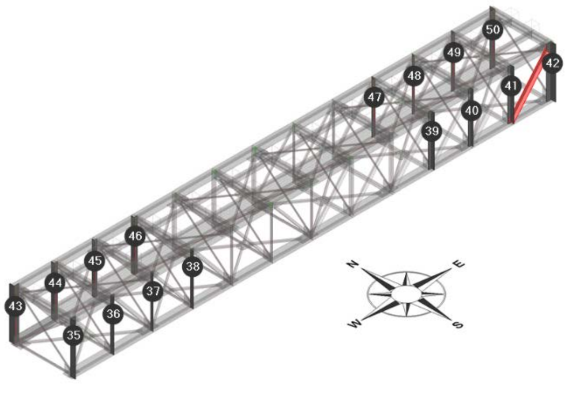

#	Unit	Ref	UD	D	Δε / |ε_U| [%]		Δε/ε_y [%]
35	[mε]	N,M	-0.0459 C	-0.0464 C	-1.05%	↑	-0.03%
36	[mε]	N,M	-0.1886 C	-0.1913 C	-1.41%	↑	-0.17%
37	[mε]	N,M	-0.3837 C	-0.3873 C	-0.94%	↑	-0.23%
38	[mε]	N,M	-0.2206 C	-0.2213 C	-0.31%	↑	-0.04%
39	[mε]	N,M	-0.2057 C	-0.2099 C	-2.04%	↑	-0.27%
40	[mε]	N,M	-0.4439 C	-0.5002 C	-12.68%	↑	-3.57%
41	[mε]	N,M	-0.2429 C	-0.1325 C	45.46%	↓	7.01%
42	[mε]	N,M	-0.0483 C	0.2925 T	705.51%	↻	21.64%

#	Unit	Ref	UD	D	Δε / |ε_U| [%]		Δε/ε_y [%]
43	[mε]	N,M	-0.0508 C	-0.0494 C	2.77%	↓	0.09%
44	[mε]	N,M	-0.2714 C	-0.2680 C	1.25%	↓	0.22%
45	[mε]	N,M	-0.4339 C	-0.4298 C	0.95%	↓	0.26%
46	[mε]	N,M	-0.1949 C	-0.1936 C	0.68%	↓	0.08%
47	[mε]	N,M	-0.2316 C	-0.2311 C	0.22%	↓	0.03%
48	[mε]	N,M	-0.4986 C	-0.5034 C	-0.96%	↑	-0.30%
49	[mε]	N,M	-0.2414 C	-0.2493 C	-3.30%	↑	-0.51%
50	[mε]	N,M	-0.0668 C	-0.0550 C	17.59%	↓	0.75%

Fig. 28 | Strain results for the vertical monitored elements.

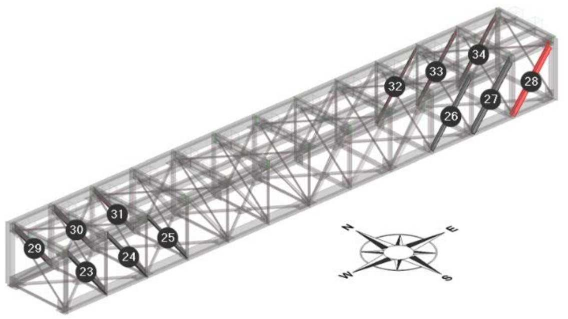

#	Unit	Ref	UD	D	Δε / |ε_U| [%]		Δε/ε_y [%]
23	[mε]	N	0.4439 T	0.4463 T	0.54%	↑	0.15%
24	[mε]	N	0.4994 T	0.5028 T	0.69%	↑	0.22%
25	[mε]	N	0.7003 T	0.7053 T	0.72%	↑	0.32%
26	[mε]	N	0.6697 T	0.6643 T	-0.80%	↓	-0.34%
27	[mε]	N	0.4588 T	0.5850 T	27.50%	↑	8.01%
28	[mε]	N	0.4185 T	0.0012 T	-99.72%	↓	-26.50%

#	Unit	Ref	UD	D	Δε / |ε_U| [%]		Δε/ε_y [%]
29	[mε]	N	0.4443 T	0.4403 T	-0.90%	↓	-0.25%
30	[mε]	N	0.4615 T	0.4580 T	-0.74%	↓	-0.22%
31	[mε]	N	0.6551 T	0.6503 T	-0.72%	↓	-0.30%
32	[mε]	N	0.6692 T	0.6621 T	-1.06%	↓	-0.45%
33	[mε]	N	0.5248 T	0.5390 T	2.70%	↑	0.90%
34	[mε]	N	0.4225 T	0.4732 T	12.02%	↑	3.22%

Fig. 29 | Strain results for the diagonal monitored elements.

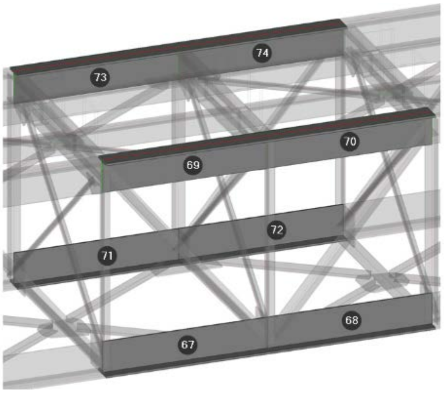

#	Measure	Ref	UD	D	Δε / |ε_U| [%]		Δε/ε_y [%]
67	[mε]	N	0.4118 T	0.4089 T	-0.70%	↓	-0.18%
68	[mε]	N	0.4039 T	0.3959 T	-1.99%	↓	-0.51%
69	[mε]	N	-0.3091 C	-0.3060 C	0.99%	↓	0.19%
70	[mε]	N	-0.3578 C	-0.3367 C	5.88%	↓	1.34%

#	Unit	Ref	UD	D	Δε / |ε_U| [%]		Δε/ε_y [%]
71	[mε]	N	0.3986 T	0.4016 T	0.77%	↑	0.19%
72	[mε]	N	0.4067 T	0.4154 T	2.13%	↑	0.55%
73	[mε]	N	-0.3339 C	-0.3355 C	-0.46%	↑	-0.10%
74	[mε]	N	-0.3206 C	-0.3270 C	-1.99%	↑	-0.40%

Fig. 30 | Strain results for the main chords in the centre of the gravity and centre of the length of the monitored elements, only representatives of the axial forces.

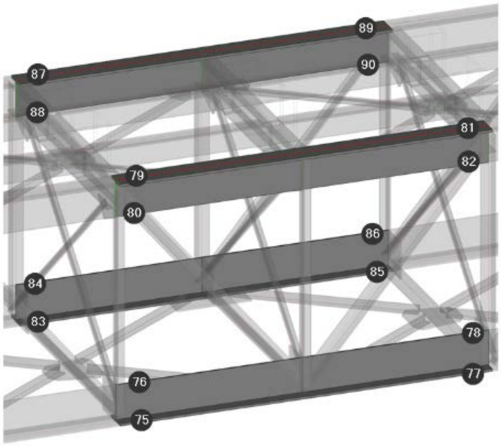

#	Unit	Ref	UD	D	Δε / |ε_U| [%]		Δε/ε_y [%]
75	[mε]	N,M	0.5292 T	0.5257 T	-0.66%	↓	-0.22%
76	[mε]	N,M	0.1897 T	0.1876 T	-1.14%	↓	-0.14%
77	[mε]	N,M	0.4826 T	0.4743 T	-1.71%	↓	-0.52%
78	[mε]	N,M	0.2469 T	0.2405 T	-2.62%	↓	-0.41%
79	[mε]	N,M	-0.4950 C	-0.4870 C	1.61%	↓	0.51%
80	[mε]	N,M	-0.2514 C	-0.2410 C	4.15%	↓	0.66%
81	[mε]	N,M	-0.3882 C	-0.3814 C	1.76%	↓	0.43%
82	[mε]	N,M	-0.4271 C	-0.4092 C	4.19%	↓	1.14%

#	Unit	Ref	UD	D	Δε / |ε_U| [%]		Δε/ε_y [%]
83	[mε]	N,M	0.4824 T	0.4867 T	0.90%	↑	0.27%
84	[mε]	N,M	0.2818 T	0.2856 T	1.33%	↑	0.24%
85	[mε]	N,M	0.5164 T	0.5251 T	1.68%	↑	0.55%
86	[mε]	N,M	0.1721 T	0.1793 T	4.22%	↑	0.46%
87	[mε]	N,M	-0.5015 C	-0.5020 C	-0.09%	↑	-0.03%
88	[mε]	N,M	-0.1702 C	-0.1250 C	26.54%	↓	2.87%
89	[mε]	N,M	-0.4273 C	-0.4340 C	-1.56%	↑	-0.42%
90	[mε]	N,M	-0.1939 C	-0.2027 C	-4.57%	↑	-0.56%

Fig. 31 | Strain results for the main chords near joints and far from the centre of gravity of the monitored elements, representing both axial and bending forces.

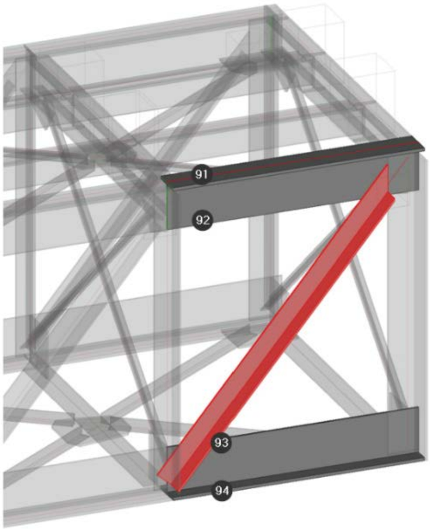

#	Unit	Ref	UD	D	Δε / |ε$_U$| [%]		Δε/ε$_y$ [%]
91	[mε]	N,M	-0.0401 C	-0.1188 C	-196.10%	↑	-5.00%
92	[mε]	N,M	-0.0773 C	0.2721 T	451.81%	↻	22.19%
93	[mε]	N,M	-0.1266 C	-0.2317 C	-83.08%	↑	-6.68%
94	[mε]	N,M	0.0060 T	0.1274 T	2024.80%	↑	7.71%

Fig. 32 | Strain results for the main chords near joints and far from the centre of gravity of the monitored elements (this case close to the removed element), representing both axial and bending forces.

In summary, in a Pratt truss, the verticals and diagonals are the key elements for transferring shear forces. When a diagonal fails, the affected truss loses stiffness, leading to increased vertical displacements and distortions in the area near the damage. As a result, the structure must redistribute the forces, activating various ALPs. The bridge experiences torsion, which causes an asymmetric redistribution of loads: elements diagonally opposite the damaged zone are unloaded, while those nearer the damage bear increased loads. This response is accompanied by Vierendeel-type behaviour, where the ALP is defined by the presence of bending moments in chords and verticals adjacent to the removed diagonal.

1.4.4 DS – Loss of a vertical

Fig. 33 shows the definition of this damage scenario.

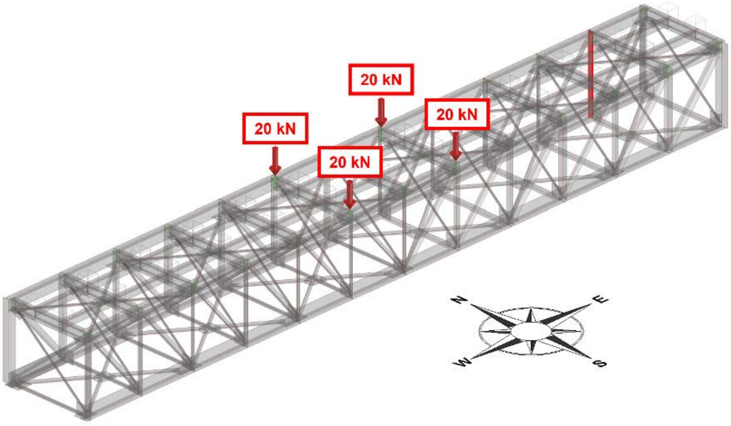

Fig. 33 | Bridge, loads and damage scenario identified by the red colour element.

The results obtained for vertical displacements and distortions are shown in Fig. 34 and Fig. 35. Each figure includes the references and units for the sensor measurements: d for displacements (in mm) and θ for distortions (in degrees). Additionally, the absolute values recorded by each sensor in undamaged (UD) and damaged (D) conditions are presented, along with the processed results, as discussed at the beginning of Section 1.4. From the results, it can be concluded that:

- The removal of the vertical at the northeast end leads to increased vertical displacements, particularly concentrated in the affected area and on the northwest side of the bridge, while the southwest side exhibits a reduction in vertical displacements.
- These results indicate, on the one hand, a change in stiffness due to the loss of the vertical and the redistribution of loads and displacements in both the north-south and west-east directions.

This results in a global torsional behaviour that induces an asymmetric distribution of loads and distortions.

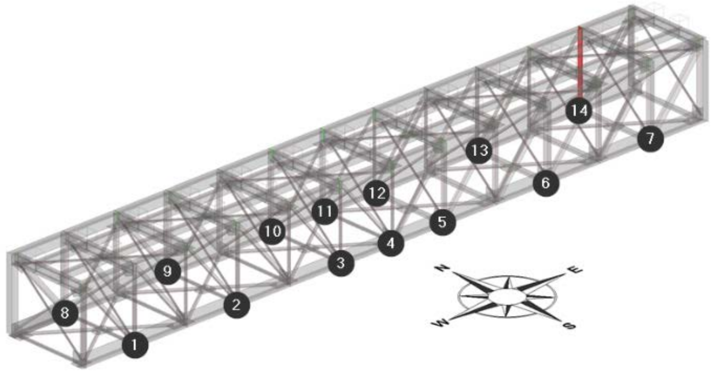

#	Unit	Ref	UD	D	$\Delta d / \lvert d_U \rvert$ [%]
1	[mm]	d	2.48	2.29	-7.67%
2	[mm]	d	5.74	5.63	-1.99%
3	[mm]	d	8.14	8.11	-0.37%
4	[mm]	d	8.36	8.35	-0.09%
5	[mm]	d	7.93	7.94	0.08%
6	[mm]	d	5.49	5.54	1.02%
7	[mm]	d	2.22	2.28	2.56%

#	Unit	Ref	UD	D	$\Delta d / \lvert d_U \rvert$ [%]
8	[mm]	d	1.82	2.10	15.34%
9	[mm]	d	4.97	5.36	7.82%
10	[mm]	d	7.54	8.05	6.84%
11	[mm]	d	8.02	8.60	7.33%
12	[mm]	d	7.69	8.33	8.25%
13	[mm]	d	5.13	5.91	15.23%
14	[mm]	d	1.83	1.65	-9.68%

Fig. 34 | Vertical displacement results.

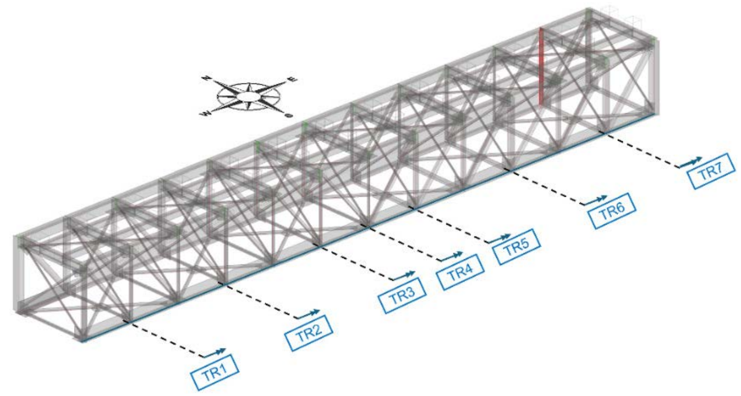

#	Unit	Ref	UD	D	$\Delta\theta / \lvert \theta_U \rvert$ [%]
TR1	[º]	θ	0.053	0.015	-71.98%
TR2	[º]	θ	0.063	0.022	-64.93%
TR3	[º]	θ	0.050	0.005	-89.87%
TR4	[º]	θ	0.028	-0.021	-173.88%
TR5	[º]	θ	0.020	-0.032	-261.50%
TR6	[º]	θ	0.029	-0.030	-204.37%
TR7	[º]	θ	0.032	0.052	58.95%

Fig. 35 | Distortion results.

The results of the measured strains are presented in Figures 36-42. Each figure details the monitored elements, including the unit of measurement for each sensor and whether the location is representative of axial forces (N) or both axial forces and moments (N, M). The absolute values recorded by each sensor are displayed for both undamaged (UD) and damaged (D) conditions, along with an indication of whether the values represent tension (T) or compression (C). The data were processed as described at the start of Section 1.4. This analysis enables the following conclusions to be drawn:

- *Horizontal bracings* (Fig. 36): The lower horizontal bracing system contributes to the load redistribution and ALP activation through increases in axial forces, helping to control the torsion generated by the loss of the vertical. As a result, the distortions in the affected zone activate the bracing in a distinct pattern: unloading along the Southwest-Northeast axis and loading along the Northwest-Southeast axis. This alternating pattern is characteristic of the activation of a torsional-resistant mechanism. Moreover, the degree of activation depends on the distance from the affected zone, which explains why the eastern elements exhibit more significant $\Delta\varepsilon/\varepsilon_y$ ratios than the western ones. The magnitudes of the $\Delta\varepsilon/\varepsilon_y$ ratios for the bracing system range from -1.96 % to 2.00 %, indicating that, while they are not critical elements in the load redistribution (they are located far from the failure), they still contribute to it.
- *Vertical bracings* (Fig. 37): $\Delta\varepsilon/\varepsilon_y$ ratios in the vertical bracings (located far from the DS) range from -0.39 % to 0.50 %, indicating that they cannot be considered fundamental elements in the activation of ALPs. However, their response helps to understand how the structure, on a global scale, reacts to the loss of the vertical. In the event of a rotation towards the damage zone, the vertical bracing is activated as a control mechanism. Consequently, the elements connecting the lower chords on the north side to the upper chords on the south side are loaded, while those connecting the lower chords on the south side to the upper chords on the north side are unloaded.

- *Verticals* (Fig. 38 The loss of the vertical affects the stiffness on one side of the bridge, modifying the vertical force transfer path. The verticals adjacent to the affected zone take on more load, while the verticals diagonally opposite to the affected zone (South-West) experience unloading processes due to the torsional effects in the bridge, leading to an asymmetric load distribution (see Fig. 37). With the loss of the vertical, equilibrium can no longer be maintained solely through axial forces in the areas near the damage. Bending mechanisms are activated, causing bending moments at the joints and initiating a new alternative load path (ALP). $\Delta\varepsilon/\varepsilon_y$ ratios exceeding 1.00 % highlight the significance of the verticals in load redistribution and how ALPs are activated.
- *Diagonals* (Fig. 39): Upon the loss of the vertical, load redistribution and ALPs activation follow three distinct mechanisms based on their $\Delta\varepsilon/\varepsilon_y$ ratios: (1) $\Delta\varepsilon/\varepsilon_y$ ratios of -4.59% and -7.07% indicate that the diagonals immediately adjacent to the removed vertical are unloaded, initiating a bending resistance mechanism in conjunction with the chords, similar to Vierendeel-type behaviour; (2) the loss of the vertical, forces the bridge to redirect shear forces towards the diagonals near the affected area, loading them and leading to further development of tensile stresses, as evidenced by $\Delta\varepsilon/\varepsilon_y$ ratios higher than 1.00%; and (3) the torsion-induced asymmetric load redistribution results in unloading the diagonals diagonally opposite to the affected zone. In conclusion, relative increments with respect to the yielding strain ranging from -7.07% to 4.01% highlight the crucial role of the diagonals in load redistribution.
- *Chords* (Fig. 40 & Fig. 41 & Fig. 42): The $\Delta\varepsilon/\varepsilon_y$ ratios in the central chords, both lower and upper, do not exceed values of 1.00%. This indicates the low importance of the central chords, far from the failure, in the load redistribution and the ALPs activation. However, they allow the identification of alternative paths where the load transfers from one truss to another as a result of the loss of the vertical in the north truss. The redistribution of loads in the face of the vertical loss does not occur through the activation of bending mechanisms in the chords away from the failure. Locally, near the damaged area, the bridge responds to the failure of the vertical through bending mechanisms, similar to Vierendeel-type behaviour. Judging by the ratio values that reach up to 41.97% in the chords, this mechanism becomes the most significant and critical activation mechanism for this failure scenario. Without the vertical, the truss cannot transfer loads by axial forces alone, and the new frame formed by diagonals and chords responds to bending and displacement mechanisms through Vierendeel-type.

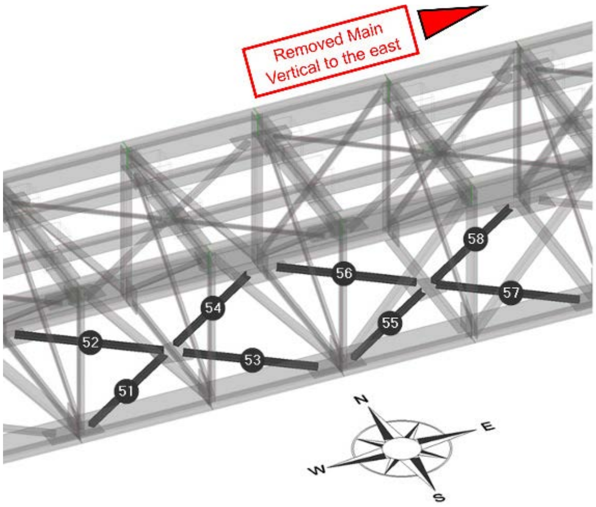

#	Unit	Ref	UD	D	$\Delta\varepsilon / \lvert\varepsilon_u\rvert$ [%]		$\Delta\varepsilon/\varepsilon_y$ [%]
51	[mε]	N	0.2334 T	0.2172 T	-6.95%	↓	-1.03%
52	[mε]	N	0.2586 T	0.2741 T	5.99%	↑	0.98%
53	[mε]	N	0.3185 T	0.3393 T	6.54%	↑	1.32%
54	[mε]	N	0.2320 T	0.2104 T	-9.28%	↓	-1.37%
55	[mε]	N	0.2498 T	0.2227 T	-10.82%	↓	-1.72%
56	[mε]	N	0.3102 T	0.3348 T	7.95%	↑	1.57%
57	[mε]	N	0.2479 T	0.2794 T	12.72%	↑	2.00%
58	[mε]	N	0.2248 T	0.1939 T	-13.75%	↓	-1.96%

Fig. 36 | Strain results for the horizontal lower bracing monitored elements.

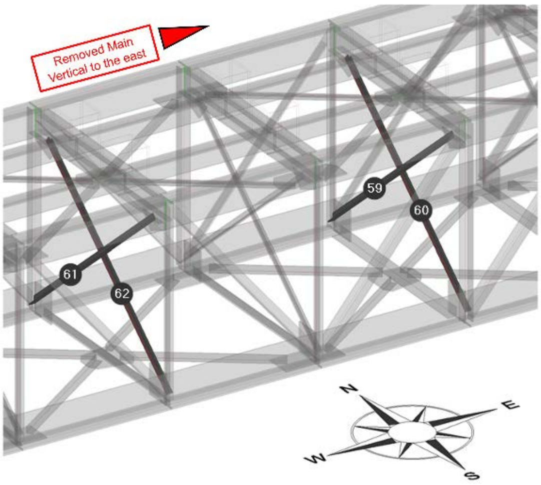

#	Unit	Ref	UD	D	Δε / |ε_U| [%]		Δε/ε_y [%]
59	[mε]	N	-0.0624 C	-0.0686 C	-9.86%	↑	-0.39%
60	[mε]	N	-0.1546 C	-0.1467 C	5.08%	↓	0.50%
61	[mε]	N	-0.1178 C	-0.1195 C	-1.44%	↑	-0.11%
62	[mε]	N	-0.0840 C	-0.0815 C	2.98%	↓	0.16%

Fig. 37 | Strain results for the vertical bracing monitored elements.

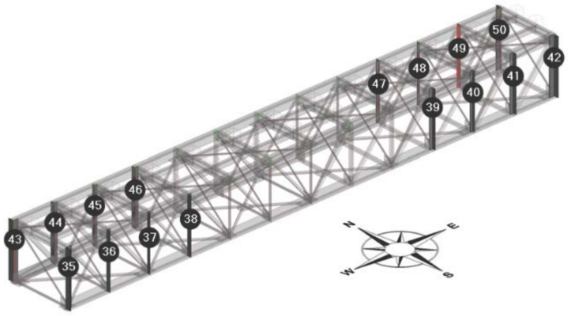

#	Unit	Ref	UD	D	Δε / |ε_U| [%]		Δε/ε_y [%]
35	[mε]	N,M	-0.0452 C	-0.0439 C	2.77%	↓	0.08%
36	[mε]	N,M	-0.1891 C	-0.1879 C	0.62%	↓	0.07%
37	[mε]	N,M	-0.3810 C	-0.3775 C	0.92%	↓	0.22%
38	[mε]	N,M	-0.2216 C	-0.2204 C	0.55%	↓	0.08%
39	[mε]	N,M	-0.2090 C	-0.2084 C	0.31%	↓	0.04%
40	[mε]	N,M	-0.4542 C	-0.4562 C	-0.45%	↑	-0.13%
41	[mε]	N,M	-0.2487 C	-0.2001 C	19.52%	↓	3.08%
42	[mε]	N,M	-0.0511 C	-0.0554 C	-8.24%	↑	-0.27%

#	Unit	Ref	UD	D	Δε / |ε_U| [%]		Δε/ε_y [%]
43	[mε]	N,M	-0.0569 C	-0.0583 C	-2.53%	↑	-0.09%
44	[mε]	N,M	-0.2739 C	-0.2757 C	-0.64%	↑	-0.11%
45	[mε]	N,M	-0.4440 C	-0.4468 C	-0.64%	↑	-0.18%
46	[mε]	N,M	-0.2038 C	-0.2047 C	-0.41%	↑	-0.05%
47	[mε]	N,M	-0.2315 C	-0.2183 C	5.72%	↓	0.84%
48	[mε]	N,M	-0.4955 C	-0.6260 C	-26.32%	↑	-8.28%
49	[mε]	N,M	-0.2316 C	0.0046 T	101.98%	↺	14.99%
50	[mε]	N,M	-0.0687 C	0.0421 T	161.19%	↺	7.04%

Fig. 38 | Strain results for the vertical monitored elements.

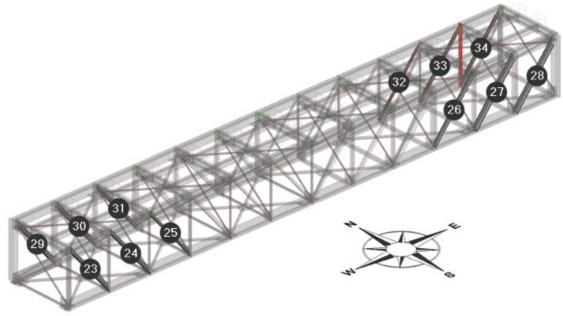

#	Unit	Ref	UD	D	Δε / |ε_U| [%]		Δε/ε_y [%]
23	[mε]	N	0.4352 T	0.4313 T	-0.89%	↓	-0.25%
24	[mε]	N	0.4924 T	0.4887 T	-0.76%	↓	-0.24%
25	[mε]	N	0.6883 T	0.6848 T	-0.51%	↓	-0.22%
26	[mε]	N	0.6764 T	0.6783 T	0.28%	↑	0.12%
27	[mε]	N	0.4649 T	0.5014 T	7.84%	↑	2.31%
28	[mε]	N	0.4246 T	0.4421 T	4.11%	↑	1.11%

#	Unit	Ref	UD	D	Δε / |ε_U| [%]		Δε/ε_y [%]
29	[mε]	N	0.4441 T	0.4456 T	0.33%	↑	0.09%
30	[mε]	N	0.4674 T	0.4690 T	0.34%	↑	0.10%
31	[mε]	N	0.6597 T	0.6607 T	0.15%	↑	0.06%
32	[mε]	N	0.6649 T	0.7280 T	9.49%	↑	4.01%
33	[mε]	N	0.5170 T	0.4447 T	-13.99%	↓	-4.59%
34	[mε]	N	0.4048 T	0.2935 T	-27.50%	↓	-7.07%

Fig. 39 | Strain results for the diagonal monitored elements.

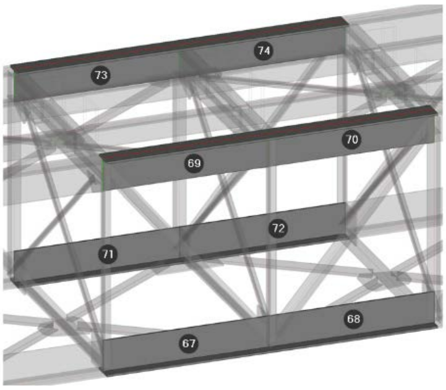

#	Measure	Ref	UD	D	Δε / |ε_U| [%]		Δε/ε_y [%]
67	[mε]	N	0.4088 T	0.4120 T	0.80%	↑	0.21%
68	[mε]	N	0.4031 T	0.4108 T	1.92%	↑	0.49%
69	[mε]	N	-0.3041 C	-0.3086 C	-1.47%	↑	-0.28%
70	[mε]	N	-0.3453 C	-0.3600 C	-4.25%	↑	-0.93%

#	Unit	Ref	UD	D	Δε / |ε_U| [%]		Δε/ε_y [%]
71	[mε]	N	0.3893 T	0.3847 T	-1.18%	↓	-0.29%
72	[mε]	N	0.4005 T	0.3913 T	-2.30%	↓	-0.59%
73	[mε]	N	-0.3235 C	-0.3194 C	1.27%	↓	0.26%
74	[mε]	N	-0.3123 C	-0.3019 C	3.32%	↓	0.66%

Fig. 40 | Strain results for the main chords in the centre of the gravity and centre of the length of the monitored elements, only representatives of the axial forces.

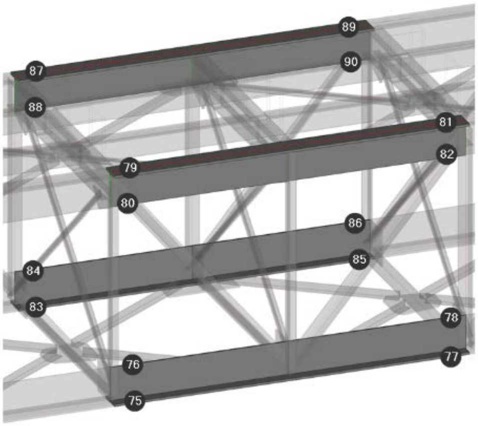

#	Unit	Ref	UD	D	$\Delta\varepsilon / \lvert\varepsilon_u\rvert$ [%]		$\Delta\varepsilon/\varepsilon_y$ [%]
75	[mε]	N,M	0.5207 T	0.5222 T	0.29%	↑	0.10%
76	[mε]	N,M	0.1934 T	0.1983 T	2.54%	↑	0.31%
77	[mε]	N,M	0.4813 T	0.4895 T	1.70%	↑	0.52%
78	[mε]	N,M	0.2395 T	0.2466 T	2.96%	↑	0.45%
79	[mε]	N,M	-0.4654 C	-0.4721 C	-1.44%	↑	-0.42%
80	[mε]	N,M	-0.1829 C	-0.1870 C	-2.23%	↑	-0.26%
81	[mε]	N,M	-0.3758 C	-0.3835 C	-2.04%	↑	-0.49%
82	[mε]	N,M	-0.3913 C	-0.4028 C	-2.95%	↑	-0.73%

#	Unit	Ref	UD	D	$\Delta\varepsilon / \lvert\varepsilon_u\rvert$ [%]		$\Delta\varepsilon/\varepsilon_y$ [%]
83	[mε]	N,M	0.4795 T	0.4752 T	-0.89%	↓	-0.27%
84	[mε]	N,M	0.2677 T	0.2631 T	-1.73%	↓	-0.29%
85	[mε]	N,M	0.5069 T	0.4969 T	-1.96%	↓	-0.63%
86	[mε]	N,M	0.1715 T	0.1625 T	-5.21%	↓	-0.57%
87	[mε]	N,M	-0.4788 C	-0.4732 C	1.15%	↓	0.35%
88	[mε]	N,M	-0.1463 C	-0.1365 C	6.72%	↓	0.62%
89	[mε]	N,M	-0.4107 C	-0.4031 C	1.85%	↓	0.48%
90	[mε]	N,M	-0.1873 C	-0.1742 C	6.97%	↓	0.83%

Fig. 41 | Strain results for the main chords near joints and far from the centre of gravity of the monitored elements, representing both axial and bending forces.

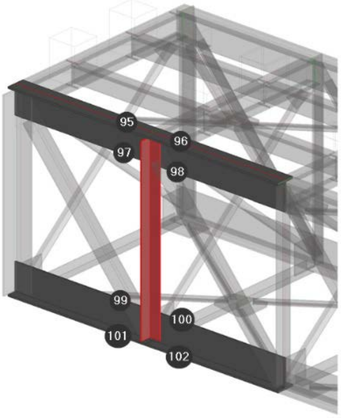

#	Unit	Ref	UD	D	$\Delta\varepsilon / \lvert\varepsilon_u\rvert$ [%]		$\Delta\varepsilon/\varepsilon_y$ [%]
95	[mε]	N,M	-0.0355 C	-0.1992 C	-460.82%	↑	-10.39%
96	[mε]	N,M	-0.1372 C	-0.2836 C	-106.73%	↑	-9.30%
97	[mε]	N,M	-0.0817 C	0.3057 T	474.28%	↻	24.60%
98	[mε]	N,M	-0.6558 C	0.0052 T	100.79%	↻	41.97%
99	[mε]	N,M	-0.2878 C	0.3100 T	207.70%	↻	37.96%
100	[mε]	N,M	0.1145 T	0.3378 T	194.96%	↑	14.18%
101	[mε]	N,M	-0.0196 C	-0.1886 C	-862.01%	↑	-10.73%
102	[mε]	N,M	0.0714 T	-0.0364 C	-150.93%	↻	-6.84%

Fig. 42 | Strain results for the main chords near joints and far from the centre of gravity of the monitored elements (this case close to the removed element), representing both axial and bending forces.

In summary, in a Pratt truss, the verticals and diagonals are the primary elements responsible for transferring shear forces. The failure of a vertical leads to **increased displacements and distortions** in the affected area. This forces the structure to redistribute the loads, **activating different ALPs**. The bridge undergoes **torsion**, causing an asymmetric redistribution of loads: elements diagonally opposite the damaged zone experience unloading, while those closer to the failure take more load. Furthermore, the vertical removal leads to **load transfers between trusses** and towards the nearest verticals and diagonals. Finally, the loss of the vertical results in a **local geometric reconfiguration** of the affected truss, forming a frame composed of diagonals and chords. In this zone, **Vierendeel-type behaviour** is observed, where the ALP involves **bending moments in the chords and diagonals**, which is characteristic of the failure scenario.

1.4.5 DS – Loss of horizontal lower bracings

Fig. 43 shows the definition of this damage scenario, which was reproduced in two levels of damage. In the first damage level, only an element of the lower bracing was removed (partial loss), causing asymmetry, while in the second level of damage, two elements were removed (total loss).

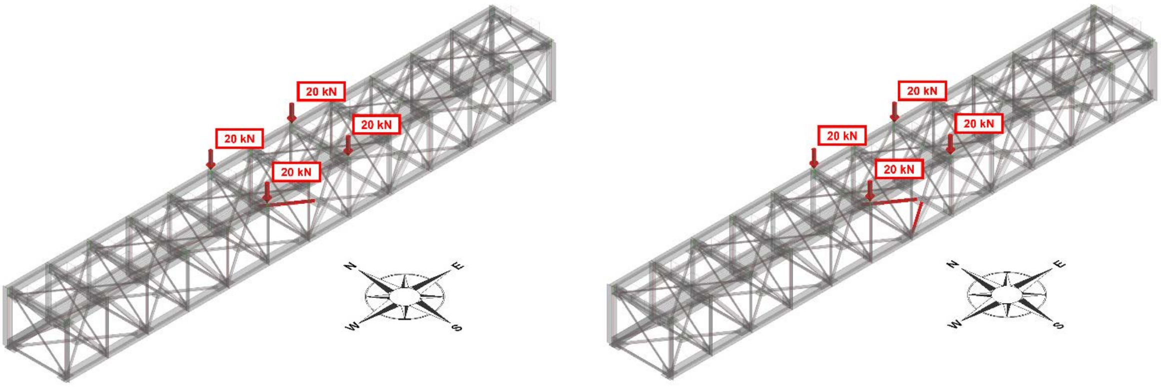

1st level of damage: Partial loss *2nd level of damage: Total loss*

Fig. 43 | Bridge, loads and damage scenario identified by the red colour elements for the first and second level of damage.

The results obtained for vertical displacements and distortions are shown in Fig. 44 and Fig. 45. Each figure includes the references and units for the sensor measurements: d for displacements (in mm) and θ for distortions (in degrees). Additionally, the absolute values recorded by each sensor in undamaged (UD) and damaged (D) conditions are presented, along with the processed results, as discussed at the beginning of Section 1.4. From the results, it can be concluded that the partial and total loss of the horizontal bracing system has no significant effect on the vertical displacements and distortions of the bridge.

- The partial and total loss of the horizontal bracing leads to a slight reduction in the torsional and vertical stiffness of the bridge, resulting in vertical displacements and distortions up to 2.01% greater than those in the undamaged condition.
- The partial loss of horizontal bracing introduces an asymmetry in the structural behaviour of the bridge, which can be observed through the resulting vertical displacements.

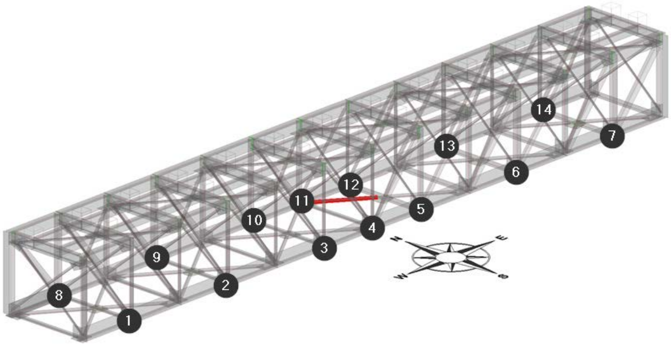

#	Unit	Ref	UD	D	Δd / $\lvert d_U \rvert$ [%]
1	[mm]	d	2.56	2.60	1.57%
2	[mm]	d	5.82	5.89	1.24%
3	[mm]	d	8.22	8.31	1.06%
4	[mm]	d	8.44	8.51	0.81%
5	[mm]	d	8.00	8.02	0.23%
6	[mm]	d	5.47	5.46	-0.22%
7	[mm]	d	2.22	2.22	0.06%

#	Unit	Ref	UD	D	Δd / $\lvert d_U \rvert$ [%]
8	[mm]	d	1.71	1.71	0.30%
9	[mm]	d	4.89	4.86	-0.73%
10	[mm]	d	7.40	7.40	0.09%
11	[mm]	d	7.91	7.90	-0.20%
12	[mm]	d	7.59	7.71	1.59%
13	[mm]	d	5.07	5.15	1.69%
14	[mm]	d	1.81	1.85	2.01%

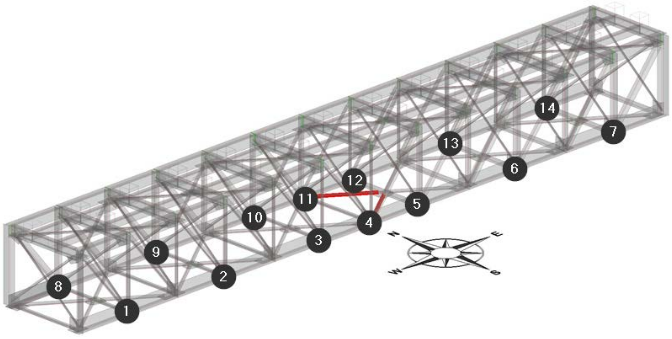

#	Unit	Ref	UD	D	Δd / $\lvert d_U \rvert$ [%]
1	[mm]	d	2.56	2.58	0.51%
2	[mm]	d	5.82	5.84	0.36%
3	[mm]	d	8.22	8.26	0.40%
4	[mm]	d	8.44	8.46	0.26%
5	[mm]	d	8.00	8.01	0.07%
6	[mm]	d	5.47	5.47	0.05%
7	[mm]	d	2.22	2.22	0.22%

#	Unit	Ref	UD	D	Δd / $\lvert d_U \rvert$ [%]
8	[mm]	d	1.71	1.74	1.84%
9	[mm]	d	4.89	4.93	0.69%
10	[mm]	d	7.40	7.44	0.57%
11	[mm]	d	7.91	7.95	0.51%
12	[mm]	d	7.59	7.73	1.78%
13	[mm]	d	5.07	5.13	1.30%
14	[mm]	d	1.81	1.86	2.36%

Fig. 44 | Vertical displacement results for the two levels of damage.

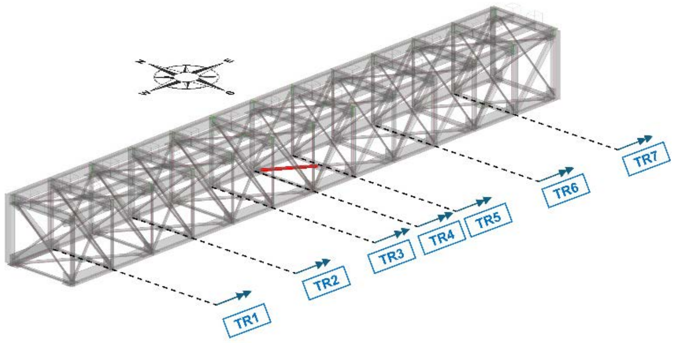

#	Unit	Ref	UD	D	$\Delta\theta / \lvert\theta_U\rvert$ [%]
TR1	[º]	θ	0.070	0.073	4.09%
TR2	[º]	θ	0.076	0.085	11.68%
TR3	[º]	θ	0.068	0.074	9.76%
TR4	[º]	θ	0.043	0.050	16.07%
TR5	[º]	θ	0.034	0.025	-24.93%
TR6	[º]	θ	0.033	0.025	-24.42%
TR7	[º]	θ	0.033	0.030	-8.68%

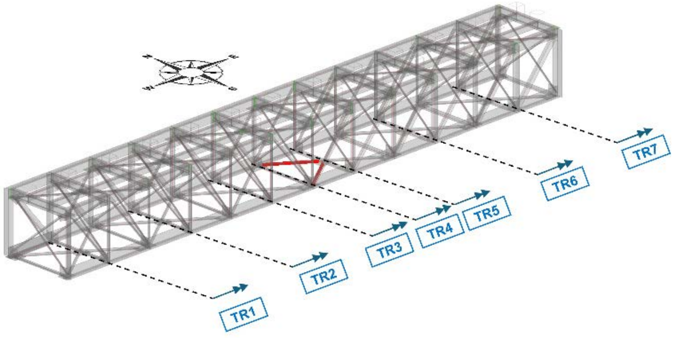

#	Unit	Ref	UD	D	$\Delta\theta / \lvert\theta_U\rvert$ [%]
TR1	[º]	θ	0.070	0.069	-2.14%
TR2	[º]	θ	0.076	0.075	-1.37%
TR3	[º]	θ	0.068	0.067	-1.10%
TR4	[º]	θ	0.043	0.042	-3.58%
TR5	[º]	θ	0.034	0.023	-31.52%
TR6	[º]	θ	0.033	0.028	-15.73%
TR7	[º]	θ	0.033	0.030	-9.41%

Fig. 45 | Distortion results for the two levels of damage.

The results of the measured strains are presented in Figures 46-51. Each figure details the monitored elements, including the unit of measurement for each sensor and whether the location is representative of axial forces (N) or both axial forces and moments (N, M). The absolute values recorded by each sensor are displayed for both undamaged (UD) and damaged (D) conditions, along with an indication of whether the values represent tension (T) or compression (C). The data were processed as described at the start of Section 1.4. This analysis enables the following conclusions to be drawn:

- *Horizontal bracings* (Fig. 46): The horizontal bracings in the undamaged zone (West), adjacent to those removed in the damage scenario, show no significant changes in their structural response and, consequently, do not contribute to the activation of the ALPs. In contrast, the bracings in the East section are significantly unloaded, even entirely, when the second level of damage occurs
- *Vertical bracings* (Fig. 47): For the first level of damage, the vertical bracing system plays a crucial role in compensating for the asymmetric failure of the structure. The loss of horizontal bracing leads to a degradation in the bridge's torsional stiffness, inducing localised distortions, which are mitigated by the vertical bracing. In the case of total loss (second level of damage), the vertical bracing becomes less important, as the failure is symmetrical.
- *Verticals* (Fig. 48): The partial and total loss of the lower horizontal bracing system does not cause a significant response in the verticals, preventing them from being considered important elements in the load redistribution. In none of the cases do the ratios $\Delta\varepsilon/\varepsilon_y$ exceed +/-1.00% of the yield strain, indicating that these elements are neither critical nor important in the activation of ALPs for this damage scenario.
- *Diagonals* (Fig. 49): Upon partial or total loss of the horizontal bracing system, the ratio $\Delta\varepsilon/\varepsilon_y$ does not fall below -1.00% or exceed 1.00%. Thus, the response of the outer diagonals to the damage scenario indicates that load redistribution and ALPs activation do not primarily occur through them. Consequently, the diagonals cannot be considered critical or important elements for this failure scenario.
- *Chords* (Fig. 50 & Fig. 51): The partial removal of the bracing system causes an asymmetric load distribution in the central chords. The chords located in the module affected by the partial loss of bracing (northeast) exhibit greater strains, while those in the neighbouring module

(northwest) experience unloading. In contrast, the chords further from the affected zone (southwest and southeast) show an increase in loading. The greatest strains are observed in the module where the bracing was removed, demonstrating the sensitivity of this element in load redistribution and ALPs activation in the local zone where the failure occurs. Total removal of the bracing system also results in an asymmetric load distribution. However, the $\Delta\varepsilon/\varepsilon_y$ relationship results suggest that, in this case, the ALPs pass through the lower chords on the east side, indicating a loss of torsional and vertical stiffness in the centre-east of the bridge, precisely where the failure occurs. This behaviour is evident in the axial forces in the chords (Fig. 52) and quantitatively aligns with the values representative of axial forces in combination with bending moments (Fig. 53). In other words, the redistribution of loads and the activation of the ALP depends on the development of tensile and compressive forces in the chords (axial forces) and not so much on the activation of greater or lesser bending moments in these elements.

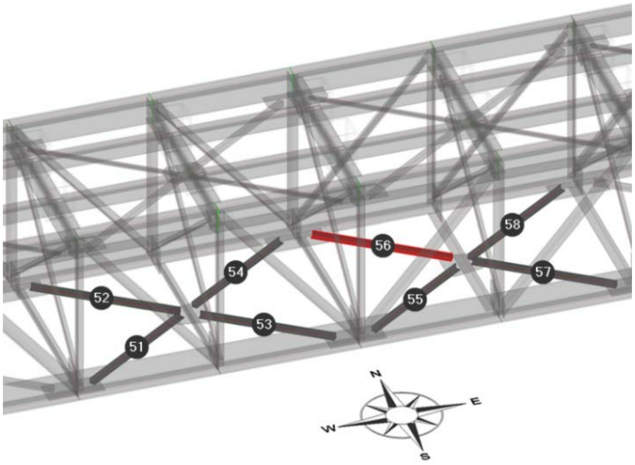

#	Unit	Ref	UD	D	Δε / |ε_U| [%]		$\Delta\varepsilon/\varepsilon_y$ [%]
51	[mε]	N	0.2348 T	0.2420 T	3.05%	↑	0.45%
52	[mε]	N	0.2618 T	0.2706 T	3.36%	↑	0.56%
53	[mε]	N	0.3243 T	0.3428 T	5.71%	↑	1.18%
54	[mε]	N	0.2240 T	0.2212 T	-1.24%	↓	-0.18%
55	[mε]	N	0.2115 T	0.1421 T	-32.80%	↓	-4.40%
56	[mε]	N	0.3419 T	0.0006 T	-99.83%	↓	-21.68%
57	[mε]	N	0.2637 T	0.0796 T	-69.82%	↓	-11.69%
58	[mε]	N	0.2140 T	0.0549 T	-74.34%	↓	-10.10%

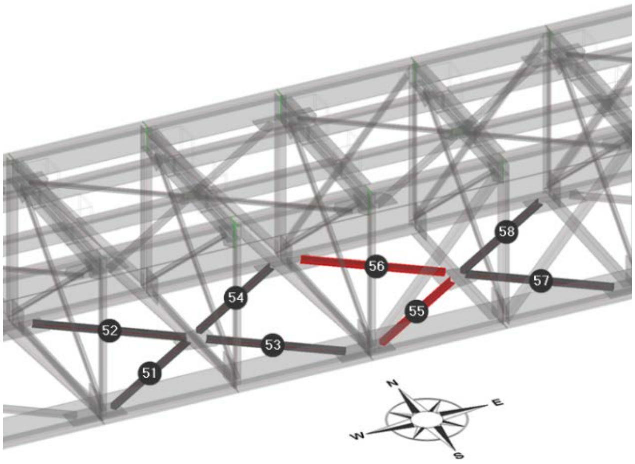

#	Unit	Ref	UD	D	Δε / |ε_U| [%]		$\Delta\varepsilon/\varepsilon_y$ [%]
51	[mε]	N	0.2348 T	0.2442 T	4.02%	↑	0.60%
52	[mε]	N	0.2618 T	0.2721 T	3.92%	↑	0.65%
53	[mε]	N	0.3243 T	0.3405 T	5.02%	↑	1.03%
54	[mε]	N	0.2240 T	0.2317 T	3.43%	↑	0.49%
55	[mε]	N	0.2115 T	-0.0084 C	-103.99%	↻	-13.97%
56	[mε]	N	0.3419 T	0.0001 T	-99.98%	↓	-21.71%
57	[mε]	N	0.2637 T	0.0115 T	-95.63%	↓	-16.01%
58	[mε]	N	0.2140 T	-0.0053 C	-102.47%	↻	-13.92%

Fig. 46 | Strain results for the horizontal lower bracing monitored elements.

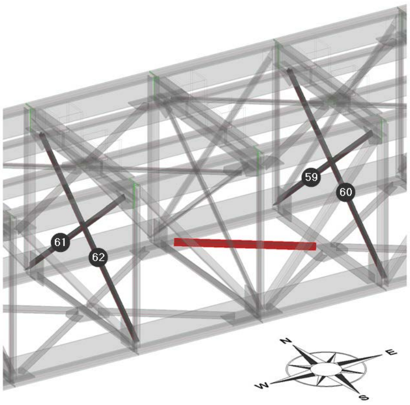

#	Unit	Ref	UD	D	$\Delta\varepsilon / \lvert\varepsilon_u\rvert$ [%]		$\Delta\varepsilon/\varepsilon_y$ [%]
59	[mε]	N	-0.0625 C	-0.1094 C	-75.03%	↑	-2.98%
60	[mε]	N	-0.1566 C	-0.0909 C	41.98%	↓	4.18%
61	[mε]	N	-0.1427 C	-0.1300 C	8.95%	↓	0.81%
62	[mε]	N	-0.0827 C	-0.0959 C	-15.94%	↑	-0.84%

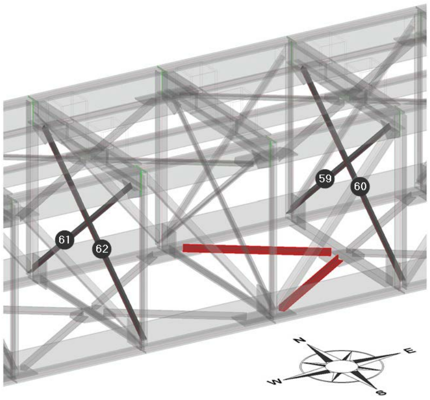

#	Unit	Ref	UD	D	$\Delta\varepsilon / \lvert\varepsilon_u\rvert$ [%]		$\Delta\varepsilon/\varepsilon_y$ [%]
59	[mε]	N	-0.0625 C	-0.0810 C	-29.51%	↑	-1.17%
60	[mε]	N	-0.1566 C	-0.1211 C	22.68%	↓	2.26%
61	[mε]	N	-0.1427 C	-0.1361 C	4.63%	↓	0.42%
62	[mε]	N	-0.0827 C	-0.0905 C	-9.44%	↑	-0.50%

Fig. 47 | Strain results for the vertical bracing monitored elements.

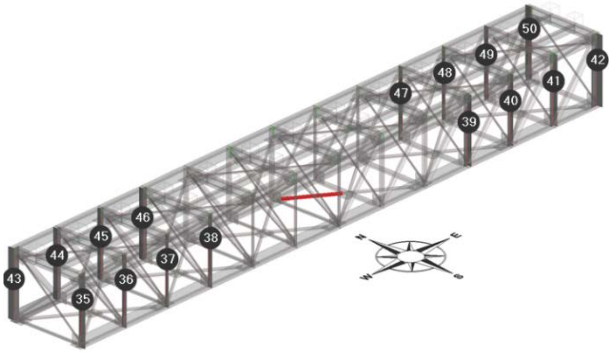

#	Unit	Ref	UD	D	$\Delta\varepsilon / \lvert\varepsilon_u\rvert$ [%]		$\Delta\varepsilon/\varepsilon_y$ [%]
35	[mε]	N,M	-0.0447 C	-0.0451 C	-0.95%	↑	-0.03%
36	[mε]	N,M	-0.1900 C	-0.1916 C	-0.83%	↑	-0.10%
37	[mε]	N,M	-0.3819 C	-0.3869 C	-1.29%	↑	-0.31%
38	[mε]	N,M	-0.2217 C	-0.2248 C	-1.38%	↑	-0.19%
39	[mε]	N,M	-0.2109 C	-0.2080 C	1.41%	↓	0.19%
40	[mε]	N,M	-0.4590 C	-0.4494 C	2.09%	↓	0.61%
41	[mε]	N,M	-0.2518 C	-0.2466 C	2.07%	↓	0.33%
42	[mε]	N,M	-0.0530 C	-0.0514 C	3.03%	↓	0.10%

#	Unit	Ref	UD	D	$\Delta\varepsilon / \lvert\varepsilon_u\rvert$ [%]		$\Delta\varepsilon/\varepsilon_y$ [%]
43	[mε]	N,M	-0.0559 C	-0.0558 C	0.21%	↓	0.01%
44	[mε]	N,M	-0.2765 C	-0.2706 C	2.11%	↓	0.37%
45	[mε]	N,M	-0.4416 C	-0.4364 C	1.17%	↓	0.33%
46	[mε]	N,M	-0.2011 C	-0.1996 C	0.78%	↓	0.10%
47	[mε]	N,M	-0.2274 C	-0.2335 C	-2.67%	↑	-0.39%
48	[mε]	N,M	-0.4863 C	-0.4958 C	-1.97%	↑	-0.61%
49	[mε]	N,M	-0.2273 C	-0.2320 C	-2.08%	↑	-0.30%
50	[mε]	N,M	-0.0655 C	-0.0673 C	-2.83%	↑	-0.12%

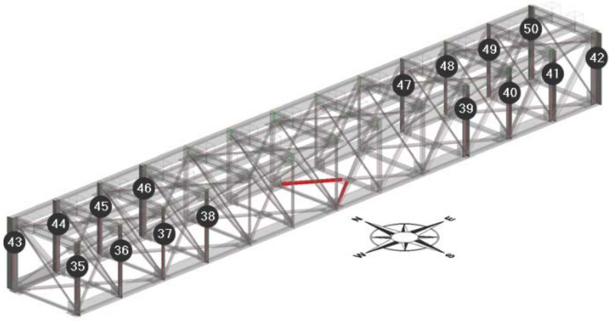

#	Unit	Ref	UD	D	$\Delta\varepsilon / \lvert\varepsilon_u\rvert$ [%]		$\Delta\varepsilon/\varepsilon_y$ [%]
35	[mε]	N,M	-0.0447 C	-0.0445 C	0.50%	↓	0.01%
36	[mε]	N,M	-0.1900 C	-0.1904 C	-0.18%	↑	-0.02%
37	[mε]	N,M	-0.3819 C	-0.3831 C	-0.30%	↑	-0.07%
38	[mε]	N,M	-0.2217 C	-0.2219 C	-0.09%	↑	-0.01%
39	[mε]	N,M	-0.2109 C	-0.2091 C	0.88%	↓	0.12%
40	[mε]	N,M	-0.4590 C	-0.4529 C	1.34%	↓	0.39%
41	[mε]	N,M	-0.2518 C	-0.2493 C	0.98%	↓	0.16%
42	[mε]	N,M	-0.0530 C	-0.0526 C	0.90%	↓	0.03%

#	Unit	Ref	UD	D	$\Delta\varepsilon / \lvert\varepsilon_u\rvert$ [%]		$\Delta\varepsilon/\varepsilon_y$ [%]
43	[mε]	N,M	-0.0559 C	-0.0566 C	-1.23%	↑	-0.04%
44	[mε]	N,M	-0.2765 C	-0.2729 C	1.30%	↓	0.23%
45	[mε]	N,M	-0.4416 C	-0.4401 C	0.33%	↓	0.09%
46	[mε]	N,M	-0.2011 C	-0.2012 C	-0.06%	↑	-0.01%
47	[mε]	N,M	-0.2274 C	-0.2315 C	-1.81%	↑	-0.26%
48	[mε]	N,M	-0.4863 C	-0.4916 C	-1.09%	↑	-0.34%
49	[mε]	N,M	-0.2273 C	-0.2297 C	-1.07%	↑	-0.15%
50	[mε]	N,M	-0.0655 C	-0.0671 C	-2.40%	↑	-0.10%

Fig. 48 | Strain results for the vertical monitored elements.

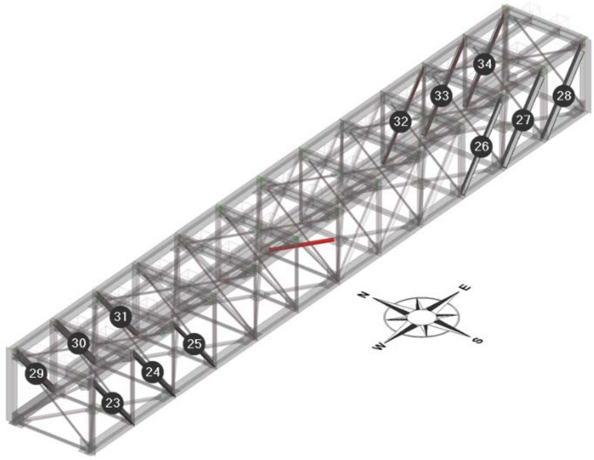

#	Unit	Ref	UD	D	$\Delta\varepsilon / \lvert\varepsilon_u\rvert$ [%]		$\Delta\varepsilon/\varepsilon_y$ [%]
23	[mε]	N	0.4363 T	0.4415 T	1.19%	↑	0.33%
24	[mε]	N	0.4936 T	0.4989 T	1.07%	↑	0.34%
25	[mε]	N	0.6916 T	0.6982 T	0.96%	↑	0.42%
26	[mε]	N	0.6841 T	0.6727 T	-1.68%	↓	-0.73%
27	[mε]	N	0.4704 T	0.4633 T	-1.51%	↓	-0.45%
28	[mε]	N	0.4289 T	0.4229 T	-1.41%	↓	-0.38%

#	Unit	Ref	UD	D	$\Delta\varepsilon / \lvert\varepsilon_u\rvert$ [%]		$\Delta\varepsilon/\varepsilon_y$ [%]
29	[mε]	N	0.4422 T	0.4378 T	-1.00%	↓	-0.28%
30	[mε]	N	0.4649 T	0.4609 T	-0.86%	↓	-0.26%
31	[mε]	N	0.6571 T	0.6493 T	-1.18%	↓	-0.49%
32	[mε]	N	0.6555 T	0.6658 T	1.57%	↑	0.65%
33	[mε]	N	0.5084 T	0.5173 T	1.75%	↑	0.57%
34	[mε]	N	0.3980 T	0.4049 T	1.76%	↑	0.44%

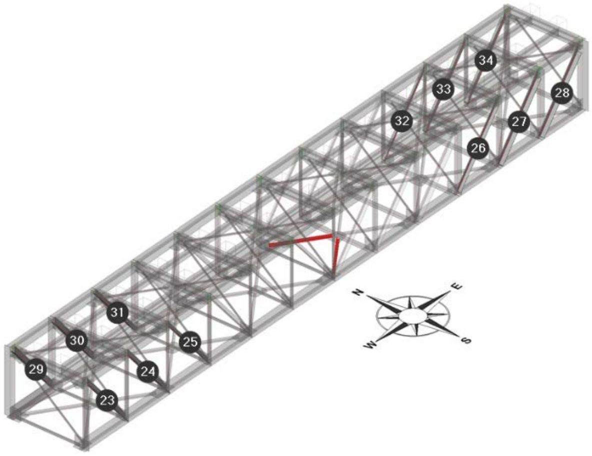

#	Unit	Ref	UD	D	$\Delta\varepsilon / \lvert\varepsilon_u\rvert$ [%]		$\Delta\varepsilon/\varepsilon_y$ [%]
23	[mε]	N	0.4363 T	0.4375 T	0.27%	↑	0.08%
24	[mε]	N	0.4936 T	0.4953 T	0.35%	↑	0.11%
25	[mε]	N	0.6916 T	0.6941 T	0.37%	↑	0.16%
26	[mε]	N	0.6841 T	0.6771 T	-1.03%	↓	-0.45%
27	[mε]	N	0.4704 T	0.4663 T	-0.89%	↓	-0.27%
28	[mε]	N	0.4289 T	0.4249 T	-0.94%	↓	-0.26%

#	Unit	Ref	UD	D	$\Delta\varepsilon / \lvert\varepsilon_u\rvert$ [%]		$\Delta\varepsilon/\varepsilon_y$ [%]
29	[mε]	N	0.4422 T	0.4403 T	-0.42%	↓	-0.12%
30	[mε]	N	0.4649 T	0.4631 T	-0.39%	↓	-0.11%
31	[mε]	N	0.6571 T	0.6539 T	-0.50%	↓	-0.21%
32	[mε]	N	0.6555 T	0.6620 T	0.98%	↑	0.41%
33	[mε]	N	0.5084 T	0.5139 T	1.09%	↑	0.35%
34	[mε]	N	0.3980 T	0.4009 T	0.73%	↑	0.18%

Fig. 49 | Strain results for the diagonal monitored elements.

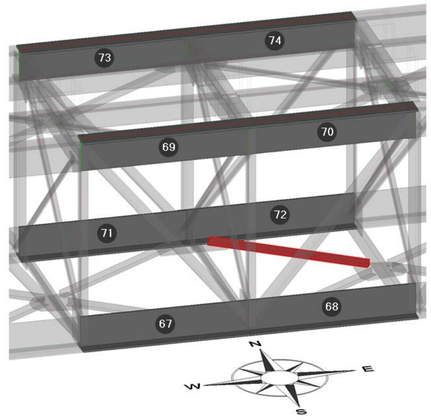

#	Measure	Ref	UD	D	$\Delta\varepsilon / \lvert\varepsilon_u\rvert$ [%]		$\Delta\varepsilon/\varepsilon_y$ [%]
67	[mε]	N	0.4083 T	0.4118 T	0.84%	↑	0.22%
68	[mε]	N	0.4028 T	0.4141 T	2.82%	↑	0.72%
69	[mε]	N	-0.3044 C	-0.3117 C	-2.37%	↑	-0.46%
70	[mε]	N	-0.3483 C	-0.3461 C	0.63%	↓	0.14%

#	Unit	Ref	UD	D	$\Delta\varepsilon / \lvert\varepsilon_u\rvert$ [%]		$\Delta\varepsilon/\varepsilon_y$ [%]
71	[mε]	N	0.3891 T	0.3832 T	-1.53%	↓	-0.38%
72	[mε]	N	0.4012 T	0.4272 T	6.47%	↑	1.65%
73	[mε]	N	-0.3229 C	-0.3173 C	1.73%	↓	0.35%
74	[mε]	N	-0.3086 C	-0.3103 C	-0.57%	↑	-0.11%

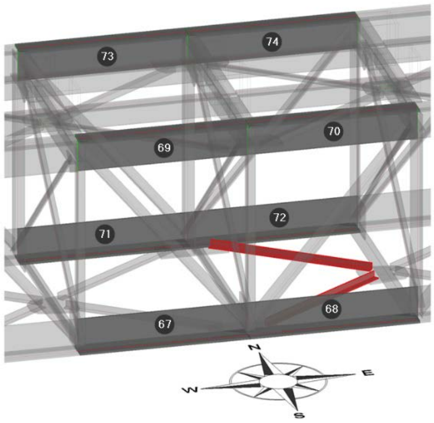

#	Measure	Ref	UD	D	$\Delta\varepsilon / \lvert\varepsilon_u\rvert$ [%]		$\Delta\varepsilon/\varepsilon_y$ [%]
67	[mε]	N	0.4083 T	0.4095 T	0.28%	↑	0.07%
68	[mε]	N	0.4028 T	0.4240 T	5.27%	↑	1.35%
69	[mε]	N	-0.3044 C	-0.3082 C	-1.24%	↑	-0.24%
70	[mε]	N	-0.3483 C	-0.3447 C	1.03%	↓	0.23%

#	Unit	Ref	UD	D	$\Delta\varepsilon / \lvert\varepsilon_u\rvert$ [%]		$\Delta\varepsilon/\varepsilon_y$ [%]
71	[mε]	N	0.3891 T	0.3858 T	-0.85%	↓	-0.21%
72	[mε]	N	0.4012 T	0.4294 T	7.04%	↑	1.79%
73	[mε]	N	-0.3229 C	-0.3203 C	0.80%	↓	0.16%
74	[mε]	N	-0.3086 C	-0.3093 C	-0.22%	↑	-0.04%

Fig. 50 | Strain results for the main chords in the centre of the gravity and centre of the length of the monitored elements, only representatives of the axial forces.

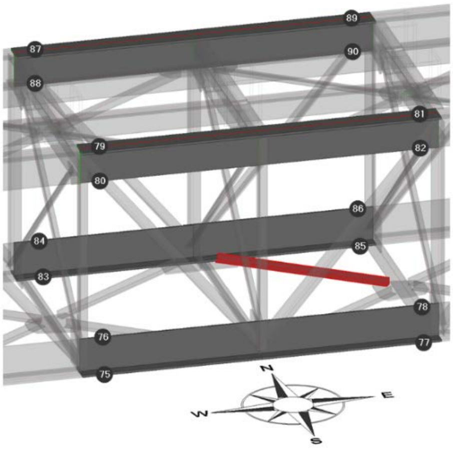

#	Unit	Ref	UD	D	Δε / |ε_u| [%]		Δε/ε_y [%]
75	[mε]	N,M	0.5206 T	0.5283 T	1.47%	↑	0.49%
76	[mε]	N,M	0.1913 T	0.1934 T	1.11%	↑	0.14%
77	[mε]	N,M	0.4793 T	0.4816 T	0.47%	↑	0.14%
78	[mε]	N,M	0.2437 T	0.2666 T	9.43%	↑	1.46%
79	[mε]	N,M	-0.4722 C	-0.4804 C	-1.74%	↑	-0.52%
80	[mε]	N,M	-0.2133 C	-0.2044 C	4.20%	↓	0.57%
81	[mε]	N,M	-0.3760 C	-0.3749 C	0.29%	↓	0.07%
82	[mε]	N,M	-0.4073 C	-0.4143 C	-1.71%	↑	-0.44%

#	Unit	Ref	UD	D	Δε / |ε_u| [%]		Δε/ε_y [%]
83	[mε]	N,M	0.4765 T	0.4668 T	-2.03%	↓	-0.61%
84	[mε]	N,M	0.2751 T	0.2719 T	-1.16%	↓	-0.20%
85	[mε]	N,M	0.5067 T	0.5359 T	5.77%	↑	1.86%
86	[mε]	N,M	0.1730 T	0.1840 T	6.32%	↑	0.69%
87	[mε]	N,M	-0.4784 C	-0.4709 C	1.56%	↓	0.47%
88	[mε]	N,M	-0.1450 C	-0.1431 C	1.33%	↓	0.12%
89	[mε]	N,M	-0.4068 C	-0.4148 C	-1.98%	↑	-0.51%
90	[mε]	N,M	-0.1842 C	-0.1628 C	11.63%	↓	1.36%

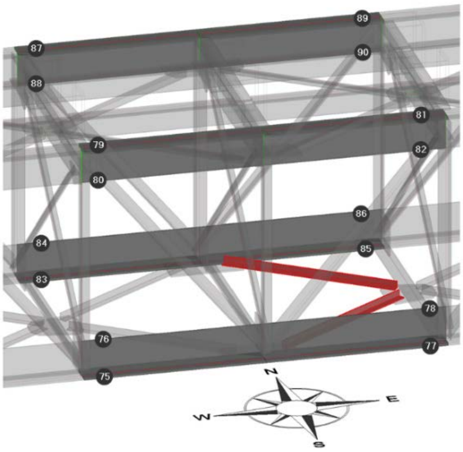

#	Unit	Ref	UD	D	Δε / |ε_u| [%]		Δε/ε_y [%]
75	[mε]	N,M	0.5206 T	0.5216 T	0.18%	↑	0.06%
76	[mε]	N,M	0.1913 T	0.1927 T	0.71%	↑	0.09%
77	[mε]	N,M	0.4793 T	0.4940 T	3.06%	↑	0.93%
78	[mε]	N,M	0.2437 T	0.2676 T	9.83%	↑	1.52%
79	[mε]	N,M	-0.4722 C	-0.4742 C	-0.42%	↑	-0.13%
80	[mε]	N,M	-0.2133 C	-0.2031 C	4.76%	↓	0.65%
81	[mε]	N,M	-0.3760 C	-0.3780 C	-0.51%	↑	-0.12%
82	[mε]	N,M	-0.4073 C	-0.4030 C	1.07%	↓	0.28%

#	Unit	Ref	UD	D	Δε / |ε_u| [%]		Δε/ε_y [%]
83	[mε]	N,M	0.4765 T	0.4716 T	-1.02%	↓	-0.31%
84	[mε]	N,M	0.2751 T	0.2733 T	-0.65%	↓	-0.11%
85	[mε]	N,M	0.5067 T	0.5361 T	5.81%	↑	1.87%
86	[mε]	N,M	0.1730 T	0.1909 T	10.31%	↑	1.13%
87	[mε]	N,M	-0.4784 C	-0.4748 C	0.74%	↓	0.22%
88	[mε]	N,M	-0.1450 C	-0.1459 C	-0.62%	↑	-0.06%
89	[mε]	N,M	-0.4068 C	-0.4117 C	-1.21%	↑	-0.31%
90	[mε]	N,M	-0.1842 C	-0.1737 C	5.70%	↓	0.67%

Fig. 51 | Strain results for the main chords near joints and far from the centre of gravity of the monitored elements, representing both axial and bending forces.

In summary, in a Pratt truss, the horizontal bracing system is one of the main elements contributing to the torsional and lateral stiffness of the bridge. However, under the effect of vertical loads, it mainly contributes to **global (positive) bending** by **absorbing some of the stresses at the lower chord level**, thus allowing these chords to assume lower loads.

In the event of a **partial loss**, an asymmetry in the geometric configuration of the bridge occurs, its torsional and vertical stiffness weakens, and **load redistribution passes through the vertical bracing and chords**. In both cases, **only increases in axial forces** were detected. **After total loss**, **load redistribution mainly occurs through the lower chords on the affected (east) side** as the lost horizontal bracing elements no longer participate in the global positive bending response.

1.4.6 DS – Loss of vertical bracings

Fig. 52 shows the definition of this damage scenario, which was reproduced in two levels of damage. In the first damage level, only an element of the vertical bracing was removed (partial loss), while in the second level of damage two elements were removed (total loss).

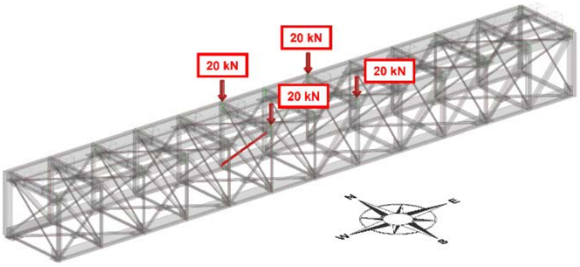

1st level of damage: Partial loss

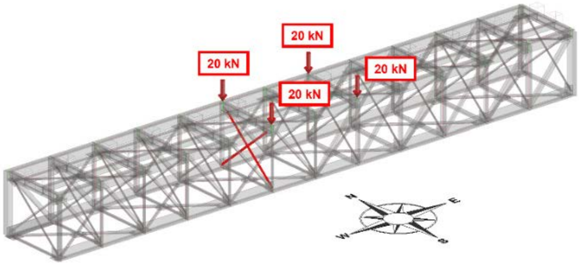

2nd level of damage: Total loss

Fig. 52 | Bridge, loads and damage scenario identified by the red colour elements for the first and second level of damage.

The results obtained for vertical displacements and distortions are shown in Fig. 53 and Fig. 54. Each figure includes the references and units for the sensor measurements: d for displacements (in mm) and θ for distortions (in degrees). Additionally, the absolute values recorded by each sensor in undamaged (UD) and damaged (D) conditions are presented, along with the processed results, as discussed at the beginning of Section 1.4. From the results, it can be concluded that the partial and total loss of the vertical bracing system has no significant effect on the vertical displacements and distortions of the bridge.

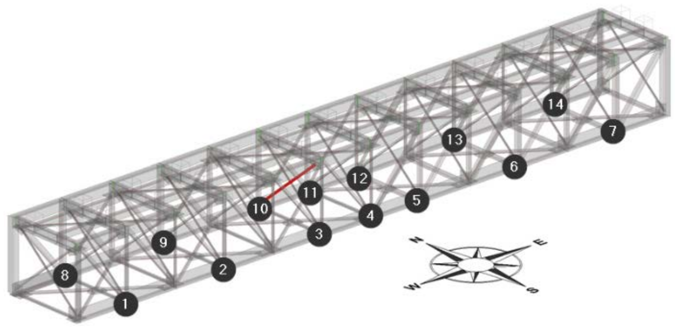

#	Unit	Ref	UD	D	$\Delta d / \lvert d_U \rvert$ [%]
1	[mm]	d	2.50	2.49	-0.13%
2	[mm]	d	5.76	5.75	-0.14%
4	[mm]	d	8.39	8.39	0.00%
5	[mm]	d	7.96	7.96	0.02%
6	[mm]	d	5.31	5.22	-1.65%
7	[mm]	d	2.21	2.21	-0.12%

#	Unit	Ref	UD	D	$\Delta d / \lvert d_U \rvert$ [%]
8	[mm]	d	1.81	1.83	0.74%
9	[mm]	d	4.95	4.96	0.17%
10	[mm]	d	7.58	7.57	-0.14%
11	[mm]	d	7.99	8.01	0.31%
12	[mm]	d	7.68	7.73	0.66%
13	[mm]	d	5.13	5.15	0.30%
14	[mm]	d	1.84	1.85	0.42%

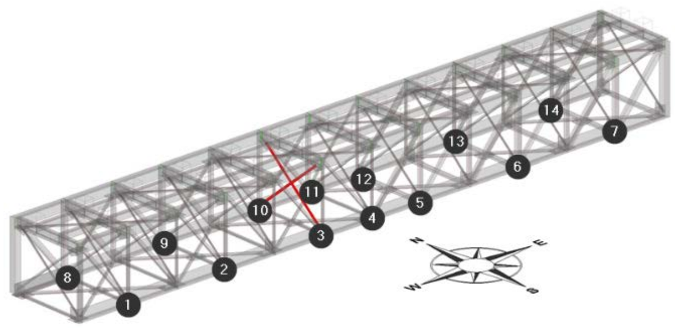

#	Unit	Ref	UD	D	$\Delta d / \lvert d_U \rvert$ [%]
1	[mm]	d	2.50	2.49	-0.33%
2	[mm]	d	5.76	5.75	-0.10%
4	[mm]	d	8.39	8.39	0.00%
5	[mm]	d	7.96	7.96	-0.02%
6	[mm]	d	5.31	5.15	-2.97%
7	[mm]	d	2.21	2.21	0.04%

#	Unit	Ref	UD	D	$\Delta d / \lvert d_U \rvert$ [%]
8	[mm]	d	1.81	1.83	0.70%
9	[mm]	d	4.95	4.95	0.01%
10	[mm]	d	7.58	7.54	-0.60%
11	[mm]	d	7.99	8.01	0.24%
12	[mm]	d	7.68	7.73	0.68%
13	[mm]	d	5.13	5.14	0.24%
14	[mm]	d	1.84	1.84	-0.12%

Fig. 53 | Vertical displacement results for the two levels of damage.

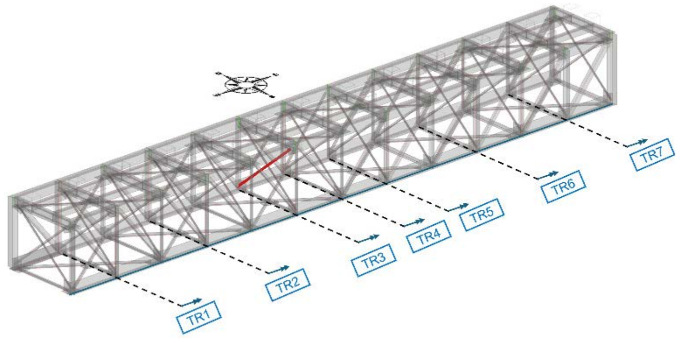

#	Unit	Ref	UD	D	$\Delta\theta / \lvert\theta_U\rvert$ [%]
TR1	[º]	θ	0.056	0.054	-2.44%
TR2	[º]	θ	0.066	0.065	-2.02%
TR4	[º]	θ	0.033	0.031	-6.06%
TR5	[º]	θ	0.023	0.019	-17.25%
TR6	[º]	θ	0.015	0.006	-58.03%
TR7	[º]	θ	0.030	0.029	-2.78%

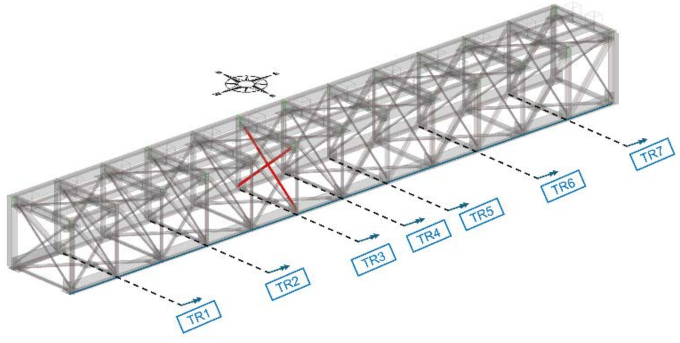

#	Unit	Ref	UD	D	$\Delta\theta / \lvert\theta_U\rvert$ [%]
TR1	[º]	θ	0.056	0.054	-3.07%
TR2	[º]	θ	0.066	0.066	-0.78%
TR4	[º]	θ	0.033	0.031	-4.85%
TR5	[º]	θ	0.023	0.019	-18.96%
TR6	[º]	θ	0.015	0.001	-95.67%
TR7	[º]	θ	0.030	0.031	0.88%

Fig. 54 | Distortion results for the two levels of damage.

The results of the measured strains are presented in Figures 55-60. Each figure details the monitored elements, including the unit of measurement for each sensor and whether the location is representative of axial forces (N) or both axial forces and moments (N, M). The absolute values recorded by each sensor are displayed for both undamaged (UD) and damaged (D) conditions, along with an indication of whether the values represent tension (T) or compression (C). The data were processed as described at the start of Section 1.4. This analysis enables the following conclusions to be drawn:

- *Horizontal bracings* (Fig. 55): The lower horizontal bracing system contributes slightly to load redistribution and ALPs activation through increases in its axial load, helping to control the minor torsions generated by the loss of the vertical bracing. However, the ratios $\Delta\varepsilon/\varepsilon_y$ barely exceed 1.40%, indicating the low importance of these elements in load redistribution and ALPs activation.
- *Vertical bracings* (Fig. 56): At a global level, the vertical bracing has no significant influence on load redistribution or ALPs activation in the event of partial or total loss of one of its neighbouring elements. Ratios $\Delta\varepsilon/\varepsilon_y$ below 0.17% indicate that this system is not critical for load redistribution, and its response does not reflect ALPs activation. However, in the case of partial loss, the response within the same vertical bracing system allows for the identification of failure and load redistribution, as indicated by a ratio $\Delta\varepsilon/\varepsilon_y$ of 3.50%.
- *Verticals* (Fig. 57): The small $\Delta\varepsilon/\varepsilon_y$ ratios obtained for the external verticals indicate their limited contribution to load redistribution in the event of partial or total loss of the vertical bracing. Noticeable strain measurements are observed only at a local level, in the vicinity of the damage, but these values remain low.
- *Diagonals* (Fig. 58): Conclusions like those drawn for the verticals can be applied to diagonals.
- *Chords* (Fig. 59 & Fig. 60): The behaviour of the central chords follows a diagonal loading and unloading pattern, closely resembling that observed in the horizontal bracing. This may be linked to the activation of the structure's torsional response. However, the ratios $\Delta\varepsilon/\varepsilon_y$ are negligible, confirming that these elements should not be considered significant in load redistribution. Similarly, it is concluded that load redistribution and ALP activation do not involve the occurrence of bending moments at the ends of the central chords.

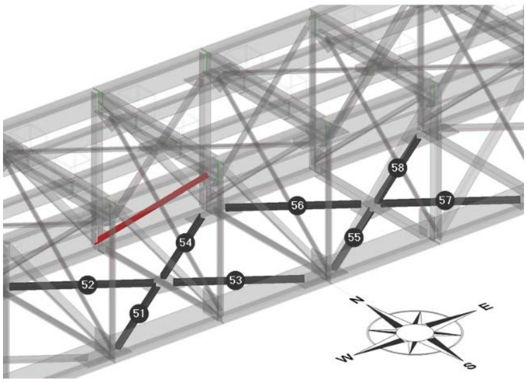

#	Unit	Ref	UD	D	$\Delta\varepsilon / \lvert\varepsilon_U\rvert$ [%]		$\Delta\varepsilon/\varepsilon_y$ [%]
51	[mε]	N	0.2364 T	0.2208 T	-6.58%	↓	-0.99%
52	[mε]	N	0.2595 T	0.2758 T	6.28%	↑	1.04%
53	[mε]	N	0.3188 T	0.2964 T	-7.01%	↓	-1.42%
54	[mε]	N	0.2288 T	0.2435 T	6.39%	↑	0.93%
55	[mε]	N	0.2442 T	0.2503 T	2.50%	↑	0.39%
56	[mε]	N	0.3149 T	0.3073 T	-2.41%	↓	-0.48%
57	[mε]	N	0.2519 T	0.2505 T	-0.56%	↓	-0.09%
58	[mε]	N	0.2198 T	0.2194 T	-0.18%	↓	-0.03%

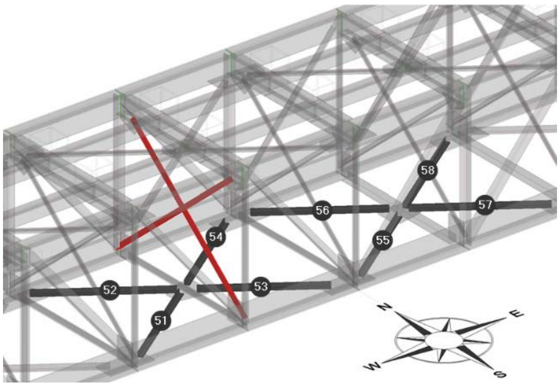

#	Unit	Ref	UD	D	$\Delta\varepsilon / \lvert\varepsilon_U\rvert$ [%]		$\Delta\varepsilon/\varepsilon_y$ [%]
51	[mε]	N	0.2364 T	0.2267 T	-4.10%	↓	-0.61%
52	[mε]	N	0.2595 T	0.2700 T	4.03%	↑	0.66%
53	[mε]	N	0.3188 T	0.3021 T	-5.23%	↓	-1.06%
54	[mε]	N	0.2288 T	0.2355 T	2.89%	↑	0.42%
55	[mε]	N	0.2442 T	0.2492 T	2.05%	↑	0.32%
56	[mε]	N	0.3149 T	0.3089 T	-1.92%	↓	-0.38%
57	[mε]	N	0.2519 T	0.2500 T	-0.76%	↓	-0.12%
58	[mε]	N	0.2198 T	0.2206 T	0.37%	↑	0.05%

Fig. 55 | Strain results for the horizontal lower bracing monitored elements.

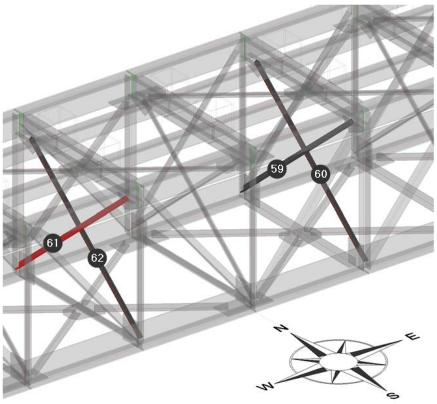

#	Unit	Ref	UD	D	Δε / |ε_U| [%]		Δε/ε_y [%]
59	[mε]	N	-0.0622 C	-0.0639 C	-2.81%	↑	-0.11%
60	[mε]	N	-0.1528 C	-0.1502 C	1.70%	↓	0.17%
61	[mε]	N	-0.1391 C	-0.0003 C	99.80%	↓	8.82%
62	[mε]	N	-0.0854 C	-0.0302 C	64.61%	↓	3.50%

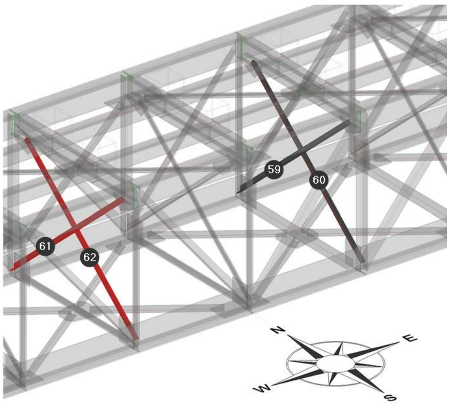

#	Unit	Ref	UD	D	Δε / |ε_U| [%]		Δε/ε_y [%]
59	[mε]	N	-0.0622 C	-0.0631 C	-1.48%	↑	-0.06%
60	[mε]	N	-0.1528 C	-0.1521 C	0.47%	↓	0.05%
61	[mε]	N	-0.1391 C	-0.0002 C	99.85%	↓	8.82%
62	[mε]	N	-0.0854 C	-0.0004 C	99.58%	↓	5.40%

Fig. 56 | Strain results for the vertical bracing monitored elements.

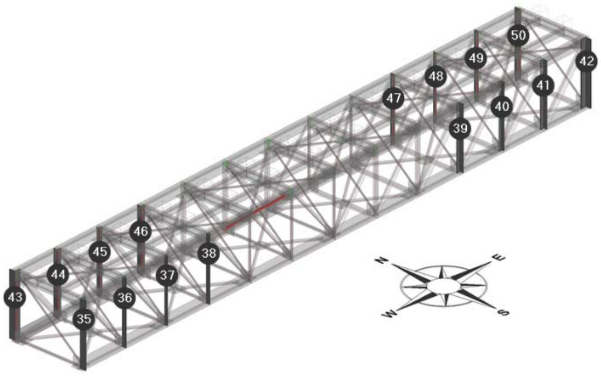

#	Unit	Ref	UD	D	Δε / |ε_U| [%]		Δε/ε_y [%]
35	[mε]	N,M	-0.0445 C	-0.0452 C	-1.50%	↑	-0.04%
36	[mε]	N,M	-0.1888 C	-0.1897 C	-0.47%	↑	-0.06%
37	[mε]	N,M	-0.3807 C	-0.3812 C	-0.14%	↑	-0.03%
38	[mε]	N,M	-0.2212 C	-0.2215 C	-0.12%	↑	-0.02%
39	[mε]	N,M	-0.2094 C	-0.2083 C	0.52%	↓	0.07%
40	[mε]	N,M	-0.4553 C	-0.4546 C	0.15%	↓	0.04%
41	[mε]	N,M	-0.2489 C	-0.2491 C	-0.07%	↑	-0.01%
42	[mε]	N,M	-0.0513 C	-0.0516 C	-0.48%	↑	-0.02%

#	Unit	Ref	UD	D	Δε / |ε_U| [%]		Δε/ε_y [%]
43	[mε]	N,M	-0.0562 C	-0.0564 C	-0.43%	↑	-0.02%
44	[mε]	N,M	-0.2747 C	-0.2739 C	0.27%	↓	0.05%
45	[mε]	N,M	-0.4415 C	-0.4436 C	-0.47%	↑	-0.13%
46	[mε]	N,M	-0.2016 C	-0.2027 C	-0.54%	↑	-0.07%
47	[mε]	N,M	-0.2297 C	-0.2305 C	-0.34%	↑	-0.05%
48	[mε]	N,M	-0.4920 C	-0.4929 C	-0.20%	↑	-0.06%
49	[mε]	N,M	-0.2299 C	-0.2298 C	0.07%	↓	0.01%
50	[mε]	N,M	-0.0675 C	-0.0683 C	-1.22%	↑	-0.05%

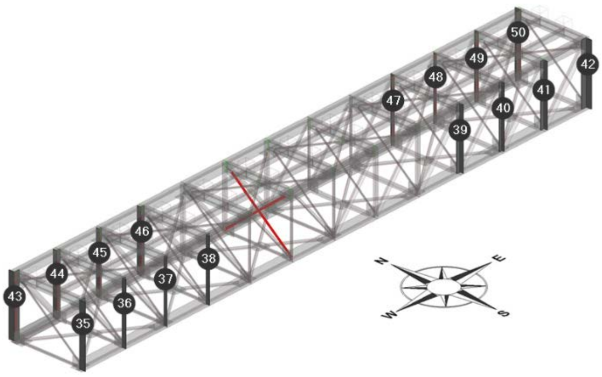

#	Unit	Ref	UD	D	Δε / |ε_U| [%]		Δε/ε_y [%]
35	[mε]	N,M	-0.0445 C	-0.0456 C	-2.56%	↑	-0.07%
36	[mε]	N,M	-0.1888 C	-0.1896 C	-0.44%	↑	-0.05%
37	[mε]	N,M	-0.3807 C	-0.3809 C	-0.06%	↑	-0.01%
38	[mε]	N,M	-0.2212 C	-0.2217 C	-0.23%	↑	-0.03%
39	[mε]	N,M	-0.2094 C	-0.2091 C	0.14%	↓	0.02%
40	[mε]	N,M	-0.4553 C	-0.4539 C	0.30%	↓	0.09%
41	[mε]	N,M	-0.2489 C	-0.2491 C	-0.09%	↑	-0.01%
42	[mε]	N,M	-0.0513 C	-0.0521 C	-1.59%	↑	-0.05%

#	Unit	Ref	UD	D	Δε / |ε_U| [%]		Δε/ε_y [%]
43	[mε]	N,M	-0.0562 C	-0.0571 C	-1.61%	↑	-0.06%
44	[mε]	N,M	-0.2747 C	-0.2743 C	0.16%	↓	0.03%
45	[mε]	N,M	-0.4415 C	-0.4446 C	-0.70%	↑	-0.20%
46	[mε]	N,M	-0.2016 C	-0.2025 C	-0.45%	↑	-0.06%
47	[mε]	N,M	-0.2297 C	-0.2317 C	-0.87%	↑	-0.13%
48	[mε]	N,M	-0.4920 C	-0.4949 C	-0.59%	↑	-0.18%
49	[mε]	N,M	-0.2299 C	-0.2313 C	-0.58%	↑	-0.08%
50	[mε]	N,M	-0.0675 C	-0.0685 C	-1.45%	↑	-0.06%

Fig. 57 | Strain results for the vertical monitored elements.

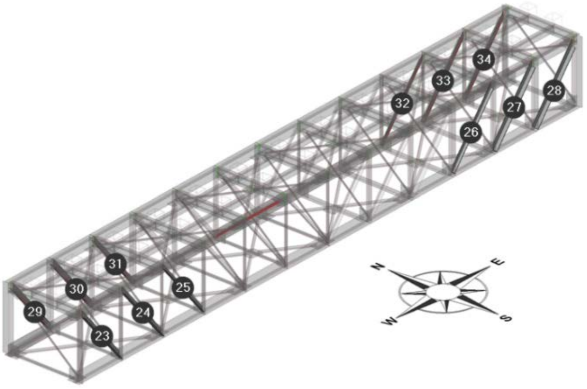

#	Unit	Ref	UD	D	$\Delta\varepsilon / \|\varepsilon_u\|$ [%]		$\Delta\varepsilon/\varepsilon_y$ [%]
23	[mε]	N	0.4374 T	0.4351 T	-0.52%	↓	-0.15%
24	[mε]	N	0.4941 T	0.4926 T	-0.29%	↓	-0.09%
25	[mε]	N	0.6953 T	0.6902 T	-0.73%	↓	-0.32%
26	[mε]	N	0.6802 T	0.6780 T	-0.33%	↓	-0.14%
27	[mε]	N	0.4679 T	0.4661 T	-0.38%	↓	-0.11%
28	[mε]	N	0.4266 T	0.4251 T	-0.34%	↓	-0.09%

#	Unit	Ref	UD	D	$\Delta\varepsilon / \|\varepsilon_u\|$ [%]		$\Delta\varepsilon/\varepsilon_y$ [%]
29	[mε]	N	0.4419 T	0.4425 T	0.14%	↑	0.04%
30	[mε]	N	0.4668 T	0.4671 T	0.06%	↑	0.02%
31	[mε]	N	0.6592 T	0.6591 T	-0.02%	↓	-0.01%
32	[mε]	N	0.6610 T	0.6628 T	0.27%	↑	0.11%
33	[mε]	N	0.5136 T	0.5152 T	0.32%	↑	0.10%
34	[mε]	N	0.4019 T	0.4018 T	-0.03%	↓	-0.01%

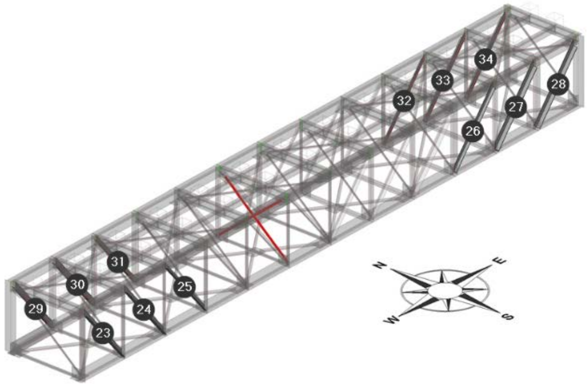

#	Unit	Ref	UD	D	$\Delta\varepsilon / \|\varepsilon_u\|$ [%]		$\Delta\varepsilon/\varepsilon_y$ [%]
23	[mε]	N	0.4374 T	0.4348 T	-0.59%	↓	-0.16%
24	[mε]	N	0.4941 T	0.4929 T	-0.25%	↓	-0.08%
25	[mε]	N	0.6953 T	0.6888 T	-0.93%	↓	-0.41%
26	[mε]	N	0.6802 T	0.6776 T	-0.39%	↓	-0.17%
27	[mε]	N	0.4679 T	0.4655 T	-0.52%	↓	-0.15%
28	[mε]	N	0.4266 T	0.4247 T	-0.44%	↓	-0.12%

#	Unit	Ref	UD	D	$\Delta\varepsilon / \|\varepsilon_u\|$ [%]		$\Delta\varepsilon/\varepsilon_y$ [%]
29	[mε]	N	0.4419 T	0.4425 T	0.13%	↑	0.04%
30	[mε]	N	0.4668 T	0.4660 T	-0.19%	↓	-0.06%
31	[mε]	N	0.6592 T	0.6579 T	-0.20%	↓	-0.08%
32	[mε]	N	0.6610 T	0.6620 T	0.15%	↑	0.06%
33	[mε]	N	0.5136 T	0.5147 T	0.22%	↑	0.07%
34	[mε]	N	0.4019 T	0.4020 T	0.02%	↑	0.00%

Fig. 58 | Strain results for the diagonal monitored elements.

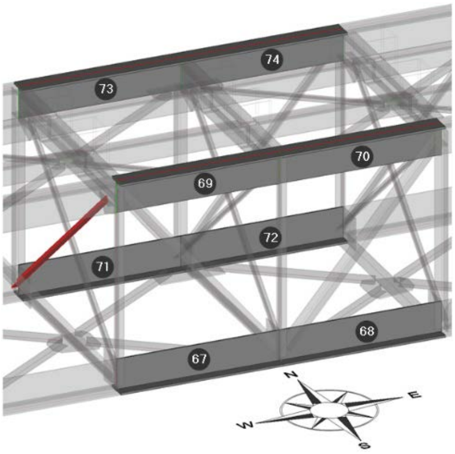

#	Measure	Ref	UD	D	$\Delta\varepsilon / \|\varepsilon_u\|$ [%]		$\Delta\varepsilon/\varepsilon_y$ [%]
67	[mε]	N	0.4084 T	0.4114 T	0.73%	↑	0.19%
68	[mε]	N	0.4016 T	0.4003 T	-0.33%	↓	-0.08%
69	[mε]	N	-0.3059 C	-0.3078 C	-0.63%	↑	-0.12%
70	[mε]	N	-0.3490 C	-0.3465 C	0.71%	↓	0.16%

#	Unit	Ref	UD	D	$\Delta\varepsilon / \|\varepsilon_u\|$ [%]		$\Delta\varepsilon/\varepsilon_y$ [%]
71	[mε]	N	0.3896 T	0.3861 T	-0.90%	↓	-0.22%
72	[mε]	N	0.4013 T	0.4016 T	0.07%	↑	0.02%
73	[mε]	N	-0.3229 C	-0.3211 C	0.54%	↓	0.11%
74	[mε]	N	-0.3106 C	-0.3110 C	-0.13%	↑	-0.03%

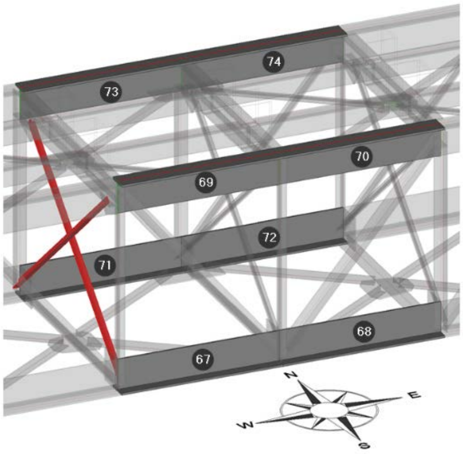

#	Measure	Ref	UD	D	$\Delta\varepsilon / \|\varepsilon_u\|$ [%]		$\Delta\varepsilon/\varepsilon_y$ [%]
67	[mε]	N	0.4084 T	0.4091 T	0.18%	↑	0.05%
68	[mε]	N	0.4016 T	0.4013 T	-0.10%	↓	-0.02%
69	[mε]	N	-0.3059 C	-0.3066 C	-0.23%	↑	-0.05%
70	[mε]	N	-0.3490 C	-0.3463 C	0.78%	↓	0.17%

#	Unit	Ref	UD	D	$\Delta\varepsilon / \|\varepsilon_u\|$ [%]		$\Delta\varepsilon/\varepsilon_y$ [%]
71	[mε]	N	0.3896 T	0.3863 T	-0.84%	↓	-0.21%
72	[mε]	N	0.4013 T	0.4006 T	-0.19%	↓	-0.05%
73	[mε]	N	-0.3229 C	-0.3219 C	0.29%	↓	0.06%
74	[mε]	N	-0.3106 C	-0.3108 C	-0.06%	↑	-0.01%

Fig. 59 | Strain results for the main chords in the centre of the gravity and centre of the length of the monitored elements, only representatives of the axial forces.

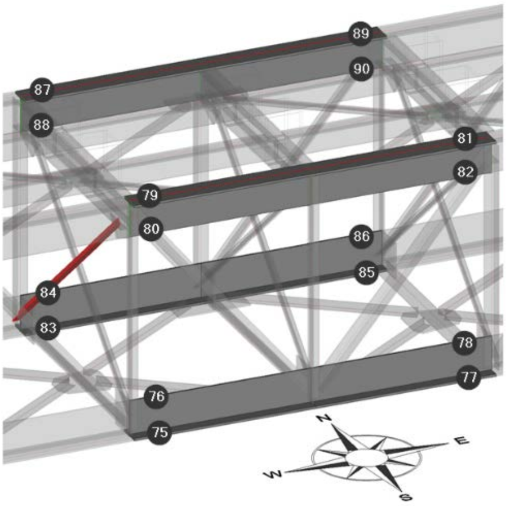

#	Unit	Ref	UD	D	Δε / |ε_U| [%]		Δε/ε_Y [%]
75	[mε]	N,M	0.5197 T	0.5245 T	0.91%	↑	0.30%
76	[mε]	N,M	0.1932 T	0.1930 T	-0.08%	↓	-0.01%
77	[mε]	N,M	0.4801 T	0.4788 T	-0.28%	↓	-0.09%
78	[mε]	N,M	0.2400 T	0.2397 T	-0.14%	↓	-0.02%
79	[mε]	N,M	-0.4714 C	-0.4775 C	-1.29%	↑	-0.39%
80	[mε]	N,M	-0.2030 C	-0.2152 C	-6.02%	↑	-0.78%
81	[mε]	N,M	-0.3772 C	-0.3772 C	0.00%	↑	0.00%
82	[mε]	N,M	-0.3975 C	-0.3974 C	0.04%	↓	0.01%

#	Unit	Ref	UD	D	Δε / |ε_U| [%]		Δε/ε_Y [%]
83	[mε]	N,M	0.4780 T	0.4709 T	-1.48%	↓	-0.45%
84	[mε]	N,M	0.2743 T	0.2854 T	4.06%	↑	0.71%
85	[mε]	N,M	0.5061 T	0.5075 T	0.29%	↑	0.09%
86	[mε]	N,M	0.1717 T	0.1696 T	-1.21%	↓	-0.13%
87	[mε]	N,M	-0.4794 C	-0.4757 C	0.78%	↓	0.24%
88	[mε]	N,M	-0.1419 C	-0.1480 C	-4.30%	↑	-0.39%
89	[mε]	N,M	-0.4104 C	-0.4108 C	-0.10%	↑	-0.03%
90	[mε]	N,M	-0.1853 C	-0.1829 C	1.34%	↓	0.16%

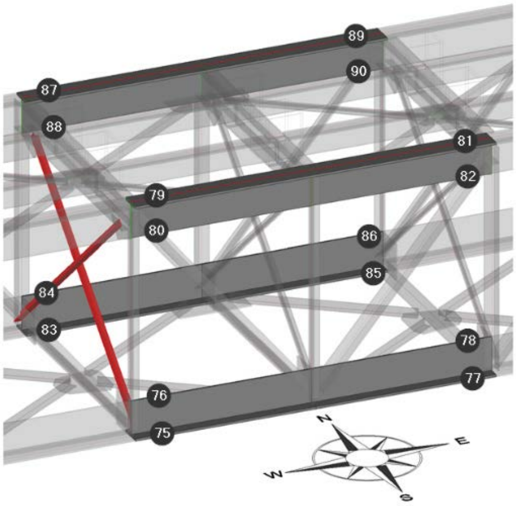

#	Unit	Ref	UD	D	Δε / |ε_U| [%]		Δε/ε_Y [%]
75	[mε]	N,M	0.5197 T	0.5204 T	0.13%	↑	0.04%
76	[mε]	N,M	0.1932 T	0.1961 T	1.52%	↑	0.19%
77	[mε]	N,M	0.4801 T	0.4791 T	-0.22%	↓	-0.07%
78	[mε]	N,M	0.2400 T	0.2395 T	-0.20%	↓	-0.03%
79	[mε]	N,M	-0.4714 C	-0.4750 C	-0.76%	↑	-0.23%
80	[mε]	N,M	-0.2030 C	-0.2131 C	-4.97%	↑	-0.64%
81	[mε]	N,M	-0.3772 C	-0.3757 C	0.40%	↓	0.10%
82	[mε]	N,M	-0.3975 C	-0.3957 C	0.45%	↓	0.11%

#	Unit	Ref	UD	D	Δε / |ε_U| [%]		Δε/ε_Y [%]
83	[mε]	N,M	0.4780 T	0.4727 T	-1.10%	↓	-0.33%
84	[mε]	N,M	0.2743 T	0.2832 T	3.25%	↑	0.57%
85	[mε]	N,M	0.5061 T	0.5067 T	0.13%	↑	0.04%
86	[mε]	N,M	0.1717 T	0.1691 T	-1.49%	↓	-0.16%
87	[mε]	N,M	-0.4794 C	-0.4784 C	0.20%	↓	0.06%
89	[mε]	N,M	-0.4104 C	-0.4099 C	0.10%	↓	0.03%
90	[mε]	N,M	-0.1853 C	-0.1844 C	0.51%	↓	0.06%

Fig. 60 | Strain results for the main chords near joints and far from the centre of gravity of the monitored elements, representing both axial and bending forces.

In summary, for the considered load setup, the partial and total loss of the vertical bracing system does not significantly affect the shear force transfer paths, nor does it compromise the truss's bending resistance, inducing only slight torsion in the structure. Consequently, this is characterised as a damage scenario in which load redistribution and ALP activation occur locally, primarily affecting elements in the immediate vicinity of the affected zone that are capable of transmitting vertical loads. This confirms the primary purpose of these elements, which is to counteract horizontal loads, resist unsymmetrical loading, and keep the structure braced. When only vertical loads are present, the failure of these elements does not represent a critical scenario.

1.4.7 DS – Loss of a transversal beam

Fig. 61 shows the definition of this damage scenario, which was reproduced in two levels of damage. In the first damage level, only the central part of the transversal beam was cut (partial loss), while in the second level of damage the whole transversal beam was removed (total loss).

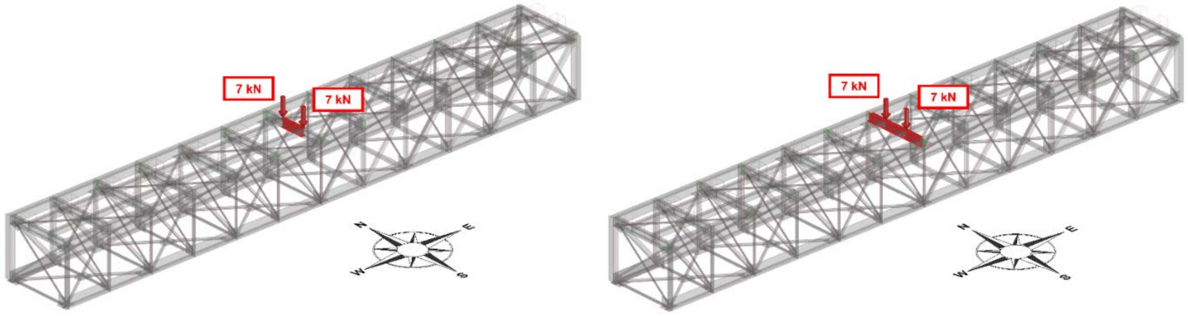

1st level of damage: Partial loss *2nd level of damage: Total loss*

Fig. 61 | Bridge, loads and damage scenario identified by the red colour elements for the first and second level of damage.

The results obtained for vertical displacements and distortions are shown in Fig. 62 and Fig. 63. Each figure includes the references and units for the sensor measurements: d for displacements (in mm) and θ for distortions (in degrees). Additionally, the absolute values recorded by each sensor in undamaged (UD) and damaged (D) conditions are presented, along with the processed results, as discussed at the beginning of Section 1.4. From the results, it can be concluded that partial and total loss of the transversal beam does not significantly affect the vertical stiffness of the bridge. Furthermore, it is evident that such losses do not induce substantial vertical displacements or distortions. However, in the case of the total loss of the transversal beam, the bridge's response in its central part is characterised by a slight reduction in the vertical displacements of the nodes in the same transverse plane as the removed element. This is due to a portion of the load from the transversal beam being transferred to the adjacent transversal beams.

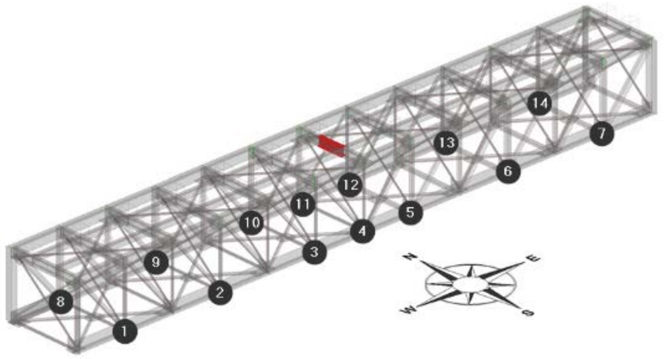

#	Unit	Ref	UD	D	Δd / $\|d_U\|$ [%]
1	[mm]	d	0.47	0.49	4.20%
2	[mm]	d	1.06	1.12	6.21%
3	[mm]	d	1.52	1.60	5.57%
4	[mm]	d	1.72	1.84	6.95%
5	[mm]	d	1.52	1.64	8.09%
6	[mm]	d	1.06	1.13	6.33%
7	[mm]	d	0.48	0.50	3.27%

#	Unit	Ref	UD	D	Δd / $\|d_U\|$ [%]
8	[mm]	d	0.47	0.50	6.88%
9	[mm]	d	1.05	1.10	4.52%
10	[mm]	d	1.52	1.60	5.51%
11	[mm]	d	1.69	1.81	6.72%
12	[mm]	d	1.46	1.55	5.50%
13	[mm]	d	0.99	1.03	4.10%
14	[mm]	d	0.36	0.39	9.21%

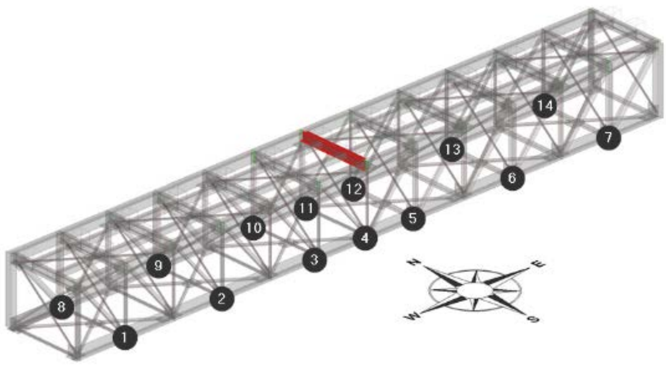

#	Unit	Ref	UD	D	Δd / $\|d_U\|$ [%]
1	[mm]	d	0.47	0.49	2.78%
2	[mm]	d	1.06	1.10	4.49%
3	[mm]	d	1.52	1.63	7.15%
4	[mm]	d	1.72	1.68	-2.33%
5	[mm]	d	1.52	1.60	5.09%
6	[mm]	d	1.06	1.11	4.21%
7	[mm]	d	0.48	0.49	1.09%

#	Unit	Ref	UD	D	Δd / $\|d_U\|$ [%]
8	[mm]	d	0.47	0.49	4.06%
9	[mm]	d	1.05	1.07	1.79%
10	[mm]	d	1.52	1.57	3.31%
11	[mm]	d	1.69	1.62	-4.21%
12	[mm]	d	1.46	1.57	7.27%
13	[mm]	d	0.99	1.03	3.74%
14	[mm]	d	0.36	0.39	9.20%

Fig. 62 | Vertical displacement results for the two levels of damage.

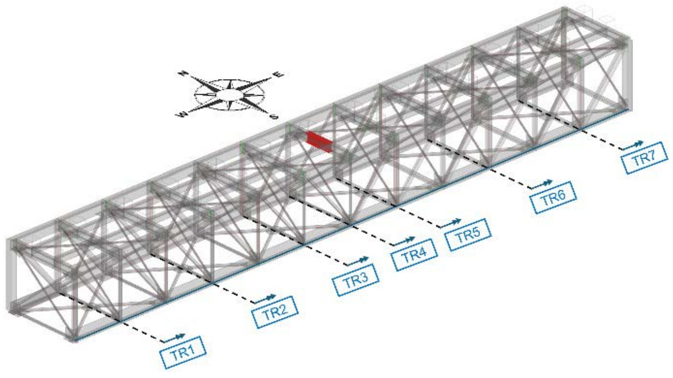

#	Unit	Ref	UD	D	$\Delta\theta / \lvert\theta_U\rvert$ [%]
TR1	[º]	θ	0.000	-0.001	-299.67%
TR2	[º]	θ	0.001	0.002	209.25%
TR3	[º]	θ	0.000	0.000	-52.13%
TR4	[º]	θ	0.002	0.002	25.06%
TR5	[º]	θ	0.005	0.008	76.20%
TR6	[º]	θ	0.006	0.008	36.75%
TR7	[º]	θ	0.010	0.009	-13.21%

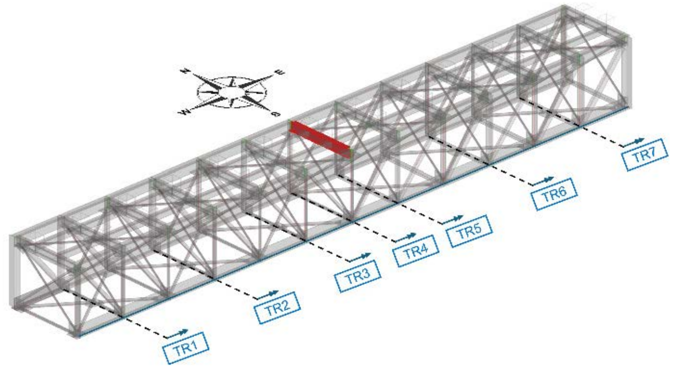

#	Unit	Ref	UD	D	$\Delta\theta / \lvert\theta_U\rvert$ [%]
TR1	[º]	θ	0.000	0.000	-142.29%
TR2	[º]	θ	0.001	0.003	326.98%
TR3	[º]	θ	0.000	0.005	-3418.90%
TR4	[º]	θ	0.002	0.004	142.34%
TR5	[º]	θ	0.005	0.002	-52.25%
TR6	[º]	θ	0.006	0.007	10.62%
TR7	[º]	θ	0.010	0.008	-21.39%

Fig. 63 | Distortion results for the two levels of damage.

The results of the measured strains are presented in Figures 64-70. Each figure details the monitored elements, including the unit of measurement for each sensor and whether the location is representative of axial forces (N) or both axial forces and moments (N, M). The absolute values recorded by each sensor are displayed for both undamaged (UD) and damaged (D) conditions, along with an indication of whether the values represent tension (T) or compression (C). The data were processed as described at the start of Section 1.4. This analysis enables the following conclusions to be drawn:

- *Horizontal bracings* (Fig. 64): The lower horizontal bracing system cannot be considered as critical in the load redistribution and the ALPs following the loss of the transversal beam. This is due to the fact that the elements of the bracing system are not highly sensitive to element removal and exhibit low $\Delta\varepsilon/\varepsilon_y$ ratios. These factors highlight the reduced importance of the bracing system in the structural response.
- *Vertical bracings* (Fig. 65): The vertical bracing system cannot be considered a critical element in the redistribution of loads following the partial or total loss of the transversal beam. Ratios below 1.00% reveal the limited contribution of the vertical bracing to load transfer.
- *Verticals* (Fig. 66): The verticals do not significantly contribute to load redistribution when the transversal beam is removed. Ratios $\Delta\varepsilon/\varepsilon_y$ lower than 0.30% indicate the low importance of these elements in the structural response for the damage scenario analysed.
- *Diagonals* (Fig. 67): The diagonals do not significantly contribute to load redistribution in the event of partial or total loss of the transversal beam. Ratios $\Delta\varepsilon/\varepsilon_y$ lower than 0.21% highlight the low importance of these elements.
- *Chords* (Fig. 68 & Fig. 69): The redistribution of loads occurs through the chords, but the activation of ALPs cannot be characterised by the axial loads travelling through them. Ratios $\Delta\varepsilon/\varepsilon_y$ below 1.00% reveal the low importance of these elements and their insensitivity to the damage scenarios. While the redistribution of loads and activation of ALPs are not associated with axial loads through the chords, they are clearly identifiable through the occurrence of bending moments. Ratios $\Delta\varepsilon/\varepsilon_y$ above 5.00% indicate that bending moments play a significant role in load redistribution. In the case of partial loss of the transversal beam, vertical loads continue to be transferred to the central part of the truss, with hogging bending moments

appearing in the joint with the chords. Conversely, with the total loss of the transversal beam, the load is transferred through the longitudinal members (stringers) to the adjacent transversal beams via bending mechanisms. This bending induces torsion in the adjacent transversal beams, leading to sagging bending moments at the ends of the upper chords.

- <u>*Adjacent stringers*</u> (Fig. 70): The removal of the transversal beam significantly affects the adjacent stringers, to the extent that load redistribution and ALP activation primarily occur through them. The loss of the central transversal beam reconfigures the stringers' behaviour, resulting in beam-like behaviour equivalent to twice its original length. This behaviour is characterised by hogging bending moments at its ends and sagging bending moments at the point of load application (where the failure occurs). This mechanism begins with the partial loss and consolidates with the total loss, causing large vertical displacements and leading to higher strains that ultimately cause yielding in the adjacent stringers.

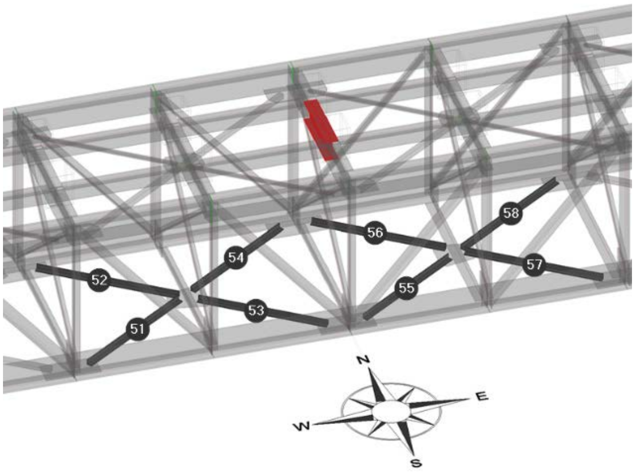

#	Unit	Ref	UD	D	$\Delta\varepsilon / \lvert\varepsilon_U\rvert$ [%]		$\Delta\varepsilon/\varepsilon_y$ [%]
51	[mε]	N	0.0493 T	0.0535 T	8.52%	↑	0.27%
52	[mε]	N	0.0455 T	0.0591 T	29.97%	↑	0.87%
53	[mε]	N	0.0471 T	0.0577 T	22.35%	↑	0.67%
54	[mε]	N	0.0559 T	0.0422 T	-24.55%	↓	-0.87%
55	[mε]	N	0.0641 T	0.0698 T	8.82%	↑	0.36%
56	[mε]	N	0.0355 T	0.0257 T	-27.51%	↓	-0.62%
57	[mε]	N	0.0351 T	0.0375 T	6.76%	↑	0.15%
58	[mε]	N	0.0524 T	0.0608 T	16.11%	↑	0.54%

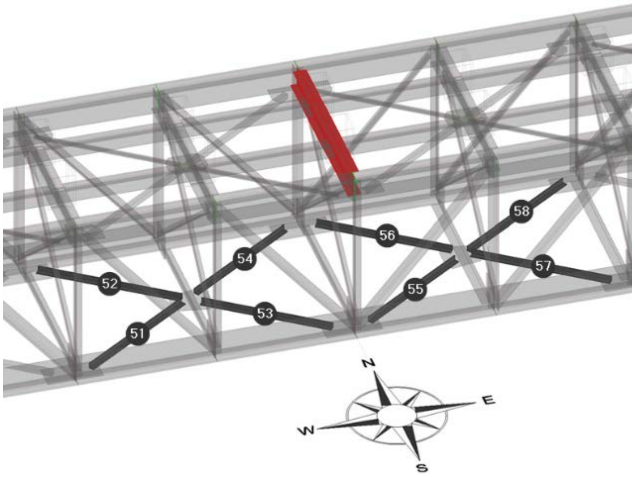

#	Unit	Ref	UD	D	$\Delta\varepsilon / \lvert\varepsilon_U\rvert$ [%]		$\Delta\varepsilon/\varepsilon_y$ [%]
51	[mε]	N	0.0493 T	0.0507 T	2.73%	↑	0.09%
52	[mε]	N	0.0455 T	0.0458 T	0.73%	↑	0.02%
53	[mε]	N	0.0471 T	0.0488 T	3.50%	↑	0.10%
54	[mε]	N	0.0559 T	0.0607 T	8.54%	↑	0.30%
55	[mε]	N	0.0641 T	0.0675 T	5.20%	↑	0.21%
56	[mε]	N	0.0355 T	0.0437 T	23.13%	↑	0.52%
57	[mε]	N	0.0351 T	0.0438 T	24.72%	↑	0.55%
58	[mε]	N	0.0524 T	0.0512 T	-2.31%	↓	-0.08%

Fig. 64 | Strain results for the horizontal lower bracing monitored elements.

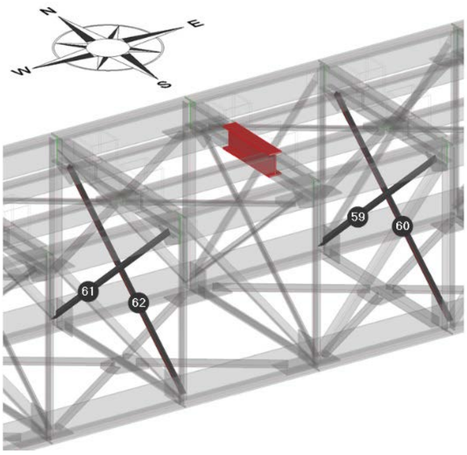

#	Unit	Ref	UD	D	$\Delta\varepsilon / \|\varepsilon_U\|$ [%]		$\Delta\varepsilon/\varepsilon_y$ [%]
59	[mε]	N	-0.0200 C	-0.0372 C	-86.29%	↑	-1.09%
60	[mε]	N	-0.0233 C	-0.0195 C	16.30%	↓	0.24%
61	[mε]	N	-0.0184 C	-0.0256 C	-39.36%	↑	-0.46%
62	[mε]	N	-0.0197 C	-0.0253 C	-28.31%	↑	-0.35%

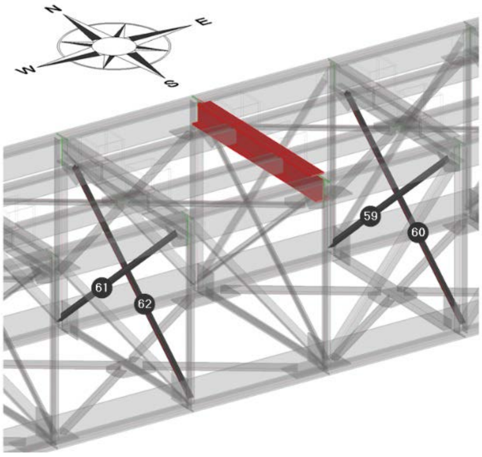

#	Unit	Ref	UD	D	$\Delta\varepsilon / \|\varepsilon_U\|$ [%]		$\Delta\varepsilon/\varepsilon_y$ [%]
59	[mε]	N	-0.0200 C	-0.0346 C	-73.64%	↑	-0.93%
60	[mε]	N	-0.0233 C	-0.0340 C	-46.25%	↑	-0.68%
61	[mε]	N	-0.0184 C	-0.0419 C	-127.96%	↑	-1.49%
62	[mε]	N	-0.0197 C	-0.0276 C	-40.22%	↑	-0.50%

Fig. 65 | Strain results for the vertical bracing monitored elements.

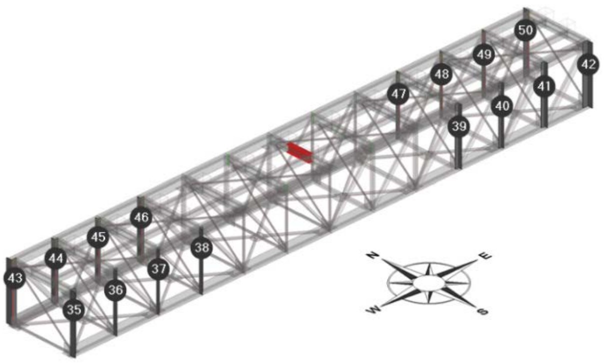

#	Unit	Ref	UD	D	$\Delta\varepsilon / \|\varepsilon_U\|$ [%]		$\Delta\varepsilon/\varepsilon_y$ [%]
35	[mε]	N,M	-0.0088 C	-0.0085 C	4.02%	↓	0.02%
36	[mε]	N,M	-0.0345 C	-0.0344 C	0.15%	↓	0.00%
37	[mε]	N,M	-0.0695 C	-0.0702 C	-1.03%	↑	-0.05%
38	[mε]	N,M	-0.0399 C	-0.0401 C	-0.47%	↑	-0.01%
39	[mε]	N,M	-0.0373 C	-0.0372 C	0.18%	↓	0.00%
40	[mε]	N,M	-0.0789 C	-0.0820 C	-4.04%	↑	-0.20%
41	[mε]	N,M	-0.0427 C	-0.0432 C	-1.19%	↑	-0.03%
42	[mε]	N,M	-0.0079 C	-0.0069 C	11.83%	↓	0.06%

#	Unit	Ref	UD	D	$\Delta\varepsilon / \|\varepsilon_U\|$ [%]		$\Delta\varepsilon/\varepsilon_y$ [%]
43	[mε]	N,M	-0.0082 C	-0.0085 C	-3.47%	↑	-0.02%
44	[mε]	N,M	-0.0470 C	-0.0471 C	-0.31%	↑	-0.01%
45	[mε]	N,M	-0.0753 C	-0.0779 C	-3.45%	↑	-0.17%
46	[mε]	N,M	-0.0343 C	-0.0361 C	-5.31%	↑	-0.12%
47	[mε]	N,M	-0.0411 C	-0.0406 C	1.10%	↓	0.03%
48	[mε]	N,M	-0.0878 C	-0.0880 C	-0.25%	↑	-0.01%
49	[mε]	N,M	-0.0424 C	-0.0424 C	0.01%	↓	0.00%
50	[mε]	N,M	-0.0114 C	-0.0114 C	0.73%	↓	0.01%

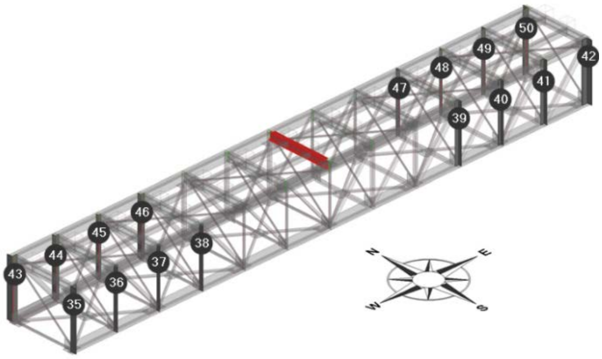

#	Unit	Ref	UD	D	$\Delta\varepsilon / \|\varepsilon_U\|$ [%]		$\Delta\varepsilon/\varepsilon_y$ [%]
35	[mε]	N,M	-0.0088 C	-0.0068 C	23.21%	↓	0.13%
36	[mε]	N,M	-0.0345 C	-0.0307 C	10.92%	↓	0.24%
37	[mε]	N,M	-0.0695 C	-0.0679 C	2.30%	↓	0.10%
38	[mε]	N,M	-0.0399 C	-0.0352 C	11.71%	↓	0.30%
39	[mε]	N,M	-0.0373 C	-0.0364 C	2.37%	↓	0.06%
40	[mε]	N,M	-0.0789 C	-0.0786 C	0.34%	↓	0.02%
41	[mε]	N,M	-0.0427 C	-0.0412 C	3.44%	↓	0.09%
42	[mε]	N,M	-0.0079 C	-0.0075 C	4.42%	↓	0.02%

#	Unit	Ref	UD	D	$\Delta\varepsilon / \|\varepsilon_U\|$ [%]		$\Delta\varepsilon/\varepsilon_y$ [%]
43	[mε]	N,M	-0.0082 C	-0.0047 C	42.41%	↓	0.22%
44	[mε]	N,M	-0.0470 C	-0.0474 C	-0.84%	↑	-0.02%
45	[mε]	N,M	-0.0753 C	-0.0727 C	3.50%	↓	0.17%
46	[mε]	N,M	-0.0343 C	-0.0295 C	13.81%	↓	0.30%
47	[mε]	N,M	-0.0411 C	-0.0387 C	5.70%	↓	0.15%
48	[mε]	N,M	-0.0878 C	-0.0866 C	1.38%	↓	0.08%
49	[mε]	N,M	-0.0424 C	-0.0418 C	1.45%	↓	0.04%
50	[mε]	N,M	-0.0114 C	-0.0092 C	19.30%	↓	0.14%

Fig. 66 | Strain results for the vertical monitored elements.

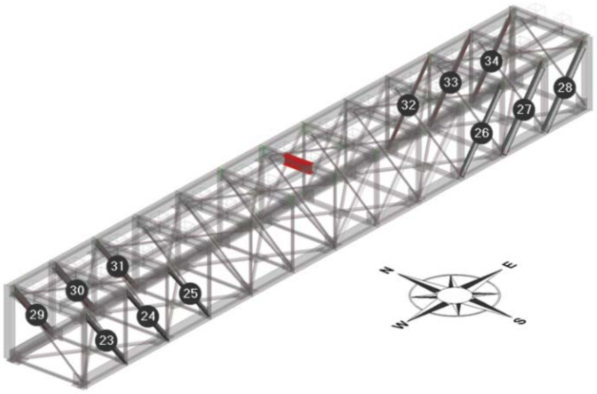

#	Unit	Ref	UD	D	Δε / |ε_U| [%]		Δε/ε_y [%]
23	[mε]	N	0.0810 T	0.0806 T	-0.52%	↓	-0.03%
24	[mε]	N	0.0905 T	0.0906 T	0.13%	↑	0.01%
25	[mε]	N	0.1283 T	0.1272 T	-0.88%	↓	-0.07%
26	[mε]	N	0.1201 T	0.1203 T	0.12%	↑	0.01%
27	[mε]	N	0.0809 T	0.0812 T	0.43%	↑	0.02%
28	[mε]	N	0.0750 T	0.0754 T	0.58%	↑	0.03%

#	Unit	Ref	UD	D	Δε / |ε_U| [%]		Δε/ε_y [%]
29	[mε]	N	0.0792 T	0.0796 T	0.58%	↑	0.03%
30	[mε]	N	0.0811 T	0.0818 T	0.87%	↑	0.04%
31	[mε]	N	0.1169 T	0.1163 T	-0.56%	↓	-0.04%
32	[mε]	N	0.1187 T	0.1182 T	-0.43%	↓	-0.03%
33	[mε]	N	0.0924 T	0.0917 T	-0.77%	↓	-0.04%
34	[mε]	N	0.0766 T	0.0750 T	-2.02%	↓	-0.10%

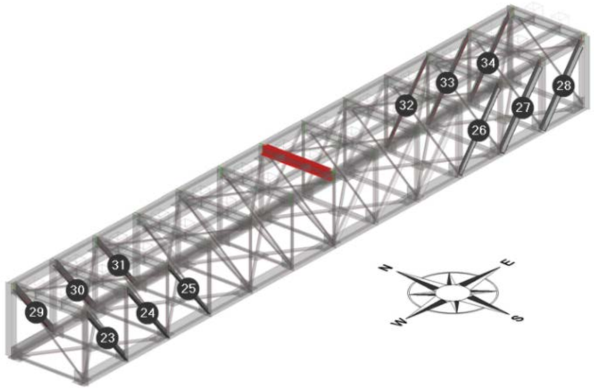

#	Unit	Ref	UD	D	Δε / |ε_U| [%]		Δε/ε_y [%]
23	[mε]	N	0.0810 T	0.0832 T	2.72%	↑	0.14%
24	[mε]	N	0.0905 T	0.0909 T	0.47%	↑	0.03%
25	[mε]	N	0.1283 T	0.1272 T	-0.88%	↓	-0.07%
26	[mε]	N	0.1201 T	0.1211 T	0.80%	↑	0.06%
27	[mε]	N	0.0809 T	0.0834 T	3.09%	↑	0.16%
28	[mε]	N	0.0750 T	0.0755 T	0.59%	↑	0.03%

#	Unit	Ref	UD	D	Δε / |ε_U| [%]		Δε/ε_y [%]
29	[mε]	N	0.0792 T	0.0825 T	4.19%	↑	0.21%
30	[mε]	N	0.0811 T	0.0817 T	0.77%	↑	0.04%
31	[mε]	N	0.1169 T	0.1197 T	2.39%	↑	0.18%
32	[mε]	N	0.1187 T	0.1203 T	1.28%	↑	0.10%
33	[mε]	N	0.0924 T	0.0935 T	1.20%	↑	0.07%
34	[mε]	N	0.0766 T	0.0784 T	2.45%	↑	0.12%

Fig. 67 | Strain results for the diagonal monitored elements.

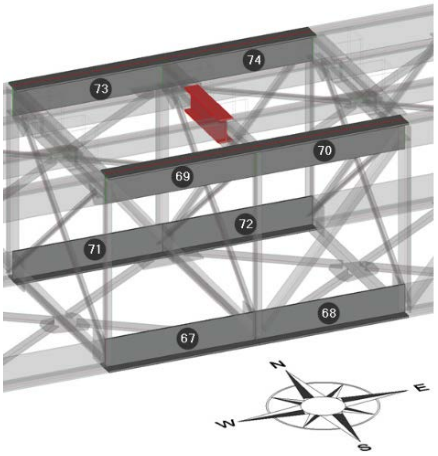

#	Measure	Ref	UD	D	Δε / |ε_U| [%]		Δε/ε_y [%]
67	[mε]	N	0.0732 T	0.0734 T	0.30%	↑	0.01%
68	[mε]	N	0.0775 T	0.0781 T	0.75%	↑	0.04%
69	[mε]	N	-0.0696 C	-0.0848 C	-21.81%	↑	-0.96%
70	[mε]	N	-0.0682 C	-0.0992 C	-45.38%	↑	-1.97%

#	Unit	Ref	UD	D	Δε / |ε_U| [%]		Δε/ε_y [%]
71	[mε]	N	0.0767 T	0.0713 T	-7.03%	↓	-0.34%
72	[mε]	N	0.0727 T	0.0704 T	-3.21%	↓	-0.15%
73	[mε]	N	-0.0690 C	-0.0761 C	-10.30%	↑	-0.45%
74	[mε]	N	-0.0702 C	-0.0841 C	-19.87%	↑	-0.89%

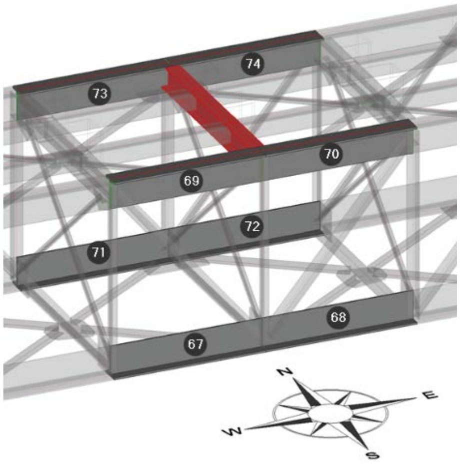

#	Measure	Ref	UD	D	Δε / |ε_U| [%]		Δε/ε_y [%]
67	[mε]	N	0.0732 T	0.0755 T	3.09%	↑	0.14%
68	[mε]	N	0.0775 T	0.0796 T	2.71%	↑	0.13%
69	[mε]	N	-0.0696 C	-0.0715 C	-2.75%	↑	-0.12%
70	[mε]	N	-0.0682 C	-0.0729 C	-6.81%	↑	-0.29%

#	Unit	Ref	UD	D	Δε / |ε_U| [%]		Δε/ε_y [%]
71	[mε]	N	0.0767 T	0.0774 T	0.86%	↑	0.04%
72	[mε]	N	0.0727 T	0.0767 T	5.49%	↑	0.25%
73	[mε]	N	-0.0690 C	-0.0676 C	2.05%	↓	0.09%
74	[mε]	N	-0.0702 C	-0.0712 C	-1.48%	↑	-0.07%

Fig. 68 | Strain results for the main chords in the centre of the gravity and centre of the length of the monitored elements, only representatives of the axial forces.

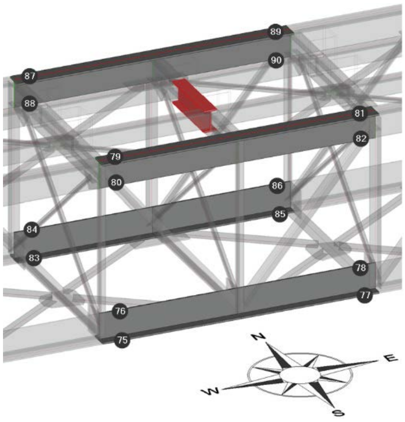

#	Unit	Ref	UD	D	$\Delta\varepsilon / \lvert\varepsilon_u\rvert$ [%]		$\Delta\varepsilon/\varepsilon_y$ [%]
75	[mε]	N,M	0.0899 T	0.0949 T	5.47%	↑	0.31%
76	[mε]	N,M	0.0473 T	0.0416 T	-12.05%	↓	-0.36%
77	[mε]	N,M	0.0904 T	0.0928 T	2.67%	↑	0.15%
78	[mε]	N,M	0.0517 T	0.0454 T	-12.18%	↓	-0.40%
79	[mε]	N,M	-0.0811 C	-0.0861 C	-6.09%	↑	-0.31%
80	[mε]	N,M	-0.0975 C	-0.1198 C	-22.86%	↑	-1.42%
81	[mε]	N,M	-0.0859 C	-0.1179 C	-37.16%	↑	-2.03%
82	[mε]	N,M	-0.1033 C	-0.1427 C	-38.12%	↑	-2.50%

#	Unit	Ref	UD	D	$\Delta\varepsilon / \lvert\varepsilon_u\rvert$ [%]		$\Delta\varepsilon/\varepsilon_y$ [%]
83	[mε]	N,M	0.0846 T	0.0922 T	9.05%	↑	0.49%
84	[mε]	N,M	0.0650 T	0.0391 T	-39.89%	↓	-1.65%
85	[mε]	N,M	0.0865 T	0.0875 T	1.15%	↑	0.06%
86	[mε]	N,M	0.0455 T	0.0365 T	-19.71%	↓	-0.57%
87	[mε]	N,M	-0.0847 C	-0.0988 C	-16.55%	↑	-0.89%
88	[mε]	N,M	-0.0836 C	-0.0980 C	-17.27%	↑	-0.92%
89	[mε]	N,M	-0.0841 C	-0.0819 C	2.66%	↓	0.14%
90	[mε]	N,M	-0.1134 C	-0.1736 C	-52.99%	↑	-3.82%

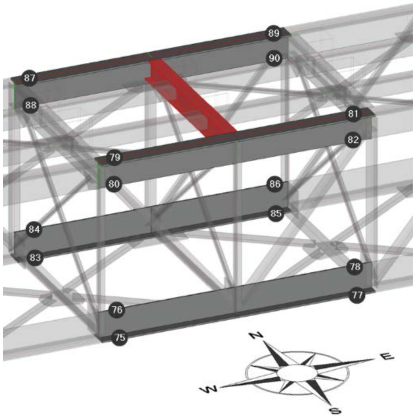

#	Unit	Ref	UD	D	$\Delta\varepsilon / \lvert\varepsilon_u\rvert$ [%]		$\Delta\varepsilon/\varepsilon_y$ [%]
75	[mε]	N,M	0.0899 T	0.0958 T	6.57%	↑	0.38%
76	[mε]	N,M	0.0473 T	0.0401 T	-15.17%	↓	-0.46%
77	[mε]	N,M	0.0904 T	0.1021 T	12.92%	↑	0.74%
78	[mε]	N,M	0.0517 T	0.0444 T	-14.21%	↓	-0.47%
79	[mε]	N,M	-0.0811 C	-0.1025 C	-26.34%	↑	-1.36%
80	[mε]	N,M	-0.0975 C	-0.0289 C	70.33%	↓	4.35%
81	[mε]	N,M	-0.0859 C	-0.0958 C	-11.44%	↑	-0.62%
82	[mε]	N,M	-0.1033 C	-0.0362 C	64.96%	↓	4.26%

#	Unit	Ref	UD	D	$\Delta\varepsilon / \lvert\varepsilon_u\rvert$ [%]		$\Delta\varepsilon/\varepsilon_y$ [%]
83	[mε]	N,M	0.0846 T	0.0945 T	11.74%	↑	0.63%
84	[mε]	N,M	0.0650 T	0.0529 T	-18.62%	↓	-0.77%
85	[mε]	N,M	0.0865 T	0.0954 T	10.21%	↑	0.56%
86	[mε]	N,M	0.0455 T	0.0376 T	-17.31%	↓	-0.50%
87	[mε]	N,M	-0.0847 C	-0.0992 C	-17.06%	↑	-0.92%
88	[mε]	N,M	-0.0836 C	-0.0001 C	99.92%	↓	5.30%
89	[mε]	N,M	-0.0841 C	-0.1022 C	-21.54%	↑	-1.15%
90	[mε]	N,M	-0.1134 C	-0.0298 C	73.69%	↓	5.31%

Fig. 69 | Strain results for the main chords near joints and far from the centre of gravity of the monitored elements, representing both axial and bending forces.

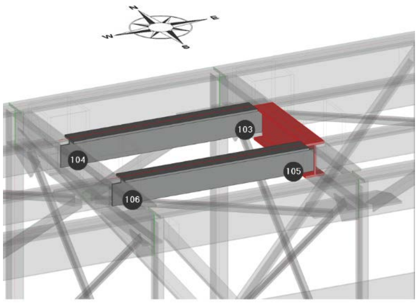

#	Unit	Ref	UD	D	$\Delta\varepsilon / \lvert\varepsilon_u\rvert$ [%]		$\Delta\varepsilon/\varepsilon_y$ [%]
103	[mε]	N,M	0.2102 T	0.1572 T	-25.20%	↓	-3.36%
104	[mε]	N,M	-0.1297 C	-0.4132 C	-218.61%	↑	-18.00%
105	[mε]	N,M	0.1581 T	0.1908 T	20.70%	↑	2.08%
106	[mε]	N, M	-0.1241 C	-0.3290 C	-165.14%	↑	-13.01%

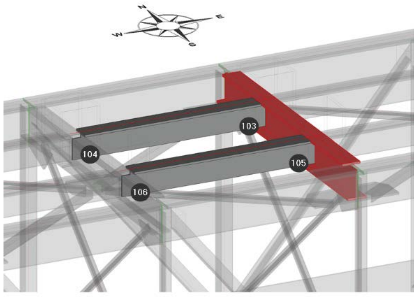

#	Unit	Ref	UD	D	$\Delta\varepsilon / \lvert\varepsilon_u\rvert$ [%]		$\Delta\varepsilon/\varepsilon_y$ [%]
103	[mε]	N, M	0.2102 T	5.3011 T	2421.66%	↑	323.26%
104	[mε]	N, M	-0.1297 C	-0.3399 C	-162.09%	↑	-13.35%
105	[mε]	N, M	0.1581 T	4.9258 T	3015.60%	↑	302.73%
106	[mε]	N, M	-0.1241 C	-0.7973 C	-542.56%	↑	-42.74%

Fig. 70 | Strain results for the adjacent stringers, near joints and far from the centre of gravity of the monitored elements, representing both axial and bending forces.

In summary, the loss of a transversal beam needs the active and significant participation of the adjacent stringers in the load redistribution. This redistribution through the stringers significantly affects the adjacent transversal beams, which, in turn, via flexural and torsional actions, exert minimal bending moments on the upper chords of the bridge. At this point, if the structure has efficiently activated this ALP, no further significant involvement of additional bridge elements is required for the bridge's stability.

Section 2. Evaluation of the first defence mechanisms after initial failure

Section 2.1: Description of the computational modelling

This section describes the three-dimensional (3D) Finite Element (FE) model of the scaled bridge using Diana FEA software[9]. As illustrated in Fig. 71, the model represents a steel truss-type bridge, consisting of an isostatic 6 m span, 0.7 m width, and 0.6 m height. The truss is symmetrical about the midspan and consists of twelve modules, each of 0.5 m in length. The reproduced geometry coincides with the specimen tested in Section 1. The bridge is modelled using two-node, three-dimensional fully numerically integrated Mindlin beam elements[9], where the normal strain varies linearly over the cross-section area and the transverse shear strains are forced to be constant.

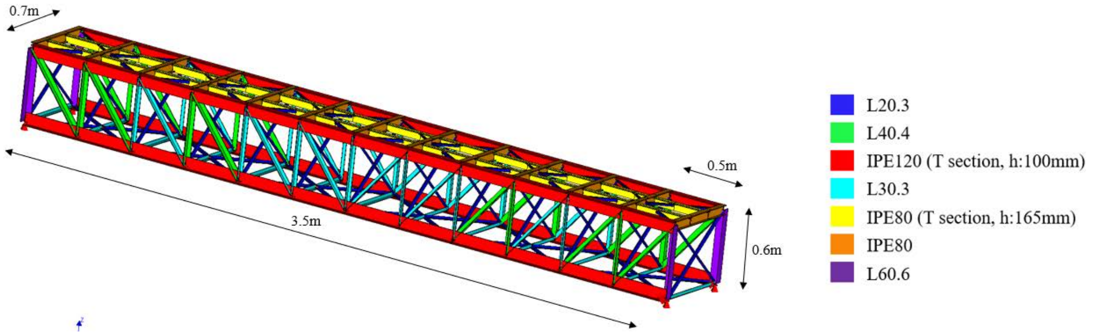

Fig. 71 | Numerical model with the adopted profiles.

The material used in the numerical model corresponds to the one characterised during the experimental campaign (see Section 1). Hence, the steel is simulated using the Von Mises Plasticity model, which accounts for both elastic and plastic behaviour. The material properties include Young's modulus of 207 GPa, a Poisson's ratio of 0.3, and a density of 7850 kg/m^3. The material exhibits a strain-hardening curve (see Fig. 4), where the stress progressively increases with plastic strain, reaching a peak of approximately 454 MPa. This model effectively covers strain hardening and damage-induced softening, and is thus suitable for scenarios involving large deformations and post-yield response. The boundary conditions reproduced the same applied experimentally, consisting of a rolling support at the western part, and a hinged support on the other side, where all translational displacements are constrained. As shown in Fig. 72, the two load setups are reproduced in accordance with the experimental campaign, either by applying four-point loads of 20 kN or two-point loads of 7 kN. Additionally, the total self-weight of the truss bridge, including the testing rig, welds, and gusset plates, is 9.58 kN. To accurately represent this in the numerical model, the testing rig is simulated as four nodal masses of 137.66 kg, applied at the same nodes as the load setup, while the connections are also represented as nodal masses. Regarding connection types, all truss joints are assumed to be rigid, except for the mid-connection of the vertical X-bracings, which are considered free to accurately replicate the scaled and the real bridge (see Fig. 72.a). Fig. 72.c shows the modelled finite element mesh.

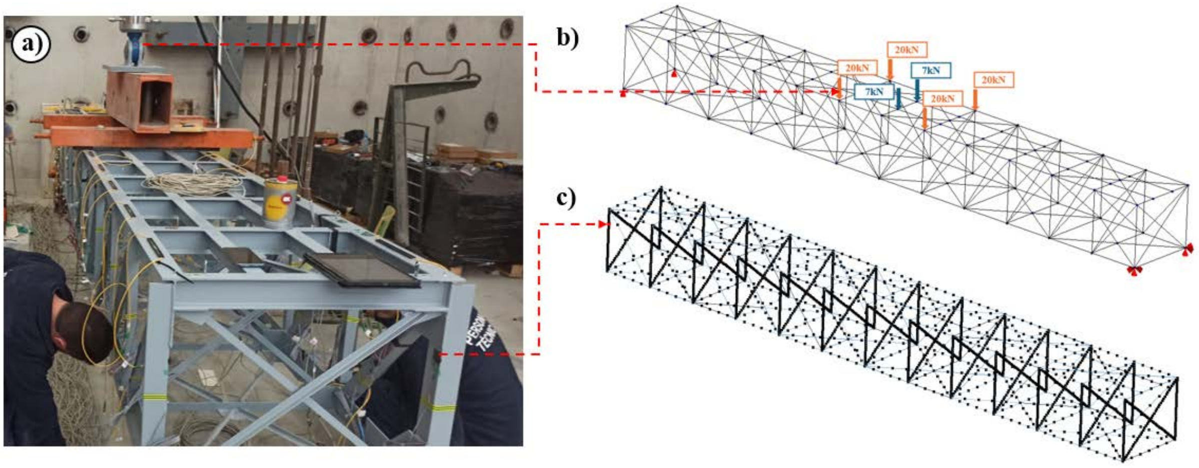

Fig. 72 | a) Scaled-down bridge, b) Numerical loading setups, c) Numerical mesh discretization.

Section 2.2: Validation of Simulation Strategy

In this section, the numerical model is validated before simulating the potential damage scenarios. The primary objective is to develop a reference model that accurately represents the actual behaviour of the bridge, ensuring a reliable simulation of potential structural damages. Thus, the validation process is performed by evaluating the numerical and experimental static responses, including vertical displacement and longitudinal strain. For this, the undamaged model (UD) and the nine damage scenarios conducted in the experimental campaign (DS1, DS2, etc.) are analysed. Fig. 73 demonstrates the considered damage scenarios for the validation, including:

- DS1: Loss of a lower chord.
- DS2: Loss of a diagonal.
- DS3: Loss of a vertical.
- DS4: Partial loss of horizontal lower bracing.
- DS5: Total loss of horizontal lower bracing.
- DS6: Partial loss of vertical bracing.
- DS7: Total loss of vertical bracing.
- DS8: Partial loss of transversal beam.
- DS9: Total loss of transversal beam.

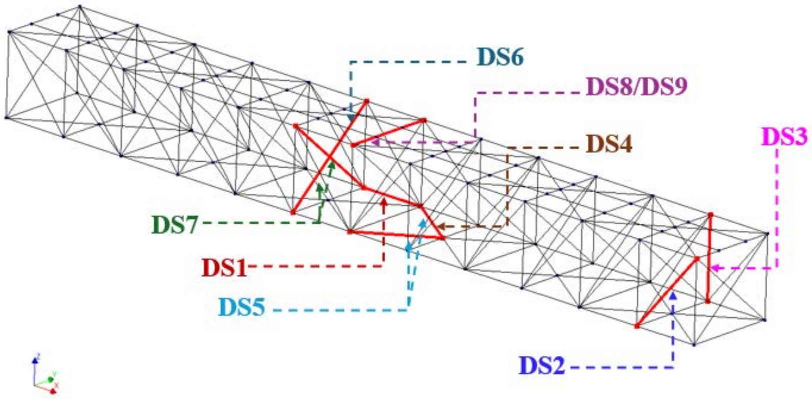

Fig. 73 | Considered damage scenarios in the validation.

2.2.1 Displacements

Following the experimental campaign that was performed using 14 displacement transducers (see Fig. 74.a), Fig. 74.b illustrates the corresponding monitored nodes in the numerical model.

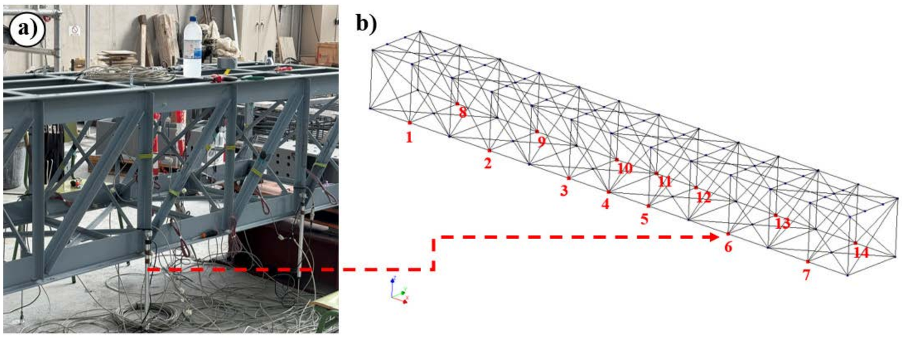

Fig. 74 | Vertical displacement sensors: a) Experimental setup, b) Numerical model.

To carry out the evaluation, Fig. 75 presents a comparative analysis of experimental and numerical vertical displacement values across Degrees of Freedom (DoFs) for all considered scenarios including the undamaged (UD) and damaged (DS1–DS9) conditions. In this regard, within all scenarios, the numerical and experimental displacement curves follow a similar trend, with peak displacements occurring at the mid-span regions, specifically node 4 on the south and node 11 on the north. The numerical results closely follow experimental trends with deviations remaining relatively small (~1 mm), while larger deviations are observed in mid-span regions, where structural deformation is most pronounced. In addition, DS1 exhibit the highest deviation, particularly nodes 10 and 11, due to its significantly larger displacement values compared to other scenarios.

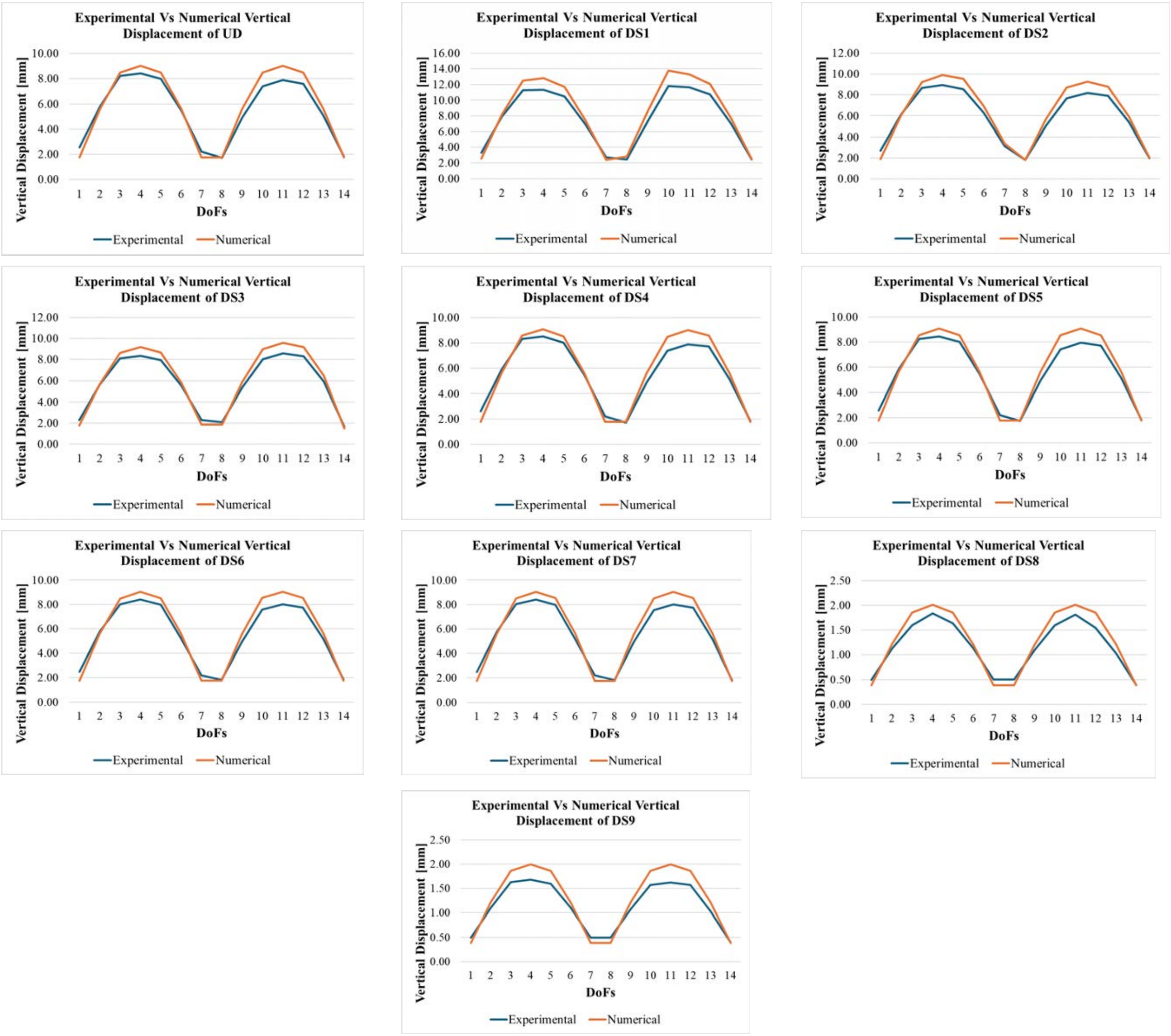

Fig. 75 | Experimental vs Numerical Vertical Displacement of each considered scenario.

2.2.2 Strains

This section aims to validate the numerical model using strain measurements considered in the experimental campaign, focusing on diagonals and central chords. The validation involves undamaged condition and all considered damage scenarios.

2.2.2.1 At Diagonals

Fig. 76.a demonstrates the experimental setup for measuring strain on diagonals, while Fig. 76.b presents the corresponding locations on the numerical model. Fig. 77 presents a comparison between experimental and numerical strain values across various measurement points for the undamaged (UD) and damaged (DS1 to DS9) conditions. While the numerical and experimental strain trends closely align overall, DS8 and DS9 exhibit very slight discrepancies, with the numerical model generally overestimating strain values at multiple measurement points (negligible).

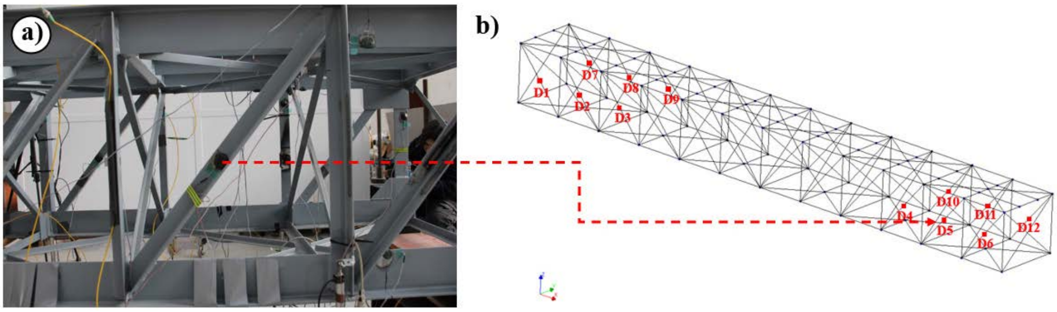

Fig. 76 | Selected locations for measuring strain at the diagonals: a) Experimental setup, b) Numerical model.

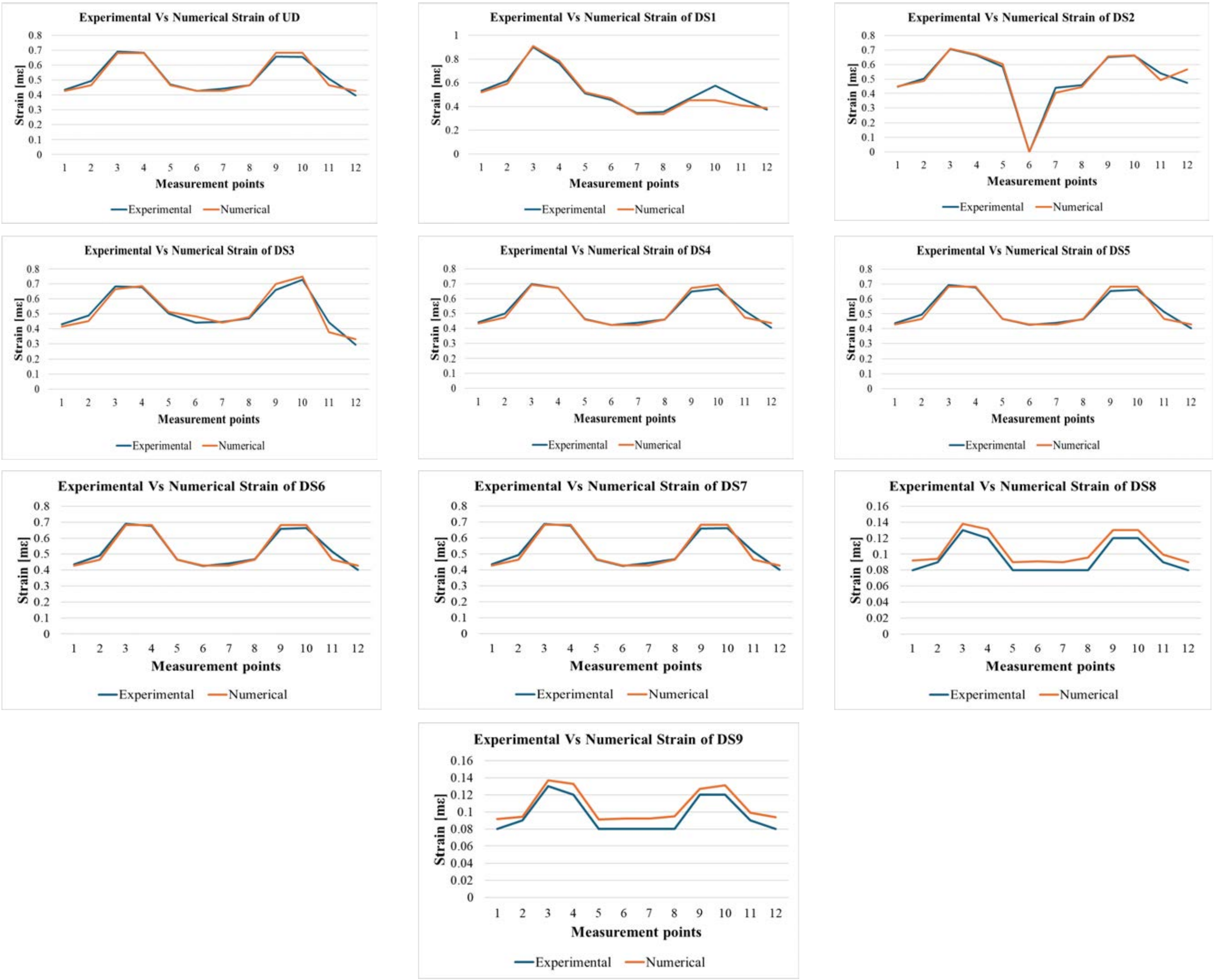

Fig. 77 | Experimental vs Numerical Strain (at diagonals) of each considered scenario.

2.2.2.2 At Central Chords

In Fig. 78.a, the experimental setup for measuring strain on central chords is shown, while in Fig. 78.b and Fig. 78.c show the corresponding locations on the numerical model. Fig. 79 and Fig. 80 illustrates the comparison between experimental and numerical strain values at measurement points located on the south side and north side, respectively. Overall, the numerical and experimental strain results for all considered scenarios (UD and DS1, etc) indicate a strong correlation. The results generally indicate small deviations across each scenario (UD, DS1 to DS9), confirming a strong alignment between the numerical model and experimental results.

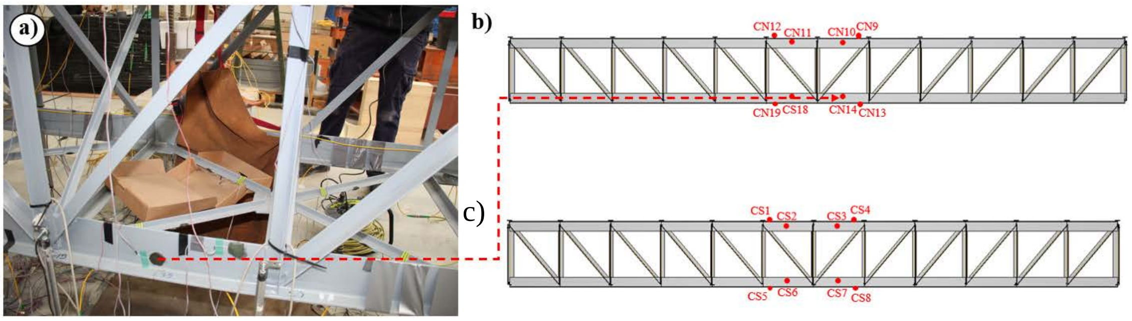

Fig. 78 | Selected points for measuring strain at the central chords: a) Experimental setup, b) North View of Numerical model, c) South View of Numerical model.

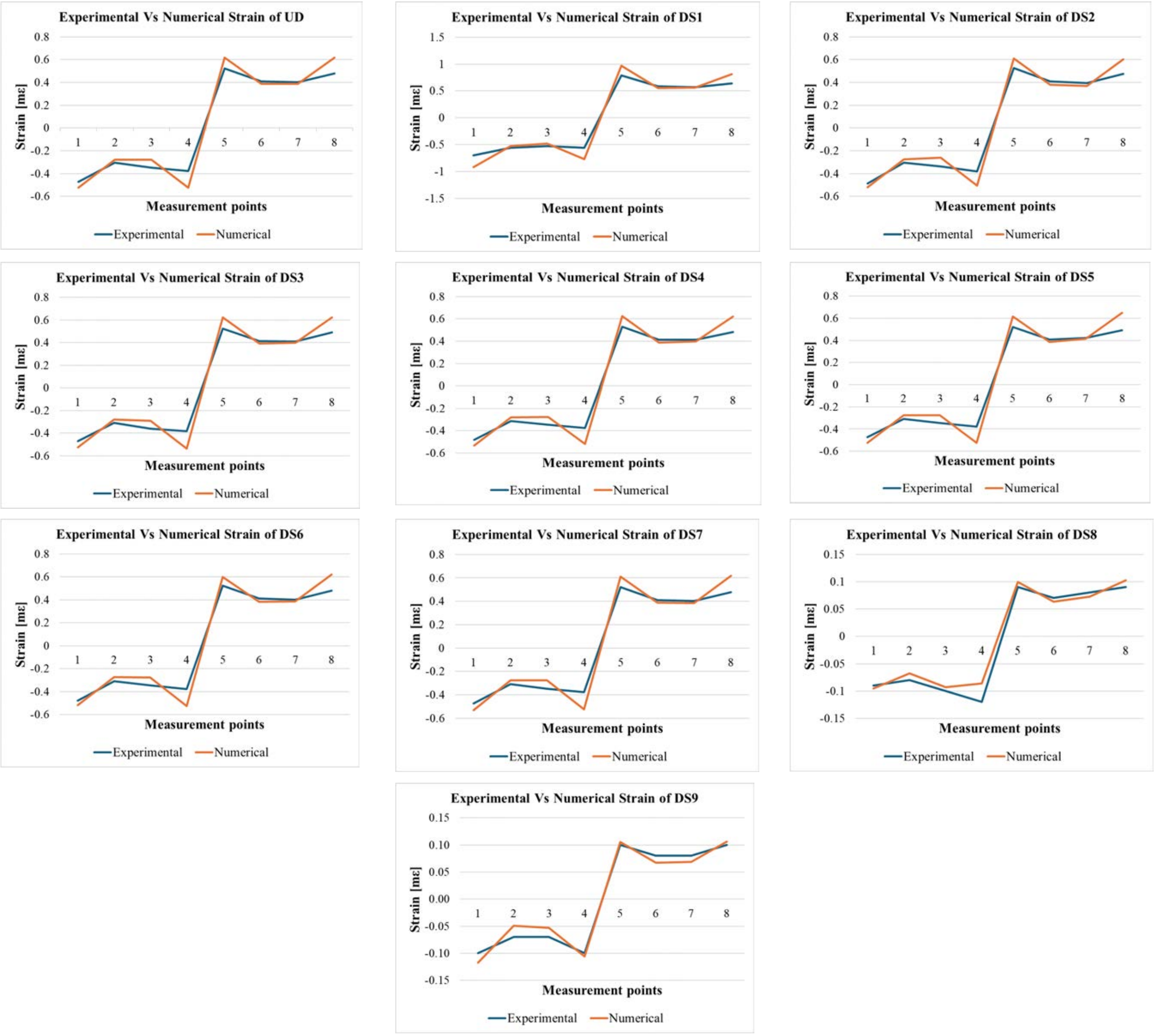

Fig. 79 | Experimental vs Numerical strain (at south central chords) of each considered scenario.

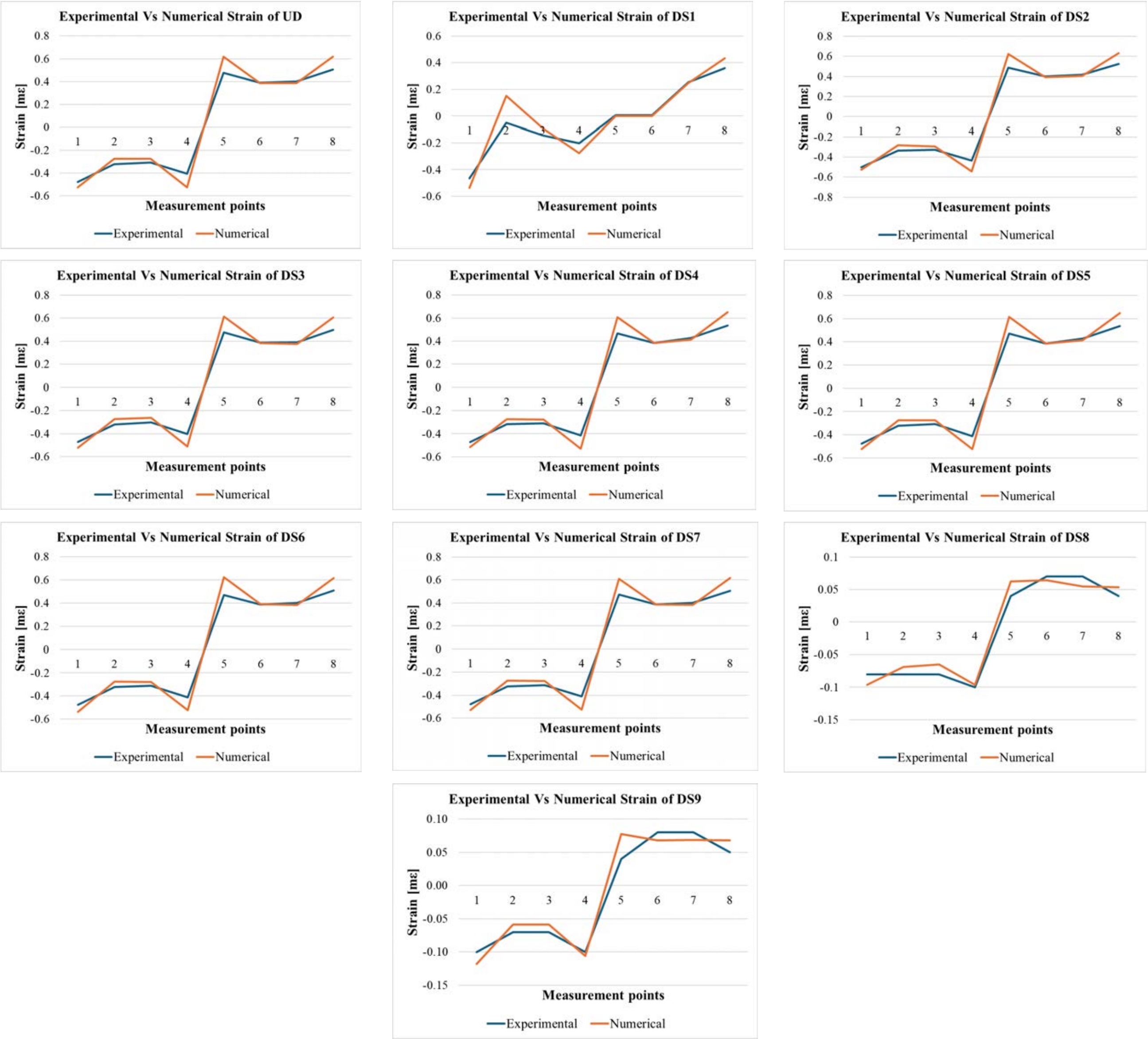

Fig. 80 | Experimental vs Numerical strain (at north central chords) of each considered scenario.

The results presented in graphs for both vertical displacement and strain indicate a strong alignment between numerical models and experimental measurements for all considered scenarios (UD, DS1 to DS9). However, the minimal discrepancies observed between the numerical and experimental results can be attributed to inherent material variability, boundary condition simplifications, and minor experimental uncertainties. Hence, this consistency highlights the reliability of the numerical model for simulating structural behaviour in potential damage scenarios.

Section 2.3: Simulation of Potential Damage Scenarios

After validation of the numerical model, this step involves simulating the potential damage scenarios. For this, the nonlinear analysis was performed for a total of 222 damage scenarios, each simulating the sudden loss of a single structural member between truss joints. All 222 simulations took 2 h 50 min to run (on a computer with 16 GB of RAM and an Intel(R) Core(TM) i7-9750H processor with a speed of 2.60 GHz).

In order to evaluate the structural response of the damaged scenarios relative to the undamaged condition, a series of absolute differences is calculated, serving as performance indicators. The following equations show the calculated performance indicators for each considered structural response. For instance, Eq. 8. and Eq. 9. illustrate the absolute error formulations for vertical displacement Dz and

vertical reaction force Rz, respectively. These correspond to the i-th damage scenario and j-th measured point, considering upper and lower truss joints for Dz and support nodes for Rz.

$$\Delta Dz_{i,j} = Dz_{i,j}^{damaged} - Dz_{j}^{undamaged} \quad \text{Eq. (8.)}$$

$$\Delta Rz_{i,j} = Rz_{i,j}^{damaged} - Rz_{j}^{undamaged} \quad \text{Eq. (9.)}$$

Regarding performance indicators of axial forces, Eq. 10. demonstrates the absolute error of the longitudinal axial forces Fx for the i-th damage scenario and k-th structural member.

$$\Delta Fx_{i,k} = Fx_{i,k}^{damaged} - Fx_{k}^{undamaged} \quad \text{Eq. (10.)}$$

Lastly, as illustrated in Eq. 11., the absolute error of the bending moment is calculated by comparing the moment differences at the beam end-nodes (j_start, j_end) of each structural member k between damage (i-th scenario) and undamaged state (Eq. 11.a and Eq. 11.b). It is good to mention that in order to account for the most critical variation in the bending moment distribution, the norm of vertical and transversal bending moment Myz is considered, as shows Eq. 11.c.

$$\Delta Myz_{i,k} = \begin{cases} \Delta Myz_{i,k,j_{end}} \ , & if \ \left|\Delta Myz_{i,k,j\_end}\right| > \left|\Delta Myz_{i,k,j\_start}\right| \\ \Delta Myz_{i,k,j\_start} \ , & otherwise \end{cases} \quad \text{Eq. (11.)}$$

$$\Delta Myz_{i,k,j\_end} = Myz_{i,k,j\_end}^{damaged} - Myz_{k,j\_end}^{undamaged} \quad \text{Eq. (11.a)}$$

$$\Delta Myz_{i,k,j\_start} = Myz_{i,k,j\_start}^{damaged} - Myz_{k,j\_start}^{undamaged} \quad \text{Eq. (11.b)}$$

$$Myz_{k,j} = \sqrt{My_{k,j}^{2} + Mz_{k,j}^{2}} \quad \text{Eq. (11.c)}$$

A colour scale is employed to enhance the visualization of internal forces (IF), axial forces and bending moments. A binary colour scheme has been adopted, where red represents an increase in IF magnitude (regardless of whether it corresponds to tension or compression), while blue indicates a decrease in IF magnitude. A gradient is applied to reflect the intensity of these changes, with darker shades representing higher magnitudes of increase or decrease. Elements whose indicators exhibit variations of less than 5% of the maximum value of the indicator across all structural elements for the evaluated DS are displayed in grey. This approach facilitates a clearer interpretation of the processed results and highlights the most significant differences between the damaged and undamaged conditions. The corresponding colour scale and its interpretation are provided in Fig. 81.

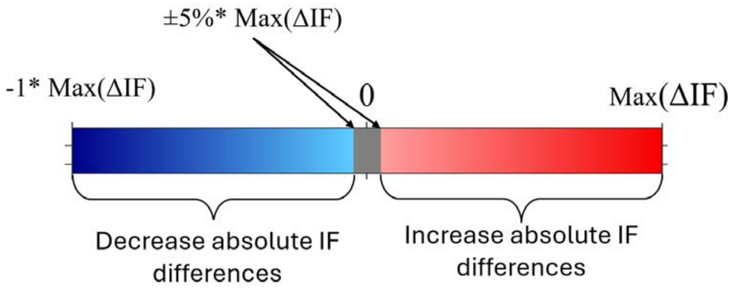

Fig. 81 | Performance Indicators range of values and colour convention.

Section 2.4: Results and Analyses

In this section, the obtained results are analysed to identify key observations that characterize the alternative load paths (ALPs). To achieve this, a heatmap of visualization was designed and employed. These heatmaps, in which the y-axis displays each damage scenario, providing a visual comparison that enhances the understanding of variations and patterns in the data. All the structural members are further subdivided based on their location including north, south, lower, and upper. The colour bar indicates the magnitude of response differences. In the heatmaps of axial forces, the colour bar has a specific meaning, from purple to blue denote compression while from yellow to dark red are tension values. White squares

(NaN) are the removed members. The illustrated results provide a deep insight into the impact of the different member failures on each response and along with each type of member.

2.4.1 Chord Removal Scenarios

In this section, the discussion of these results is presented, with a critical analysis of the common and specific structural patterns observed in these cases. The objective is to conclude which are the primary ALPs or defence mechanisms that the bridge relies on to resist the failure of any of its chords, particularly when the load level of the bridge is comparable to the real load associated with railway traffic.

At a global level, the increments in internal forces, as well as the variations in displacements and reactions resulting from the removal of a chord, can be estimated by applying an equivalent state to the structure (Fig. 82.a-c). In this equivalent state, forces equal and opposite (Fig. 82.c) to the axial force previously carried by the chord before its removal (Fig. 82.a) are imposed on the nodes to which the chord was originally attached.

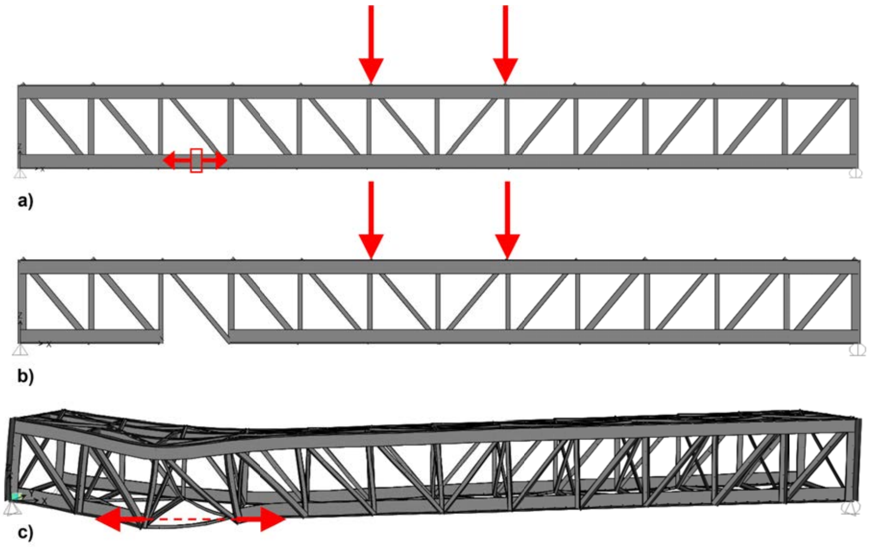

Fig. 82 | Equivalent incremental state after the removal of a chord segment: a) the internal forces carried by the member to be removed, b) the damage scenario, and c) the isolate effect of the member removal as the difference between b) and c).

This equivalent state represents, in simplified form, the changes that would occur in the bridge, whose effects, when compared with the results of the 48 DS evaluated (see figures from Fig. 85 to Fig. 94), are as follows:

1. The loss of continuity of the chord produces a substantial decrease in the bending stiffness of the structure. In the vicinity of the damaged panel, this stiffness is reduced to that of the chord opposite to the damaged one. The undamaged chord undergoes very significant and localised increments in bending within the vertical plane. The corresponding deformation resembles more of a rotational movement of each part of the structure on either side of the damage rather than a smooth deflection, as seen in a typical triangulated beam. **Each part of the structure rotates around a horizontal axis, passing through the joint directly above the removed chord segment** (indicated by the red dot in the front view in Fig. 83), almost acting as if hinged at that point. The load previously resisted by the damaged chord is transferred to the undamaged side, which causes both chords on the undamaged side to exhibit appreciable increments in deflection.

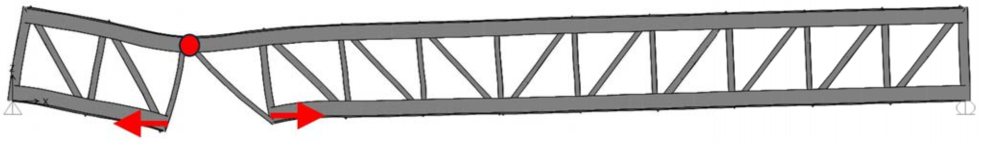

Fig. 83 | Front view showing hinge effect after chord removal.

2. Increments in bending also appear in the vertical plane of the stringers, particularly near the damaged area; however, these increments are smaller than those observed in the chords, as the stringers are much more flexible members (see Fig. 87). It is important to highlight that the deflections occurring in the verticals and diagonals adjacent to the damaged member are negligible, meaning that, in this case, one cannot speak of a Vierendeel effect in the lateral trusses.
3. The second effect of chord damage is **the global horizontal bending of the Pratt beam**. The deflection shape is strongly influenced by the reduction in global bending stiffness within the damaged panel. Although the hinge effect is less pronounced in the horizontal plane than in the vertical plane, the damaged panel still undergoes a significant opening. The global rotations in the horizontal plane led to localised bending of the chords near the damage. Overall, the lower and upper faces of the bridge act like beams in horizontal bending. As a result, the lower triangulation and the upper floor system become crucial in redistributing the failure. Specifically, in scenarios involving the failure of the upper chord, **the floor system is activated as a bending frame along the upper face of the bridge**, with bending increments in the horizontal plane of the transversal beams and stringers, which are not as localised as the bending of the chords (see Fig. 87).

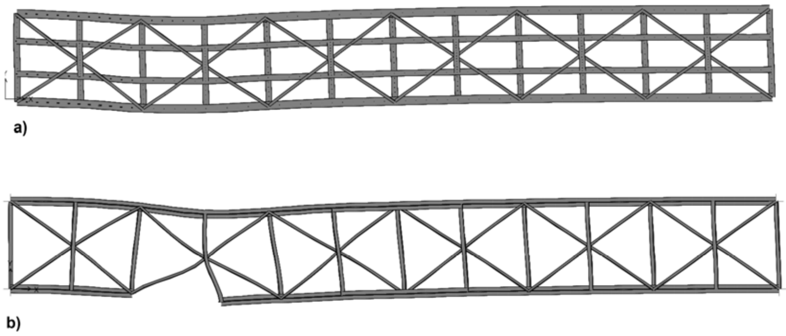

Fig. 84 | Bridge horizontal bending: a) Top level plan view, b) Bottom level plan view.

4. The transfer of the external load from the damaged side to the opposite side is clearly manifested through two mechanisms:
   a. An increment in **global torsional deformation** (i.e., considering the 3D component of the bridge) occurs **in the same direction on both sides of the damage**. On both sides, the Pratt beam sections increase their rotation in the same direction. As a result, the structural elements experience stress increments typical of the global torsional mode: axial forces increase or decrease (see Fig. 88), with these changes being widely distributed in the horizontal bracings, more concentrated in the chords, diagonals, and verticals, and very localised in the vertical bracings and transversal beams, which try to counteract the significant deformation near the damage. The fact that the torsion on both sides of the damage occurs in the same direction, rather than opposite directions, marks a key difference from the failure scenarios involving verticals and diagonals.
   b. Locally, the vertical bracing planes immediately adjacent to the damaged panel experience significant increments in axial force magnitude, especially considering they are small cross-section elements (see Fig. 88). This is due to the **localised distortion** of the Pratt beam sections in which these bracings are situated (the quadrilateral cross-sections distort into parallelograms), which is superimposed on the torsional mechanism. As mentioned in the previous point, the combined effect of local distortion and global torsion **transfers the external load from the damaged side to the opposite side**.

Chord loss ALP have the following similarities: the forces resisted by the lower (or upper) chord span that is removed are redistributed throughout the structure by (a) significant increments in vertical bending across all chords, with more pronounced effects in the upper (or lower, respectively) chord on the same side as the damage. These increments are highly localised within the two spans adjacent to the

junction where the diagonal and vertical members meet the removed segment; similar increments in vertical bending are observed in the stringers within the same area.; (b) increments in horizontal bending in all chords and a Vierendeel effect on the upper face of the bridge, more pronounced in upper chord failure scenarios; (c) changes in axial values in chords, verticals, diagonals, and bracing along the entire length of the bridge due to torsional patterns in the same direction on each side of the damage; (d) major, very large, changes in axial values in the upper face of the bridge due to torsional patterns in the same direction on each side of the damage; and (e) major, highly localised changes in axial forces in the vertical bracing planes and bending in the transversal beams next to the damaged vertical due to distortion of the bridge sections located on the sides of the damage.

Although all ALP resulting from the loss of successive chord sections exhibit the common pattern explained above, **there are differences between the ALP associated with damage to the upper chord compared to those of the lower chord**. On the one hand, the aforementioned horizontal truss effect of the floor system is much more pronounced in upper chord loss scenarios. On the other hand, in the upper chord damage zone, the stringers experience a significant increase in vertical plane bending, which leads to a considerably smaller increment in bending in the undamaged chord compared to the lower chord loss ALP.

There are also minor differences in the orientation of the reaction increments when odd or even segments of the bottom chord are removed. These differences are partly due to the combination of vertical deformation, horizontal deformation, and torsional mechanisms, and partly because the horizontal bracing spans the width of the bridge across two panels. As a result, the force redistribution pattern varies depending on the position of the removed chord segment relative to the adjacent horizontal bracing.

As with other failure scenarios, differences in the response magnitude arise primarily from how close or far the removed chord segment is from the nearest support. Before damage, the chords resist less axial force the closer they are to the supports, meaning the effect of their removal diminishes as it approaches the supports, becoming practically negligible when the damage occurs in the first or last module. In the following sections, we detail the effects observed in the different structural elements across these 48 DS. To do so, we employ heatmaps that illustrate changes in various KPIs, including displacements, bending moments, axial forces, and reactions.

2.4.1.1 Displacements

We begin by examining the effect of damage scenarios along the lower chord (Fig. 85). On the damaged side, all displacements along the remaining chord increase (i.e., negative values representing a downward movement). This increment intensifies from the nearest support towards the panel where the chord is removed and decreases from the damage towards the farthest support. The increment is much more pronounced in scenarios involving the removal of the central section.

On the side opposite to the damage and on the chord at the same level, the effect is similar: all displacements increase, although in absolute terms these changes are smaller (approximately 80%) compared to the displacements along the damaged chord.

The impact of these lower chord failure scenarios on the upper chords is very similar.

The pattern displayed by these results corroborates (1) the horizontal axis rotations around the joint near the damage (evident in the uniform progression of displacement increments on both sides of the damage in each scenario, with an abrupt change at the damage location), and (2) the torsional response mode in the same direction on both sides of the damage (visible in the differences in the magnitude of vertical displacement between corresponding points on the South and North sides).

It is striking to note the significant point differences in values between joints adjacent to the damage on the damaged side, very marked in some scenarios (dark or bright colours next to light or dull values on the same line). This localised increment of the vertical displacement between adjacent joints is a consequence of the distortion of the global sections next to the damage, which adds deformation to the global torsion effect.

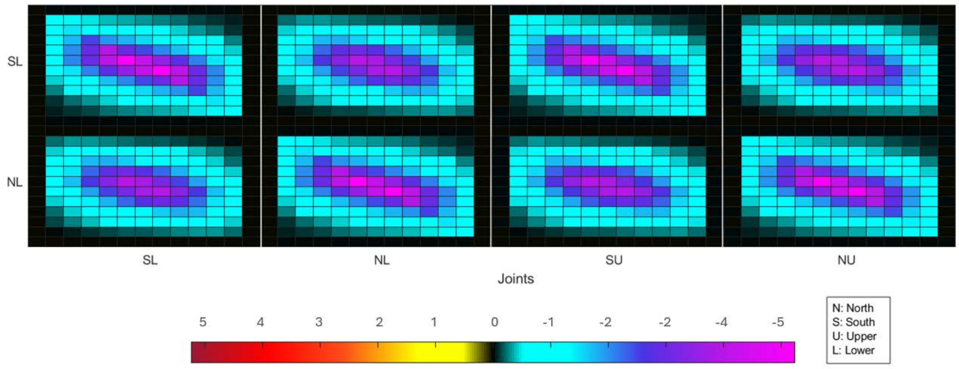

Fig. 85 | Absolute differences of vertical displacements in mm for the 24 lower chord segments' DSs between the damaged and undamaged states. The vertical axis shows the location of the removed lower chord segment, while the horizontal axis shows the joint on the bottom part of the bridge which the displacement is evaluated for each DS.

On the other hand, the upper chord failure scenarios show a similar, but much smoother pattern, due to the stringers coming into operation and having a distributing effect on load increments and deformations (see Fig. 86).

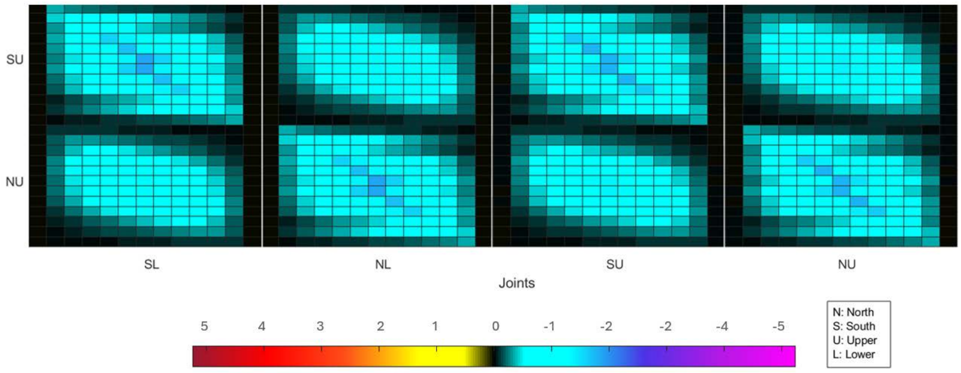

Fig. 86 | Absolute differences of vertical displacements in mm for the 24 upper chord segments' DSs between the damaged and undamaged states. The vertical axis shows the location of the removed lower chord segment, while the horizontal axis shows the joint on the bottom part of the bridge which the displacement is evaluated for each DS.

2.4.1.2 Bending Moments

The analysis (see Fig. 87) shows strong and very localised (deep red) increments in the magnitude of the moments in the chord opposite (above or below) the damaged chord, for all scenarios of this type. The strong increment is concentrated in the two chord sections reached by the vertical and the diagonal that bound the damaged segment, and the moment increments dissipate very quickly. This is characteristic of the mechanism of rotation of the two parts of the bridge around the joint near the damage, with significant moment increments. The increments are very small when the damage occurs near the supports and increase considerably when the damage occurs in the central span area. Along the damaged chord, the moments decrease.

On the undamaged side, the moments increment in both the upper and lower chords. The increments are very distributed and demonstrate that the loads have been transferred from the damaged side to the undamaged side.

It is worth noting the difference between (a) damage scenarios in the lower chord, which produce strong bending increments in the upper chord, fairly localised bending increments around the damage in both stringers, and very small bending increments in the transversal beams, and (b) damage scenarios in the upper chord, which follow an analogous pattern, but of much smaller magnitude in the chords and stringers, but show relevant bending increments in a significant group of transversal beams around the damage. The reason for this is that in the latter scenarios the set of stringers and floor beams are activated as a truss working in bending in the horizontal plane. The stringers also increase the bending in the

vertical plane close to the damage, and therefore the bending required to compensate for the loss of the upper chord is distributed between them and the bottom chord.

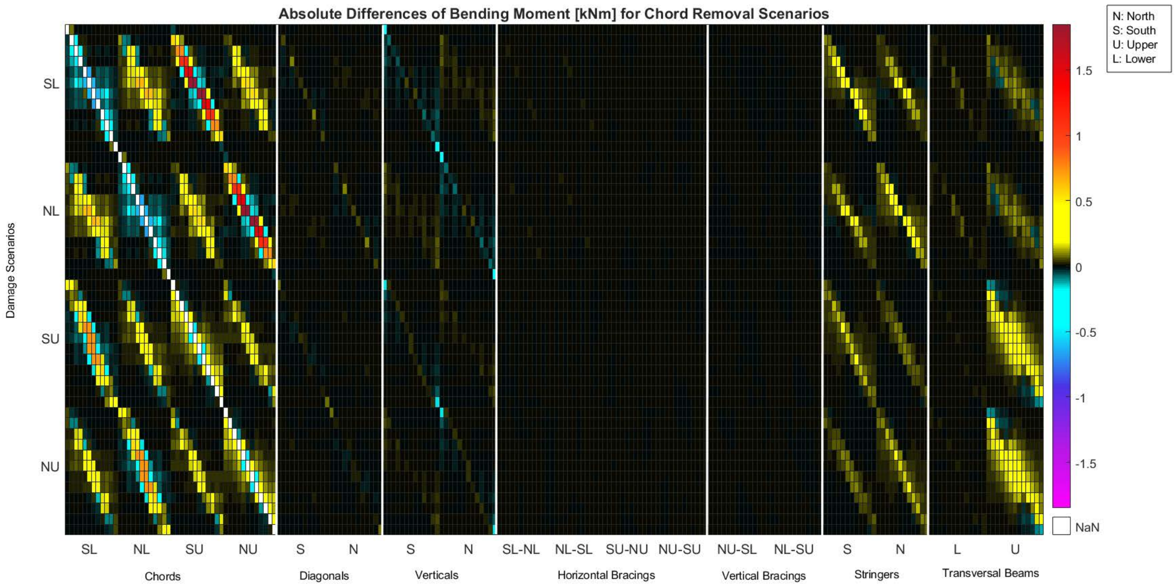

Fig. 87 | Absolute differences of bending moments in kNm for the 48 chord segments' DSs between the damaged and undamaged states. The vertical axis shows the location of the removed lower chord segment, while the horizontal axis shows the members for which the bending moment effect is evaluated for each DS.

The moment increments in the other elements are of much smaller magnitude, or practically zero in the case of the horizontal bracings. It is worth noting that, in scenarios of chord loss near the supports, the adjacent end vertical element experiences a moment increment (isolated blue dots in the diagram) due to the fact that the end vertical members are much more rigid compared to the other verticals.

Another very characteristic aspect of this group of DS, as already mentioned, is the development of truss action on the upper face formed by stringers and cross beams. This is particularly evident in the upper chord failure scenarios, where significant increments in bending are observed in a noticeable band of transversal beams around the damaged module. This framing action becomes very clear when observing, on the right side of Fig. 87, the moment increments in transversal beams and stringers in the upper chord failure scenarios (bottom) and comparing them with those in the lower chord failure scenarios (top).

2.4.1.3 Axial Forces

Fig. 88 shows the axial forces obtained for all 48 DS evaluated. The distribution of axial forces in the individual elements demonstrates the load transmission mechanism by torsion, with the same direction of torsion on both sides of the damage. This mechanism activates nearly all members of the bridge to varying degrees. We will primarily focus on the lower chord failure scenarios and briefly discuss the differences observed in the case of upper chord scenarios.

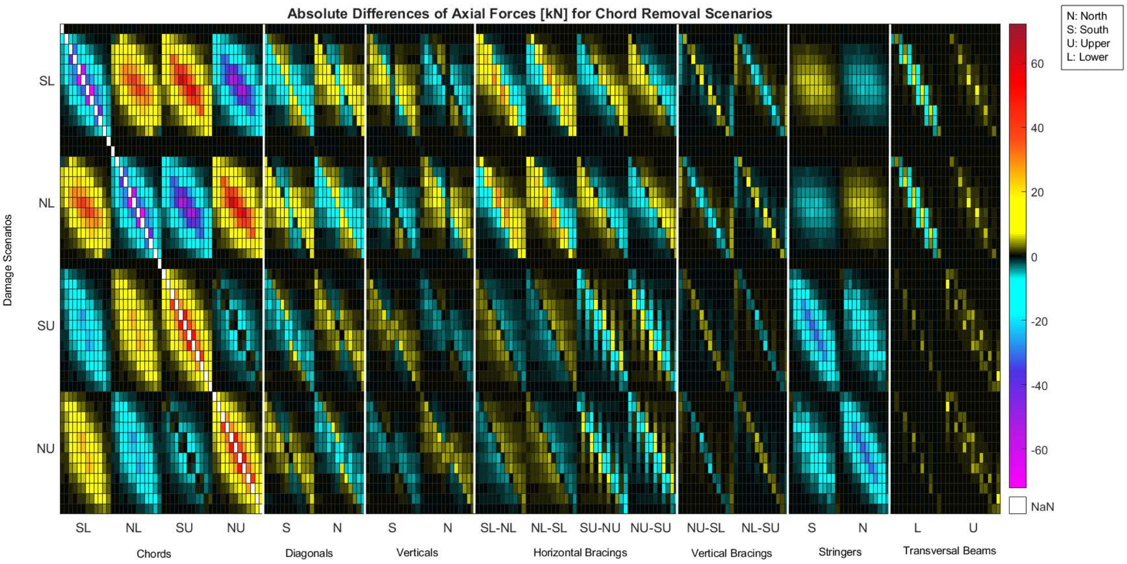

Fig. 88 | Absolute differences of axial forces in kN for the 48 chord segments' DSs between the damaged and undamaged states. The vertical axis shows the location of the removed lower chord segment, while the horizontal axis shows the members for which the axial force effect is evaluated for each DS.

Starting with the effect of lower chord damage on the **horizontal bracings** (see Fig. 89), it is notable that, within the same plane (upper or lower), the axial force increments exhibit an alternating sign pattern between the members in one direction compared to the members in the opposite direction. This pattern is, in turn, opposite to the alternating pattern in the other (lower or upper) plane. It is also remarkable that the trend of the axial force increment changes on either side of the panel where the damage occurs, within the same group of bars: an increase (or decrease) on one side corresponds to a decrease (or increase) on the opposite side. This behaviour is characteristic of the overall torsional effect in the same direction on each side of the damage. From this observation, it can be concluded that the structure exhibits global torsional behaviour in the same direction on both sides of the damage.

The axial force increments in the horizontal bracing caused by the failure of the upper chord (see Fig. 89) follow a similar pattern to those of the lower chord but with smaller magnitudes.

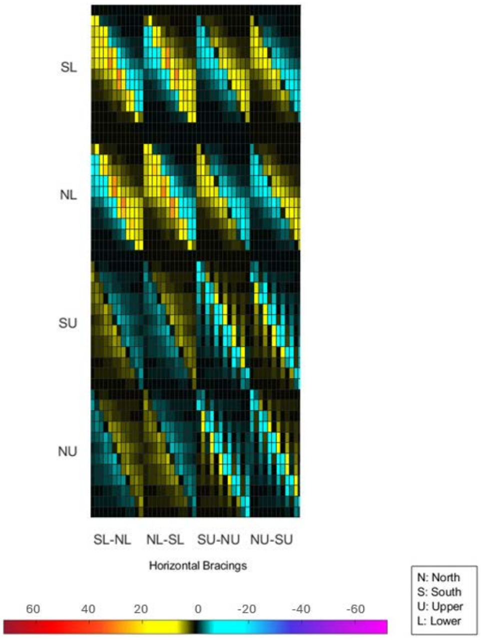

Fig. 89 | Absolute differences of axial forces in kN for chord segments' DSs between the damaged and undamaged states. The vertical axis shows the location of the removed chord segment, while the horizontal axis shows the horizontal bracing members for which the axial force effect is evaluated for each DS.

As for the diagonals (see Fig. 90), on the damaged side, we observe the following: from the nearest support to the damaged panel, the axial force on the diagonals decreases; from the damage point to the span centre, it increases; and finally, it decreases again towards the furthest support. When comparing this with the homologous diagonals on the undamaged side, we see that the behaviour is exactly the opposite of that on the damaged side, thus corroborating again the global torsional mechanism of the same sign on both sides of the damage. The change from increase to decrease (or vice versa) at the bridge's centre is due to the different orientation of the diagonals in each half.

The axial increments in the diagonals caused by the failure of the upper chord follow a similar pattern to those of the lower chord but are smaller in magnitude.

To understand the response of the verticals (see Fig. 90), it is helpful to examine them in conjunction with the diagonals, as they are part of the same resisting mechanism. On the left, we observe the axial increments in the diagonals, which have already been analysed, and on the right, the increments in the verticals. The patterns correspond inversely: on the damaged side, axial force increments occur between the nearest support and the damage, then decrements up to the span centre, followed by increments towards the farthest support. On the side opposite the damage, the opposite pattern is observed.

The axial force increments in the verticals caused by the failure of the upper chord follow a similar pattern to those of the lower chord but with smaller magnitudes.

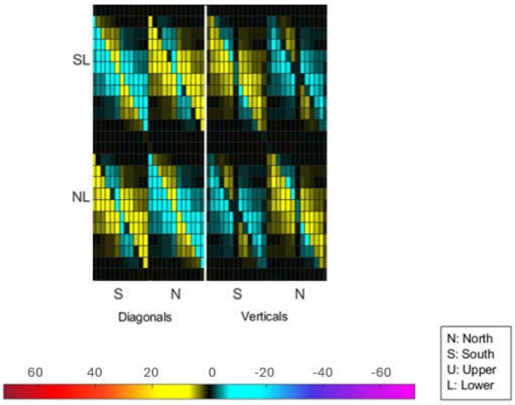

Fig. 90 | Absolute differences of axial forces in kN for the 24 lower chord segments' DSs between the damaged and undamaged states. The vertical axis shows the location of the removed lower chord segment, while the horizontal axis shows the diagonal and vertical members for which the axial force effect is evaluated for each DS.

As for the chords (see Fig. 91), the pattern is consistent with the torsional mechanism explained above: generalised axial force increments (or decrements), increasing towards the damaged side and decreasing away from it, with a very regular pattern. On the damaged side, axial forces decrease in the lower (damaged) chord and increase in the upper chord. On the undamaged side, the behaviour is the opposite, with smaller magnitudes. These changes are characteristic of the global torsional mode.

The axial force increments in the chords caused by the failure of the upper chord follow a similar pattern to those caused by the lower chord failure but with smaller magnitudes.

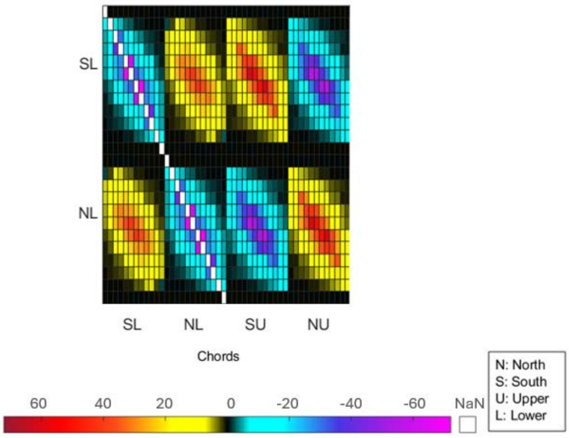

Fig. 91 | Absolute differences of axial forces in kN for the 24 lower chord segments' DSs between the damaged and undamaged states. The vertical axis shows the location of the removed lower chord segment, while the horizontal axis shows the chord members for which the axial force effect is evaluated for each DS.

The vertical bracings exhibit increments or decrements in axial force (of opposite magnitude and sign in each orientation) that are localised in one or two of the bracing planes adjacent to the damage, with smaller magnitude increments or decrements in the vertical bracings closest to the support nearer to the damage in all cases (see Fig. 92). The very localised changes close to the damage are the result of the activation of the local distortional mechanism of the bridge section, which, together with the damage mentioned at the beginning of this section. **This distortional pattern transfers forces from the damaged to the undamaged side of the bridge.**

The axial force increments in the vertical bracings produced by the failure of the upper chord follow a similar pattern to those made by the lower chord failure but with smaller magnitudes.

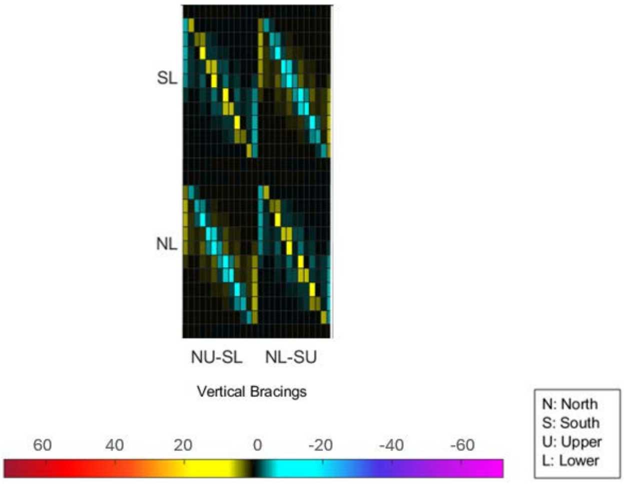

Fig. 92 | Absolute differences of axial forces in kN for the 24 lower chord segments' DSs between the damaged and undamaged states. The vertical axis shows the location of the removed lower chord segment, while the horizontal axis shows the vertical bracings for which the axial force effect is evaluated for each DS.

It remains to analyse the axial force increments corresponding to the lower chord failure scenarios **in stringers and transversal beams** (see Fig. 93). Starting with the stringers, we observe that along the one closest to the damaged side, the axial force increases. In contrast, along the far side, it decreases. This is consistent with the situation reflected in Fig. 82: the fictitious forces that produce the increments in axial force tend to induce lateral bending in the Pratt beam, lengthening the stringer on the damaged side and shortening the stringer on the side opposite the damage. The effect is distributed along the length of the beam. However, the axial force changes in transversal beams are much more localised,

with greater significance in the lower transversal beams near the damaged chord. These changes follow an increase-decrease-increase sequence in the latter.

In the case of stringers and transversal beams, differences are observed when the failure occurs in segments of the upper chord (Fig. 93). In this situation, the stringers experience significant decreases in axial force, with the largest magnitudes occurring next to the damaged chord (these "decreases" in axial force correspond to increments in compression). The axial force changes in the upper transversal beams are minimal, and in the lower transversal beams, they are negligible. In this case, the stringers contribute by absorbing a significant portion of the compression forces in the damaged chord.

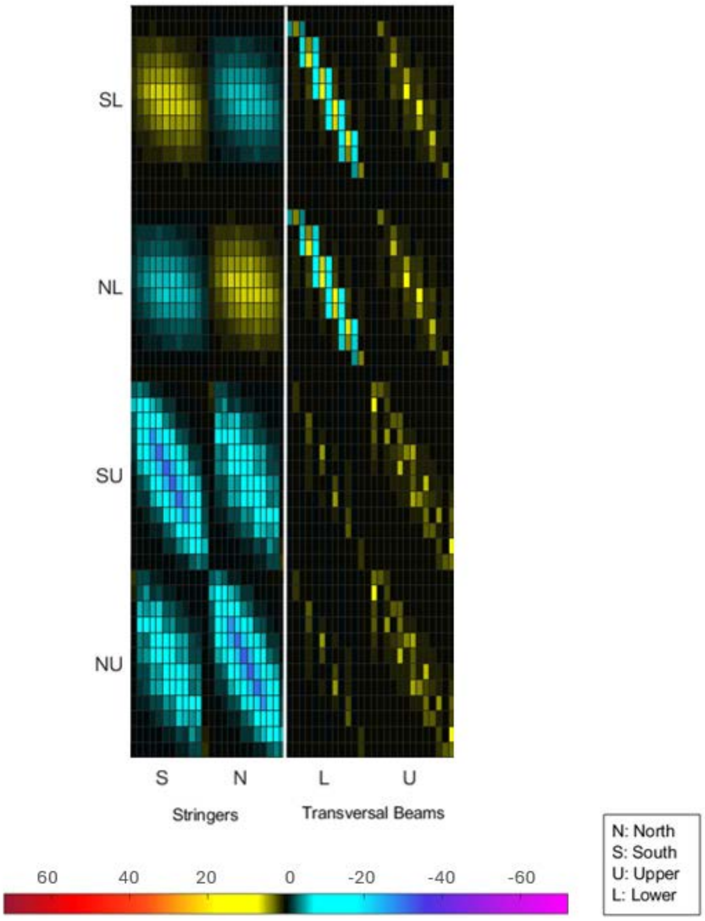

Fig. 93 | Absolute differences of axial forces in kN for chord segments' DSs between the damaged and undamaged states. The vertical axis shows the location of the removed chord segment, while the horizontal axis shows the stringer and transversal beam members for which the axial force effect is evaluated for each DS.

2.4.1.4 Reactions

The changes in the reactions are minor and, unlike other groups of ALP, more difficult to interpret (Fig. 94). This is because the four modes of deformation (horizontal axis rotations, horizontal bending, torsional deformation and transverse distortion) influence the magnitude of the reactions. The alternation or jumping between reaction values for consecutive segment removal scenarios is also due to the fact that the horizontal bracings span two modules. The consequence is that the forces in these bracings are redistributed along somewhat different paths depending on whether the removed segment is even or odd.

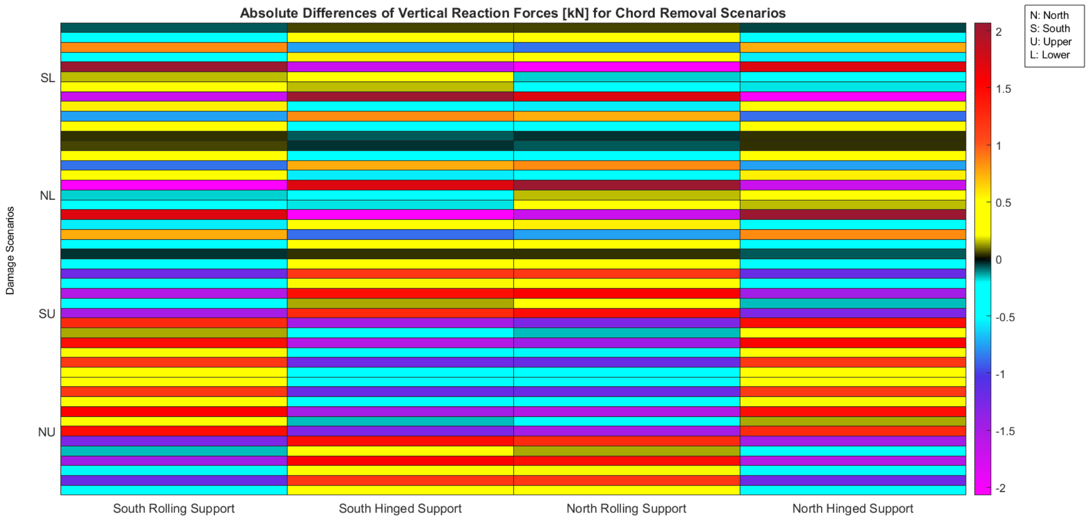

Fig. 94 **| Absolute differences of vertical reactions in kN for the 24 lower and upper chord segments' DSs between the damaged and undamaged states. The vertical axis shows the location of the removed chord segment, while the horizontal axis shows the support location for which the vertical reaction effect is evaluated for each DS.**

2.4.1.5 Validation of the experimental campaign and conclusions

The analysis conducted in this section has confirmed the results obtained from the experimental campaign, as summarised in Section 1. Fig. 95 presents the numerical results for the DS that coincide with the scenario tested in the experimental campaign. This three-dimensional representation of the bridge illustrates the increase or decrease in demand for the elements considering only the absolute values of the damaged and undamaged states, i.e., the absolute differences in axial force and bending moment for each element. The consequences of the previously described behaviours are clearly visible: the rotations around the joint near the damage and the horizontal bending are well reflected in the moment increments, while the torsion on both sides of the damage aligns with the generalised occurrence of axial forces, demonstrating a clear load transfer from the damaged to the undamaged sections of the structure.

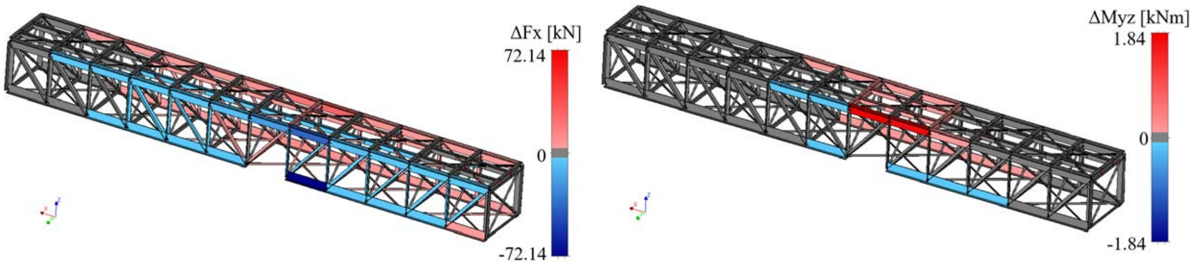

Fig. 95 | 3D view of the bridge representing absolute differences of axial forces [kN] and bending moments [kNm] for the lower chord DS carried out in the experimental campaign between the damaged and undamaged states.

In addition, the understanding of the activated bridge defence mechanisms in real loading situations has been further enhanced after evaluating a total of 48 different DSs of removal of different chord spans. The removal of a chord segment causes a combination of several phenomena: (1) a global horizontal axis rotation of each part of the structure around the joint close to the damage, (2) horizontal bending of the whole structure, and (3) a load transfer mechanism from one side to the other that manifests itself in (3-a) torsion in the same direction in the whole bridge, together with (3-b) an appreciable distortion of the sections immediately adjacent to the damage. It has been determined that the bending in the vertical plane of the chords at the level opposite to the damage, and also of the stringers (to a greater extent when the damage occurs in the upper chord), has very significant importance in this first defence mechanism, presenting notable increases in bending moments. They are, therefore, critical elements in securing this

first line of defence of the bridge. On the other hand, and especially in scenarios of damage to the upper chord, a second defence mechanism is activated: bending in the horizontal plane, which translates into a truss effect on the upper face of the bridge in the vicinity of the damaged panel. Additionally, the vertical bracings near the damaged area, along with the horizontal bracings across the entire bridge, play a crucial role in transmitting loads from the damaged to the undamaged parts. These elements contribute significantly to the activation of the (3a) defence mechanism: a global torsional effect in the same direction throughout the bridge, which leads to changes in axial forces in the verticals, diagonals, chords, and horizontal bracings. The combination of these load transfer mechanisms is critical. Spatial rotations foster bending in both the vertical and horizontal planes within the damaged area, while the torsional response demonstrates the three-dimensional nature of the system. This characteristic is vital for distributing the load of the removed chord across the entire structure, preventing the stress from being borne solely by the elements immediately adjacent to the DS.

2.4.2 Diagonal Removal Scenarios

This section presents a discussion of these findings, including a critical analysis of the shared and case-specific structural patterns observed. The aim is to identify the principal alternative load paths (ALPs) or defence mechanisms that the bridge uses in response to the failure of any of its diagonals when subjected to load levels comparable to the actual railway traffic loads considered.

At a global level, increments in internal forces, as well as the variations in displacements and reactions resulting from the removal of a diagonal, can be estimated by applying an equivalent state to the structure (Fig. 96.a-c). This approach involves imposing forces equal in magnitude but opposite in direction (Fig. 96.c) to the axial force carried by the diagonal prior to its removal (Fig. 96.a) at the nodes where the diagonal was connected.

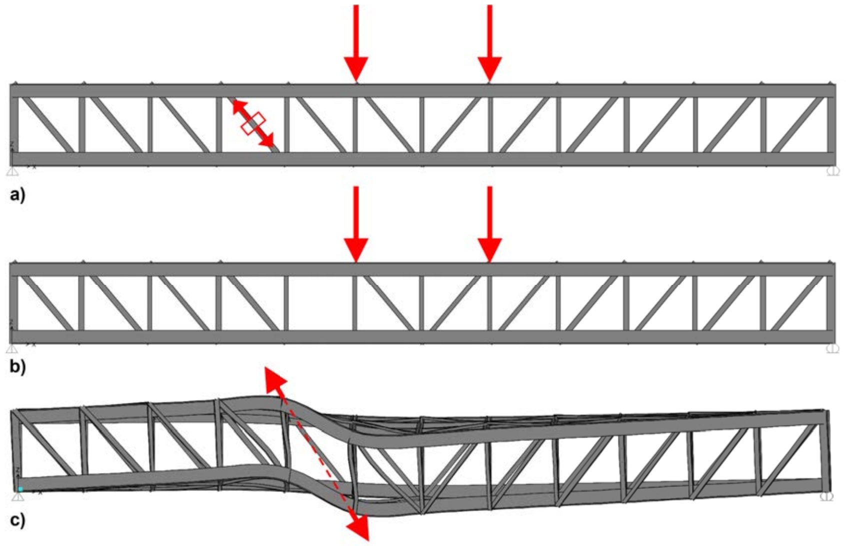

Fig. 96 | Equivalent incremental state after a diagonal removal: a) the internal forces carried out by the member to be removed, b) the damage scenario, and c) the isolate effect of the member removal as the difference between b) and c).

This equivalent state represents a simplified model of the bridge's behaviour. When contrasted with the results of the 24 DSs evaluated (see Fig. 97 to Fig. 104), the following effects can be observed:

1. The panel where the diagonal is removed undergoes significant distortion. A Vierendeel-type mechanism is activated, causing a substantial increase in bending in the upper and lower chords on the damaged side. Bending moments also occur in the vertical members that border the panel, although to a lesser extent, as these members are much more flexible (see Fig. 98).
2. The significant distortion of the damaged panel induces an increase in global torsional deformation (i.e. considering the 3D behaviour of the bridge), with opposite rotational directions on each side of the damage. On one side, the Pratt truss sections experience increased rotation in one direction, while on the opposite side, they rotate in the other. Consequently, the structural

elements undergo stress changes characteristic of this global torsional mode: increases or decreases in axial forces (Fig. 99) in the verticals, diagonals, horizontal bracings, and chords, with the direction of force change depending on the orientation of each member.

3. At a local level, only the vertical bracing planes between the damaged panel and the nearest support experience significant increments in axial forces, while the transversal beams adjacent to the damaged panel exhibit increments in both axial forces and moments (see Fig. 98 and Fig. 99). These elements contribute to redistributing the external load from the damaged side to the opposite side of the structure.

All ALP activated due to the loss of a diagonal follow a consistent pattern: the axial force previously resisted by the diagonal is redistributed throughout the structure by (a) highly localised increments in bending in the chords and verticals of the damaged panel and adjacent ones, (b) changes in axial force values in chords, verticals, diagonals, and bracing elements along the entire length of the bridge, driven by opposite torsional effects on either side of the damage, and (c) very localised, significant changes in axial forces in the vertical bracing and bending moments in transversal beams adjacent to the damaged diagonal.

The differences between the ALPs in this group of 24 DSs are minimal. These variations are primarily attributed to the proximity of the removed diagonal to the nearest support and the fact that the central diagonals, located between the sections where the loads are applied, carry very little axial force before the removal. Consequently, the impact of their removal is much less significant.

In the following section, we provide a detailed explanation of the effects observed in the various structural elements resulting from this set of 24 DSs. To illustrate these effects, we refer to the heatmaps that depict changes in key performance indicators (KPIs), including displacements, bending moments, axial forces, and reactions.

2.4.2.1 Displacements

On the damaged side, from the nearest support to the panel where the diagonal has been removed, vertical displacements decrease (i.e. positive values, indicating upward movement). Conversely, from the damaged panel towards the farthest support, vertical displacements increase (i.e. negative values, indicating downward movement). The most significant decreases in displacement occur when the removed diagonal is located closer to the support.

On the opposite side of the damage, the downward vertical displacement increases across a large portion of the span. Between the damaged zone and the nearest support, there is minimal movement, while the displacement values remain fairly uniform across the rest of the span.

Considering the differences in values between the damaged and undamaged sides, it becomes apparent that the cross sections of the Pratt beam rotate in one direction from the nearest support towards the damaged side and in the opposite direction from the damaged side towards the farthest support, as illustrated in simplified form in Fig. 96.

This pattern confirms the two primary structural response modes described earlier: (1) alternating vertical displacement increases on both sides of the damaged panel, driven by the Vierendeel mechanism, and (2) rotations along the global longitudinal axis, resulting from global torsional deformations in opposing directions.

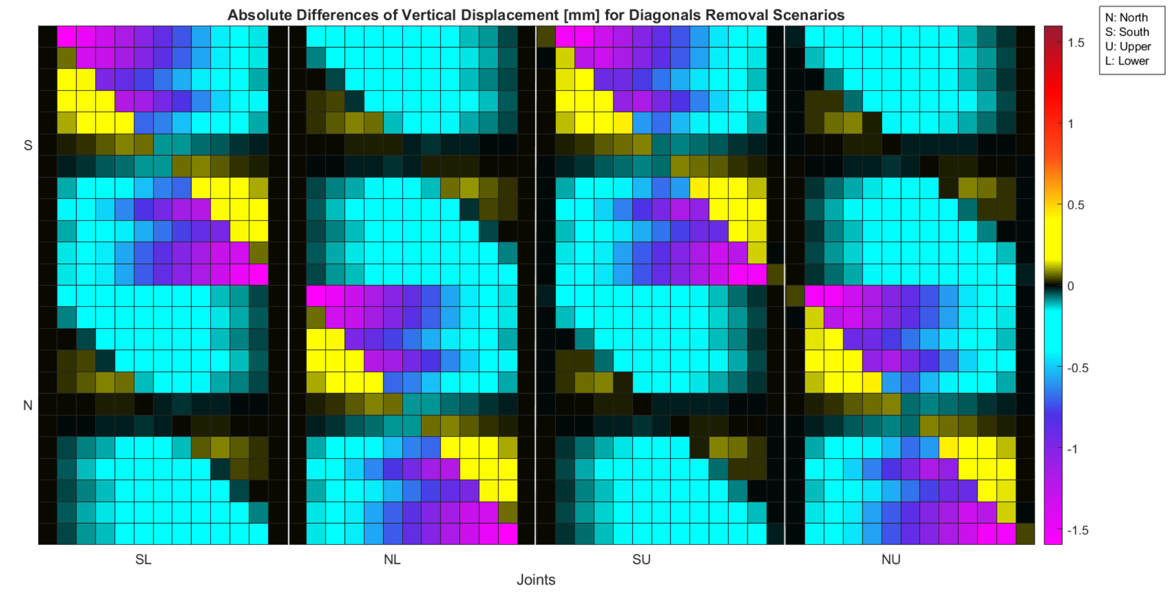

Fig. 97 | Absolute differences of vertical displacements in mm for the 24 diagonals DSs between the damaged and undamaged states. The vertical axis shows the location of the removed diagonal, while the horizontal axis shows the joint on the bottom part of the bridge, where the displacement is evaluated for each DS.

2.4.2.2 Bending Moments

The analysis reveals highly localised increments in the magnitude of moments in both the upper and lower chords on the side of the removed diagonal, specifically within the damaged panel and the adjacent panels, across all damage scenarios of this type. These moment increments dissipate quickly, being primarily concentrated in four chord segments. On the undamaged side, there are also moment increments in the upper and lower chords, but these are significantly smaller by an order of magnitude.

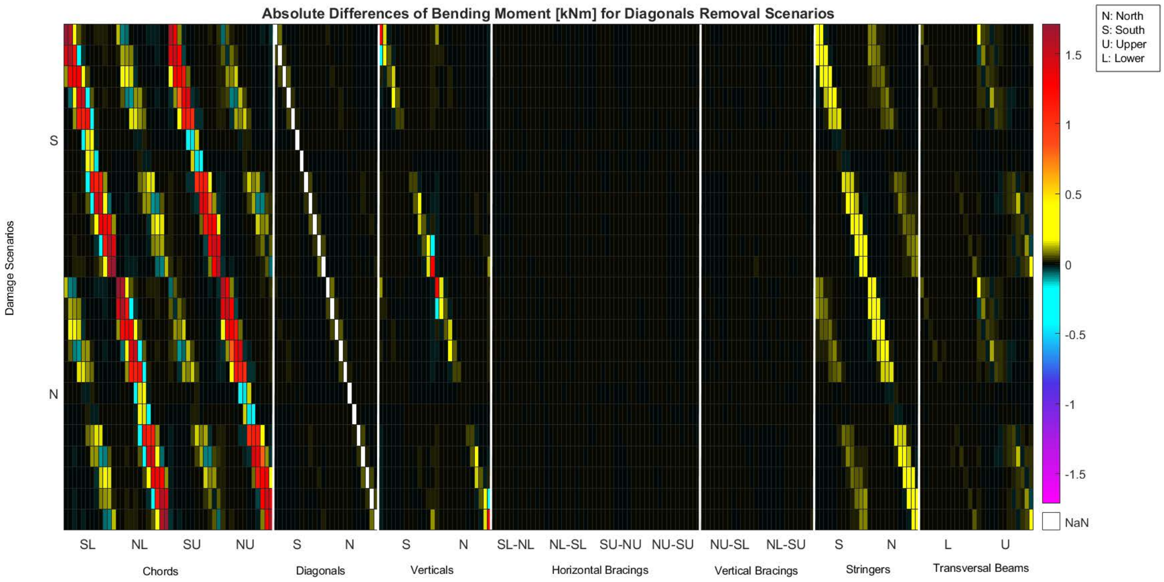

Fig. 98 | Absolute differences of bending moments in kNm for the 24 diagonals DSs between the damaged and undamaged states. The vertical axis shows the location of the removed diagonal, while the horizontal axis shows the members for which the bending moment effect is evaluated for each DS.

The moment increments in the verticals are concentrated in the elements immediately adjacent to the damaged panel. They are significantly higher at the end verticals (shown by red colour values) due to their increased stiffness in the bridge under analysis. The moments are notably smaller when the

damaged diagonal is located in the central part of the span, between the load application points, where, theoretically, no global shear forces act on the bridge.

As for the rest of the elements, only the stringers near the damaged side exhibit relevant moment increments (although significantly smaller than those in the chords), as they absorb part of the Vierendeel action. This effect also extends to the transversal beams adjacent to the removed diagonal.

2.4.2.3 Axial Forces

Fig. 99 shows the axial forces obtained for all the 24 DS evaluated.

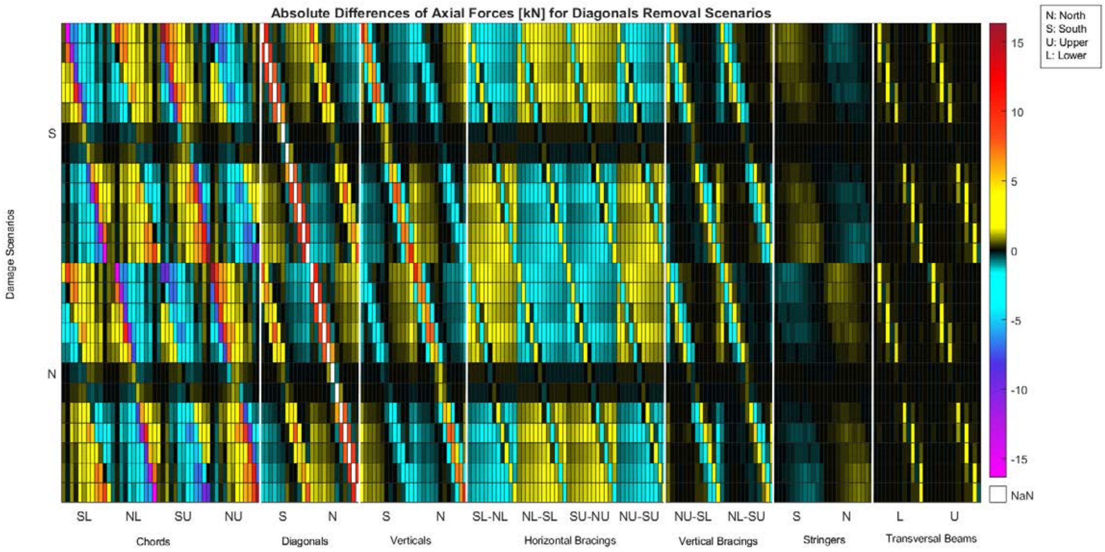

Fig. 99 | Absolute differences of axial forces in kN for the 24 diagonals DSs between the damaged and undamaged states. The vertical axis shows the location of the removed diagonal, while the horizontal axis shows the members for which the axial force effect is evaluated for each DS.

The distribution of axial forces in the different elements demonstrates the mechanism of load transmission by torsion acting in opposite directions on both sides of the damaged area. **Starting with the horizontal bracings** (see Fig. 100), it is noteworthy that, within the same plane (top or bottom), the axial force increments show an alternating pattern of opposite signs between the members oriented in one direction and those oriented in the other. This alternating pattern is, in turn, mirrored in the other plane (lower or upper). Additionally, in the panel containing the damaged diagonal, there is a disturbance in the axial forces; however, outside this zone, the direction of the force increment remains consistent within each family of bars aligned in the same direction. This behaviour is characteristic of torsion acting in opposite directions on either side of the damage. From these observations, it can be inferred that the structure exhibits a global torsional response with opposing directions of torsion on both sides of the damaged zone.

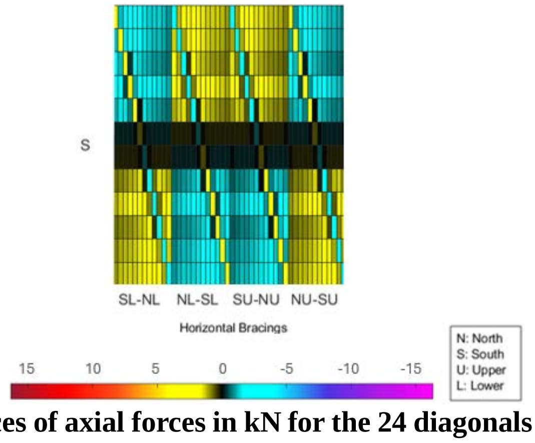

Fig. 100 | Absolute differences of axial forces in kN for the 24 diagonals DSs between the damaged and undamaged states. The vertical axis shows the location of the removed diagonal only on the South side, while the horizontal axis shows the horizontal bracing members for which the axial force effect is evaluated for each DS.

Regarding the diagonals (excluding those belonging to the damaged scenario (DS); see Fig. 101), on the damaged side, the diagonals immediately adjacent to the removed one experience significant increments in axial forces. In the half of the span containing the damaged panel, axial forces either decrease or remain almost unchanged, while in the opposite half, they show a slight increment. This change of sign is due to the diagonals having opposite orientations in each half of the bridge, combined with the effect of torsion acting in opposite directions on either side of the damage. When comparing this behaviour with the corresponding diagonals on the undamaged side, we observe exactly the opposite pattern. This further confirms the global torsional mechanism, with opposite signs across the structure.

To understand the response of the **verticals**, it is useful to analyse them alongside the diagonals, as both are integral to the same resistance mechanism (Fig. 101). On the left, we observe the axial force increments in the diagonals of the southern half-span, which have already been analysed, while on the right, the axial force increments in the verticals are shown. The correspondence between the patterns is clear: highly localised axial force increments occur in the verticals adjacent to the removed diagonal, and increments of opposite signs are observed between homologous verticals on the damaged and undamaged sides.

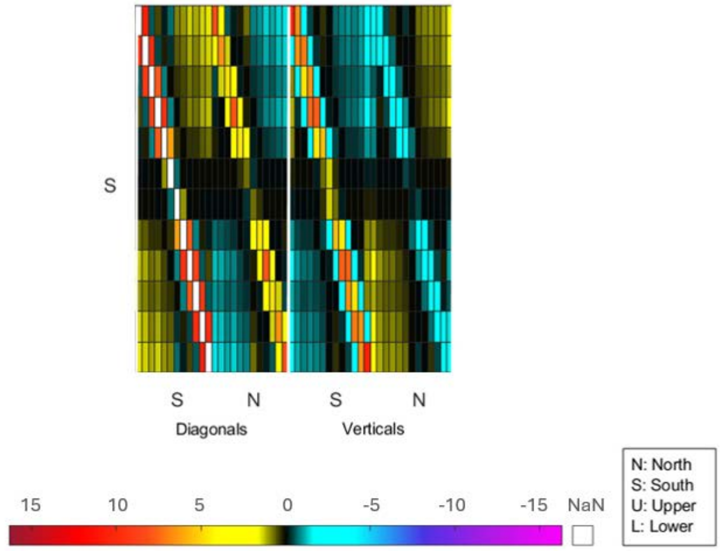

Fig. 101 | Absolute differences of axial forces in kN for diagonals DSs between the damaged and undamaged states. The vertical axis shows the location of the removed diagonal only on the South side, while the horizontal axis shows the diagonal and vertical members for which the axial force effect is evaluated for each DS.

Regarding the chords (see Fig. 102), the pattern is entirely consistent with the torsional mechanism previously described and mirrors the behaviour of the verticals and diagonals: significant axial force increments are observed in the chord sections connected to the removed diagonal. On the damaged side, in the lower chord, axial force increments extend towards the nearest support, while axial force decreases towards the farthest support. In contrast, the upper chord on the damaged side exhibits the opposite behaviour. Similarly, in both chords on the undamaged side, the pattern is reversed compared to the damaged side.

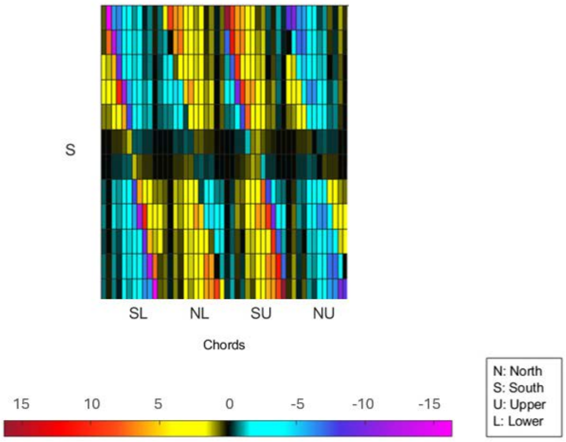

Fig. 102 | Absolute differences of axial forces in kN for the 24 diagonals DSs between the damaged and undamaged states. The vertical axis shows the location of the removed diagonal only on the South side, while the horizontal axis shows the chord members for which the axial force effect is evaluated for each DS.

It is also important to highlight the localised effects on the **vertical bracings and transversal beams** (see Fig. 103), which demonstrate the significance of the load transfer mechanism from the removed diagonal to the surrounding structure, particularly in the region immediately adjacent to the damage. For transversal beams, the increment in axial forces is highly localised, while for vertical bracings, the impact is more pronounced, with these elements becoming activated between the damaged panel and the nearest support.

Lastly, **stringers** (see Fig. 103) play a minimal role in the torsional mechanism linked to the loss-of-diagonal ALP, as the changes in their axial forces are relatively insignificant.

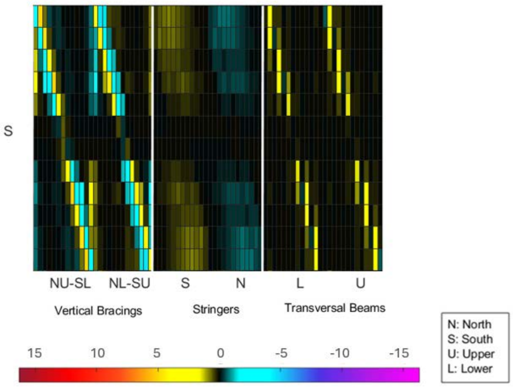

Fig. 103 | Absolute differences of axial forces in kN for the 24 diagonals DSs between the damaged and undamaged states. The vertical axis shows the location of the removed diagonal only on the South side, while the horizontal axis shows the vertical bracing, stringer and transversal beam members for which the axial force effect is evaluated for each DS.

2.4.2.4 Reactions

Increments in reactions at the four bridge supports confirm the activation of the torsional mechanism in the ALP (see Fig. 104). For all diagonals within the same half of the bridge and the same side, the direction of the reaction increments at each support remains consistent, although the values fluctuate. This distribution is characteristic of the torsional mode in opposite directions on either side of the damage. The torsional rotations exhibit opposite signs on each side of the damage, with an additional change in sign occurring when diagonals are withdrawn on the East side, compared to the West side, due to the different diagonal orientations. These combined changes in sign—both to the left and right of the damage and between the East and West sides of the bridge—lead to the results shown in Fig. 104. The total applied load on the bridge is 80 kN, distributed evenly across the four supports at 20 kN each.

The measured reaction changes are only around 2 kN (10%), further demonstrating the significance of the diaphragm-like action[10–13] of the vertical bracing in the vertical plane of the supports (see Fig. 89).

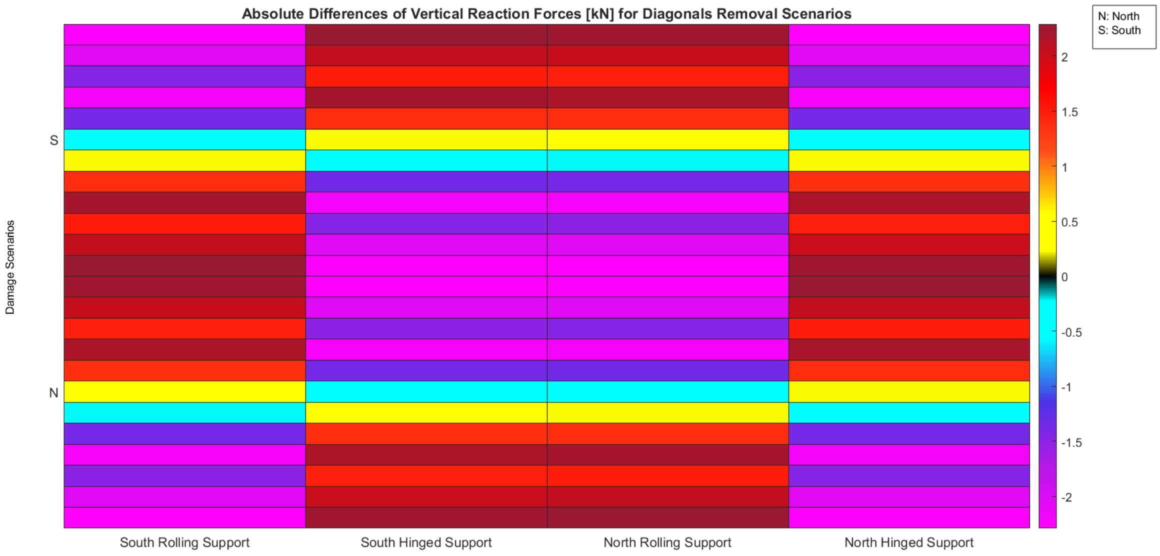

Fig. 104 | Absolute differences of vertical reactions in kN for the 24 diagonals DSs between the damaged and undamaged states. The vertical axis shows the location of the removed diagonal, while the horizontal axis shows the support location for which the vertical reaction effect is evaluated for each DS.

2.4.2.5 Validation of the experimental campaign and conclusions

The analysis conducted in this section corroborates the findings from the experimental campaign, as summarised in Section 1. Fig. 105 illustrates the numerical results of the DS that match the conditions of the experimental campaign. This three-dimensional representation of the bridge illustrates the increase or decrease in demand for the elements considering only the absolute values of the damaged and undamaged states, i.e., the absolute differences in axial force and bending moment for each element. The two key behaviours identified earlier are clearly observable: (1) the Vierendeel mechanism, characterised by the emergence of significant bending moments in the vicinity of the damaged area, and (2) the global torsional mechanism, evident through the development of axial forces across a broader portion of the structure, indicating a clear load transfer from the damaged region to the undamaged part.

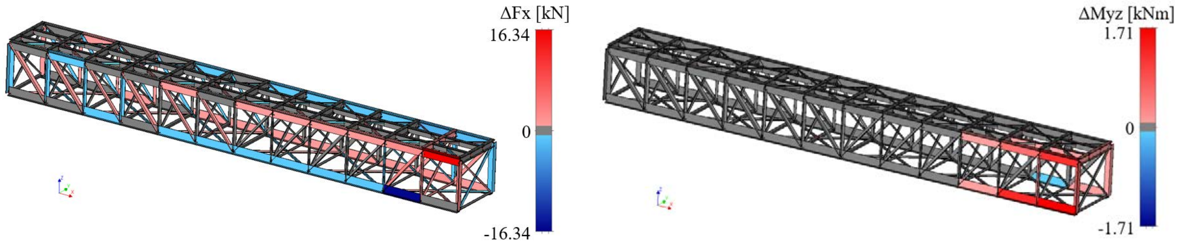

Fig. 105 | 3D view of the bridge representing absolute differences of axial forces [kN] and bending moments [kNm] for the diagonal DS carried out in the experimental campaign between the damaged and undamaged states.

Additionally, the evaluation of 24 different DSs involving diagonal removal has enhanced the understanding of the bridge's defence mechanisms under real loading conditions. The removal of a diagonal triggers a dual response: a Vierendeel-type behaviour near the removed diagonal and a torsional response throughout the entire bridge. In the first case, chords, diagonals, and verticals in the vicinity of the damage play a crucial role in this primary defence mechanism, exhibiting significant increments in both axial forces and bending moments. These elements are, therefore, critical in reinforcing the bridge's first line of defence. For the second behaviour, the vertical bracings, along with the transversal beams, have played a significant role in transferring the load from the damaged part to the undamaged part. This

load transfer, carried out through these vertical planes and assisted by the horizontal bracings, has resulted in the global torsions of opposite signs on either side of the damaged area, as previously described. These torsional effects have led to notable twists and the generalised increment of axial forces in other elements of the bridge. The combination of both load transfer mechanisms is crucial: the Vierendeel mechanism is localised to the damaged area, while the torsional mechanism reflects the activation of the system's three-dimensional behaviour. This is essential for redistributing the load from the removed diagonal across the entire structure, preventing it from being resisted solely by the elements near the damage scenario.

2.4.3 Vertical Removal Scenarios

This section presents a discussion of these findings, including a critical analysis of the shared and case-specific structural patterns observed. The aim is to identify the principal alternative load paths (ALPs) or defence mechanisms that the bridge uses in response to the failure of any of its verticals when subjected to load levels comparable to the actual railway traffic loads considered.

At a global level, the increments in internal forces, as well as the variations in displacements and reactions resulting from the removal of a vertical, can be estimated by applying an equivalent state to the structure (Fig. 106.a-c). In this equivalent state, forces equal and opposite (Fig. 106.c) to the axial force carried by the vertical before its removal (Fig. 106.a) are imposed on the nodes where the vertical was originally attached.

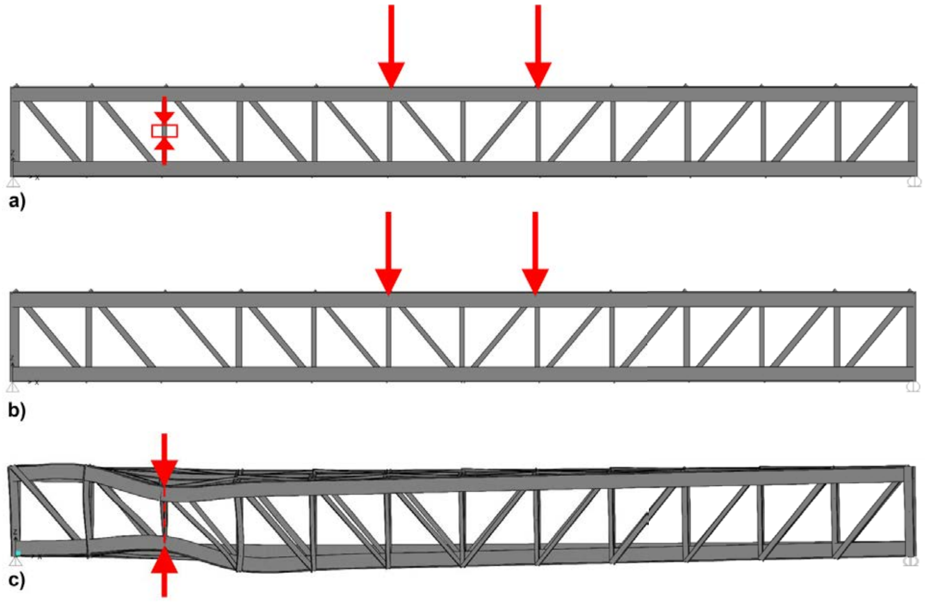

Fig. 106 | Equivalent incremental state after a diagonal removal: a) the internal forces carried out by the member to be removed, b) the damage scenario, and c) the isolate effect of the member removal as the difference between b) and c).

This equivalent state represents, in a simplified form, the changes that would occur in the bridge, the effects of which, when contrasted with the results of the 26 DS evaluated (see figures from Fig. 107 to Fig. 113), are as follows:

1. The panel where the vertical is removed undergoes significant distortion. **A local Vierendeel-type mechanism is activated**, leading to a substantial increment in the bending of the upper and lower chords on the damaged side. Bending also occurs in the stringers near the damaged area, although to a lesser extent due to their higher flexibility (see Fig. 108). It is important to note that the deflections observed in the verticals and diagonals near the damage are negligible.
2. The significant distortion of the damaged panel induces increments in **overall torsional deformation** (i.e., considering the 3D behaviour of the bridge) with opposite directions on either side of the damage: on one side, the Pratt beam sections rotate in one direction, while on the other side, they rotate in the opposite direction. As a result, the structural elements experience force increments typical of this torsional mode: axial force variations (see Fig. 109)

occur in a generalised manner in the horizontal bracings, are more concentrated in the chords, diagonals, and verticals, and are very localised in the vertical bracings and transversal beams (which resist the large torsional twists near the damage). In this case, the axial increments in the stringers are negligible.

3. At a local level, the vertical bracing planes between the damaged panel and the nearest support exhibit higher magnitude increments in axial forces compared to those further away from the nearest support. The transversal beams at the position of the removed vertical also experience larger increments in axial forces (see Fig. 108 and Fig. 109). Both element types play a key role in **transferring the external load from the damaged side to the opposite side**.

All ALP mechanisms activated by the loss of a vertical follow the same pattern: the axial force previously resisted by the vertical is redistributed throughout the structure via (a) very localised increments in bending in the chords and stringers of the damaged panel, (b) changes in the axial forces in chords, verticals, diagonals, and bracings along the entire length of the bridge due to opposing torsional patterns on each side of the damage, and (c) significant, highly localised changes in axial forces in the vertical bracing and bending planes of transversal beams adjacent to the damaged vertical.

The differences between the ALPs in this group of 26 DS are very small. They primarily depend on how close or far the removed vertical is from the nearest support and the fact that the central vertical, positioned between the load application sections, experiences very little axial force before the damage, so its removal has a much smaller effect. It's also worth noting that scenarios, where extreme verticals are removed, are particularly aggressive, although they follow similar patterns to those described. In these cases, the only differences are changes in axial forces in elements even far from the damaged area (i.e. the torsional effect is stronger in these cases) and much larger changes in bending moments (i.e. the Vierendeel-type effect is increased).

2.4.3.1 Displacements

On the damaged side, from the nearest support to the panel where the vertical is removed, the vertical displacement decreases (i.e. positive value, representing an upward movement; see Fig. 107). From the damaged side to the farthest support, the vertical displacement increases (i.e. negative values, representing a downward movement). The largest decreases occur in scenarios where the removed vertical coincides with the support.

On the opposite side of the damage, the downward vertical displacement increases across a large part of the span: between the damage zone and the nearest support, there is hardly any movement, while the values remain uniform over the rest of the span.

Considering the differences in values between the damaged and undamaged sides, it follows that the cross-sections of the Pratt beam rotate in one direction from one support to the damage and in the opposite direction from the damage to the farthest support, as depicted in simplified form in Fig. 106.

This pattern supports the two main structural response modes explained above: (1) alternating vertical displacement increments on both sides of the damaged panel due to the Vierendeel mechanism, and (2) global longitudinal axis rotations due to global torsional deformations of opposite sign.

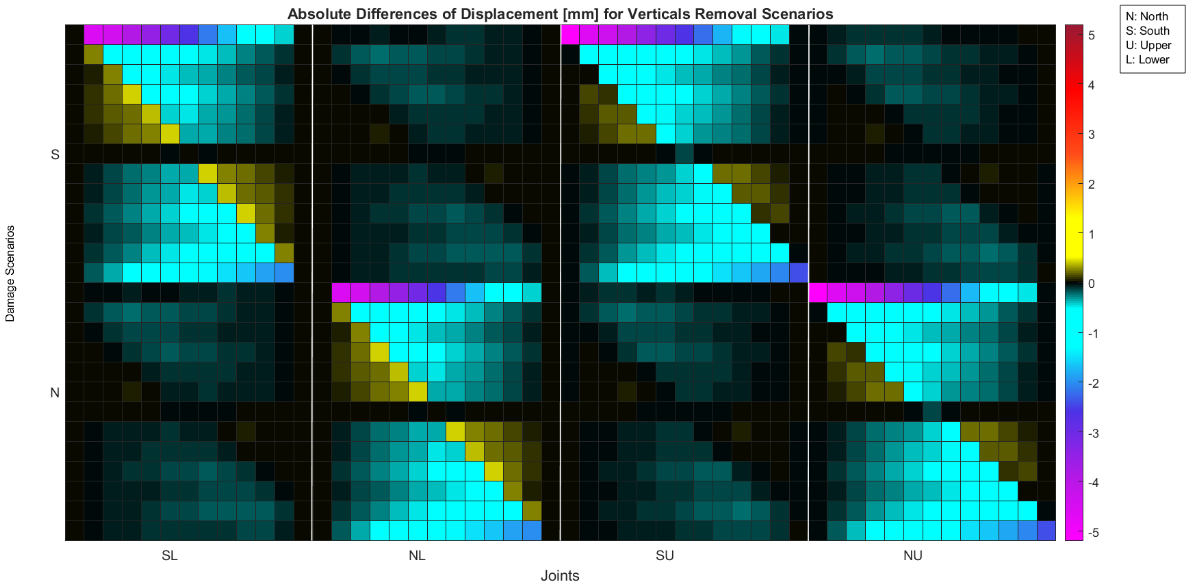

Fig. 107 | Absolute differences of vertical displacements in mm for the 26 verticals DSs between the damaged and undamaged states. The vertical axis shows the location of the removed vertical, while the horizontal axis shows the joint on the bottom part of the bridge, where the displacement is evaluated for each DS.

2.4.3.2 Bending Moments

The analysis (see Fig. 108) shows very localised increments in the magnitude of the moments in both the upper and lower chords on the side of the removed vertical in the area of the damaged panels for all damage scenarios of this type. The moment increments dissipate rapidly and are concentrated mainly in four chord segments. It should be noted that the removal of verticals has a major effect when they are aligned with the position of the supports, which places high demands on the bending capacity of the chords close to this position. On the undamaged side, there are also increments in the upper and lower chords, but they are an order of magnitude smaller. The moments are negligible when the damaged vertical is in the central part of the span, located between the load application positions, where, theoretically, there are no global shear forces acting on the bridge.

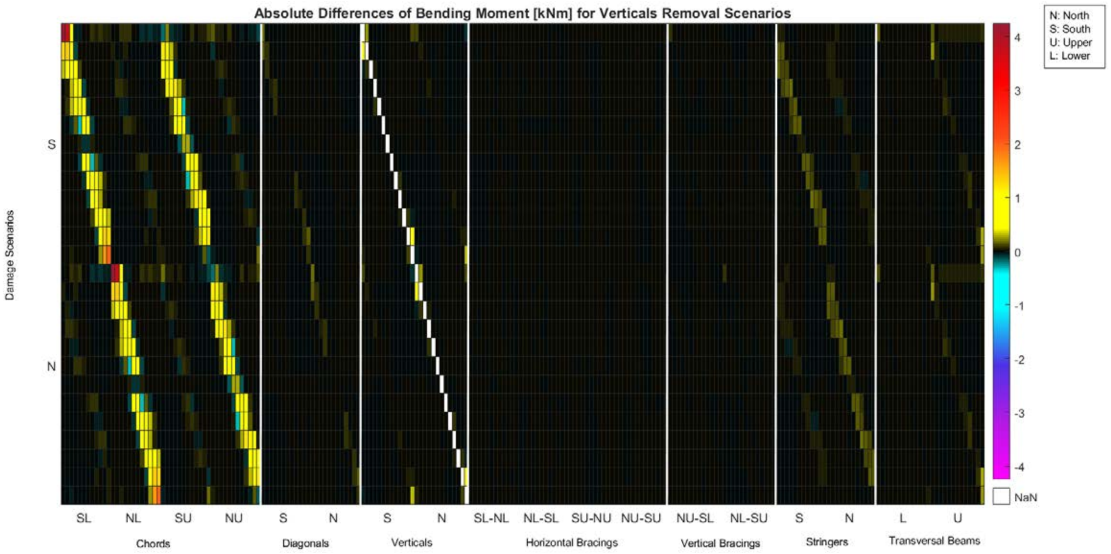

Fig. 108 | Absolute differences of bending moments in kNm for the 26 verticals DSs between the damaged and undamaged states. The vertical axis shows the location of the removed vertical, while the horizontal axis shows the members for which the bending moment effect is evaluated for each DS.

The moment increments in the rest of the elements are of lesser magnitude. It is perhaps worth highlighting those observed in the stringers, which, in this case, respond similarly to the upper chords and absorb part of the Vierendeel action, although to a lesser extent than the chords.

2.4.3.3 Axial Forces

Fig. 109 shows the axial forces obtained for all 26 DS evaluated.

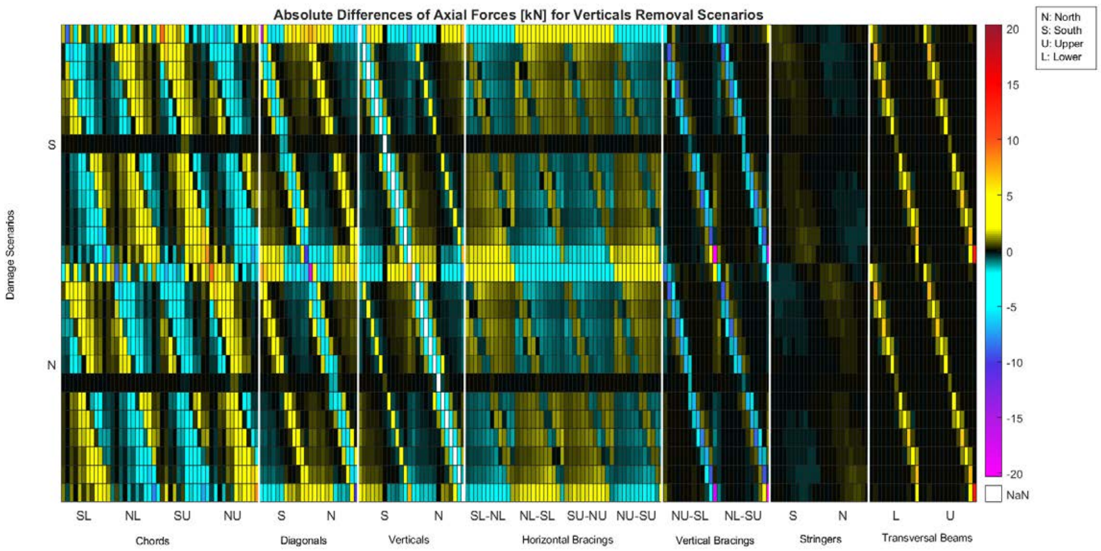

Fig. 109 | Absolute differences of axial forces in kN for the 26 verticals DSs between the damaged and undamaged states. The vertical axis shows the location of the removed vertical, while the horizontal axis shows the members for which the axial force effect is evaluated for each DS.

The distribution of axial forces in the different elements demonstrates the mechanism of load transmission by torsion in opposite directions on both sides of the damage.

Starting with the horizontal bracing (see Fig. 110), it is striking that, within the same plane (upper or lower), the axial increments present a pattern of alternating signs between the members in one direction and those in the opposite direction. This pattern is, in turn, opposite to the alternating pattern in the other (lower or upper) plane. It is also notable that in the panels corresponding to the damaged vertical, there is a disturbance in the axial forces. Still, outside this zone, the increment sign remains consistent within each set of bars going in the same direction. This is characteristic of torsion in opposite directions on each damaged side. From this observation, it can be inferred that the structure exhibits a global torsion behaviour in opposite directions on each side of the damage.

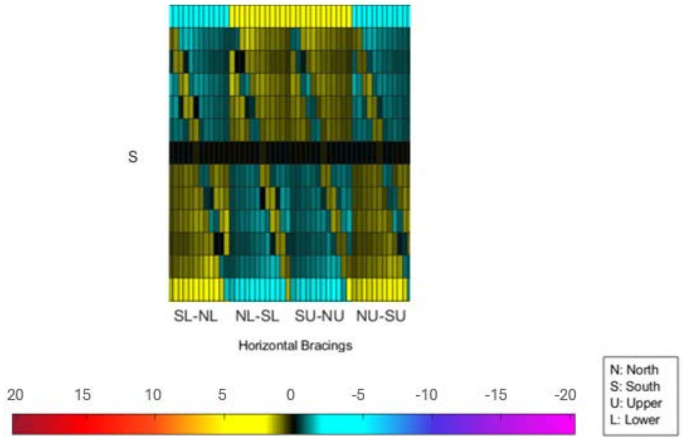

Fig. 110 | Absolute differences of axial forces in kN for the 26 verticals DSs between the damaged and undamaged states. The vertical axis shows the location of the removed vertical only on the South side, while the horizontal axis shows the horizontal bracing members for which the axial force effect is evaluated for each DS.

As for the diagonals (see Fig. 111), on the damaged side, we observe that the diagonals immediately adjacent to the removed vertical experience significant axial force increments. Comparing these with the corresponding diagonals on the undamaged side (right side of the diagram in Fig. 87), we see that the behaviour is exactly the opposite on the undamaged side, thus further confirming the global torsional mechanisms with opposite signs on each side of the damage. Once again, it is important to emphasise the significant influence of the damage scenarios (DS) involving vertical removal at the support positions. In these cases, the changes in axial forces are substantial, significantly activating the torsional behaviour of the bridge and affecting the entire span in a generalised manner.

To understand the response of the verticals (other than the removed one; see Fig. 111), it is useful to examine them alongside the response of the diagonals, as they are part of the same resisting mechanism. On the left, we observe the axial increments in the diagonals, which we have already analysed and on the right, the increments in the verticals. The correspondence between the patterns is clear: very localised axial increments in the verticals adjacent to the removed one and increments of opposite sign between corresponding verticals on the damaged and undamaged sides.

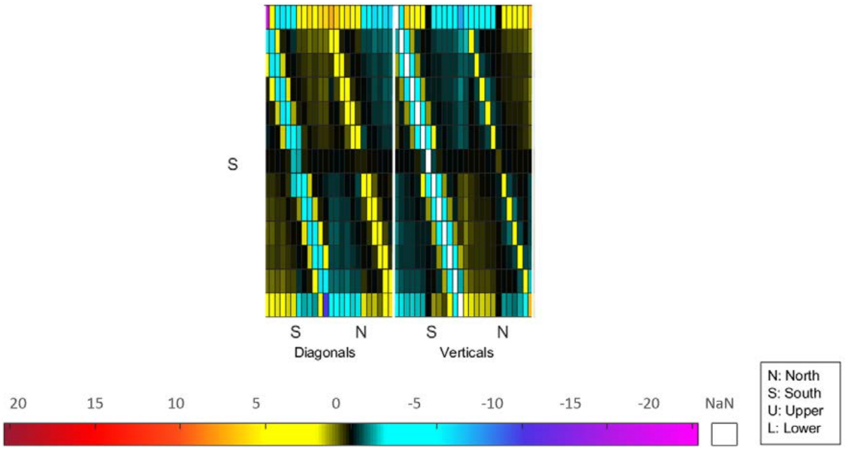

Fig. 111 | Absolute differences of axial forces in kN for the 26 verticals DSs between the damaged and undamaged states. The vertical axis shows the location of the removed vertical only on the South side, while the horizontal axis shows the diagonal and vertical members for which the axial force effect is evaluated for each DS.

As for the chords (see Fig. 112), the pattern is fully consistent with the torsional mechanism explained above: axial force increases in a generalised manner, which dissipates in areas farther from the damage. On the damaged side and in the lower chord, there are axial force increments up to the nearest support, followed by axial force decreases towards the farthest support. The opposite behaviour is observed in the upper chord on the damaged side. Both chords display behaviour opposite to the damaged sides on the undamaged side. Once again, the observations regarding DS related to the loss of the vertical located in the centre of the span or at the supports are applicable.

It is also important to mention the localised effect of **vertical bracings and transversal beams** (see Fig. 112), which highlights the significance of the localised load transfer mechanism from the removed vertical to the rest of the structure (mainly to the undamaged part) in the area immediately adjacent to the damage. In the case of axial forces in transversal beams, the effect is very localised. This is less pronounced for vertical bracings, which are activated to a greater extent towards the nearest support.

Finally, **stringers** (see Fig. 112) are minimally involved in the torsional mechanism associated with loss-of-vertical ALPs, as the modification of their axial forces is of little relevance.

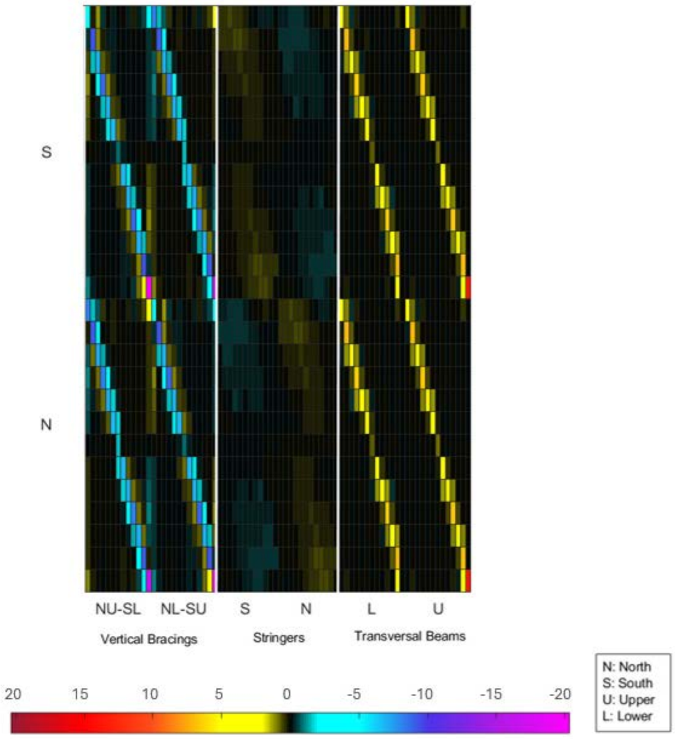

Fig. 112 | Absolute differences of axial forces in kN for the 26 verticals DSs between the damaged and undamaged states. The vertical axis shows the location of the removed vertical only on the South side, while the horizontal axis shows the vertical bracing, stringer and transversal beam members for which the axial force effect is evaluated for each DS.

2.4.3.4 Reactions

The increments in the reactions at the four supports of the bridge confirm the activation of the torsional mechanism of the ALP (see Fig. 113). For all DSs on the same half of the bridge and on the same side, the direction of the increments at each support is consistent. The distribution follows the characteristic pattern of the opposite torsional mode on each side of the damage. The rotations of this torsional mode are of opposite signs on each side of the damage, with the sign changing again at the removal of verticals on the East side compared to the West side due to the different orientation of the diagonals. The combination of both sign changes, to the left and right of the damage and across the West and East sides of the bridge, leads to the results shown in Fig. 90. The total load applied to the bridge, as mentioned earlier, is 80 kN, distributed evenly among the four supports, resulting in 20 kN per support. The measured changes are only 2 kN (10%), which also underscores the importance of the diaphragm-type behaviour[10–13] of the vertical bracing in the vertical plane corresponding to the supports (see Fig. 89). As noted before, the observations on DSs related to the loss of the vertical located at the centre of the span or at the supports remain applicable. Finally, it is worth highlighting the significant reaction imbalance when the vertical aligned with the same support is removed, where the difference increases from just 2 kN (for other DSs) to more than 8 kN (40%).

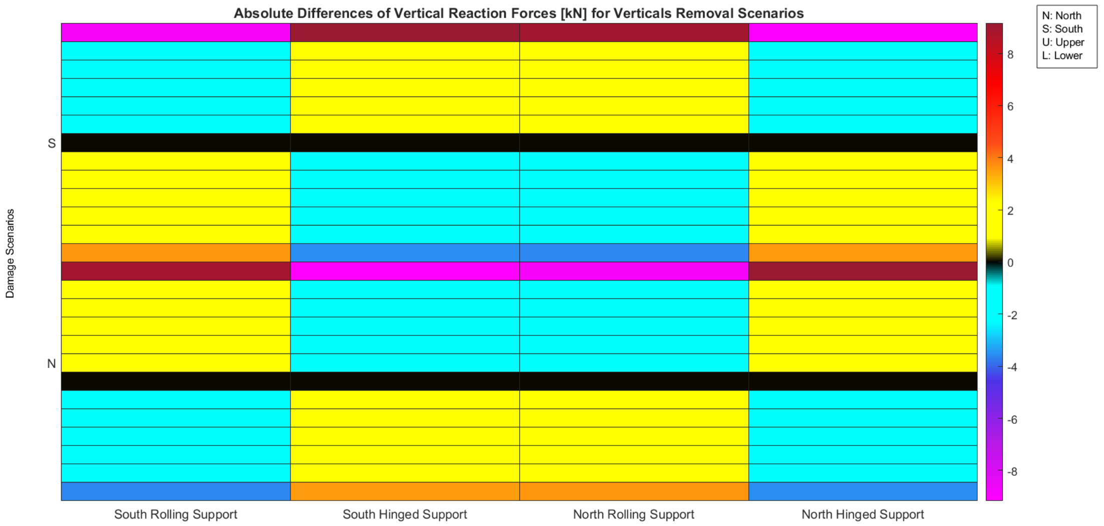

Fig. 113 **| Absolute differences of vertical reactions in kN for the 26 verticals DS between the damaged and undamaged states. The vertical axis shows the location of the removed vertical, while the horizontal axis shows the support location for which the vertical reaction effect is evaluated for each DS.**

2.4.3.5 Validation of the experimental campaign and conclusions

The analysis conducted in this section has confirmed the results obtained in the experimental campaign, summarised in Section 1. Fig. 114 presents the numerical results of the DS, which align with those obtained in the experimental campaign. This three-dimensional representation of the bridge illustrates the increase or decrease in demand for the elements considering only the absolute values of the damaged and undamaged states, i.e., the absolute differences in axial force and bending moment for each element. The two behaviours described earlier are clearly visible: the Vierendeel-type behaviour, characterised by significant bending moments in the vicinity of the damage, and the global torsional behaviour, where axial forces appear in a more widespread manner, demonstrating a clear load transfer from the damaged part to the undamaged part.

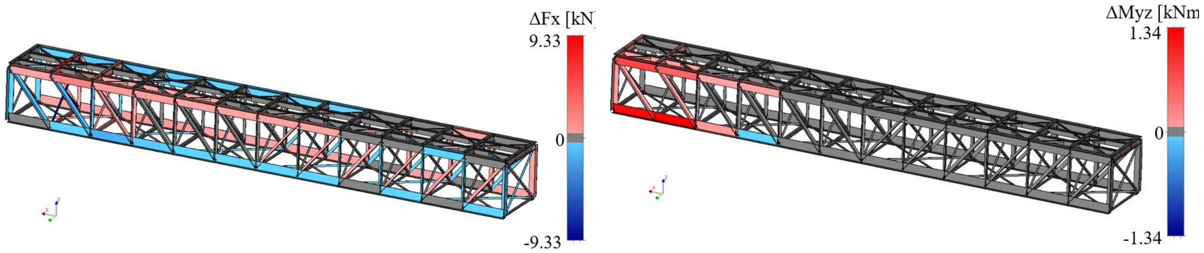

Fig. 114 | 3D view of the bridge representing absolute differences of axial forces [kN] and bending moments [kNm] for the vertical DS carried out in the experimental campaign between the damaged and undamaged states.

In addition, the understanding of the activated bridge defence mechanisms in real loading situations has been enhanced by evaluating a total of 26 different DS for the removal of various verticals. The removal of a vertical results in a dual phenomenon: on the one hand, a Vierendeel-like behaviour in the vicinity of the removed vertical, and on the other, a torsional behaviour of the bridge as a whole. In the first case, it has been determined that the chords, and to a lesser extent the stringers, close to the damage scenario, play a crucial role in this primary defence mechanism, exhibiting notable increments in bending moments. These elements are, therefore, critical in maintaining the bridge's structural integrity and securing its first line of defence.

2.4.4 Other Damage Scenarios

This section provides a concise discussion of these results, focusing on displacements, bending moments, axial forces, and reactions, along with validating the experimental results presented in Section 1 and a summary of the key conclusions.

2.4.4.1 Displacements

After the removal of any of these elements, the maximum displacements observed were all below 0.1 mm, except for the case of the stringer removals, where maximum displacements reached 0.2 mm. These minimal values confirm that these DS had minimal impact on the overall response of the bridge. In the case of the stringers, the effect was more pronounced due to the loss of the bridge's resistant section within the floor system at the level of the upper chords.

2.4.4.2 Bending Moments

As with other DS, the analysis reveals highly localised increments in the magnitude of the moments. The results displayed in Fig. 115 to Fig. 118 clearly demonstrate this effect while also showing very low quantitative values, consistently below 0.15 kNm, which is clearly an order of magnitude smaller than those observed in other damage scenarios.

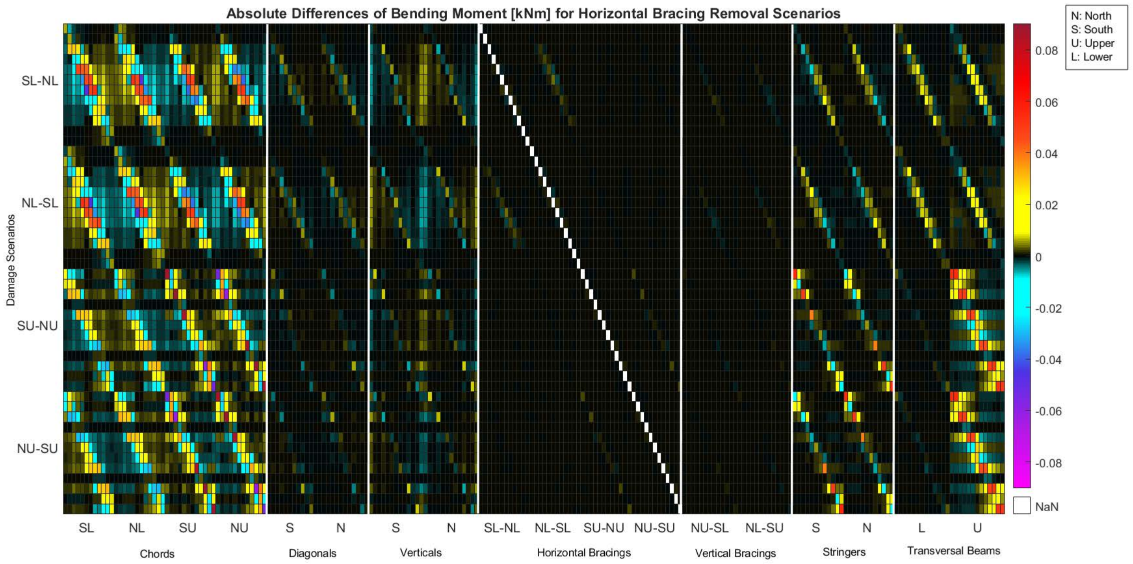

Fig. 115 | Absolute differences of bending moments in kNm for the 48-horizontal bracing DSs between the damaged and undamaged states. The vertical axis shows the location of the removed horizontal bracing, while the horizontal axis shows the members for which the bending moment effect is evaluated for each DS.

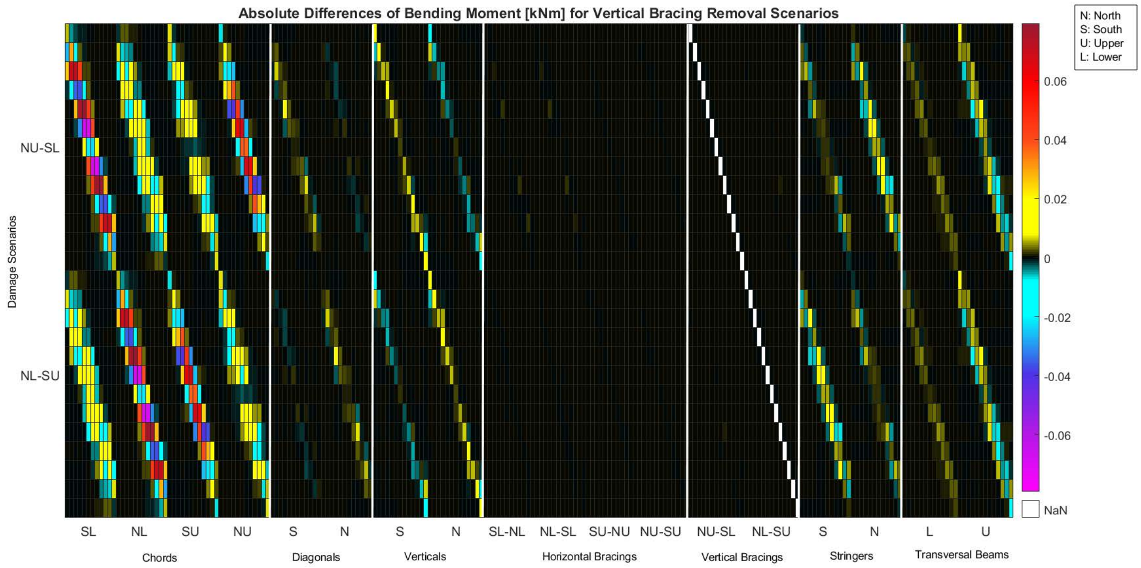

Fig. 116 | Absolute differences of bending moments in kNm for the 26 vertical bracing DSs between the damaged and undamaged states. The vertical axis shows the location of the removed vertical bracing, while the horizontal axis shows the members for which the bending moment effect is evaluated for each DS.

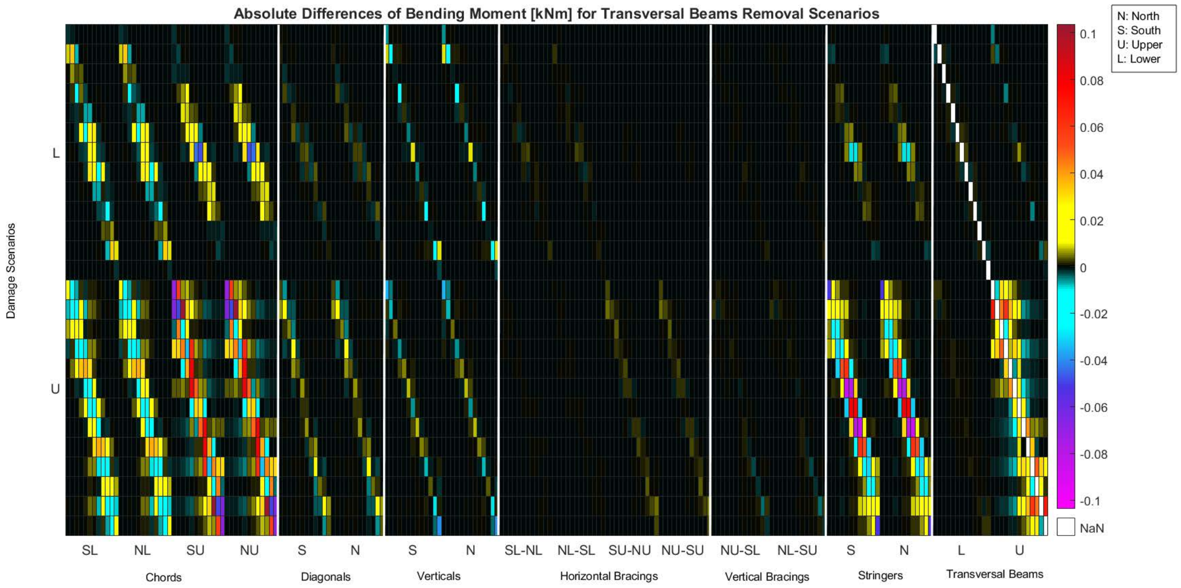

Fig. 117 | Absolute differences of bending moments in kNm for the 26 transversal beams DSs between the damaged and undamaged states. The vertical axis shows the location of the removed transversal beam, while the horizontal axis shows the members for which the bending moment effect is evaluated for each DS.

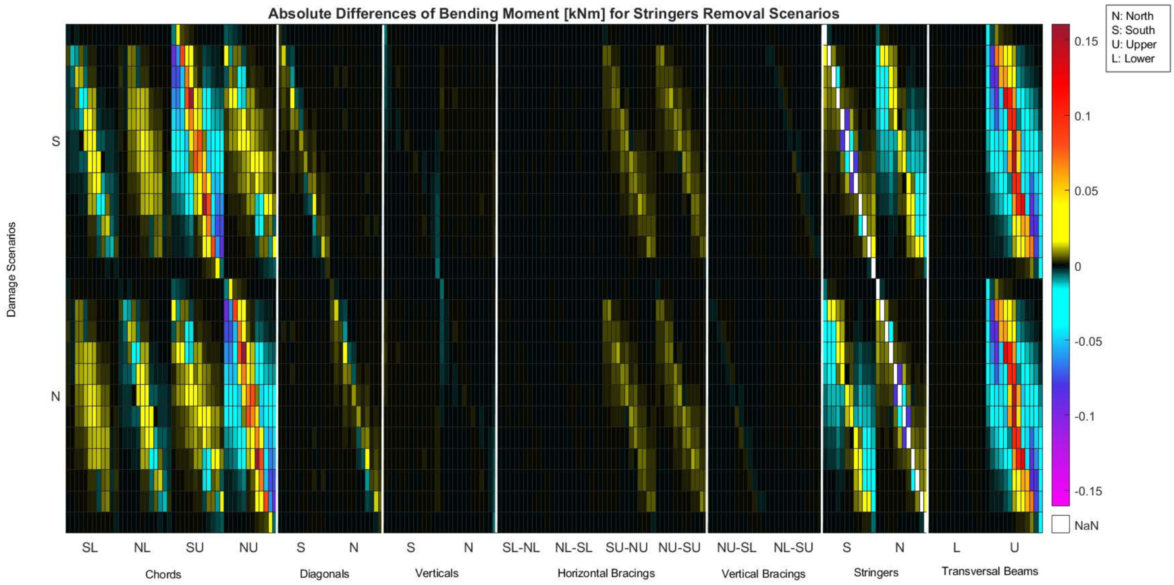

Fig. 118 | Absolute differences of bending moments in kNm for the 24 stringers DSs between the damaged and undamaged states. The vertical axis shows the location of the removed stringer, while the horizontal axis shows the members for which the bending moment effect is evaluated for each DS.

2.4.4.3 Axial Forces

The results shown in Fig. 119 to Fig. 122 highlight significant differences in the behaviour of different DSs:

- *Horizontal bracings* (Fig. 119): The recorded values are of little significance. Only the DSs involving the lower horizontal bracing show appreciable axial force increments in the nearest lower chords.
- *Vertical bracing* (Fig. 120): The recorded values are also of minimal importance. Axial force increments are only observed in the part of the vertical bracing that remains near the DS.
- *Transversal beams* (Fig. 121): The lower transversal beam DSs have negligible effects on the structure. However, damage to the upper transversal beams has a more pronounced impact on elements in the upper part of the bridge (chords, upper horizontal bracings, stringers, and other

transversal beams), as their loss reduces the structural resistance of the bridge in its upper region. This forces the remaining upper elements to bear increased internal forces.

- *Stringers* (Fig. 122): The removal of stringers similarly reduces the number of resisting elements at the top of the bridge, leading to an increment in internal forces in elements such as the top chords and upper horizontal bracing, with this effect being especially pronounced in the stringers.

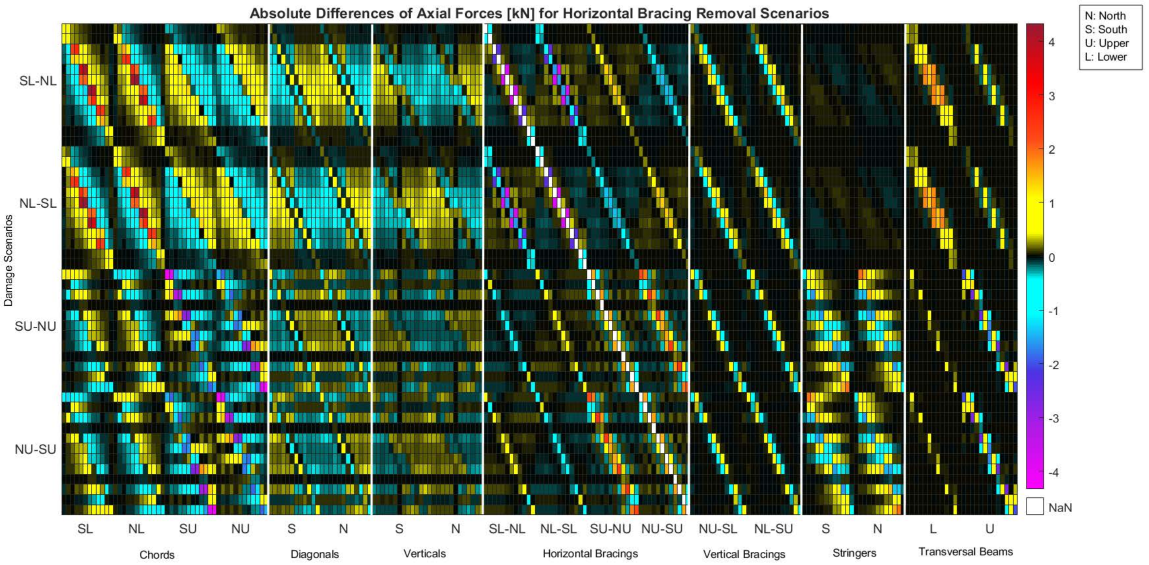

Fig. 119 | Absolute differences of axial forces in kN for the 48 horizontal bracing DSs between the damaged and undamaged states. The vertical axis shows the location of the removed horizontal bracing, while the horizontal axis shows the members for which the axial force effect is evaluated for each DS.

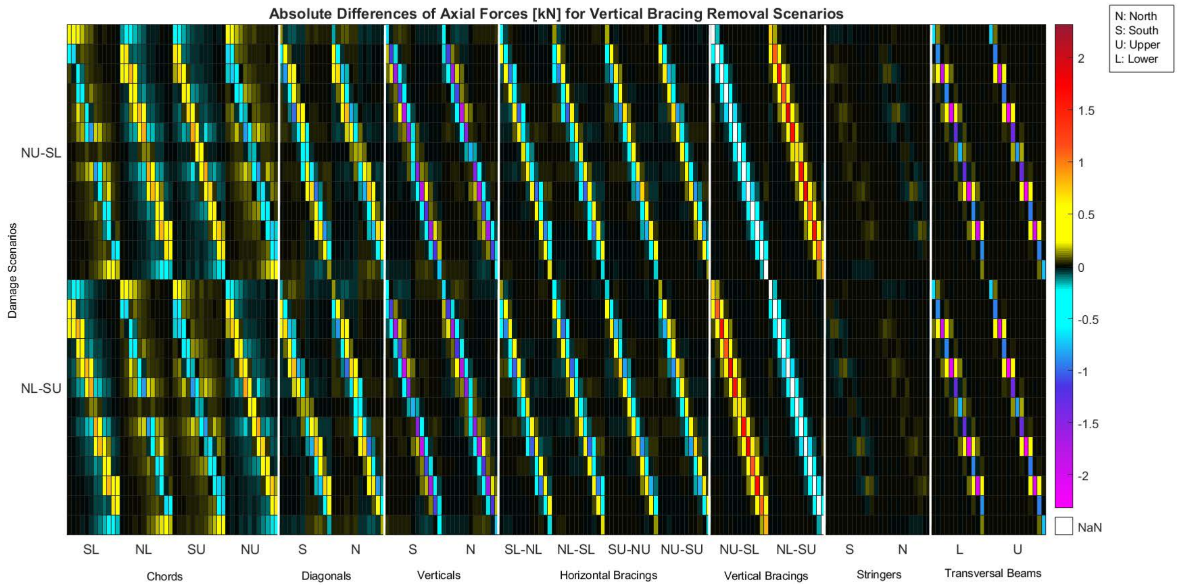

Fig. 120 | Absolute differences of axial forces in kN for the 26 vertical bracing DSs between the damaged and undamaged states. The vertical axis shows the location of the removed vertical bracing, while the horizontal axis shows the members for which the axial force effect is evaluated for each DS.

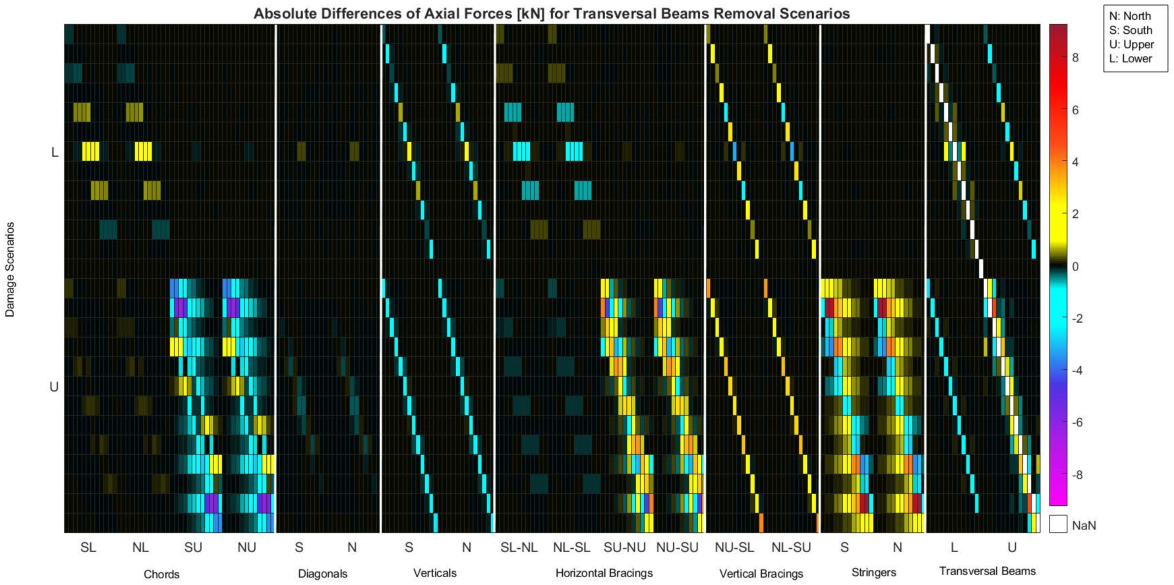

Fig. 121 | Absolute differences of axial forces in kN for the 26 transversal beams DSs between the damaged and undamaged states. The vertical axis shows the location of the removed transversal beam, while the horizontal axis shows the members for which the axial force effect is evaluated for each DS.

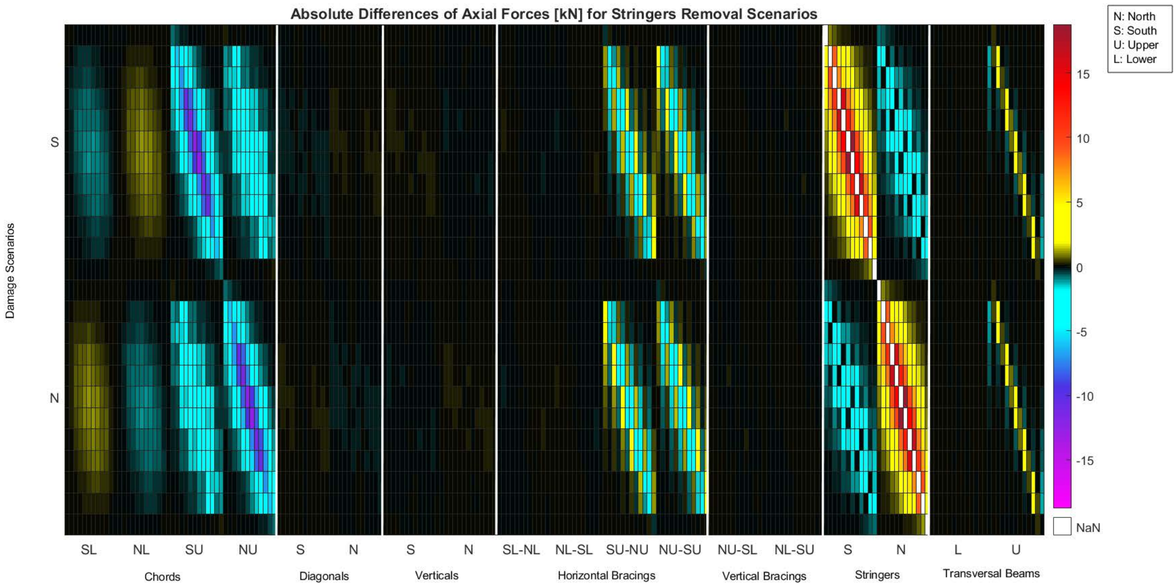

Fig. 122 **| Absolute differences of axial forces in kN for the 24 stringers DSs between the damaged and undamaged states. The vertical axis shows the location of the removed stringer, while the horizontal axis shows the members for which the axial force effect is evaluated for each DS.**

2.4.4.4 Reactions

After the removal of any of these elements, the change in the reactions has been negligible, with values consistently below 0.4 kN. These small values confirm that these DSs have had minimal impact on the overall structural response of the bridge.

2.4.4.5 Validation of the experimental campaign and conclusions

The results analysed here confirm the conclusions drawn from the experimental tests conducted on DS in horizontal and vertical bracings. Specifically, the failure of one of these elements causes very minor changes in the forces within the structure. In terms of bending, the changes are more localised, while axial force changes are somewhat more distributed. This contrasts horizontal and vertical bracings' significant role in forming ALP in DS related to chords, verticals, or diagonals. The same conclusion can be extended to DS involving transversal beams and stringers when the load is applied directly to the main system of the bridge, as outlined in Section 1 (e.g., load setup 1).

However, failures in the **upper transversal beams or stringers, which belong to the floor system**, are critical and **can greatly affect the movement of trains over the bridge** (e.g., see how these elements are usually loaded in Section 1, load setup 2). For example, in the case of stringers, a failure can directly lead to the derailment of the train. Another case with potentially fatal consequences for train movement is that of the upper transversal beams (i.e., of the floor system), whose failure has been assessed only in the experimental part (see Section 1, load setup two and results for the transversal beam damage scenario). The conclusions obtained there can still be applied to these cases. Upon **failure of an upper transversal beam, the adjacent stringers very significantly activate their bending capacity and redirect the load to the neighbouring elements.**

Section 3. Evaluation of the additional defence mechanisms up to collapse

Section 3.1: Description of most important additional assumptions for computational modelling

Using the same simulation technique considered in Section 2, the evolution of the bridge's defence mechanisms has been evaluated, from the load applied in Section 2 (i.e., 80 kN) to the collapse of the structure (i.e., non-convergence of the model). Each of these simulations took between 40 min and 4 h to run depending on the number of buckling events occurring during failure propagation. These simulations were carried out on a computer with 32 GB of RAM and a 13th Gen Intel(R) Core(TM) i7-13700HX processor with a speed of 2.10 GHz.

Various strategically selected scenarios have been analysed, and the ultimate load reached in each scenario has been defined as the "collapse load." The adopted analytical approach is similar to that of the previous section, but in this case, the focus is placed on the variation of axial forces and bending moments at different load levels. This approach enables the identification of how load redistribution evolves as the initial failure propagates to other elements, either through plastic deformations or member buckling, thereby characterizing the defence mechanisms exhibited by the bridge prior to collapse.

Unlike Section 2, in this section, a total of 10 initial local failure scenarios have been defined. These scenarios constitute a comprehensive set of Damage Scenarios (DSs) in which significant differences were observed compared to the behaviour analysed in Section 2. The DSs include one type 1 and one type 2 member for chords (upper and lower), diagonals, and verticals. Besides, two scenarios involving central chords (upper and lower) are considered, as their behaviour may slightly differ from that of the other chords. The selected scenarios are depicted in Fig. 123. Additionally, the undamaged bridge has been simulated until collapse in order to compare its equilibrium path with the other 10 damage scenarios. In this section, for the sake of brevity and cross-referencing, each DS is identified using the same DS numbering adopted in Section 2. The corresponding equivalences can be found in Fig. 123.

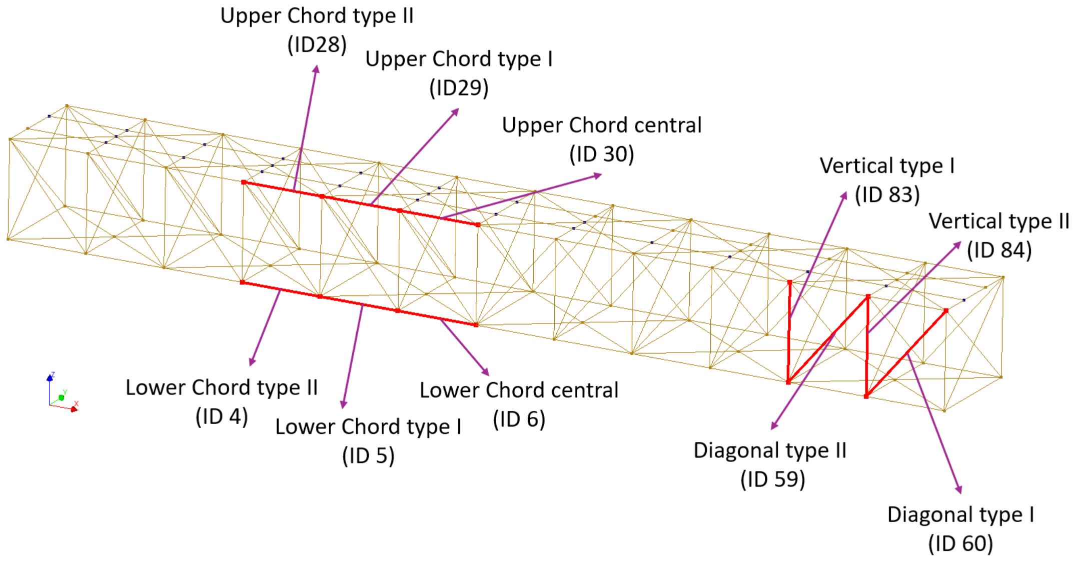

Fig. 123 | Damage scenarios selected to study additional defence mechanisms.

Before initiating the simulation and analysis process, and in order to validate that the model response remains accurate even at high load levels, a second validation stage was conducted, as discussed in Section 3.2. Once it was confirmed that the model reliably represents the laboratory structure within these nonlinear ranges, the analysis was configured for the collapse simulation in the 10 damage scenarios. It is worth mentioning that we used arc-length control[9] to track the equilibrium path up to the collapse of the structure. With this strategy, the simulation is able to overcome critical points on the equilibrium path due to sudden loss of stiffness caused by local instabilities of structural members, finding the way in which forces are redistributed up to the collapse. After simulating the 10 DSs up to the collapse load, the results were analysed using an approach similar to that of the previous sections. For this purpose, two indicators were employed: (1) differences in axial forces and (2) differences in bending moments, both between the damaged and undamaged states of the bridge. In the previous section, these indicators were computed for a load of 80 kN to assess load redistribution and identify ALPs for realistic load levels of a magnitude similar to that induced by an actual train. Since this section aims to evaluate ALPs at higher loads, specifically the collapse load, in comparison to 80 kN, new indicators have been introduced.

The first indicator (Eq. 12) corresponds to what is referred to as the "first line of defence." It quantifies the differences in Internal Forces (IF), axial forces or bending moments, between initial damage and undamaged condition, normalized by the applied load, which is fixed at 80 kN. The second indicator (Eq. 13) pertains to the collapse condition. It represents the same differences, but in this case, they are normalized by the collapse load obtained from the simulation considering initial damage. The sign of the indicator is defined as positive when it reflects an increase in load magnitude (regardless of whether in tension or compression). Conversely, if the total magnitude of the indicator decreases, it is assigned a negative value, indicating a load reduction or a decrease in demand, for both axial forces and bending moments.

It is important to highlight that the normalization of both indicators was performed based on the external load applied in the test, rather than other possible approaches, such as the IF in the undamaged state or the maximum force of the IF in both loading cases. Although this normalization lacks a strict physical meaning, it serves as a consistent and unique reference that provides greater sensitivity in the colour scale and allows for more precise comparisons between different loading conditions and DS, which is the primary objective of this section.

$$\Delta IF_{1^{st}\ line} = abs\left(\frac{IF_{Damaged\ (80kN)} - IF_{Undamaged\ (80kN)}}{80\ kN}\right) * \mathrm{sgn} \qquad \text{Eq. (12.)}$$

$$\Delta IF_{Collapse} = abs\left(\frac{IF_{Damaged\ (Collapse)} - IF_{Undamaged\ (Collapse)}}{Collapse\ Load}\right) * \mathrm{sgn} \qquad \text{Eq. (13.)}$$

$$with \begin{cases} sgn = 1\ if\ abs(IF_{Damaged}) > abs(IF_{Undamaged}) \\ sgn = -1\ if\ abs(IF_{Damaged}) < abs(IF_{Undamaged}) \end{cases}$$

To enhance the visualization of the indicators, the binary colour scale used in Section 2 (Fig. 124) has been modified. The approach used remains the same, employing a binary scale from red to blue with an insensitive zone for variations smaller than 5% of the maximum response. The difference lies in the calculation of the maximum variation, which in this case is determined as the maximum between the first line of defence situation (80 kN) and the collapse condition. This approach provides a unified colour scale for comparing the indicators associated with the first line of defence and those corresponding to the collapse

condition for a given DS. Consequently, it facilitates the comparison between both responses and enables the analysis of the evolution of ALPs as the load increases up to the collapse of the bridge.

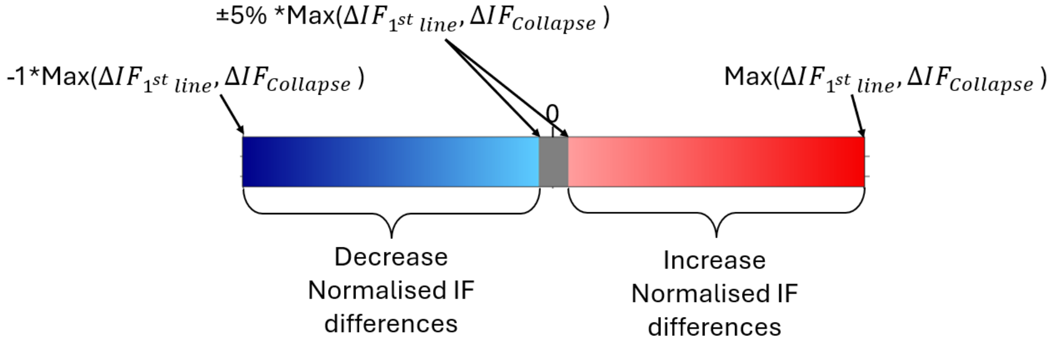

Fig. 124 | Colour scale employed in the representation of performance indicators.

The previously presented indicators are computed for each element in every scenario to enable a straightforward and direct visualization of which elements assume additional load and which experience load reduction following a local failure, up to the collapse of the bridge. To further enhance the visualization of these differences, a third indicator has been introduced. This third indicator quantifies the difference between the previous mentioned indicators for a given IF (axial force or bending moment), as defined in Eq. 14.

$$Difference = \Delta IF_{Collapse} - \Delta IF_{1^{st}\ line} \quad \text{Eq. (14.)}$$

The same colour scale has been adopted for this indicator; however, particular attention must be given to its interpretation. In this case, the colours do not represent load increase or decrease phenomena but rather the variation in internal demand between the 80 kN load case and the collapse load condition. Thus, if an element is displayed in red or blue for this indicator, it should be interpreted as an increase or decrease in internal demand, respectively, as the applied load increases. This indicator provides a more sensitive assessment than the previous two since the maximum value of the scale is determined by the largest difference in internal demand between the two load states. This allows the visualization of all changes in internal forces, regardless of whether they result in a transition between loading and unloading states. Such cases occur when the change in internal demand is smaller than the original demand at 80 kN. This third, more sensitive approach is particularly relevant because elements do not necessarily increase or decrease their internal forces in direct proportion to the externally applied load. Instead, their variation depends on additional factors such as failure propagation or the global ductility of the structural system.

Section 3.2: Validation of the simulation strategy

The increase in load can lead to significant differences due to the amplification of the model's nonlinearities. Therefore, a second validation stage was conducted to assess the model’s performance against the experimental scenario involving the loss of the diagonal (DS 60), extending the analysis up to the collapse of the structure. The validation approach follows a methodology similar to that in Section 2, where displacements and strains were evaluated at the measurement points obtained experimentally. Since the load increase induces instabilities, the arc-length control method was employed. This introduces slight differences between the experimental and the numerical load; however, due to their low magnitude, these differences are negligible for the validation of the model. The selected loads for validation consist of a total of seven Load Sets (LS), ranging from 90 kN to 210 kN, with increments of approximately 30 kN per step.

3.2.1 Displacements

The applied load increases incrementally, with corresponding displacements recorded at selected joints (DoFs), on both the south and north sides of the bridge (similar to section 2). Generally, both experimental and numerical results exhibit a similar trend, with increasing displacements as the load increases. Moreover, for a clear comparison, Fig. 125 illustrates the vertical displacement distribution across the considered DoFs for each load step, enabling a visual evaluation of the correlation between experimental and numerical results. Hence, the overall trend across all graphs shows that displacements reach their maximum at specific joints (midspan) and symmetrically diminish toward the edges, illustrating the structural deformation pattern under applied loads.

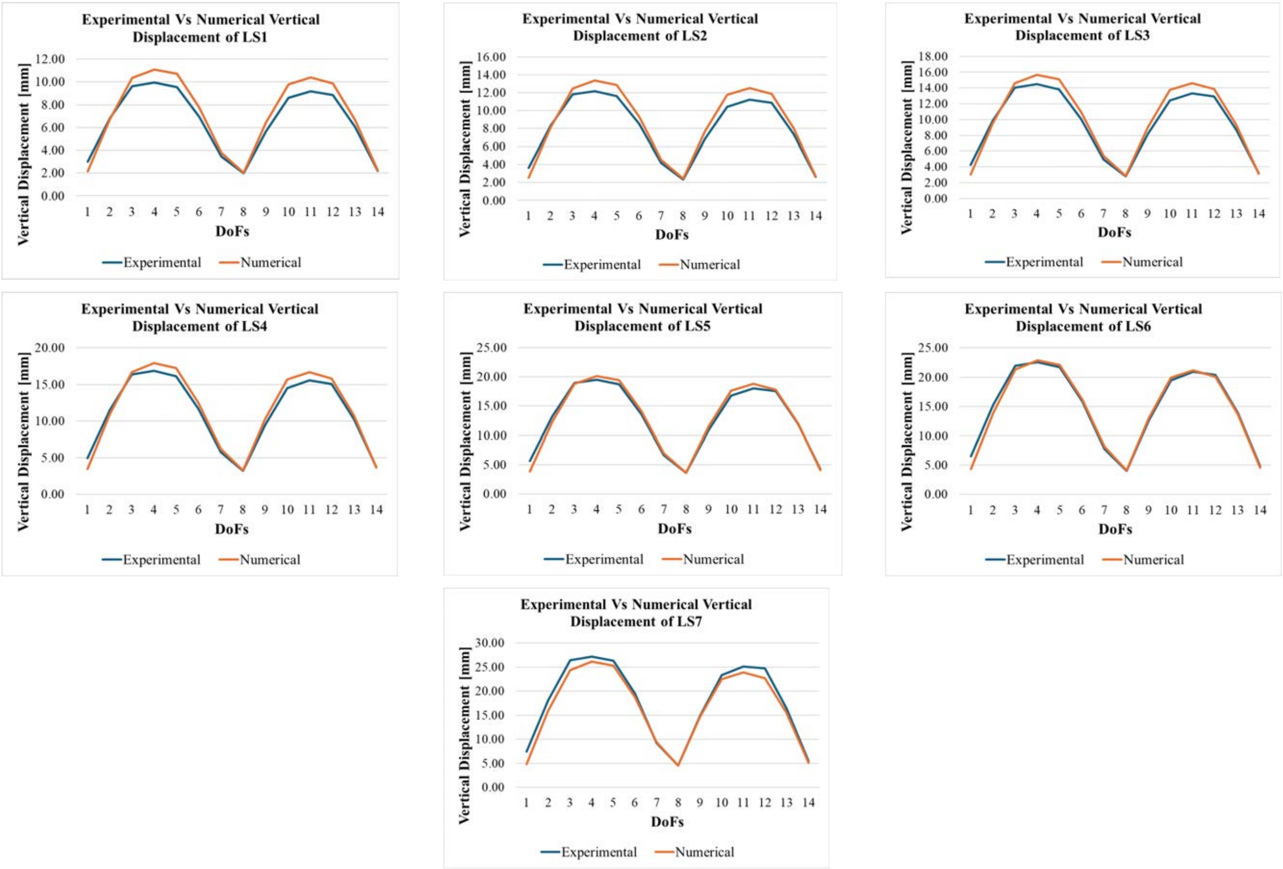

Fig. 125 | Experimental vs Numerical Vertical Displacement of each considered load steps.

The previous figures collectively present a comparative analysis of experimental vs. numerical displacements and across multiple load steps (LS1 to LS7). These results indicate a reasonable agreement between experimental and numerical values, with the numerical model capturing overall trends effectively.

3.2.2 Strains

As the second stage of validation, the strain values are evaluated at the same measurement points as in Section 2 (diagonals and chords). The experimental and numerical strain results (in mε) under the different load steps in the DS60 are illustrated in the following figures. The strain distribution along measurement points can be observed in Fig. 126 to the measurements located at diagonals and Fig. 127 and Fig. 128 to the measurements located at south and north central chords, respectively.

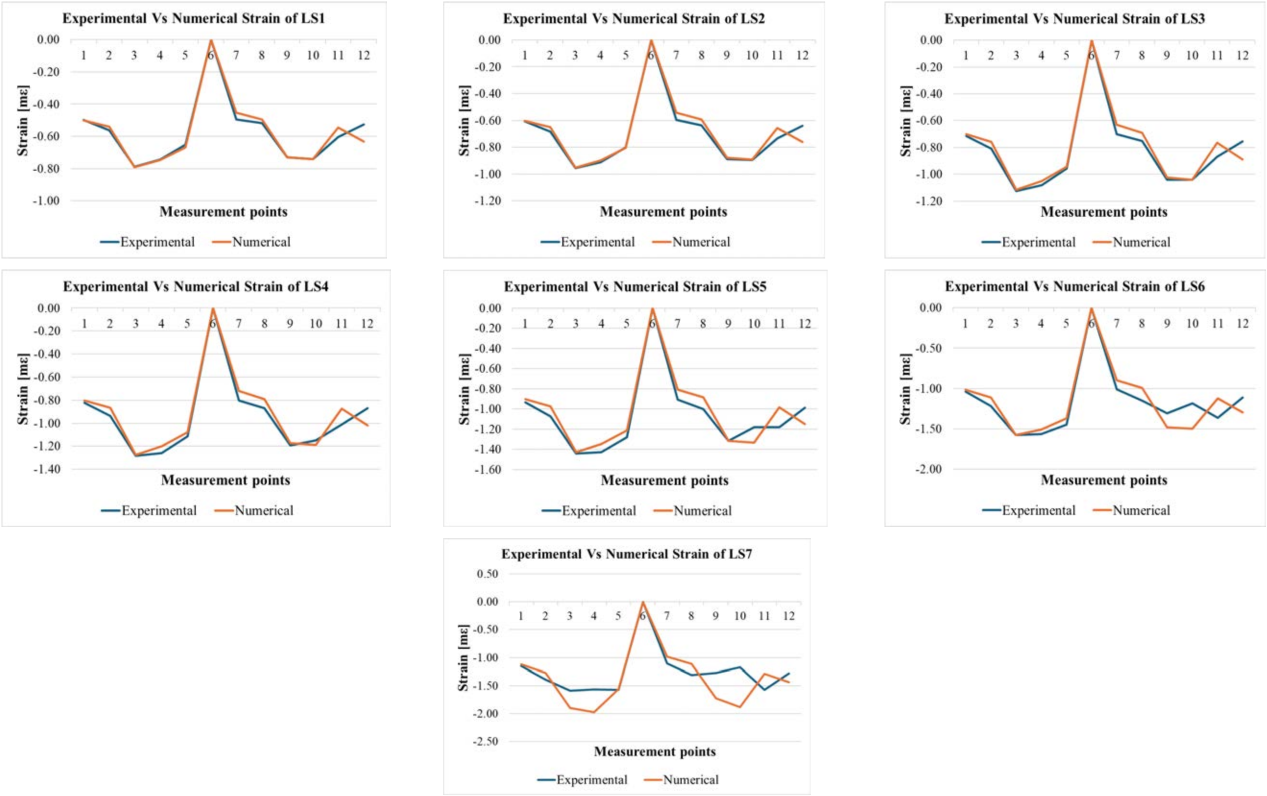

Fig. 126 | Experimental vs Numerical Strain (at diagonals) of each considered load.

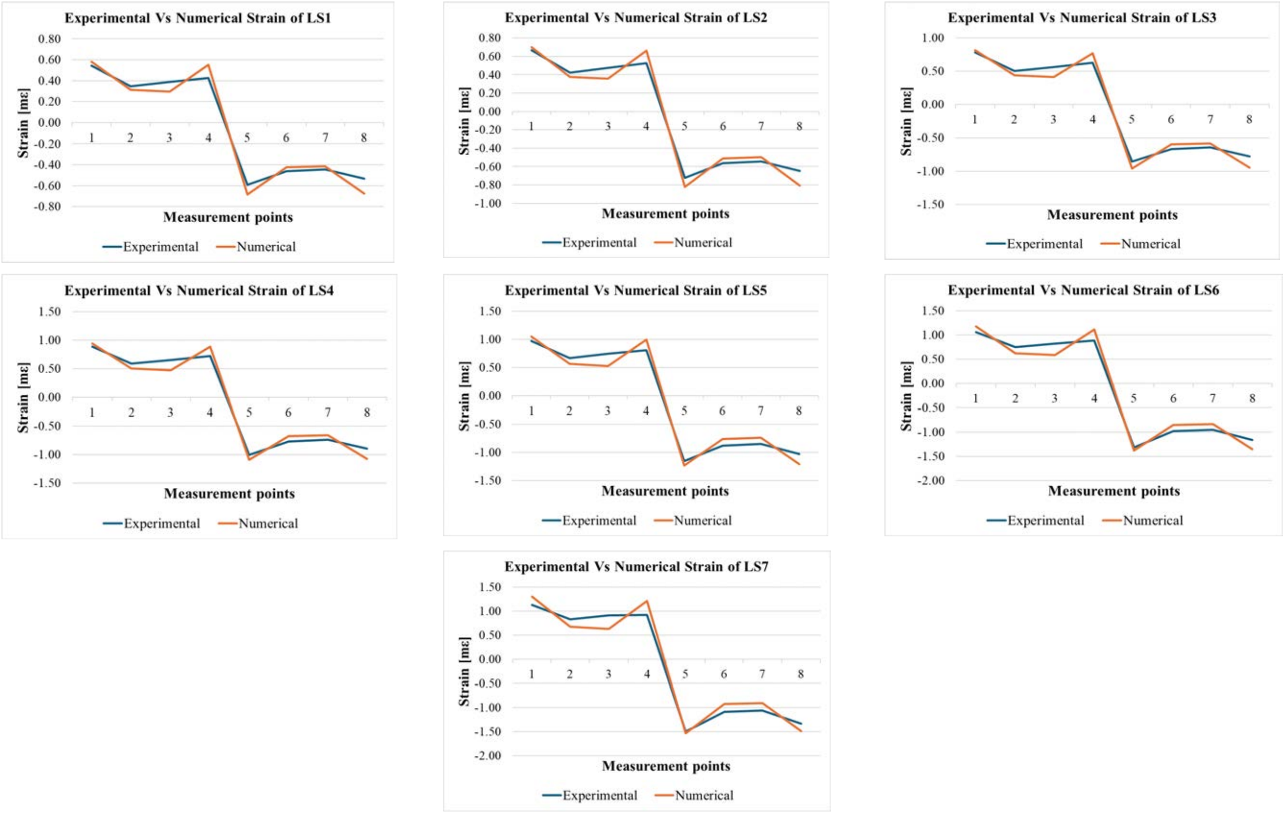

Fig. 127 | Experimental Vs Numerical strain (at south central chords) of each considered load step.

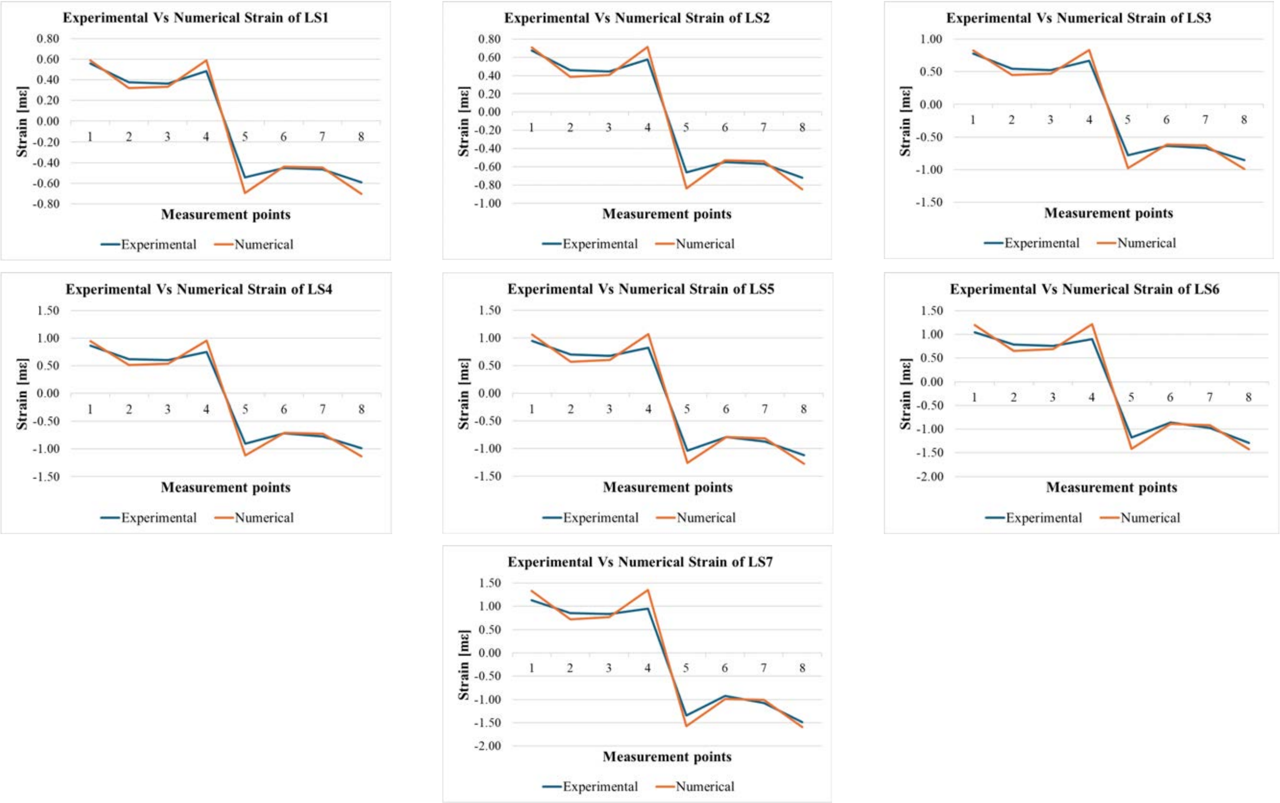

Fig. 128 | Experimental Vs Numerical strain (at north central chords) of each considered load step.

As with displacements, the previous figures collectively present a comparative analysis of experimental and numerical strain across multiple load steps (LS1 to LS7). These results indicate a reasonable agreement between experimental and numerical values, with the numerical model capturing overall trends effectively.

Section 3.3: Load increases for each damage scenario until collapse

After simulating the 10 DSs and the undamaged case, the results were plotted using equilibrium path graphs (Fig. 129). In these graphs, the displacement is measured at the central part of the bridge, specifically at the midpoint of the transversal beam connecting the two lower chords. The represented load corresponds to the load reached by the numerical model. Additionally, a label indicating the ultimate load achieved in each scenario is also displayed.

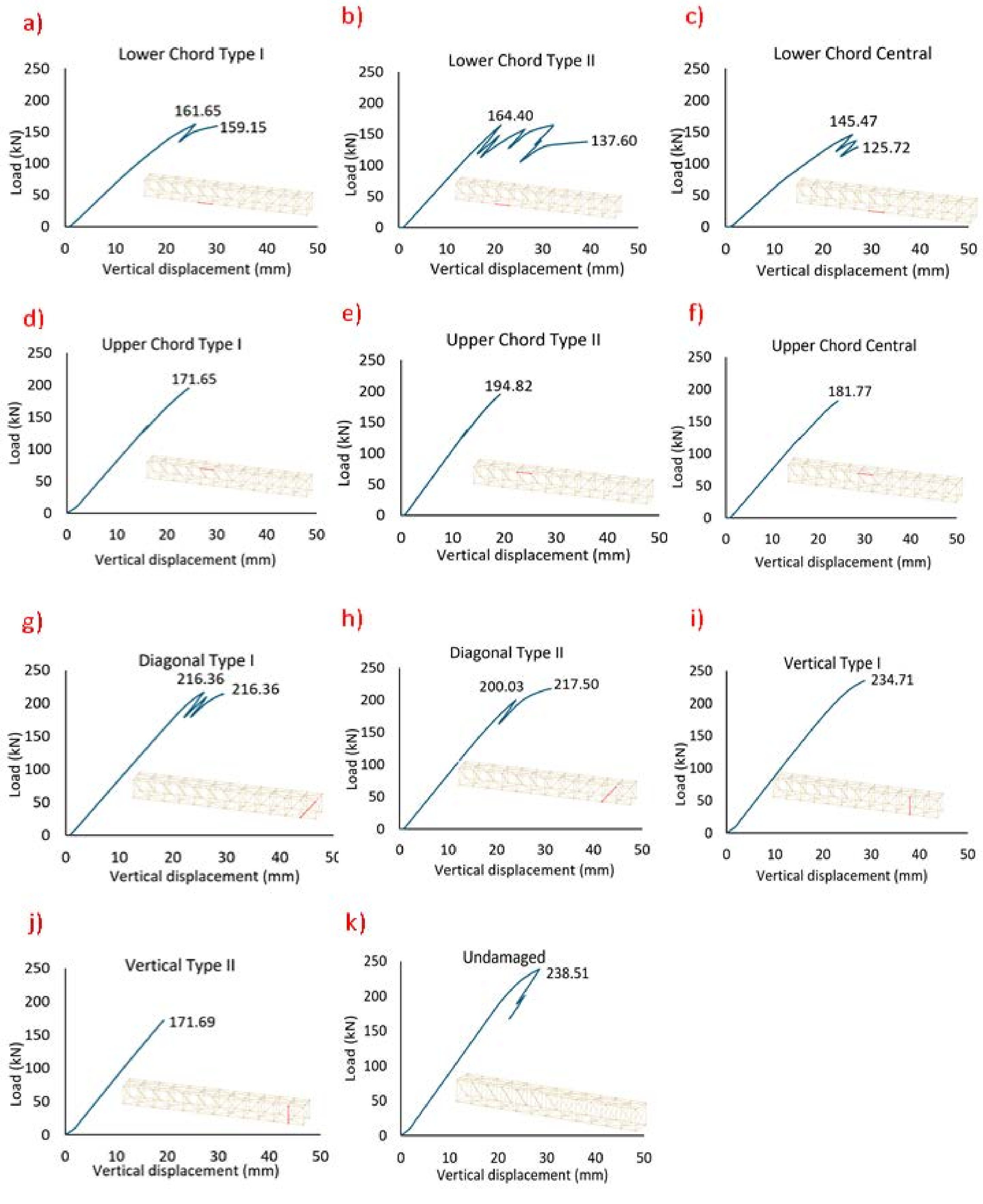

Fig. 129 | Equilibrium path of displacements in midspan for each DS.

As we can observe, the bridge, under all DSs, reaches a collapse load significantly higher than 80 kN, which represents a realistic load level for the bridge under operational conditions. As the load level increases, critical points appear in the equilibrium trajectory for most of the DSs, with a decrease and subsequent recovery of the applied load. This behaviour is primarily due to the propagation of failure caused by the sequential failure of different elements of the bridge, until the collapse load is reached.

In the lower chord scenarios (DSs 4, 5, and 6), successive critical points can be observed, corresponding to the buckling of vertical bracings and vertical elements. In all three scenarios, the maximum load occurs prior to the buckling of any main element. However, scenarios 4 and 5 reach nearly identical maximum loads after the buckling of several elements.

In the upper chord scenarios (DSs 28, 29, and 30), slight oscillations in the applied load can be observed during the process, around 100-150 kN. These oscillations correspond to the buckling of vertical and horizontal bracings.

The diagonal scenarios (DSs 59 and 60) exhibit behaviour similar to that of the lower chord scenarios, where considerable oscillations in the applied load are observed due to the buckling of vertical elements and vertical bracings. In the type 2 diagonal scenario (DS 59), the model reaches its maximum load after the buckling of one of the verticals on the undamaged side. However, in DS 60, after the buckling of one of the verticals on the undamaged side, the model increases the load again until the buckling of another vertical on the damaged side, without surpassing the previously achieved maximum load.

In the case of the vertical scenarios (DSs 83 and 84), a constant load increase is observed until the first buckling of a vertical occurs, leading directly to collapse. In both cases, the closest vertical bracings buckle. However, in the case of the vertical type 1, the buckling that causes the collapse of the structure occurs in one of the verticals on the undamaged side, while in the type 2 scenario, the buckling is localised in the vertical adjacent to the removed element (i.e., towards midspan, on the same side as the initial damage). In the case of vertical type 1, the load until buckling is much higher than for vertical type 2 (234.71 kN for type 1 versus 171.69 kN for type 2).

Section 3.4: Internal forces analysis for different load conditions

The following presents the values of the performance indicators for axial forces and bending moments corresponding to the "first line of defence" situation and the collapse situation for the different DSs. Additionally, figures are included that represent the differences in the indicators between both load states, as well as an image of the model depicting displacements under imminent collapse conditions (where failure propagation can also be identified). The reader is referred to the end of Section 3.1, where the indicators and colour scales are precisely defined.

3.4.1 Damage Scenario 4

The main changes observed between the ALP at the different load states (see Fig. 130 and Fig. 131) are due to the propagation of failure prior to collapse. This failure propagation occurs with the successive buckling of elements without significant material yielding. In general, the main changes between the ALP corresponding to the first line of defence and the ALP corresponding to collapse are reflected in the increase in load transferred from the damaged side to the undamaged side due to the increase in the applied load (from 80 to 137.6 kN). However, the increased load has also caused the buckling of five verticals (two on the damaged side and three on the undamaged side), as shown in Fig. 132. The loss of stiffness in the elements that have buckled causes both morphological and magnitude variations in the ALP. The main observed differences are as follows:

- The axial force of a vertical before buckling is transferred to adjacent elements on the same side once buckling occurs. On the undamaged side (opposite to the side where the chord was removed), the increased load causes the buckling of verticals 3, 5, and 6 (with numbering starting from the left side of the image, the west of the bridge, see Fig. 132), and several vertical bracings, triggering a load transfer process. This transfer causes vertical 4 to significantly increase its load, changing its behaviour from an inactive element (neither increases nor decreases the load) to an element

which increases load in the collapse load condition. On the damaged side (where the chord was removed), the two buckled verticals (5 and 6) transfer load to adjacent verticals (4 and 7), causing them to go from being unloaded and inactive elements to elements which increase their load in the collapse load situation. The buckling of the verticals also causes their axial force to be transferred to the adjacent diagonals (4 and 5), changing them from elements that carry load in the ALP at 80 kN to elements that unload in the collapse situation. The load from these diagonals is transferred to the adjacent diagonals (3 and 6), causing diagonal 3 to go from unloading to increase its load in the collapse situation.

- Axial forces globally increase across the structure despite the normalization of the indicators used (darker shades in the colour scale are observed for the collapse situation). These increases are much more noticeable near the damaged areas, showing their evolution not proportional to the external load applied.
- The Vierendeel mechanism is activated in the collapse load condition after several verticals have buckled in sequence. This mechanism acts locally on the areas where damage has propagated. As a result, an increase in bending moments is observed in the lower chords, upper chords, and stringers close to the damaged elements, which is characteristic of the Vierendeel mechanism.
- The pair of lower horizontal bracings connecting the side of the removed chord to the transversal beam are the elements with the highest yielding. This yielding leads to the loss of stiffness in these elements, causing a slight decrease in their axial force compared to the situation with 80 kN, but without altering their behaviour within the ALP (they continue to increasing load due to the loss of the chord in the collapse load condition).

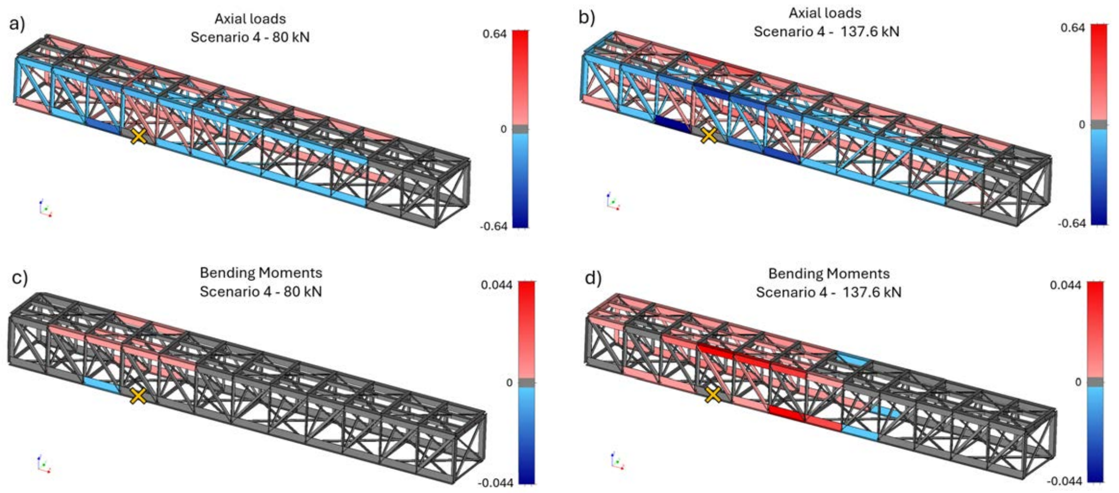

Fig. 130 | ΔIF indicators for DS 4: a) Axial load at 80 kN, b) Axial load at collapse load, c) Bending moments at 80 kN, d) Bending moments at collapse load.

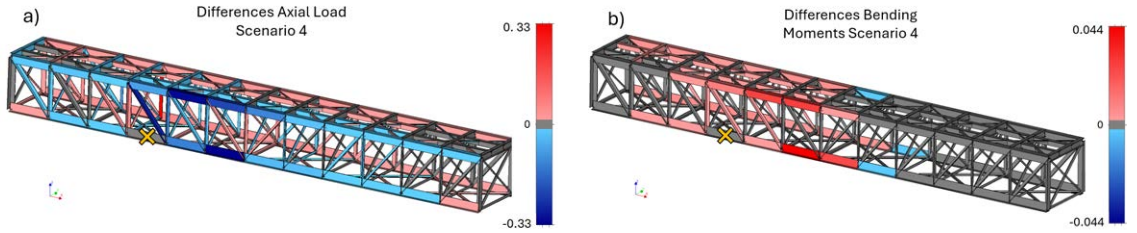

Fig. 131 | Difference of ΔIF indicators for DS 5: a) Axial load differences between 80 kN and collapse load b) Bending moments differences between 80 kN and collapse load.

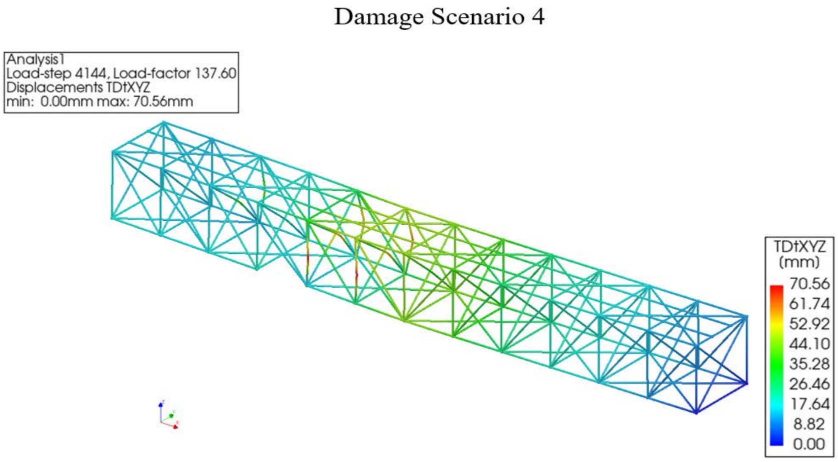

Fig. 132 | Displacements at collapse load for DS 4.

3.4.2 Damage Scenario 5

In this scenario, the main changes due to the difference in applied load are very similar to those observed in the previous case, as they are also caused by failure propagation before collapse (see Fig. 133 and Fig. 134). The damage propagation leads to the buckling of a vertical bracing and a vertical in the span where the chord was removed (Fig. 135), without significant material yielding. The main differences between the 80 kN load state and the collapse load (159.15 kN) are a global increase in the load transfer from the damaged side to the undamaged side, along with some morphological changes in the ALP due to damage propagation. The main observed differences are as follows:

- The increase in axial forces due to load transfer from the damaged side to the undamaged side has led to the buckling of vertical 5 on the undamaged side and the vertical bracings located in the same transverse plane. This results in a reduction of internal forces in these elements and also in diagonals 3 and 4. Consequently, diagonal 4 transitions from to be an element which increase its load to an element which decreases its load in the ALP of the collapse load. As a result, an increase in internal forces is observed in the adjacent verticals (4 and 6) on the undamaged side, as well as in verticals 4, 5, and 7 and their connected diagonals (3, 4, and 6) on the damaged side. However, this increase in internal forces is lower than the load transferred during the chord loss at 80 kN, so most of these elements remain in an unloading process, but with reduced magnitude. The primary morphological changes in the ALP occur in vertical 6 and diagonal 4 on the opposite side of the removed chord, which are subjected to a reverse loading process—changing from loading to unloading and vice versa, respectively. Additionally, vertical 5 on the same side transitions from an inactive state to unloading state.
- The chords also experience a significant global increase in load transfer from the damaged side to the undamaged side.
- The Vierendeel mechanism is activated, increasing bending moments locally in the damaged areas of the bridge. The increase in bending moments is particularly significant in the upper and lower chords of the undamaged side due to the buckling of the vertical. On the damaged side, the lower chords exhibit a more pronounced decrease in bending moments compared to the 80 kN load case, while the upper chords experience an increase in moment magnitudes.
- The highest yielding occurs in the lower horizontal bracings that form a triangular configuration with the removed chord. This yielding leads to stiffness loss and, consequently, a slight decrease in internal forces in these elements. The diagonal in the same span and on the same side as the removed chord (Diagonal 5) also exhibits a higher degree of yielding compared to other elements, resulting

in a loss of stiffness. This stiffness loss causes a decrease in internal forces in Diagonal 5 and Vertical 6 (connected to the diagonal at the lower end). However, this reduction in internal forces is smaller than the load increased in the 80 kN load condition, so these elements continue to decrease their load in the collapse ALP.

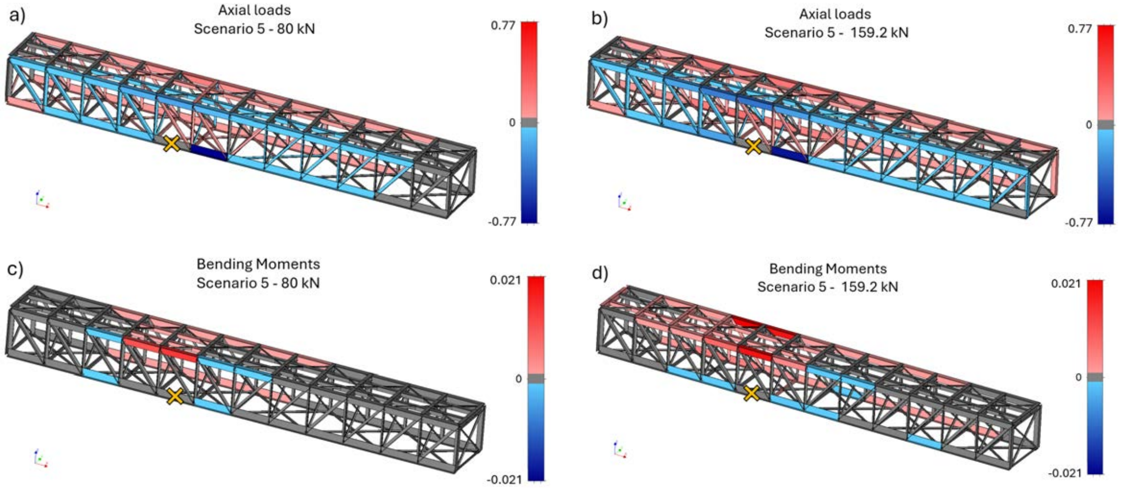

Fig. 133 | ΔIF indicators for DS 5: a) Axial load at 80 kN, b) Axial load at collapse load, c) Bending moments at 80 kN, d) Bending moments at collapse load.

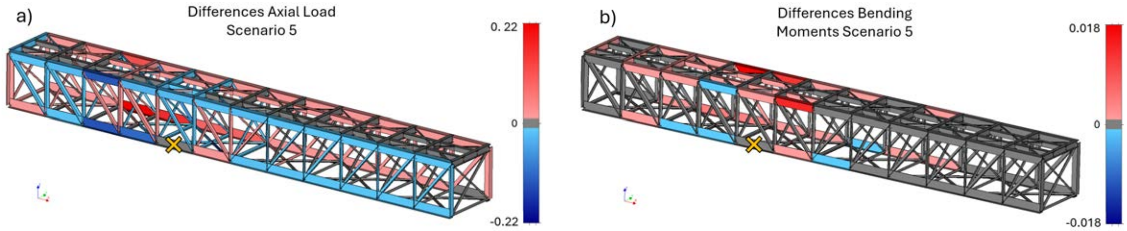

Fig. 134 | Difference of ΔIF indicators for DS 5: a) Axial load differences between 80 kN and collapse load b) Bending moments differences between 80 kN and collapse load.

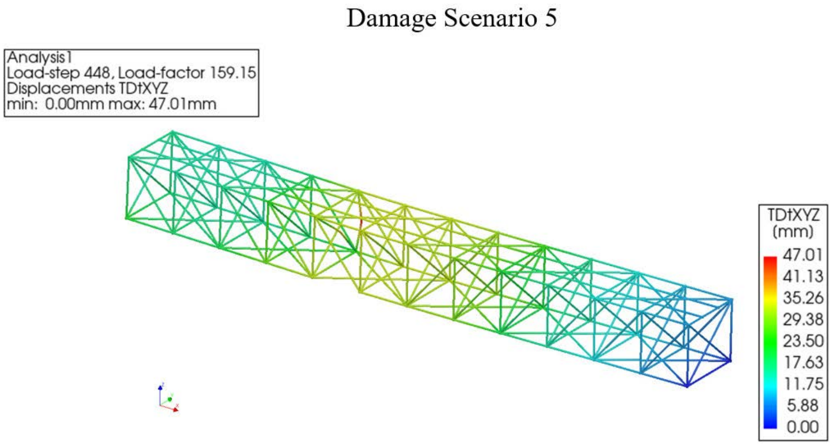

Fig. 135 | Displacements at collapse load for DS 5.

3.4.3 Damage Scenario 6

As in the other two scenarios involving lower chords, the noticeable changes (see Fig. 136 and Fig. 137) due to the difference in applied load are caused by failure propagation before collapse (Fig. 138). The load

increase from 80 kN to the collapse load (125.72 kN) leads to the buckling of two verticals and two vertical bracings on the opposite side of the removed chord, without significant yielding. In addition to the morphological changes in the ALP due to damage propagation, the load transfer from the damaged side to the undamaged side increases. The main differences between both load cases are as follows:

- The buckling of verticals 5 and 6 on the opposite side of the removed chord results in a reduction of internal forces in these elements, as well as in diagonals 4 and 5. This reduction in internal forces is partially redistributed to verticals 4 through 7 and diagonals 4 through 6 on the side where the chord was removed. However, since these elements were in an unloading process under the 80 kN load condition, and the forces redistributed among them are lower than the transferred load, only verticals 5 and 7, along with diagonal 6 on the damaged side, are subjected to a reverse loading process, transitioning from unloading to loading in the collapse load condition.
- Globally, in the chords the load transfer from the damaged side to the undamaged side increases.
- The Vierendeel mechanism is activated locally in the damaged areas (buckled elements and removed chord), primarily in the upper chords, as observed in the previous cases.
- Significant yielding occurs in the lower horizontal bracings that form a triangular configuration with the removed chord. This yielding leads to a slight decrease in internal forces compared to the 80 kN condition, but to a lesser extent than the load increased due to the removal of the chord. Therefore, these elements continue increasing their loads within the ALP at the collapse condition.

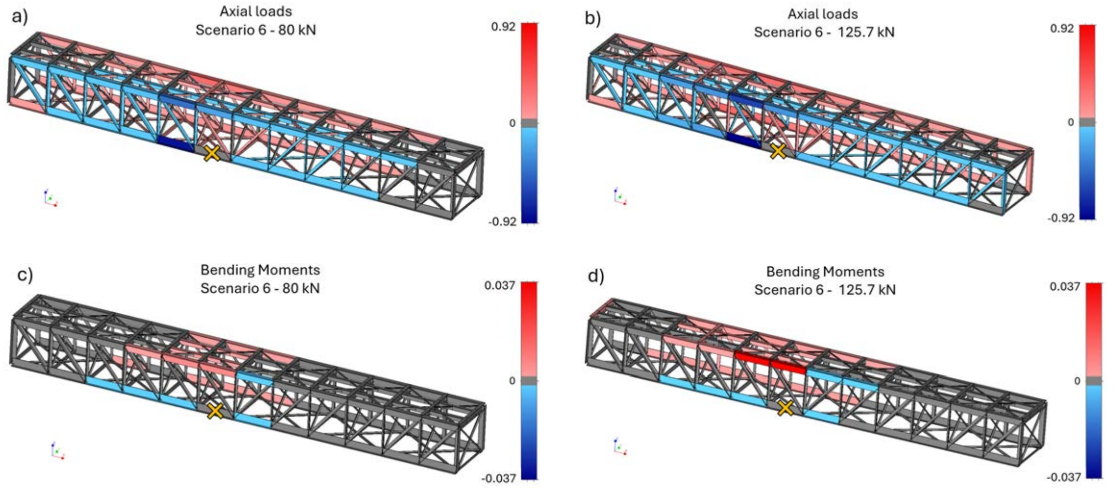

Fig. 136 | ΔIF indicators for DS 6: a) Axial load at 80 kN, b) Axial load at collapse load, c) Bending moments at 80 kN, d) Bending moments at collapse load.

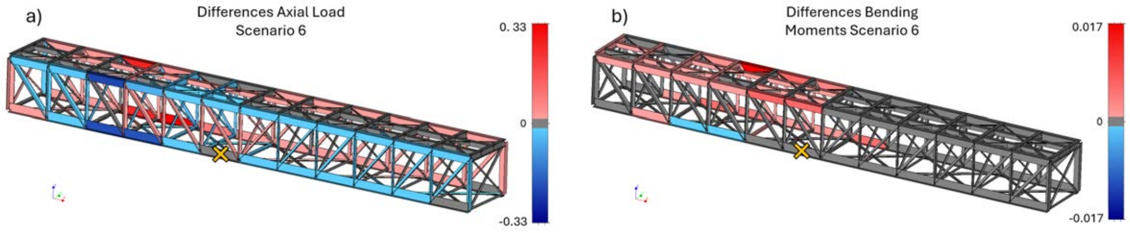

Fig. 137 | Difference of ΔIF indicators for DS 6: a) Axial load differences between 80 kN and collapse load b) Bending moments differences between 80 kN and collapse load.

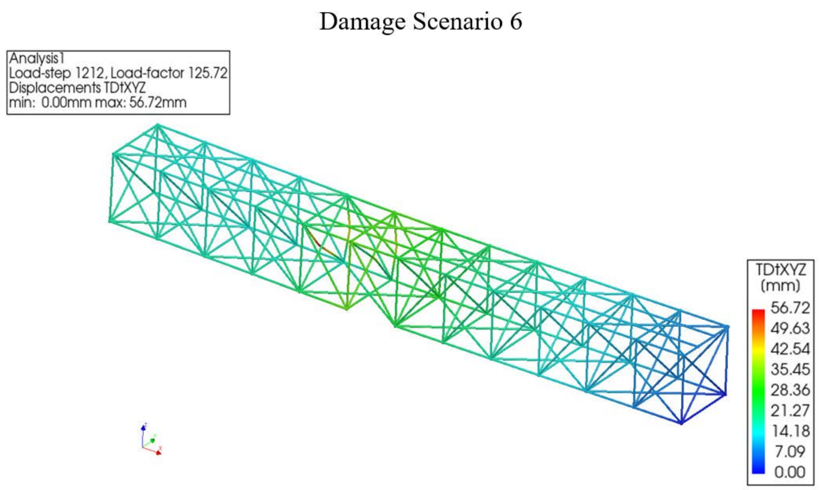

Fig. 138 | Displacements at collapse load for DS 6.

3.4.4 Damage Scenario 28

In this upper chord damage scenario, the load increase from 80 kN to the collapse load (194.82 kN) leads to the buckling of a horizontal bracing (Fig. 141), as well as high yielding in the area near the buckling. The differences between the ALPs obtained at 80 kN and at the collapse load are significantly smaller than those observed in the lower chord scenarios (Fig. 139 and Fig. 140). Additionally, there is no global increase in load transfer as observed in the lower chord scenarios. The main differences between both ALPs are described below:

- The buckling of the upper horizontal bracing leads to its unloading, which in turn reduces the demand on the upper transversal beams, primarily on the transversal beam connected to the buckled horizontal bracing. This load is mainly redistributed to the stringer, which experiences increased demand at the collapse load. The lower horizontal bracing analogous to the buckled one also exhibits a reduction in its internal forces, transitioning from an element in a loading process to an inactive element at the collapse load. The rest of the horizontal bracings (both upper and lower) are affected globally, increasing their load transfer.
- The increase in the applied load results in a global but relatively minor increase in the load transfer from the damaged side to the undamaged side.
- The variations in bending moments between both ALPs are also lower than in the previous scenarios. The most significant increases in bending moments occur in the lower chords on the undamaged side and in the chord connected to the buckled horizontal bracing.

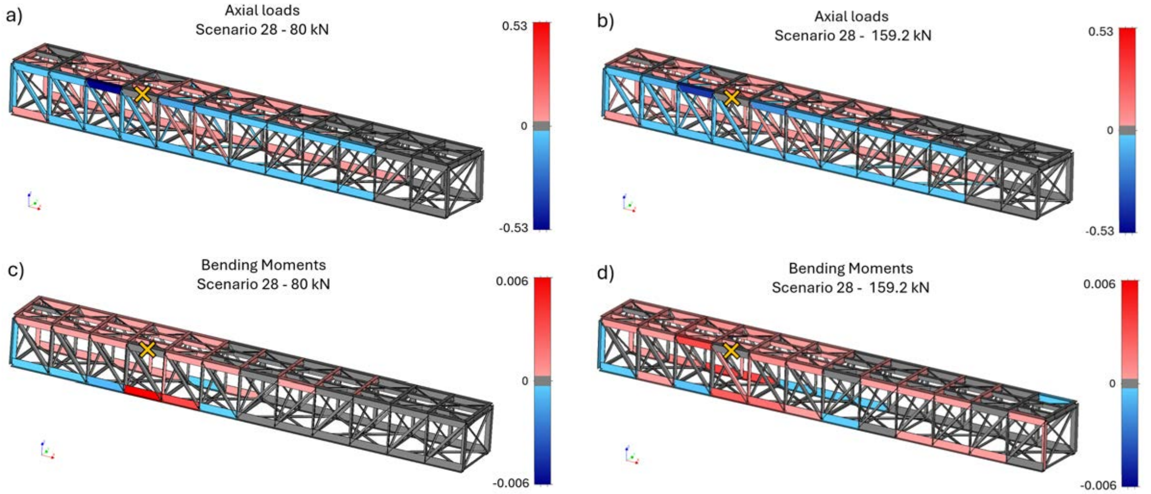

Fig. 139 | ΔIF indicators for DS 28: a) Axial load at 80 kN, b) Axial load at collapse load, c) Bending moments at 80 kN, d) Bending moments at collapse load.

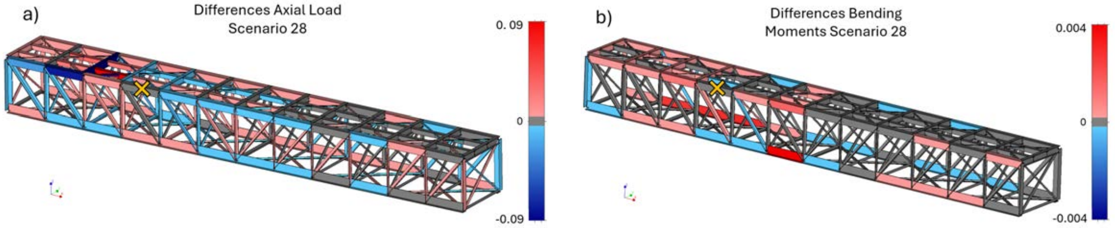

Fig. 140 | Difference of ΔIF indicators for DS 28: a) Axial load differences between 80 kN and collapse load b) Bending moments differences between 80 kN and collapse load.

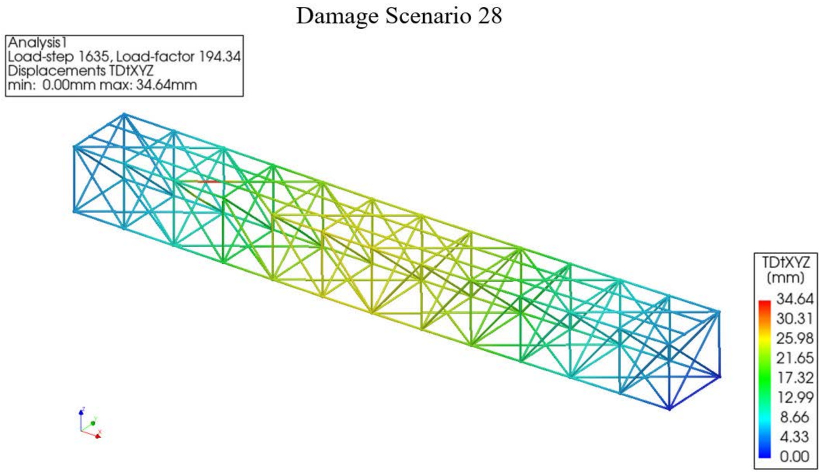

Fig. 141 | Displacements at collapse load for DS 28.

3.4.5 Damage Scenario 29

The load increase from 80 kN to the collapse load (171.65 kN) induces effects similar to those observed in the previous scenario. In this case, damage propagation occurs through the buckling of the horizontal bracing analogous to the previous case but located closer to midspan (Fig. 144). The main differences with respect to the previous scenario stem from the increased variations in axial forces between the two load states due to the local effects of the horizontal bracings (Fig. 142 and Fig. 143). The key differences are detailed below:

- The buckling of the horizontal bracing leads to a reduction in its internal forces, causing the element to transition from increasing its load in the 80 kN ALP to becoming an inactive element in the collapse ALP. This load is primarily redistributed to the parallel horizontal bracing located at midspan. The buckling also leads to a reduction in the internal forces of the upper transversal beams. Lastly, the stringers experience an increase in demand from the supports to the damaged area and a decrease in demand from the removed chord to midspan. Nevertheless, they all remain elements that increase their load in the collapse ALP, as the initial forces at 80 kN exceed the reduction in demand caused by the buckling of the horizontal bracing at the collapse load.
- The increase in the applied load results in a less global and lower-magnitude effect on the loading and unloading processes in the damaged and undamaged sides, respectively, compared to the previous scenario. This is due to the greater influence of the horizontal bracings in this case.
- The differences in bending moments relative to the 80 kN condition are minimal. The main variations include an increase in demand in the upper and lower chords near the midspan on the damaged side and a decrease in demand in the corresponding area on the undamaged side. However, all these elements continue to increase their load in the collapse condition, except for the chord adjacent to the removed one (toward midspan), which experiences unloading.

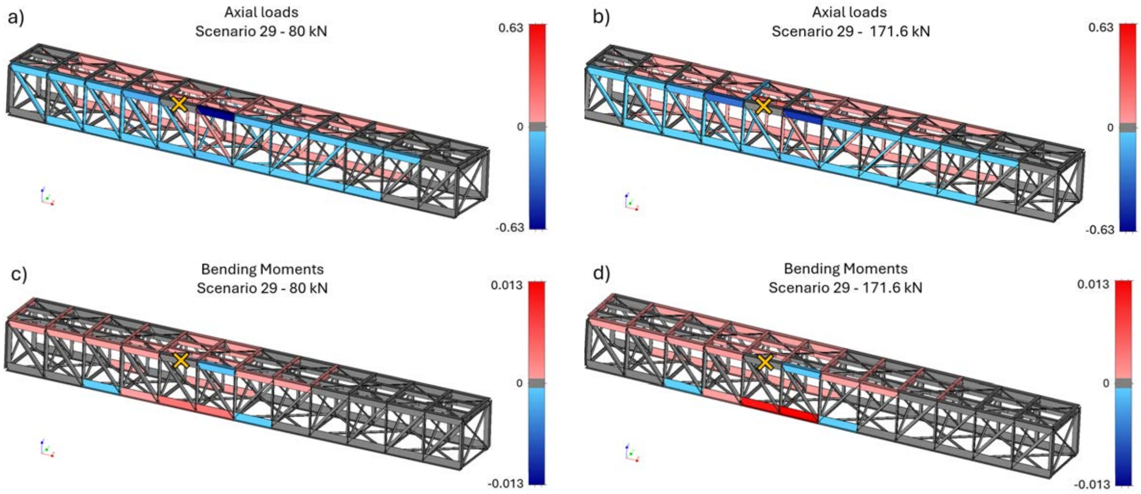

Fig. 142 | ΔIF indicators for DS 29: a) Axial load at 80 kN, b) Axial load at collapse load, c) Bending moments at 80 kN, d) Bending moments at collapse load.

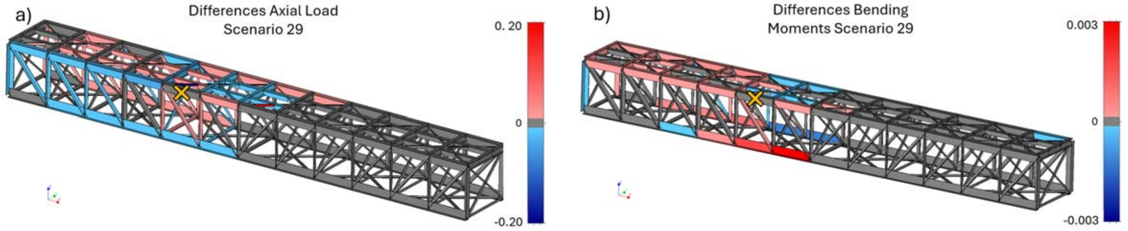

Fig. 143 | Difference of ΔIF indicators for DS 29: a) Axial load differences between 80 kN and collapse load b) Bending moments differences between 80 kN and collapse load.

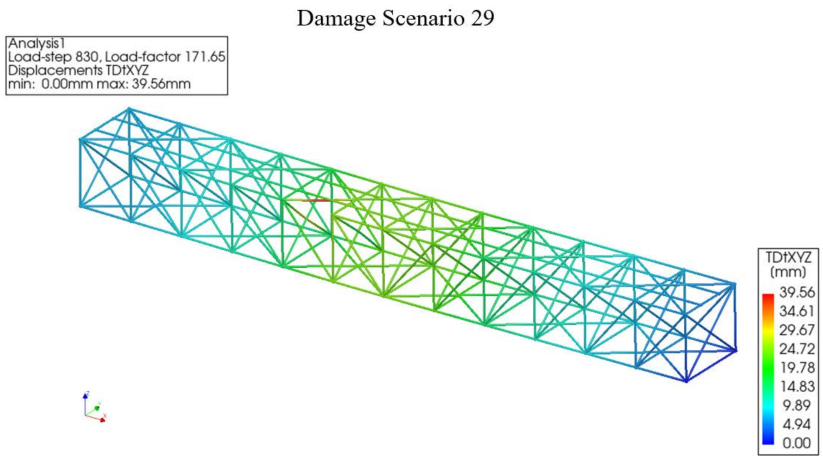

Fig. 144 | Displacements at collapse load for DS 29.

3.4.6 Damage Scenario 30

In the central upper chord damage scenario, no significant differences are observed (see Fig. 145 and Fig. 146) when increasing the load from 80 kN to the collapse load (181.77 kN). The load increase induces high yielding in the two upper horizontal bracings forming a triangle with the removed chord and its adjacent one (Fig. 147). The variations in axial force are of similar magnitude to those in Scenario 28 but occur more locally in the area subjected to high yielding. The chords experience global load increases and reductions, though of very low magnitude. The upper chord of the span preceding the horizontal bracing with the highest yielding are subjected to the greatest increase in bending moments; however, this increase remains relatively small compared to other damage scenarios. These slight variations in IF magnitudes do not result in morphological changes in the ALP compared to the 80 kN load condition. The key differences are detailed below:

- The yielding of the horizontal bracings connected to the removed chord leads to the unloading of these elements. This leads the elements to transition from increasing their load in the 80 kN ALP to become inactive elements at the collapse load. The reduction in internal forces in these elements also results in a decrease in demand in the upper transversal beam connected to them. Consequently, the only transversal beam that carried load in the 80 kN ALP becomes inactive in the collapse ALP. This redistributed load is primarily transferred to the stringers, which increase their demand in the collapse condition.
- The variations in axial forces are localised in the region experiencing high yielding. The chords exhibit global load increases and reductions (undamaged and damaged sides), but of very low magnitude.
- The bending moments remain nearly constant, except for the chord connected to one of the horizontal bracings, which is subjected to a notable increase in demand, transitioning from an unloading process to a loading process in the element in the collapse condition.

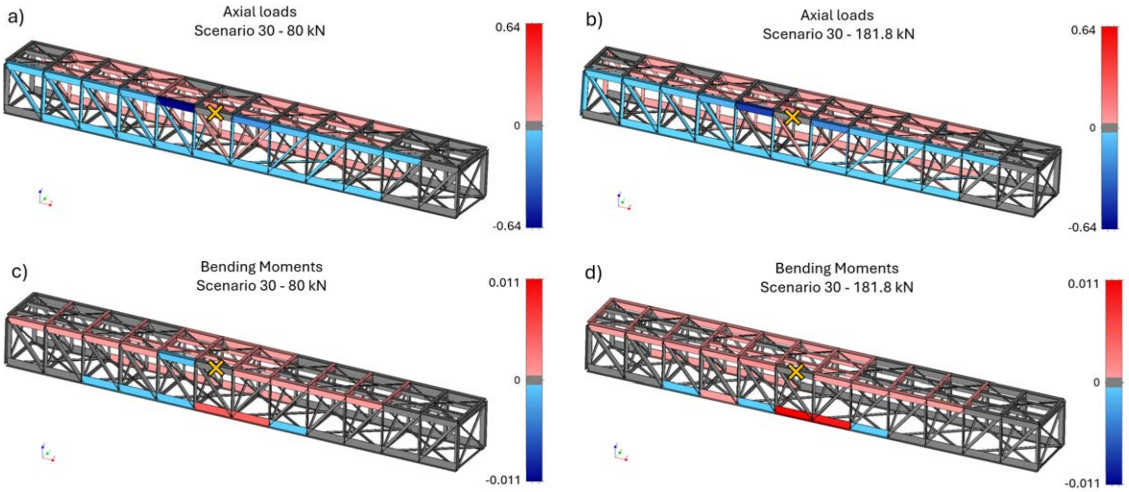

Fig. 145 | ΔIF indicators for DS 30: a) Axial load at 80 kN, b) Axial load at collapse load, c) Bending moments at 80 kN, d) Bending moments at collapse load.

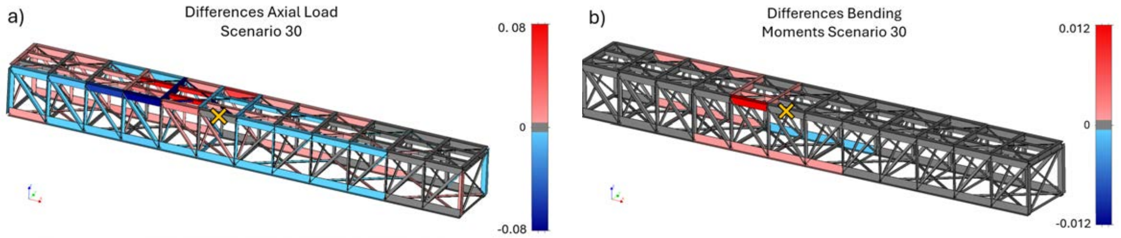

Fig. 146 | Difference of ΔIF indicators for DS 30: a) Axial load differences between 80 kN and collapse load b) Bending moments differences between 80 kN and collapse load.

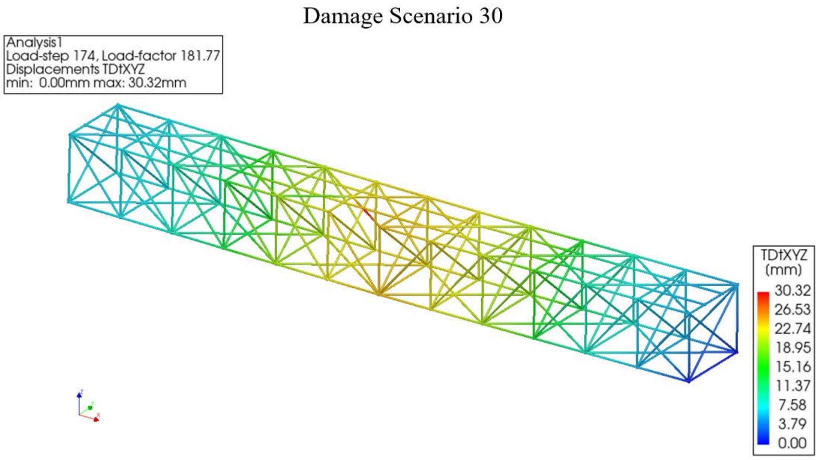

Fig. 147 | Displacements at collapse load for DS 30.

3.4.7 Damage Scenario 59

The main differences in the DS (Fig. 148 and Fig. 149) resulting from the load increase from 80 kN to the collapse load (217.50 kN) are primarily due to the damage propagation. This occurs through the buckling of the vertical bracing and the vertical member in the same span as the removed diagonal but on the undamaged side (Fig. 150). This buckling induces a load transfer to two verticals and one of the adjacent diagonals (the one connected to the bridge end). In this scenario, the increase in axial force is more localised than in other cases but leads to several morphological changes in the ALP. The damage propagation also

triggers the activation of the Vierendeel mechanism, locally increasing the bending moments in the upper and lower chords in the region where the damage spreads. The main differences between both ALPs are detailed below:

- On the undamaged side, the differences in the axial force indicator between both ALPs are due to the buckling of one of the verticals in the same span (towards midspan) as the removed diagonal but on the undamaged side. This leads to the unloading of that vertical (which carried load in the 80 kN ALP) as well as a reduction in the demand of the two adjacent diagonals connected to it. These diagonals, which initially increase their load in the 80 kN ALP, experience different changes according to their original demand: one starts an unloading process (the diagonal towards the support), while the other becomes inactive in the collapse ALP. The redistributed load is primarily assumed by adjacent vertical and diagonal members, which increase their load in the collapse ALP.
- On the damaged side, the primary differences in axial force occur in the two diagonals adjacent to the removed one. The diagonal adjacent to the removed member towards midspan is subjected to a significant reduction in internal forces, transitioning from increasing its load (at 80 kN) to becoming in an inactive component. This reduction may have been exacerbated by the yielding of this diagonal, as it is one of the elements experiencing the highest strain. Meanwhile, the two diagonals adjacent to these elements (one next to the removed diagonal and the other next to the unloaded diagonal) experience a substantial increase in load. Specifically, the diagonal towards the support increases its load, while the one towards midspan transitions from being inactive to increasing its load.
- The diagonal between the support and the removed diagonal, as well as the vertical member on the undamaged side located between the buckled vertical and the support, are the most demanded. However, this scenario does not induce significant global changes in axial force transfer between the damaged and undamaged sides.
- The vertical buckling significantly increases the bending moments in the lower and upper chords in the region where the vertical failed, producing a localised effect. The resulting ALP indicates that the right end of the bridge experiences a substantial increase in bending moments due to the presence of two Vierendeel mechanisms—one caused by the removed diagonal and the other by the buckled vertical.

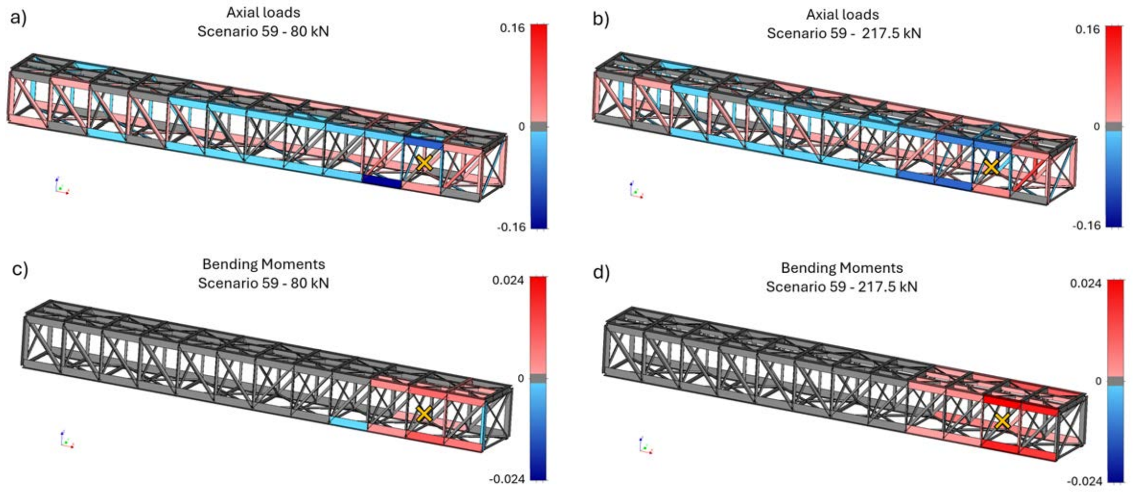

Fig. 148 | ΔIF indicators for DS 59: a) Axial load at 80 kN, b) Axial load at collapse load, c) Bending moments at 80 kN, d) Bending moments at collapse load.

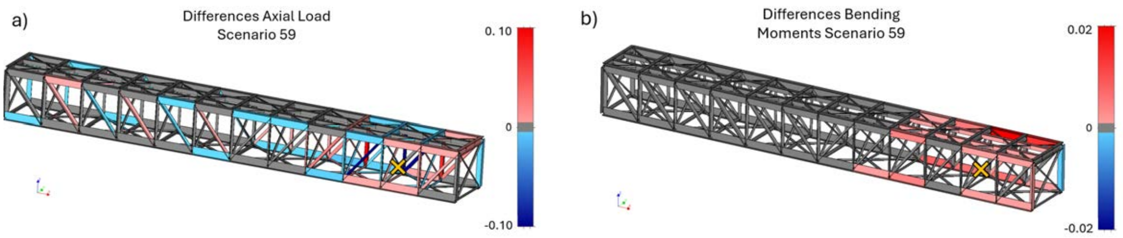

Fig. 149 | Difference of ΔIF indicators for DS 59: a) Axial load differences between 80 kN and collapse load b) Bending moments differences between 80 kN and collapse load.

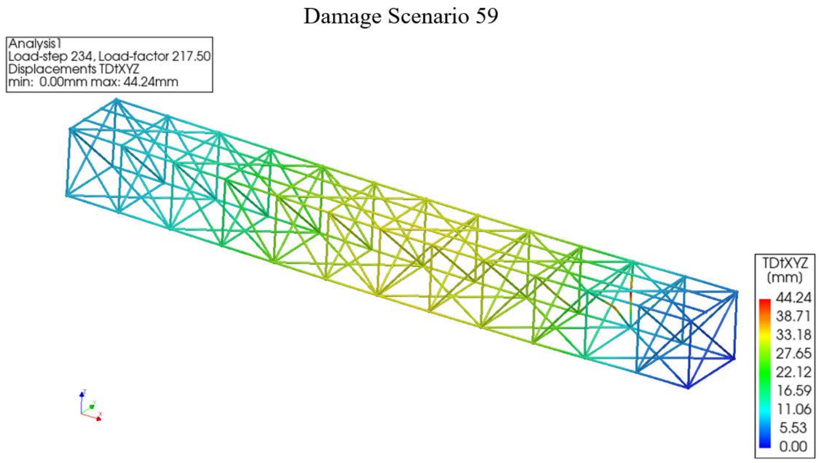

Fig. 150 | Displacements at collapse load for DS 59.

3.4.8 Damage Scenario 60

The load increase from 80 kN to the collapse load (216.36 kN) leads to damage propagation through the buckling of two verticals and two vertical bracings (Fig. 153). In this case, the differences in axial forces exhibit a more global nature compared to the previous scenario, although several morphological changes in the ALP also occur (Fig. 151 and Fig. 152). Additionally, the Vierendeel mechanism is activated in the regions where the buckling took place. The main differences between both ALPs are detailed below:

- All elements in this plane (the two verticals and one of the vertical bracings) experience a reduction in internal forces, except for the lower transversal beam, which is subjected to a slight increase. This reduction implies that these elements transition from being inactive in the 80 kN ALP (except for the vertical on the damaged side, which increases its load) to experiencing unloading in the collapse load state. The redistributed load is primarily transferred to the two pairs of verticals forming the adjacent transverse planes. This results in the verticals in the transverse plane adjacent to the unloaded one (towards midspan) transitioning from being inactive elements to increasing their load. In the other adjacent transverse plane (towards the support), the vertical on the undamaged side increases the load, whereas the vertical on the damaged side transitions from unloading to loading.
- The diagonals adjacent to the unloaded transverse plane (on both sides) also experience a reduction in internal forces. Consequently, the two diagonals adjacent towards midspan transition from being inactive in the 80 kN ALP to elements that decrease their load. Regarding the diagonals adjacent towards the support, the diagonal on the undamaged side transitions from unloading to loading, while the diagonal on the damaged side decreases its load. This load is transferred to the adjacent diagonals.

- The vertical bracings in the spans where the diagonal was removed and where the verticals buckled exchange their function in the ALP (loading/unloading) relative to the 80 kN ALP.
- The transverse plane and the two diagonals that experienced unloading also exhibit a higher state of yielding compared to the rest of the elements.
- The Vierendeel mechanism is locally activated around the buckled elements, significantly increasing the bending moments in both the upper and lower chords on both the damaged and undamaged sides.

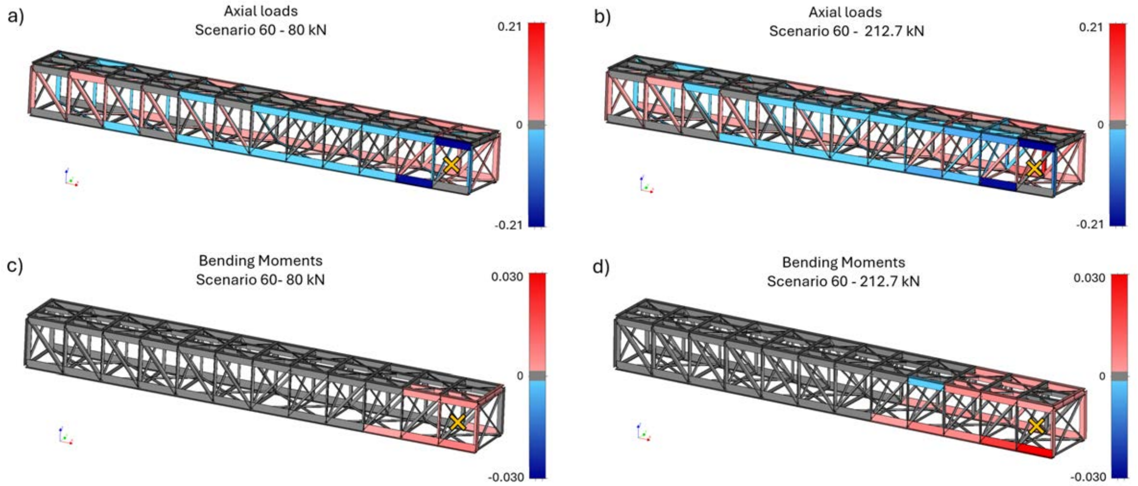

Fig. 151 | ΔIF indicators for DS 60: a) Axial load at 80 kN, b) Axial load at collapse load, c) Bending moments at 80 kN, d) Bending moments at collapse load.

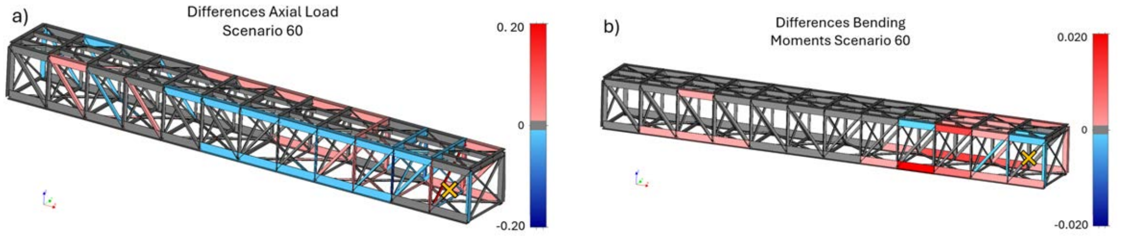

Fig. 152 | Difference of ΔIF indicators for DS 60: a) Axial load differences between 80 kN and collapse load b) Bending moments differences between 80 kN and collapse load.

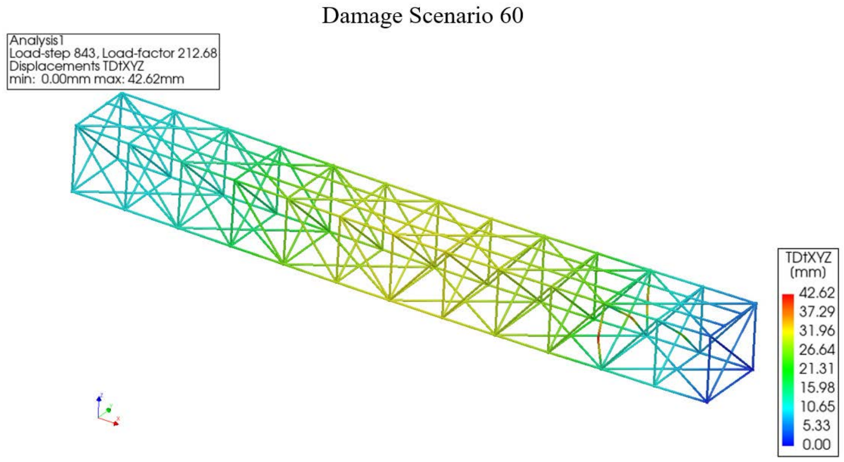

Fig. 153 | Displacements at collapse load for DS 60.

3.4.9 Damage Scenario 83

The Type I vertical scenario (DS 83) corresponds to the case with the highest collapse load (234.71 kN). The load increase led to the buckling of a vertical bracing (Fig. 156). The subsequent load transfer from the vertical bracings to two adjacent verticals triggered their near-simultaneous buckling (significantly more pronounced in one of them), ultimately leading to model unloading and subsequent divergence. In this scenario, the indicators do not show significant differences between the two loading states, as the redistribution remains mostly localised between the damaged elements and their adjacent components. There are barely any morphological changes between both loading conditions, except for the buckled vertical bracing, the lower transversal beam, and the upper and lower horizontal bracings, all within the same span (Fig. 154 and Fig. 155). The differences in axial forces are minor and primarily localised in the damage propagation area. Similarly, no significant variations are observed in the ALP in terms of bending moments, except for a slight magnitude increase (while maintaining the same morphology) due to the Vierendeel effect triggered by the vertical failure. The main differences are detailed below:

- The buckled vertical bracing caused a reduction in internal forces in the other vertical bracing and in the upper and lower transversal beams within the same transverse plane. However, this reduction did not induce any morphological change in the ALP, except for the lower transversal beam, which transitioned from increasing its load to become in an inactive element. The redistributed load was primarily transferred to the analogous vertical on the undamaged side, as well as to the adjacent vertical and diagonal (towards midspan). Despite this, given that the decrease in load in the vertical bracing was of low magnitude, no significant alterations in the ALP behaviour occurred between the two loading states.
- The overall increments in axial force were of low magnitude and practically negligible. The most significant axial force changes were localised in the vicinity of the damaged region.
- Except for the load transferred from the buckled vertical bracing to the diagonal, all other diagonals experienced slight reductions in internal forces, which can be attributed to stiffness loss due to yielding. Once again, these elements exhibited the highest strain levels.
- This scenario shows almost no differences in bending moments between the two loading states. A slight increase in moments was observed in the areas activated by the Vierendeel mechanism, which was already present in the 80 kN loading state. The remaining changes were of negligible magnitude. Morphologically, the only noticeable differences were the slight increase in moments on the undamaged side, near the buckled vertical bracing.

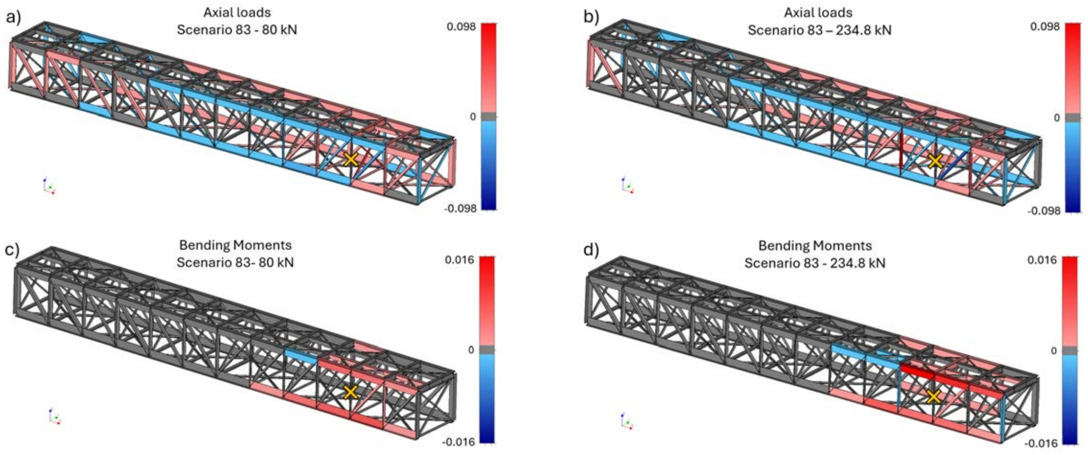

Fig. 154 | ΔIF indicators for DS 83: a) Axial load at 80 kN, b) Axial load at collapse load, c) Bending moments at 80 kN, d) Bending moments at collapse load.

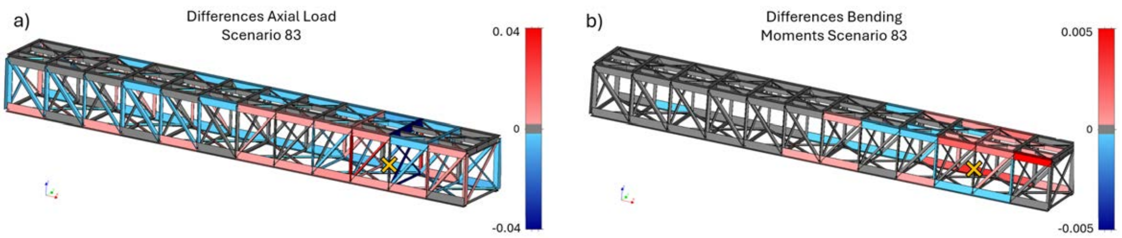

Fig. 155 | Difference of ΔIF indicators for DS 83: a) Axial load differences between 80 kN and collapse load b) Bending moments differences between 80 kN and collapse load.

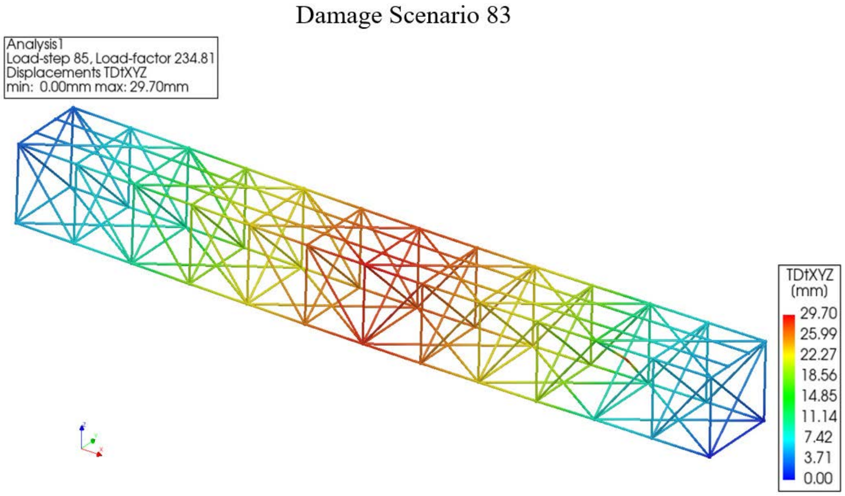

Fig. 156 | Displacements at collapse load for DS 83.

3.4.10 Damage Scenario 84

In the Type II vertical scenario (DS 84), the collapse load is reached at 171.69 kN, significantly lower than in the Type I vertical scenario. The load increase leads to the propagation of the initial failure, triggering the buckling of a vertical bracing. After this buckling, the model continues to increase its load until the adjacent vertical on the same side (towards midspan) begins to buckle. This buckling induces model unloading and subsequent numerical divergence (Fig. 159). Similar to the previous case, this scenario does not exhibit significant stiffness loss or major morphological changes. The primary differences lie in the altered behaviour of certain horizontal bracings and, most notably, in the analogous vertical to the removed element, which transitions from an unloading to loading condition (Fig. 157and Fig. 158). Regarding bending moments, the main difference is a localised and slight increase in the moments experienced by the chords around the removed vertical. The key ALP changes are detailed below:

- The buckling of the vertical bracing reduces the internal forces in the other vertical bracing within the same plane without altering its behaviour (it still increases its load in the collapse ALP). The unloading of the buckled element also decreases the forces in the upper and lower transversal beams. As a result, one of the transversal beams transitions from loading state in the 80 kN to an unloading state at collapse load. The redistributed load is primarily transferred to the analogous vertical on the undamaged side, causing it to change from an unloading state to a loading state in the collapse load scenario. The buckling in the adjacent vertical towards midspan (on the same side as the initial failure) ultimately leads to model divergence.
- The variations between both cases are very similar to those observed in the previous scenario. The most notable change is an increase in bending moments in the area where the vertical was removed, associated with the activation of the Vierendeel mechanism already present at 80 kN. However,

these variations are of low magnitude, resulting in only a slight increase in internal forces without significant morphological differences.

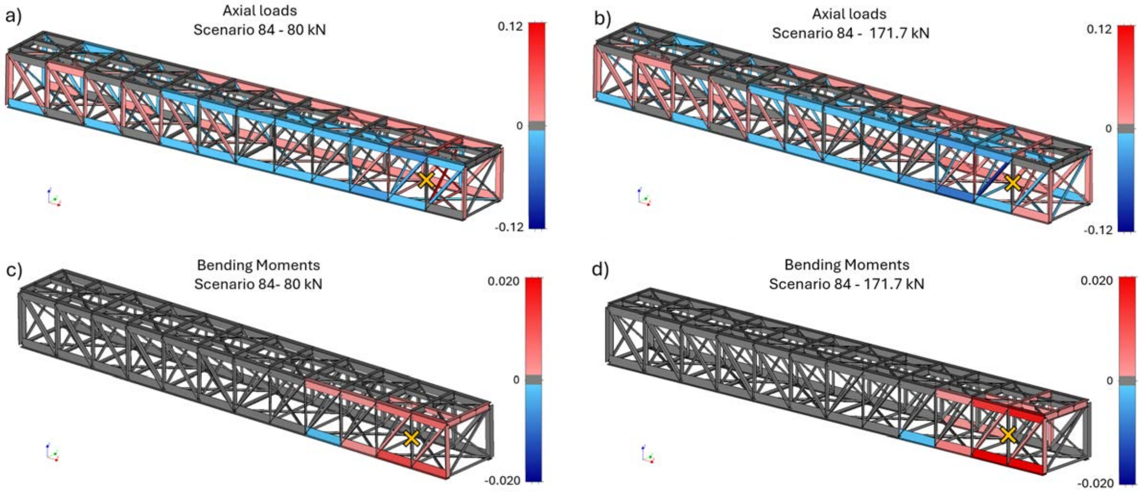

Fig. 157 | ΔIF indicators for DS 84: a) Axial load at 80 kN, b) Axial load at collapse load, c) Bending moments at 80 kN, d) Bending moments at collapse load.

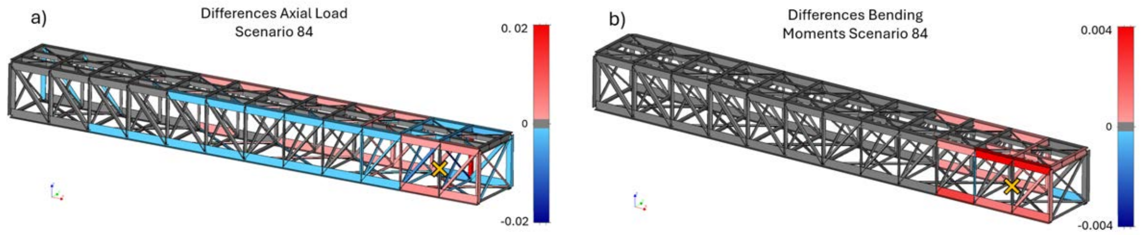

Fig. 158 | Difference of ΔIF indicators for DS 84: a) Axial load differences between 80 kN and collapse load b) Bending moments differences between 80 kN and collapse load.

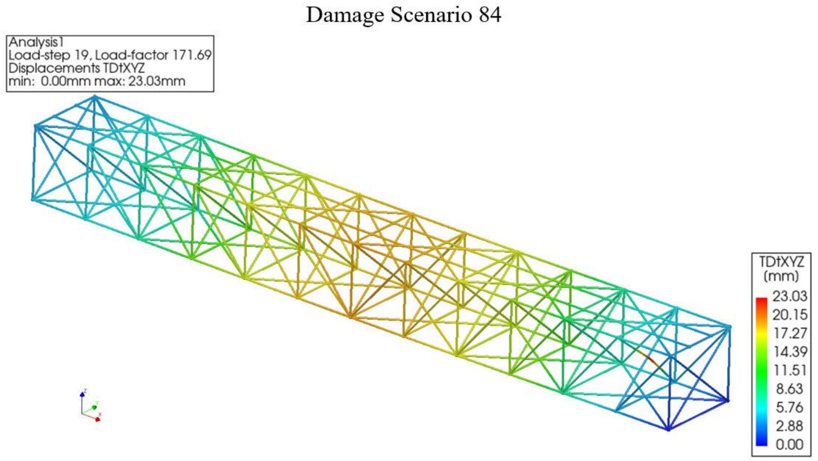

Fig. 159 | Displacements at collapse load for DS 84.

Section 3.5: Discussion of results

3.5.1 Main Effects

After a detailed analysis of the variations in the ALPs at different load levels until reaching the ultimate or collapse load of the structure, it can be concluded that the equilibrium paths studied for the different damage

scenarios show that the vertical deflection of the structure remains nearly linear until failure propagation begins due to the buckling of vertical members. However, the observed variations in the IF with respect to the initial loading state (80 kN) present several specific characteristics that influence the structural response:

1. **Non-proportional evolution of IFs with the load level.** The structural response, in terms of axial forces and bending moments in the structural members, does not increase proportionally with the applied load on the bridge. This is due to the presence of irregularities caused both by the initial failure and by the subsequent damage propagation.
2. **Morphological changes in the ALP:** Several morphological changes can be observed in the 10 DSs due to successive stiffness losses as failure propagates throughout the structure. As elements fail, load transfer mechanisms are progressively activated, which can be identified in the ALPs analysed in Section 2. As a result, there is significant interaction between different load-resisting mechanisms and ALPs, which are activated to enable load redistribution and prevent global structural collapse.

Overall, stiffness degradation, with displacements that are not proportional to the applied load, occurs at load levels significantly higher than the service load. However, locally, the individual response of structural members near the initial failure can be highly nonlinear. This is particularly evident in the failure scenario of vertical type X (ID 84), where one of the chords and one of the diagonals exhibit significant differences in their colour distribution, clearly indicating a non-proportional increase in their internal axial force (see Fig. 160). This nonlinear behaviour is primarily concentrated in areas near the initial failure or its propagation.

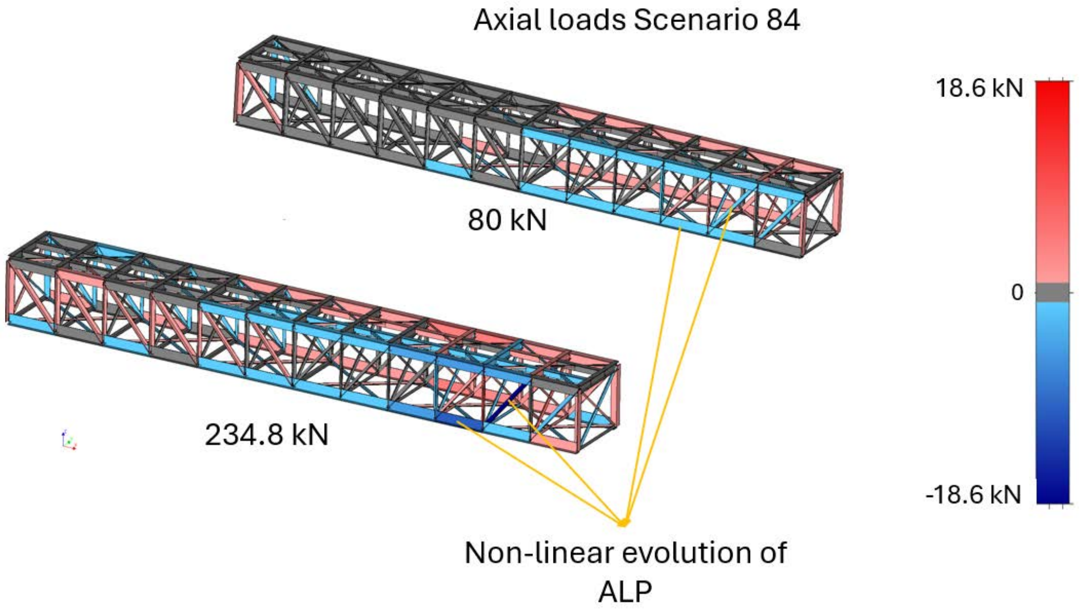

Fig. 160 | Example of ALP evolution due to the increasing load.

The second effect is the morphological changes in the ALP, which result from successive stiffness losses in the structure due to the progressive failure of elements (failure propagation). This progressive loss leads to a redistribution of internal forces, altering the structural role of certain elements during the development of the ALPs. In Section 2, different ALPs were identified for DSs involving the removal of a single element of a given type. However, when failure propagation involves multiple types of elements, the ALPs that are activated become multiple, diverse, and operate simultaneously, making their interpretation more complex. In the vicinity of each failed element, a significant increase in bending moments can be observed, while at

a global level, changes in internal axial forces reflect the combined effect of multiple ALPs activated due to the progressive failure of different elements.

A notable example can be found in the DS corresponding to chord Type II (DS 4), where the propagation of damage caused the adjacent verticals and diagonals to experience reverse loading—transitioning from elements that were unloading at 80 kN to elements that increase their load at collapse load conditions (see Fig. 161). Additionally, the horizontal bracings (both lower and upper) located farther from the damage scenario, which were inactive at 80 kN, develop loading and unloading processes in the collapse load condition.

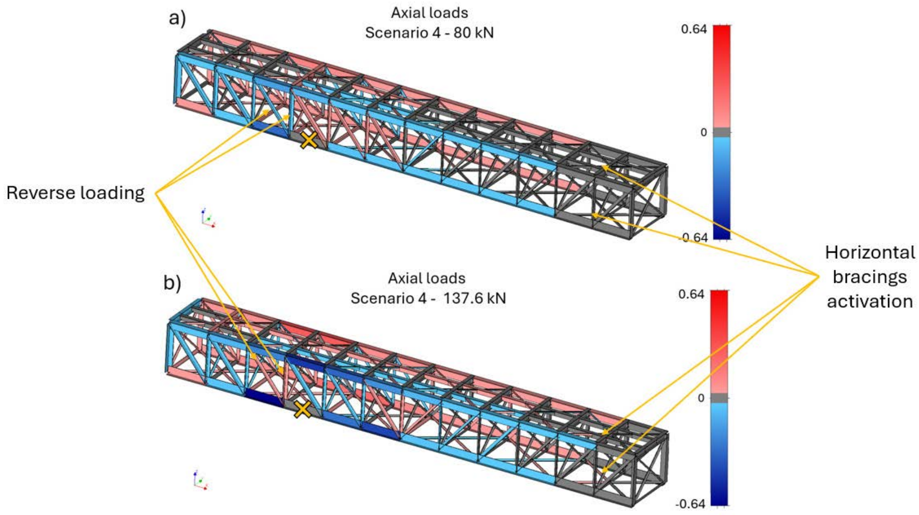

Fig. 161 | Example of morphological changes in DS involving the initial failure of a bottom chord Type II (DS 4).

3.5.2 Defence mechanisms at collapse load

To assess the structural defence mechanisms against collapse loading, the load factors corresponding to the buckling of the first element and the maximum load factor reached by the structure for each DS are evaluated. The load factor is defined as the applied load (in kN) divided by the operational load level (80 kN). This factor represents the multiplier required to bring the bridge to collapse after different initial failures defined by the DSs. It is worth mentioning that the load factors reached in the scaled-down bridge are representative of those in the real-scale bridge. Fig. 162 presents the normalized load factors (relative to the operational load) versus midspan displacement for the 10 scenarios analysed. Additionally, Table 4 provides the numerical values of the normalized load factor at the onset of the first element's buckling, as well as the maximum normalized load factor attained.

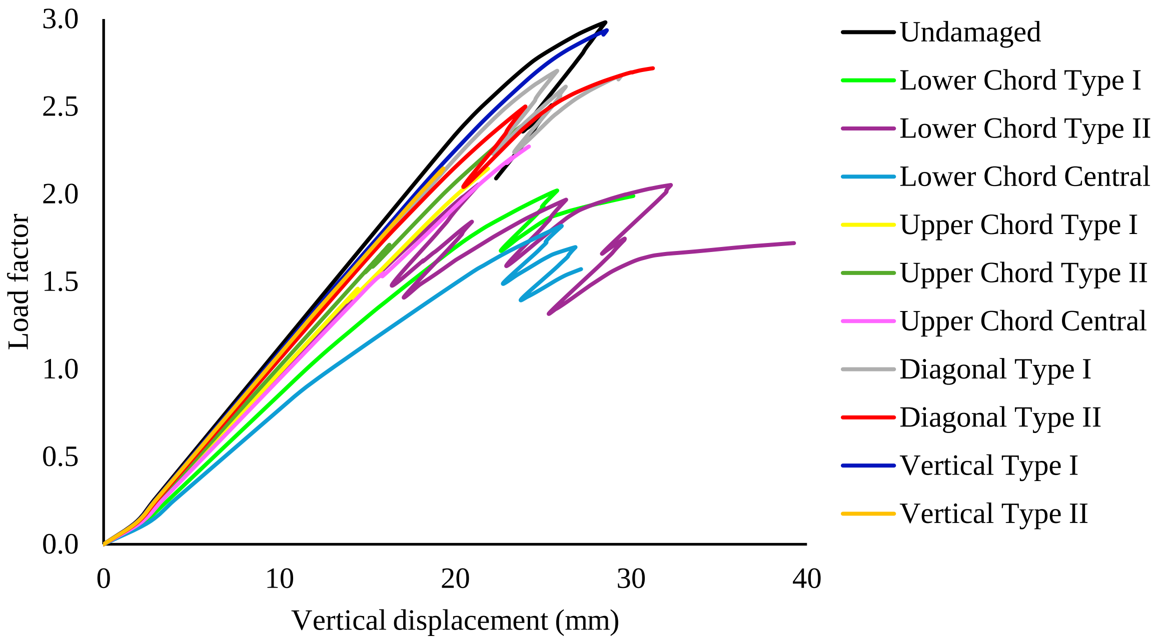

Fig. 162 | Equilibrium path of displacements in midspan for the 10 damage scenarios.

Table 4 | Load factors for the 10 damage scenarios.

Removed element in the DS	Scenario ID	Load factor 1st buckling	Maximum Load factor
Undamaged	0	2.98	2.98
Lower Chord Type II	4	2.05	2.05
Lower Chord Type I	5	2.02	2.02
Lower Chord Central	6	1.82	1.82
Upper Chord Type II	28	1.71	2.44
Upper Chord Type I	29	1.46	2.15
Upper Chord Central	30	1.55	2.27
Diagonal Type II	59	2.5	2.72
Diagonal Type I	60	2.7	2.7
Vertical Type I	83	1.11	2.93
Vertical Type II	84	1.11	2.14

These load factors, together with the curve described by the equilibrium path for each DS, allow for the classification of four categories of DSs based on their ability to redistribute loads and withstand successive structural failures of different elements:

1. Scenarios where the collapse occurs abruptly, without oscillations in the equilibrium path curve. This occurs because the structure is unable to sustain the propagation of the initial failure through primary members, which significantly affect the equilibrium path: DSs 28, 29, 30, 83, and 84.

2. Scenarios where damage progresses, leading to load-unload cycles that degrade the global stiffness of the bridge, causing displacements that are clearly non-proportional to the applied loads due to the propagation of the initial failure through primary elements. This group can be subdivided into three categories based on the load factors attained after the buckling of the first element:
   a) Scenarios where the structure redistributes loads after damage propagation in primary elements but always reaches lower load factors than those at the first buckling: DS 6.
   b) Scenarios where the structure redistributes loads after damage propagation in primary elements and reaches load factors similar to those attained at the first buckling: DSs 4, 5, and 60.
   c) Scenarios where the structure redistributes loads after damage propagation in primary elements and reaches higher load factors than those at the first buckling: DS 59 (Diagonal).

In Group 1, which includes scenarios involving the upper chords and verticals (DSs 28, 29, 30, 83, and 84), low-intensity load-unload cycles occur due to the buckling of secondary elements such as horizontal bracings (in the case of upper chords) or vertical bracings (in the case of verticals). In upper chord scenarios, the buckling of the horizontal bracings causes a slight reduction in the applied load. However, these loads are successfully redistributed, allowing the model to reach load factors 40% to 50% higher than the load that caused the first buckling. In vertical scenarios (DSs 83 and 84), the vertical bracings buckle at a low load (89 kN). Fig. 163 and Fig. 164 present the equilibrium path along with the failure propagation sequence indicated by red points. These figures illustrate that the buckling of the vertical bracing does not cause a reduction in the applied load (Point 1 in Fig. 163 and Fig. 164). Despite this failure (which involves a low-stiffness element), the bridge continues to increase its applied load until collapse. The load increments in both scenarios relative to the first buckling load (89 kN) are 93% and 164% for DSs 83 and 84, respectively. In DS 83, the increase in load triggers the buckling of a vertical (see Point 2 in Fig. 163). Once this buckling initiates, the model loses load until divergence. In DS 84, failure propagates through the adjacent vertical towards the midspan, also leading to a loss of applied load until the model fails to converge (see Point 2 in Fig. 164). The changes in these ALPs are primarily driven by the non-proportional evolution of IF with respect to the applied load, as primary elements (chords, diagonals, and verticals) are not directly involved in the failure propagation. Nevertheless, minor changes are observed in the vicinity of the buckled vertical bracings, whose loads are transferred to adjacent elements.

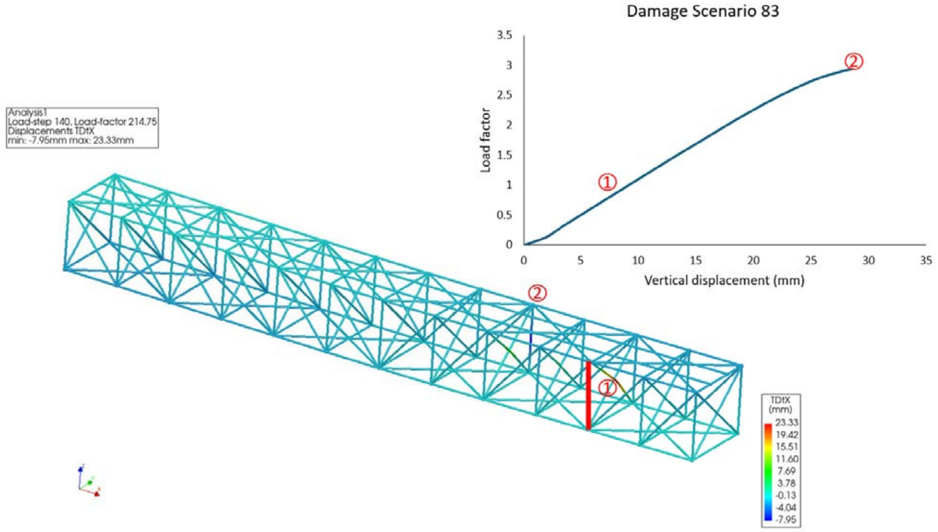

Fig. 163 | Failure propagation in DS 83.

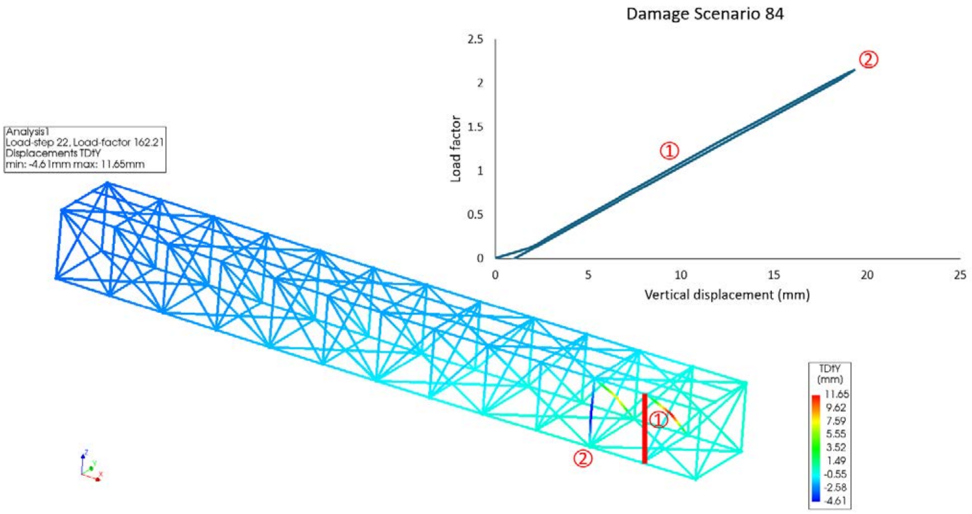

Fig. 164 | Failure propagation in DS 84.

Groups 2a and 2b represent groups where the maximum load factor is not increased after the first buckling. In Group 2a, the central chord scenario (DS 6) is identified. Fig. 165 presents the equilibrium path along with the failure propagation sequence indicated by red points. In this scenario, the maximum load is reached before the buckling of any primary structural element of the bridge. The increase in load leads to the buckling of one of the verticals on the undamaged side (see Point 1 in Fig. 165). Consequently, the adjacent vertical is activated, taking on the redistributed load. As the structure attempts to reach the same load level, this second vertical also buckles (Point 2 in Fig. 165). After this second buckling, although load redistribution occurs, the ultimate load is lower than the previously attained maximum, leading to collapse. The loss of this chord makes this the scenario with the lowest maximum load, highlighting the limited effectiveness of the activated ALPs in achieving a higher load-bearing capacity, despite having surpassed the buckling of a primary structural element (vertical). The main changes observed in the ALPs are related to both their evolution with load and their morphology, as failure propagation through primary elements significantly alters their behaviour.

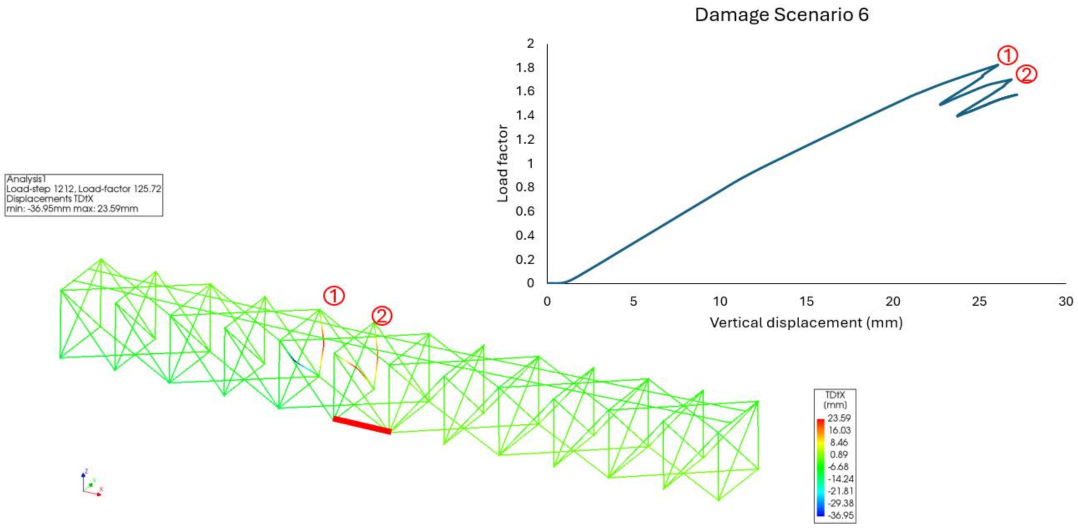

Fig. 165 | Failure propagation in DS 6.

In Group 2b, the failure scenarios of the other two lower chords (DSs 4 and 5) and the diagonal Type I (DS 60) are included. In these cases, load increases lead to the failure of primary elements (verticals). Following

these failures, the bridge successfully redistributes its loads due to specific morphological changes in the ALP. The following figures (Fig. 166, Fig. 167 and Fig. 168) illustrate the damage propagation sequence for the three DSs in this group. As observed, in some cases, the morphological transformation of the ALP facilitates load redistribution, but at the cost of subsequent element failures at significantly lower loads, as seen in Point 2 of the equilibrium path for DSs 4 and 60 (Fig. 166 and Fig. 168). Conversely, in other instances, the ALP transformation allows for effective load redistribution, enabling the bridge to withstand higher loads than those causing previous propagated failures (and similar to the maximum load), as demonstrated in Points 3 and 4 of DS 4 (Fig. 166) and the ultimate load of DS 60 (Fig. 168). For DS 5, after the buckling of the vertical bracing, the bridge redistributes the loads and reaches a load level close to the one that triggered the previous failure. However, collapse occurs before surpassing this value (ultimate load in Fig. 167). The ALPs developed in these scenarios exhibit both a strong evolution and substantial morphological changes. However, the structure is unable to activate any ALP that allows it to exceed the load of the first failure following the initial DS-induced failure.

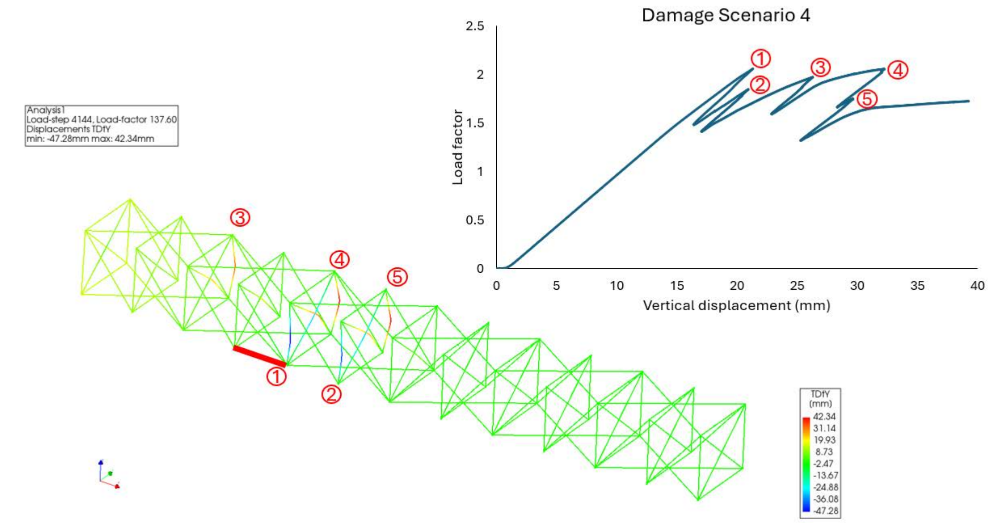

Fig. 166 | Failure propagation in DS 4.

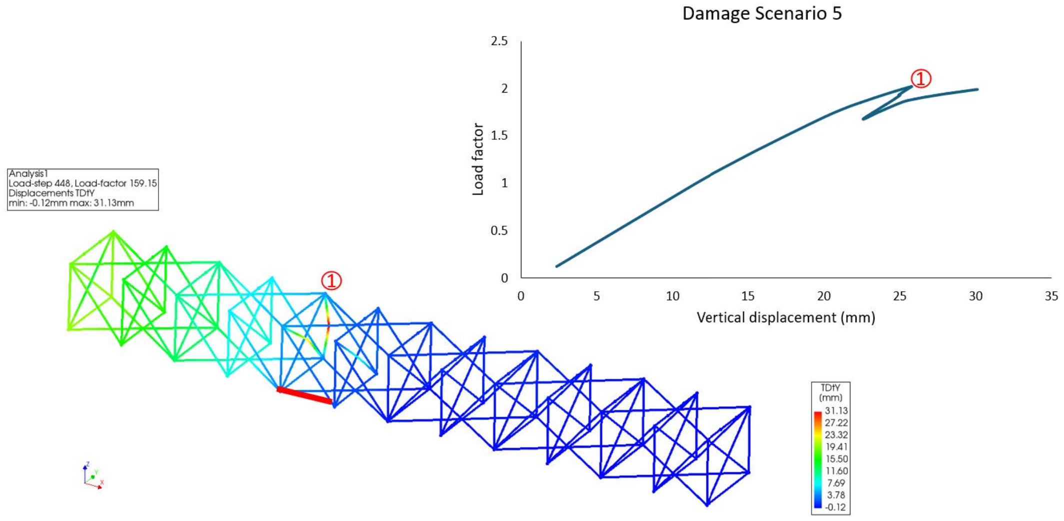

Fig. 167 | Failure propagation in DS 5.

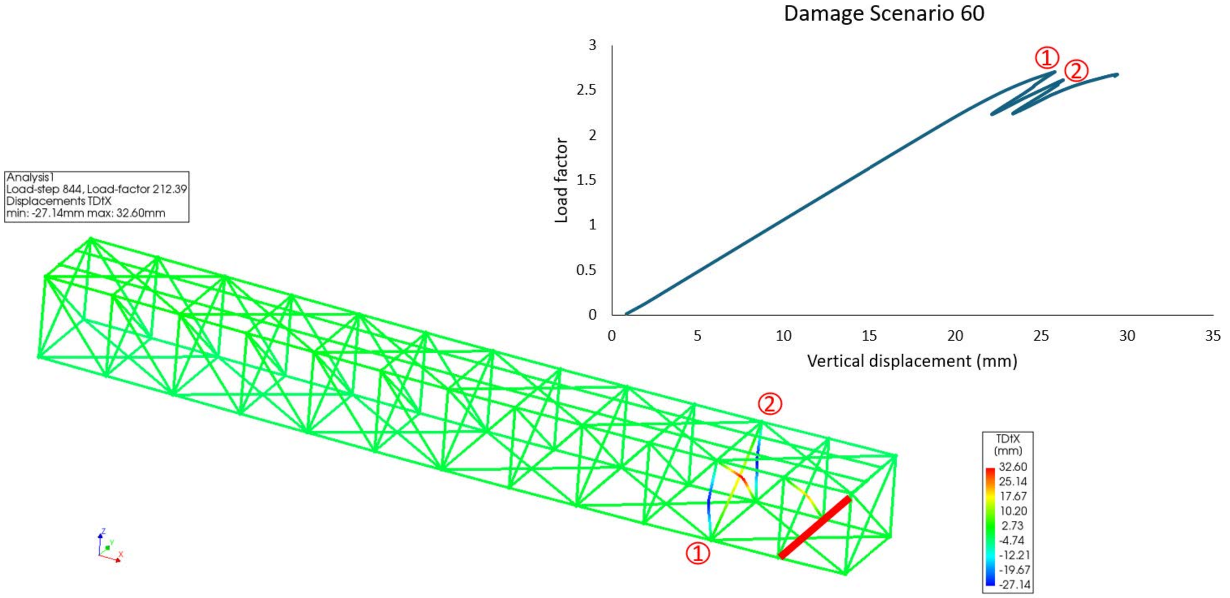

Fig. 168 | Failure propagation in DS 60.

In Group 2c, the failure scenario of the Type II diagonal (DS 59) is analysed where Fig. 169 illustrates the damage propagation sequence. In this case, load increases lead to the buckling of one of the adjacent verticals on the undamaged side (see Point 1 in Fig. 169). Following this buckling, the structure is subjected to morphological changes in the ALP, where the load of the buckled vertical is transferred to the two adjacent verticals on the same side. This redistribution mechanism allows the structure to reach loads 8.8% higher than the load that triggered the initial buckling (see ultimate load in Fig. 169), making this the second-highest maximum load scenario. The main differences between the ALP developed in DS 59 and that in DS 60 lie in the direction of damage propagation. In DS 60, the damage spreads on the same side where the diagonal was removed, whereas in DS 59, the failure propagates on the opposite side. Additionally, in this scenario, the buckling of the vertical induces a generalized unloading effect across the entire transverse plane. This means that the adjacent vertical on the damaged side unloads, despite not buckling itself, preventing what would likely have been the next local failure. In this case, the ALP developed by the structure effectively redistributes the loads beyond the initial failure load. The balance between stiffness on both sides of the bridge (both sides being damaged symmetrically due to damage propagation) results in a homogeneous structural response. Moreover, the Vierendeel effect, which increases bending moments across the damaged area, has enhanced the load transmission mechanism. As a result, the loads on the unloaded transverse-plane verticals are redistributed across the adjacent verticals and diagonals, allowing them to act as a single homogeneous beam system.

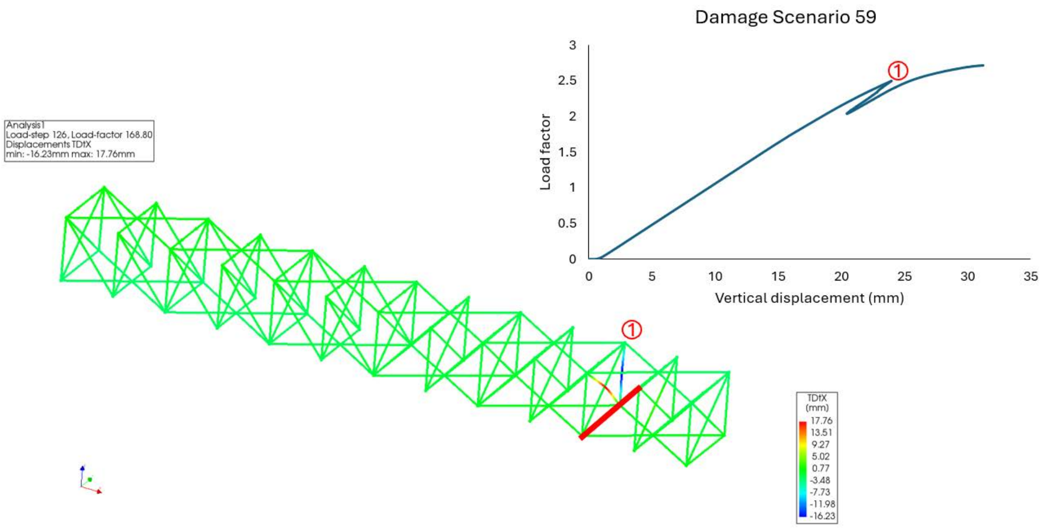

Fig. 169 | Failure propagation in DS 59.

3.5.3 Summary of Conclusions

In summary:

- The defensive mechanisms of the bridge evolve with increasing load.
- This evolution is governed by **multiple factors**, primarily depending on **how failure propagates in each DS**. Key observations include:
  - The main morphological changes in the ALP are driven by the failure propagation through primary and bracing system elements; however, it is the failure of the primary elements that ultimately leads to structural collapse.
  - More complex ALPs appear when failure propagates through successive failures of primary structural elements (chords, verticals, and diagonals). In these cases, the bridge effectively redistributes loads, and the developed ALPs can be interpreted as a combination of the individual ALPs previously characterized in Section 2 for each type of failure. For instance, if failure propagates from the chords to the verticals, the ALPs observed will resemble the superposition of ALPs identified for chord failures and vertical failures.
- The bridge exhibits **significant redundancy**, demonstrated by the higher load factors achieved in the 10 DS and the fact that **a considerable number of elements must fail before the entire structural system becomes unstable**. It is worth mentioning that the load factors reached in the scaled-down bridge are representative of those in the real-scale bridge.
- At the global level, the bridge exhibits a ductile load-displacement response in certain cases, while in others, collapse occurs with minimal evidence of nonlinear behaviour in their equilibrium path graph.
- Surprisingly, in some cases, the bridge withstands higher loads even during failure propagation.

It is important to note that the prominence of certain ALPs, and consequently of certain secondary resistance mechanisms, may vary for bridges having a different global slenderness (e.g. for bridges with longer spans) or different boundary conditions (e.g. hyperstatic spans). This will particularly affect the relative importance of secondary resistance mechanisms that involve the global torsion and the global in-plane hinged rotation of the entire bridge span (see main text section 'The first line of defence') after specific component failures.

References

1. Losanno, D., Galano, S. & Parisi, F. Influence of strand rupture on flexural behavior of reduced-scale prestressed concrete bridge girders with different prestressing levels. *Eng. Struct.* **301**, 117358 (2024).
2. Dinu, F., Marginean, I., Dubina, D. & Petran, I. Experimental testing and numerical analysis of 3D steel frame system under column loss. *Eng. Struct.* **113**, 59–70 (2016).
3. Kong, D.-Y., Yang, Y., Li, S., Yang, B. & Liew, J. Y. R. Experimental and Analytical Study on Progressive Collapse of 3D Composite Floor System under Corner Column Loss. *J. Struct. Eng.* **148**, 04022012 (2022).
4. Z. P. Bazant. Size effect on structural strength: a review. *Arch. Appl. Mech.* **69**, 703–725 (1999).
5. EN ISO 6892-1. Metallic materials. Tensile testing. Part 1: Method of test at room temperature. (2009).
6. Buitrago, M., Bertolesi, E., Calderón, P. A. & Adam, J. M. Robustness of steel truss bridges: laboratory testing of a full-scale 21-metre bridge span. *Structures* **29**, 691–700 (2021).
7. Coutinho, C. P., Baptista, A. J. & Dias Rodrigues, J. Reduced scale models based on similitude theory: A review up to 2015. *Eng. Struct.* **119**, 81–94 (2016).
8. Goodier, J. N. & Thomson, W. T. *Technical Report NACA TN-933. Applicability of similarity principles to structural models*. (1944).
9. Computational Mechanics Department of TNO Building and construction research institute, “DIANA FEA BV Documentation.” Delft, The Netherlands, 2003, [Online]. Available: https://dianafea.com.
10. BS5400-3:2000 British Standard. Steel, concrete and composite bridges - Part 3. Code of practice for design of steel bridges. BSI 2006.
11. Bracing system theory and design for I-girders and tub girders (Chapter 13 in Steel Bridge Design Handbook). American Institute of Steel Construction (AISC), 2022.
12. Parke, G. A. R. & Harding, J. E. *Design of steel bridges in ICE Manual of Bridge Engineering*. (Thomas Telford, 2008).
13. Hsu, Y. T. EBEF method for distortional analysis of steel box girder bridges. *J. Struct. Eng.* **121**, 557–566 (1995).
